# Extracorporeal photopheresis for the treatment of steroid-refractory immune related adverse events from immune checkpoint inhibitor therapy: a multi-center phase Ib/II clinical trial

**DOI:** 10.1038/s41467-026-78897-9

**Published:** 2026-10-23

**Authors:** Roxane Riemer, Lukas M. Braun, Sophie Giesler, David Rafei-Shamsabadi, Sebastian Haferkamp, Alina Hartmann, Wolfgang Melchinger, Matthias Weiss, Stefanie Birth, Ulrike Philipp, Inga Gebhard, Carsten Schwänen, James A. Hutchinson, Nikolaos Karantzelis, Till Wallrabenstein, Theresa Lowinus, Niklas Brehm, Justyna Rawluk, Dietmar Pfeifer, Natascha Osswald, Peter Bronsert, Manuel Rogg, Grigor Andreev, Melanie Boerries, Maximilian Lammer, Van Anh Nguyen, Matthias Schmuth, Natalie Köhler, Justus Duyster, Gabriele Ihorst, Philip Keye, Susana Minguet, Dirk Schadendorf, Selma Ugurel, Robert Thimme, Peter Hasselblatt, Erika L. Pearce, Burkhard Becher, Carolin Funke-Lorenz, Florian Scherer, Bertram Bengsch, Christoph Schell, Jan-Malte Placke, Frank Meiss, Petya Apostolova, Robert Zeiser

**Affiliations:** 1 Department of Internal Medicine I, Medical Center—University of Freiburg, Faculty of Medicine, University of Freiburg, Freiburg, Germany; 2 German Cancer Consortium (DKTK), Partner site Freiburg, a partnership between DKFZ and Medical Center—University of Freiburg, Freiburg, Germany; 3 Department of BioMedical Research, Tumor Immunology, University of Bern, Bern, Switzerland; 4 Department of Medical Oncology, Inselspital, Bern University Hospital, University of Bern, Bern, Switzerland; 5 Department of Dermatology, Medical Center—University of Freiburg, Faculty of Medicine, University of Freiburg, Freiburg, Germany; 6 Department of Dermatology, University Hospital Regensburg, Regensburg, Germany; 7 Division of Hematology, Oncology, Palliative Care, Ortenau Hospital Offenburg-Kehl, Offenburg, Germany; 8 Department of Surgery, University Hospital Regensburg, Regensburg, Germany; 9 Department of Medical Oncology and Hematology, University Hospital of Zurich, Zurich, Switzerland; 10 Center for Hematology and Oncology, Kantonspital Baselland, Bruderholz, Switzerland; 11 Institute of Surgical Pathology, Medical Center—University of Freiburg, Faculty of Medicine, University of Freiburg, Freiburg, Germany; 12 Institute of Medical Bioinformatics and Systems Medicine (IBSM), Medical Center—University of Freiburg, Faculty of Medicine, University of Freiburg, Freiburg, Germany; 13 Department of Dermatology, Venereology and Allergy, Medical University Innsbruck, Innsbruck, Austria; 14 CIBSS—Centre for Integrative Biological Signalling Studies, University of Freiburg, Freiburg, Germany; 15 Clinical Trials Unit, Medical Center—University of Freiburg, Faculty of Medicine, University of Freiburg, Freiburg, Germany; 16 Eye Center, Medical Center—University of Freiburg, Faculty of Medicine, University of Freiburg, Freiburg, Germany; 17 Signalling Research Centres BIOSS and CIBSS, Freiburg, Germany; 18 Department of Synthetic Immunology, Faculty of Biology and Centre for Chronic Immunodeficiency (CCI), Faculty of Medicine, University of Freiburg, Freiburg, Germany; 19 Department of Dermatology, University Hospital Essen, University of Duisburg-Essen, Essen, and German Cancer Consortium (DKTK), Partner Site Essen/Duesseldorf, Essen, Germany; 20 National Center for Tumor Diseases (NCT)-West, Campus Essen, & Research Alliance Ruhr, Research Center One Health, University Duisburg-Essen, Essen, Germany; 21 Klinikum Bielefeld Rosenhöhe, Department of Dermatology, Medical School and University Medical Center OWL, Bielefeld University, Bielefeld, Germany; 22 Department of Internal Medicine II (Gastroenterology, Hepatology, Endocrinology and Infectious Diseases), Medical Center—University of Freiburg, Faculty of Medicine, University of Freiburg, Freiburg, Germany; 23 The Bloomberg-Kimmel Institute for Cancer Immunotherapy, Department of Oncology, Johns Hopkins School of Medicine, Baltimore, MD USA; 24 Institute of Experimental Immunology, University of Zurich, Zurich, Switzerland; 25 Signalling Research Centres BIOSS and CIBSS—Centre for Integrative Biological Signalling Studies, University of Freiburg, Freiburg, Germany; 26 Department of Biomedicine, Faculty of Medicine, University Hospital Basel and University of Basel, Basel, Switzerland; 27 Division of Hematology, University Hospital Basel, Basel, Switzerland

**Keywords:** Translational research, Cancer immunotherapy

## Abstract

Severe immune-related adverse events (irAE) in cancer patients receiving immune checkpoint inhibitors (ICI) require treatment interruption and immunosuppression. Based on prior preclinical data, in this prospective, single arm, non-randomized phase-Ib/II clinical trial we test the activity of Extracorporeal Photopheresis (ECP) against steroid-refractory irAEs in 30 patients (EudraCT-No.:2021-002073-26). The primary endpoint is the evaluation of the safety of ECP treatment for patients with ICI-induced irAEs. The study meets its pre-specified primary endpoint of less than 50% of patients developing ECP-related severe adverse events (SAE). No ECP-related SAE is observed and ECP-related AEs are reported in 20% (6/30) of patients. Overall response rate for all irAEs is 96.3% (CI 81.0–99.91). Colitis-, hepatitis- and dermatitis-specific response rates are 100% (13/13), 86.7% (13/15) and 100% (3/3), respectively. Secondary endpoints include the assessment of response rate of irAE and overall survival. ECP therapy is associated with reduced granzyme-A/B/K, T-bet, interferon-γ and TNF in peripheral T cells and pro-inflammatory gene-expression in the intestinal tract. The 12-month overall survival rate is 84.3% (CI 63.4%-93.8%). Response correlates with a decrease in inflammatory gene expression in the intestinal tract. These results suggest that ECP is a safe treatment option for SR-irAEs and warrant further investigation in subsequent randomized clinical trials.

## Abstract (web summary)

Cancer patients receiving immune checkpoint inhibitor (ICI) therapy develop immune-related adverse events. Here, the authors report the results of a multi-centre prospective phase Ib/II clinical trial evaluating the safety and efficacy of extracorporeal photopheresis (ECP) therapy, showing ECP is a safe therapeutic option that could benefit the treatment of ICI-related inflammatory syndromes.

## Introduction

Immune checkpoint inhibitor (ICI) therapy has significantly improved the response rates and overall survival of patients with various cancer entities^1–3^. However, immune-related adverse events (irAEs) in cancer patients receiving ICI therapy may lead to treatment interruption and immunosuppression^4,5^. IrAEs may affect any organ system and most commonly involve the gastrointestinal tract, endocrine system, and the skin^6,7^.

Treatment of irAEs is based on treatment recommendations by ESMO and ASCO according to the severity grade^8,9^. ESMO and ASCO clinical practice guidelines are widely used frameworks for managing ICI toxicities, but they are not the only available sources of guidance^10^. Standardized first-line therapy of irAEs grade ≥2 includes treatment interruption and administration of glucocorticoids (GC)^8,9^. The reduction of anti-tumor immunity by GCs has been shown in various pre-clinical models^11–13^. In agreement with this observation, several retrospective studies showed an inferior overall survival (OS) and progression-free survival (PFS) in patients treated with GCs for irAEs^14–17^. This observation was made with GCs dosages over 10 mg/d^14,15,17^ and early GCs use^16^, while in other situations the impact of GC on PFS is controversial. Besides their potential negative impact on anti-tumor immunity, GCs can cause various side effects, including an increased risk for infections and metabolic and cardiovascular side effects^18,19^. Second-line therapies are associated with further side effects and/or additional alteration of anti-tumor immunity. A systematic review showed an overall infection rate of 22.3% (231/1036) in patients treated with GCs alone or in combination with the anti-TNF antibody infliximab for irAEs^20^. Janus kinase inhibitors, mycophenolate mofetil (MMF) and cyclophosphamide are associated with an increased risk for infection^21,22^ and gastrointestinal adverse events^22^. Calcineurin inhibitors can cause neurotoxicity^23^. Therefore, it would be desirable to treat irAEs with second-line therapies causing fewer toxicities. An inferior OS upon administration of an anti-TNF antibody plus GCs compared to GCs alone for steroid refractory (SR)-irAEs has been reported^24,25^. However, the relationship between TNF blockade and clinical outcomes is likely influenced by highly context-dependent variables that warrant further investigation. The currently available literature is not definitive. Infliximab and the gut-selective anti-α4β7-integrin antibody vedolizumab are frequently used as second-line immunosuppressive therapies for ICI-colitis with response rates up to 89%^26–29^.

Consequently, there is a crucial need for safer and more efficient therapeutic options for irAEs that allow to maintain anti-tumor immunity.

Extracorporeal photopheresis (ECP) is an immune-modulatory cellular therapy^30^ with a low rate of side effects^31–34^. It is well established in the treatment of graft-versus-host disease (GvHD), cutaneous T-cell lymphoma (CTCL), and sclerosing diseases^35–38^. ECP is available and in daily administration at the large majority of University medical centers in the USA and Europe for treatment of CTCL, organ transplant rejection, and post-allo-transplant GVHD. Moreover, our prior work and the work by others indicate high clinical activity of ECP for SR-irAEs mainly in retrospective case studies^31,32,39,40^.

The hypothesis to be tested is that ECP is a safe and efficient treatment option for patients with steroid-refractory irAEs following ICI therapy. Here we report the results of a prospective phase Ib/II clinical trial to evaluate the efficacy and safety of ECP for SR-irAEs. The objective is the evaluation of the rate of treatment-related adverse events (AEs) and serious adverse events (SAEs) in patients treated with ECP, while one of the major secondary endpoints is assessing the efficacy of ECP in patients with irAEs. Treatment-related AEs are reported in 6/30 patients, which are mild and mostly resolving without any further treatment. The ORR is 96.3%, and pathogenic immune cell activation decreases during ECP. This positions ECP as a promising second-line treatment for SR-irAEs, which needs to be evaluated in a randomized trial.

## Results

### Patient characteristics and ECPs performed

All 30 patients included in the trial had at least one irAE ≥ grade 2, involving the colon, liver, or skin. The most common irAE was ICI-hepatitis (*n* = 17, 50.0%), followed by ICI-colitis (*n* = 14, 41.2%) and ICI-dermatitis (*n* = 3, 8.8%) (Supplementary Table 1). Four patients had two irAEs simultaneously, for which the irAE with the highest grade was used for ORR assessment (3/4 ICI-colitis and hepatitis, 1/4 ICI-dermatitis and colitis). 58.8% of patients had an irAE grade$\,\ge$ 3 prior to ECP therapy.

**Fig. 1:**
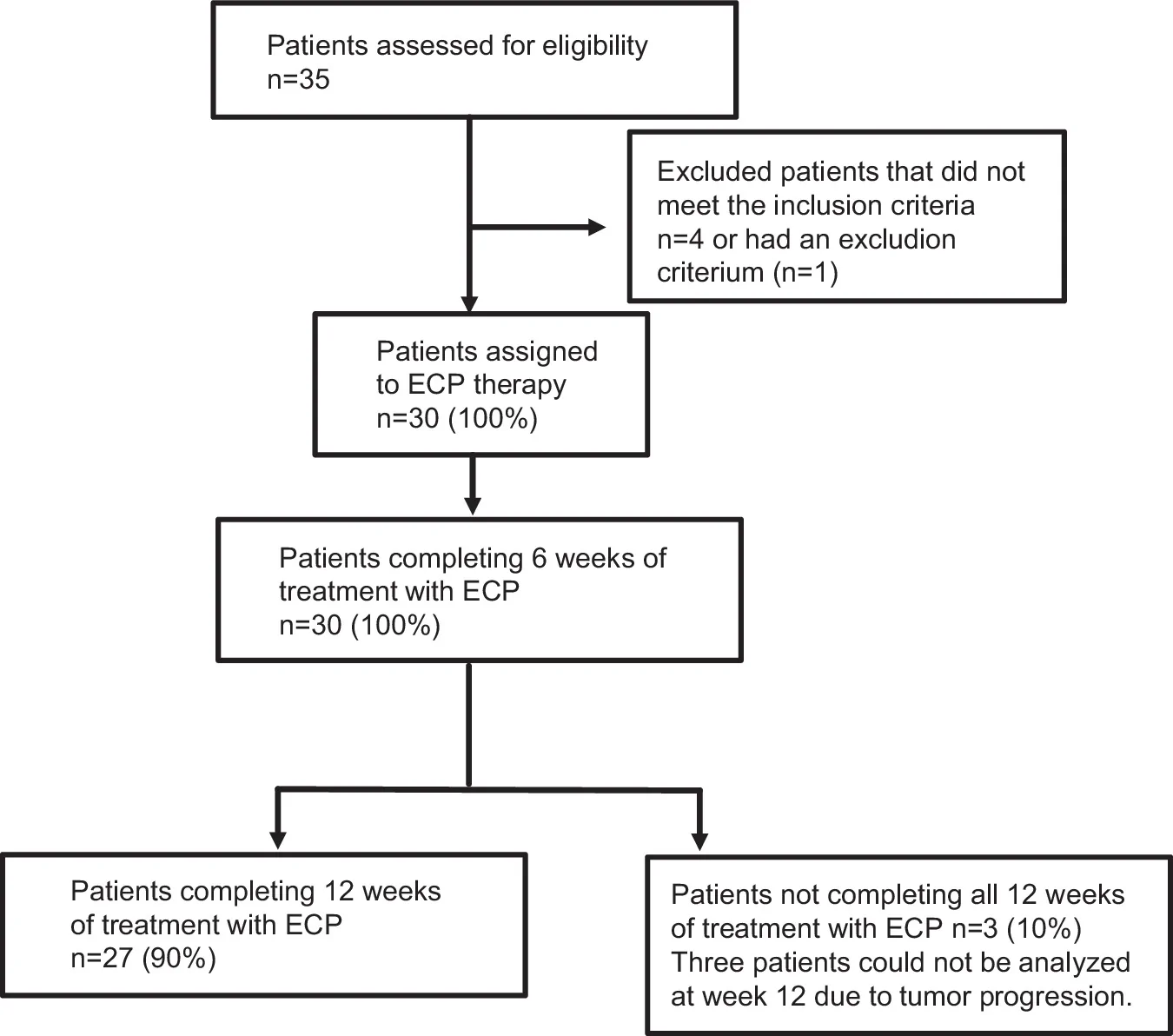
Study design flowchart. The study design flowchart displays the number of patients screened, the number of patients included, and patients completing versus not completing all 12 weeks of treatment with ECP.

The most common cancer entity was melanoma of the skin; other entities were non-small-cell lung cancer, small-cell lung cancer, thyroid cancer, uveal melanoma, pleural mesothelioma, Merkel cell carcinoma, adenocarcinoma of the breast and cancer of unknown primary (CUP) (Supplementary Table 1). Most commonly used ICI therapy was double ICI therapy with nivolumab/ipilimumab (40%). Patient characteristics and pre-treatments are summarized in Supplementary Table 1. The full participant disposition can be found in the study design flowchart (Fig. 1). Three patients could not be analyzed at week 12 due to tumor progression (Fig. 1). Two of these patients (2/3) had a premature end of study (EOS) due to generalized tumor progression. One of these patients had a premature end of treatment (EOT) due to cerebral tumor progression after completing 12 × ECP. This patient reached CR of all irAEs and remained in CR at last follow-up in month 12. The median number of ECP interventions during the treatment period was 16 (9–18) (Supplementary Fig. 1A). The analysis was by original assigned groups, as all patients received ECP.

### Safety

The study met its pre-specified primary endpoint of less than 50% of patients developing ECP-related severe adverse events (SAE). The adverse events were reported by the treating physicians of the patients, and their relation to treatment was also judged by these physicians, which is according to Good Clinical Practice (GCP). No ECP-related SAE was observed. One hundred five AEs were documented (Supplementary Table 2). The number of patients with possibly ECP-related AEs were 6/30. ECP-related AEs included hypopigmentation (1/6), exanthema (1/6), photosensitivity (3/6), and anemia (1/6), of which 33.3% resolved completely. In our study, each SAE was prospectively recorded and reviewed in detail, including timing, clinical context, and concomitant conditions. Where possible, a clear alternative etiology was established. Ten SAEs were documented in six patients, which had no causal relationship with ECP. These SAEs included SARS-CoV2 infection, intestinal infection, cholestasis (2 events), gangrenous cholecystitis, hemorrhagic colitis, hemorrhagic shock, sepsis, and hemiparesis and seizures due to known brain metastases. All SAEs resolved completely. Individual AEs and SAEs are shown (Supplementary Table 3). SAE attribution to ECP or not was based on clinical evaluation. SAEs were reported to the responsible Pharmacovigilance Clinical Trial Unit according to protocol within 24 h after occurrence. Each AE and SAE was assessed for causal relationship to ECP by the investigator.

### ECP was associated with high irAE response rates

**Fig. 2:**
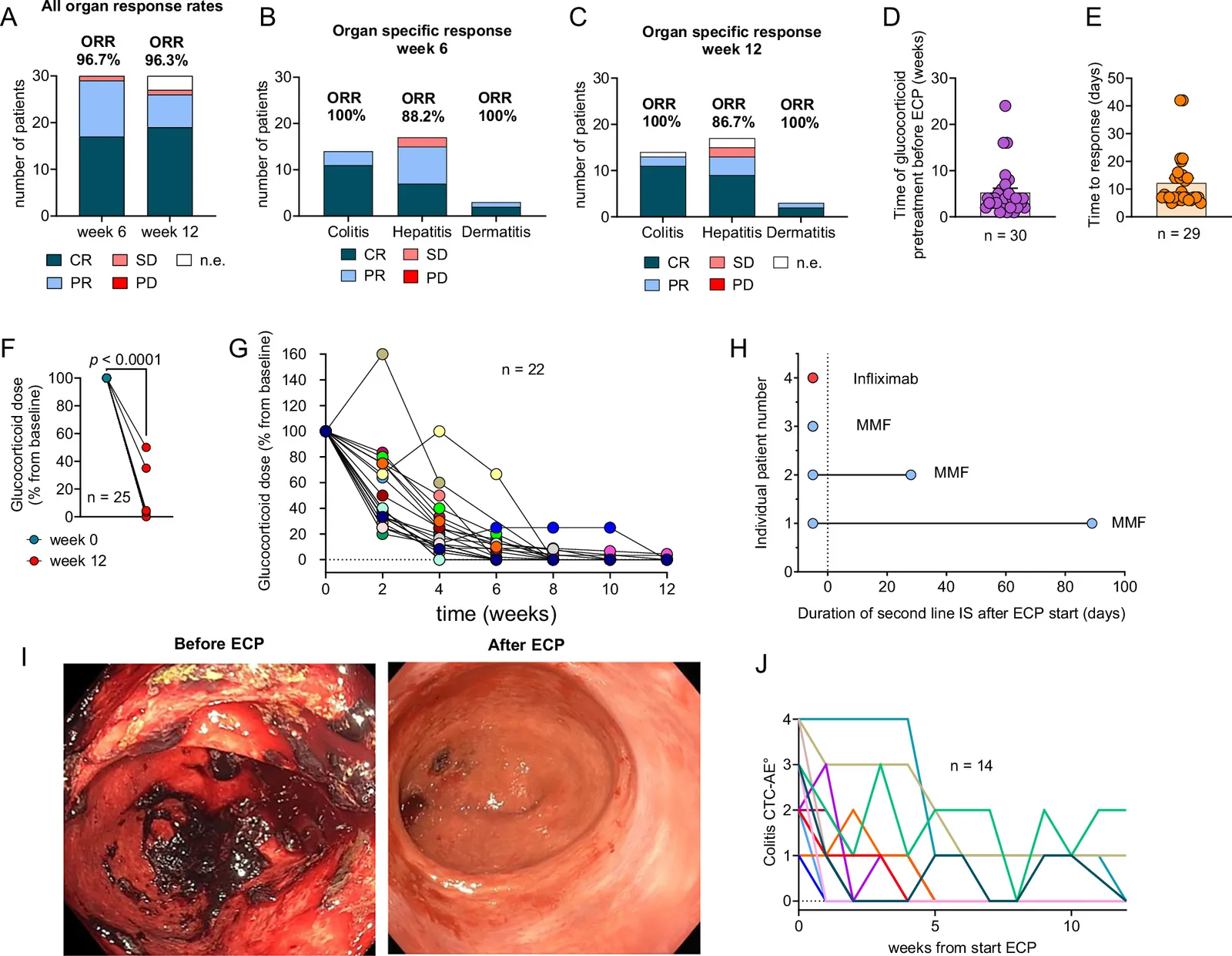
Clinical result of ECP in patients with steroid-refractory irAE. **A** irAE response assessment during (week 6) and after ECP treatment (week 12). Absolute numbers of patients with complete response (CR), partial response (PR), stable disease (SD), and progressive disease (PD). Three patients were not eligible (n.e.) for response assessment at week 12. Overall response rates (ORR) in percent are indicated above each bar. **B** Organ-specific response assessment of irAEs during ECP treatment at week 6, for ICI-colitis, ICI-hepatitis, and ICI-dermatitis. Absolute numbers of patients CR, PR, SD, and PD are shown. ORRs are indicated above each bar. **C** Organ-specific response assessment of ICI-colitis, ICI-hepatitis, and ICI-dermatitis at end of treatment (EOT) in week 12. Absolute numbers of patients with CR, PR, SD, or PD are shown. ORRs are indicated above each bar. Three patients were not eligible (n.e.) for response assessment at week 12. **D** Mean weeks (±SEM) of glucocorticoid pretreatment before ECP therapy initiation. Each dot represents one patient. In case of steroid dependency, defined as relapse of irAE after GC treatment was terminated, the times of individual GC applications were added together. **E** Time to response after ECP therapy in days (mean ± SEM). A response to therapy was counted if patients achieved a reduction of at least one CTC-AE grade of at least one organ system involved compared to the CTC-AE grade prior to the first ECP. Each dot represents one patient. **F** Relative reduction of GC as percentage from baseline after 12 weeks of ECP therapy (week 12) compared to the first day of ECP. Each dot represents an individual patient. Only patients with values in week 0 and 12 are shown (*n* = 25). Significance was calculated using a two-tailed one-sample Wilcoxon signed-rank test. **G** Relative reduction of GC dosage as percentage from baseline (first day of ECP treatment) over time in 22 of 30 patients. Each line and dot color represents an individual patient. Eight patients could not be analyzed due to different factors (*n* = 3 premature EOS/EOT, *n* = 2 steroid-free due to comorbidity, *n* = 2 additional steroid intake due to cerebral metastases or antiemetic therapy during chemotherapy, protracted steroid reduction due to psychosomatic component *n* = 1). **H** Second-line immunosuppressants other than ECP shown with respect to treatment duration. Four patients had received second-line immunosuppressants prior to ECP therapy. **I** Representative endoscopic images of one patient with ICI-induced colitis before and after ECP therapy. Results were comparable for all ICI-colitis patients where endoscopy was performed. **J** ICI-colitis grades of all patients during ECP treatment.

The ORRs of all irAEs were 96.7% (29/30) (CI: 82.8–99.9%) and 96.3% (26/27) (CI: 81.0–99.9) at week 6 and 12, respectively (Fig. 2A). ORR based on the full analysis data set (FAS) at week 12 was 29/30 (96.7%; 95% CI: 82.8–99.9). We report the calculation with 27 patients as a sensitivity analysis because three patients had premature response assessments (d39, d44, and d62). CR rates were 56.7% (CI: 37.4–74.5%) and 70.4% (CI: 49.8–86.2%) at week 6 and 12, respectively. At week 6, organ-specific response rates were 100% (CI: 76.8–100.0%), 88.2% (CI: 63.6–98.5%), and 100% (CI: 29.2–100.0%) for ICI-colitis, ICI-hepatitis, and ICI-dermatitis, respectively (Fig. 2B). At week 12, organ-specific response rates were 100% (CI: 75.3–100.0%), 86.7% (CI: 59.5–98.3%), and 100% (CI: 29.2–100.0%) for ICI-colitis, ICI-hepatitis, and ICI-dermatitis, respectively (Fig. 2C). To determine how fast the response occurred we also determined response rates at week 1 after start of ECP as a post-hoc analysis. We observed an overall response rate (ORR) of 63.3% at 1 week after treatment start (only 2 ECP sessions) (Supplementary Fig. 1B). One patient exhibited an increase in colitis irAE grade after one ECP session (Supplementary Fig. 1B). However, this patient achieved a complete remission (CR) after 4 ECP sessions, which lasted until EOS. Median duration of GC pre-treatment was 4 weeks (range 1–24 weeks) (Fig. 2D), and four patients (13.3%) received a second-line immunosuppressant prior ECP, without improvement (Supplementary Table 1). The median time to response was 7 days (range 5–42 days) (Fig. 2E and Supplementary Fig. 1C). Response to ECP was long- lasting as only one patient had a relapse of ICI-colitis at month 6, requiring ECP re-exposure. This patient responded without the need of GC dose >0.25 mg/kg BW (methylprednisolone) or other immunosuppressive agents. CR of ICI-colitis was reached already after the second ECP in this patient.

During follow-up, six patients were re-exposed to ICI therapy without relapse of irAE. GC could be reduced in all patients (Fig. 2F). Individual courses were available in 22 patients (Fig. 2G) as a post-hoc analysis.

The steroid-free response rate (post-hoc analysis, excluding patients that did not reach the time point) at week 6 was 46.2% (12/26 patients). The 14 responder patients (53.8%) that were still on steroids at week 6 had a median steroid dose of 8.75 mg/d prednisolone equivalent. 23/26 patients (88.5%) were steroid-free responders at week 12. The three patients (11.5%) that were still on steroids at week 12 had a median steroid dose of only 5 mg/d prednisolone equivalent. 26/29 (89.7%) of responders could be analyzed for steroid-free response rate at week 6 and 12. Three patients could not be analyzed due to premature EOS (*n* = 2) and premature EOT (*n* = 1).

We performed a post-hoc analysis for the steroid-free remission rate, including also patients who prematurely exited the study. The steroid-free response rate, including all patients, was 40% (12/30 patients) at week 6 and 76.6% (23/30 patients) at week 12.

A pre-specified secondary endpoint was corticosteroid treatment at week 0 compared to week 12. For this endpoint, the data of 25/30 (83.3%) patients are available, as shown in Fig. 2F. For five patients, these data are not available because: *n* = 2 did not reach week 12 and had premature end of study (EOS) due to death because of tumor progression; *n* = 1 premature end of treatment because of complete remission of irAEs; two patients were steroid-free in week 0 of ECP start after having a steroid treatment before ECP started. As a post-hoc analysis, week −1 versus week 12 is shown in Supplementary Fig. 1D.

In 84.0% of patients (21/25) GC were completely discontinued at EOT. We observed that the median time to complete discontinuation of any other immunosuppressive therapy was 1.9548 months (lower 95% confidence limit 1.577; upper 95% confidence limit 3.6797). 2/30 patients (6.6%) received a second-line immunosuppressant (MMF) before/during ECP. In both patients, MMF could be tapered, and in one patient discontinued. Two additional patients (2/30) had received a second-line immunosuppressant prior to ECP (MMF, infliximab) without clinical improvement and then received ECP and responded (Fig. 2H).

Endoscopic examination in patients with ICI-colitis revealed a decrease in inflammation upon ECP treatment, characterized by a decrease in erythema and erosions as shown for one representative patient (Fig. 2I). The colitis severity according to CTC-AE over time in all patients is shown in Fig. 2J. The colitis severity for individual patients is shown in Supplementary Fig. 1E–R. Duration of response (DOR) analysis including 29/30 patients showed a median DOR of 14.4 months, calculated with the Kaplan–Meier method.

This phase Ib/II study has safety as the pre-defined primary endpoint, and efficacy inferences are ultimately exploratory.

### ICI colitis response is associated with reduced inflammatory gene expression in the colon

**Fig. 3:**
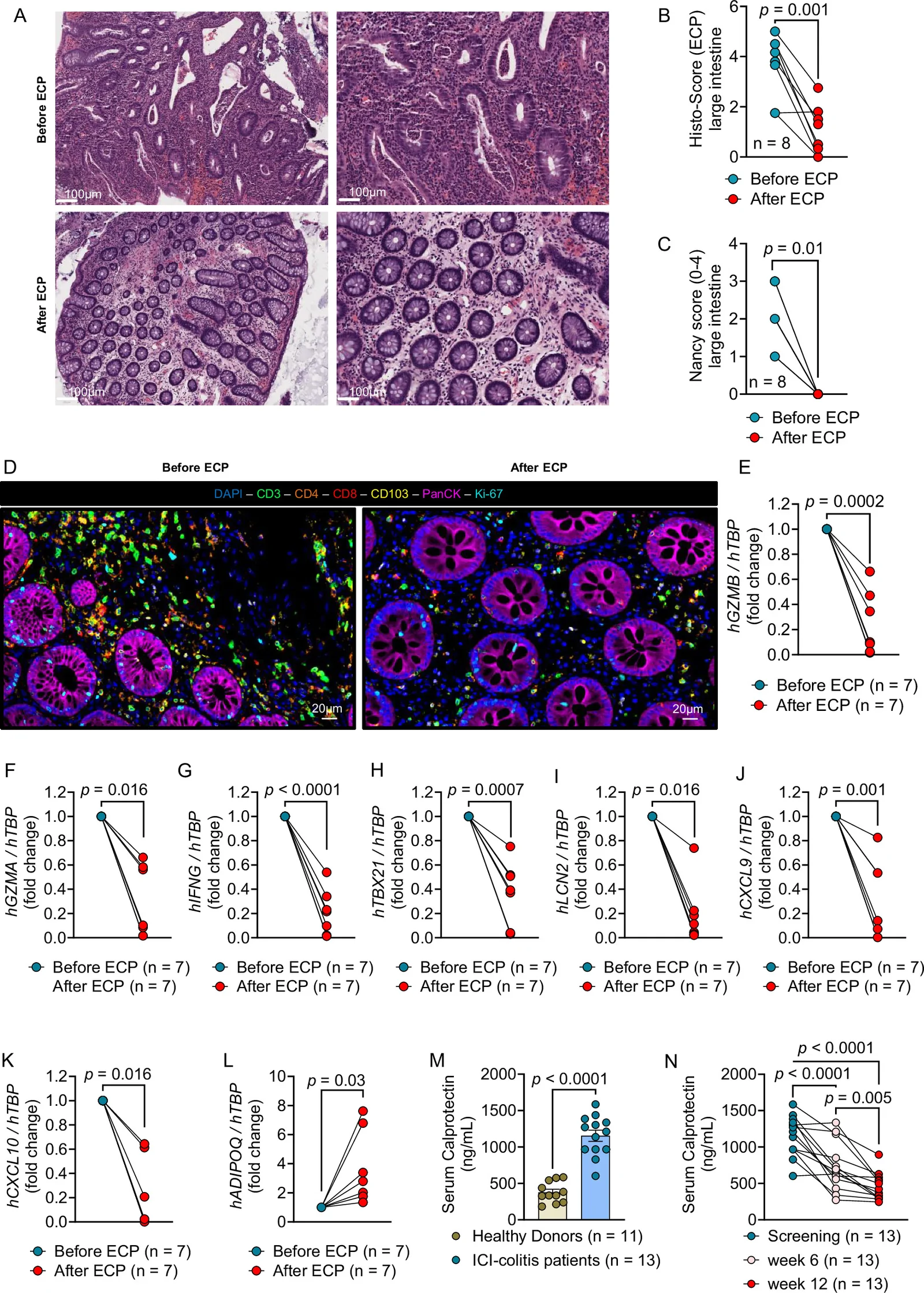
Changes of inflammation-associated gene expression in ICI-colitis patients before and after ECP therapy. **A** Microscopic images of H&E-stained colon biopsies of one patient with ICI-induced colitis before and after ECP therapy. Scale bar: 100 µm. Results were comparable for all patients where biopsies were available. **B** Histopathological severity (total score 0–9) of ICI-colitis before and after ECP treatment (week 12). Each line connecting the blue and red dots represents an individual patient (*n* = 8). The *P*-value was calculated using a two-tailed Student’s paired t-test. **C** Inflammatory intensity in the intestine of ICI-colitis patients before and after ECP treatment (week 12), using the Nancy scoring system. Each line connecting the blue and red dots represents an individual patient (*n* = 8). The *P*-value was calculated using a two-tailed Wilcoxon matched-pairs signed-rank test. **D** Multiplex immunofluorescence of colon tissue from an ICI-colitis patient before and after ECP therapy. Full staining for all indicated markers is shown. Scale bar: 20 µm. Gene expression of granzyme B (*GZMB*) (**E**), granzyme A (*GZMA*) (**F**), interferon-γ (*IFNG*) (**G**), T-bet (*TBX21*) (**H**), lipocalin 2 (*LCN2*) (**I**), *CXCL9* (**J**), and *CXCL10* (**K**) in colon tissue of ICI-colitis patients before and after ECP therapy. Gene expression was measured by qPCR, normalized to *TBP* expression, and shown as fold change relative to “before ECP.” Each dot represents an individual patient. *P*-values were calculated using a two-tailed Wilcoxon matched-pairs signed-rank test or a two-tailed Student’s paired t-test. **L** *ADIPOQ* gene expression in colon tissue of ICI-colitis patients before and after ECP therapy. Gene expression was measured by qPCR, normalized to *TBP* expression and shown as fold change relative to “before ECP.” Each dot represents an individual patient. The *p*-value was calculated using a two-tailed Student’s paired t-test. **M** Calprotectin concentrations in the serum of healthy donors or ICI-colitis patients before ECP therapy, measured by ELISA. The *p*-value was calculated using a two-tailed Student’s unpaired t-test. Data are presented as mean values ± SEM. **N** Calprotectin concentrations in the serum of ICI-colitis patients at screening (before ECP therapy) and at week 6 and week 12 of ECP therapy. The *p*-values were calculated using a repeated measures one-way ANOVA with Tukey’s multiple comparison test to correct for multiple comparisons.

Consistent with the macroscopic evaluation and clinical data, histopathological microscopic analysis of colon biopsies showed that ECP was associated with lower colitis severity compared to the analysis performed before the start of ECP in 7/8 analyzed patients (Fig. 3A, B). The assessment of ICI colitis by colonoscopy and histological analysis is reported in Supplementary Table 4. 4/6 patients (66.7%) with severe colitis (grade 3 and 4) were biopsied at baseline and after ECP. One patient with grade 4 colitis did not receive a colonoscopy because the risk for perforation was considered to be too high. One patient with colitis grade 2 and one patient with colitis grade 3 did not receive biopsy before ECP. The risk to injure the patient with the biopsy was considered higher than the benefit of histological confirmation of colitis.

Histological analysis performed prior to ECP yielded a histopathology score (Histo score) of 3 or higher in 75% (6/8) of ICI-colitis patients (Fig. 3B) and a Nancy Score^41^ of ≥2 in 75% (6/8) of ICI colitis patients (Fig. 3C). ICI-colitis severity decreased in 7/8 patients upon ECP therapy (Fig. 3B, C). When exploring the colonic immune-cell compartment of patients with ICI-colitis using microscopy, we found increased T cell infiltration in the colon mucosae of patients with ICI-colitis, which decreased after ECP therapy (Fig. 3D and Supplementary Fig. 2).

Cytotoxic T cells producing granzymes were shown to drive disease pathology in ICI-colitis patients^13,42^. Thus, to understand if the clinical and histological responses were connected to reduced intestinal inflammation, we analyzed granzyme A (*GZMA*) and granzyme B (*GZMB*) gene expression in colon biopsies before and after ECP. *GZMA* and *GZMB* gene expression decreased in colon biopsies after ECP (Fig. 3E, F). Since cytotoxic CD8^+^ T cells and Th1 cells were shown to contribute to the pathogenesis of ICI-colitis^42^, we analyzed the expression of *IFNG* and found it to be decreased after ECP (Fig. 3G). Comparably, the expression of *TBX21*, which encodes the Th1 transcription factor T-bet, was reduced in colon biopsies of ICI-colitis patients after ECP (Fig. 3H). Besides T cells, activated myeloid cells, including neutrophils were found to be drivers of ICI-colitis. Lipocalin 2 (*LCN2*) was shown to be an indicator of activated neutrophils in patients with ICI-induced colitis^13^ and was increased in patients with inflammatory bowel disease^43^. Confirming the protective activity of ECP, the colonic expression of *LCN2* was decreased after ECP therapy (Fig. 3I). In line with high inflammatory activity of colonic myeloid cells in ICI-colitis^42^ and the protective activity of ECP, the expression of *CXCL9* and *CXCL10* was significantly reduced in colon biopsies of ICI-colitis patients after ECP therapy (Fig. 3J, K). Both proteins play an important role for the recruitment of activated peripheral T cells into inflamed target organs. Adiponectin, which is encoded by *ADIPOQ*, was previously found to be produced by intestinal macrophages after ECP therapy^13^. ECP was associated with a significantly increased *ADIPOQ* expression in colon biopsies of ICI-colitis patients (Fig. 3L). Calprotectin in the stool was shown to correlate with ICI-colitis severity^44^. ICI-colitis patients had significantly higher serum levels of calprotectin compared to healthy donors (Fig. 3M), while the serum levels of colitis-related calprotectin were reduced during ECP therapy in line with the protective effect of ECP (Fig. 3N).

### ECP was associated with a reduction of pathogenic T cells in the blood of ICI-colitis patients

**Fig. 4:**
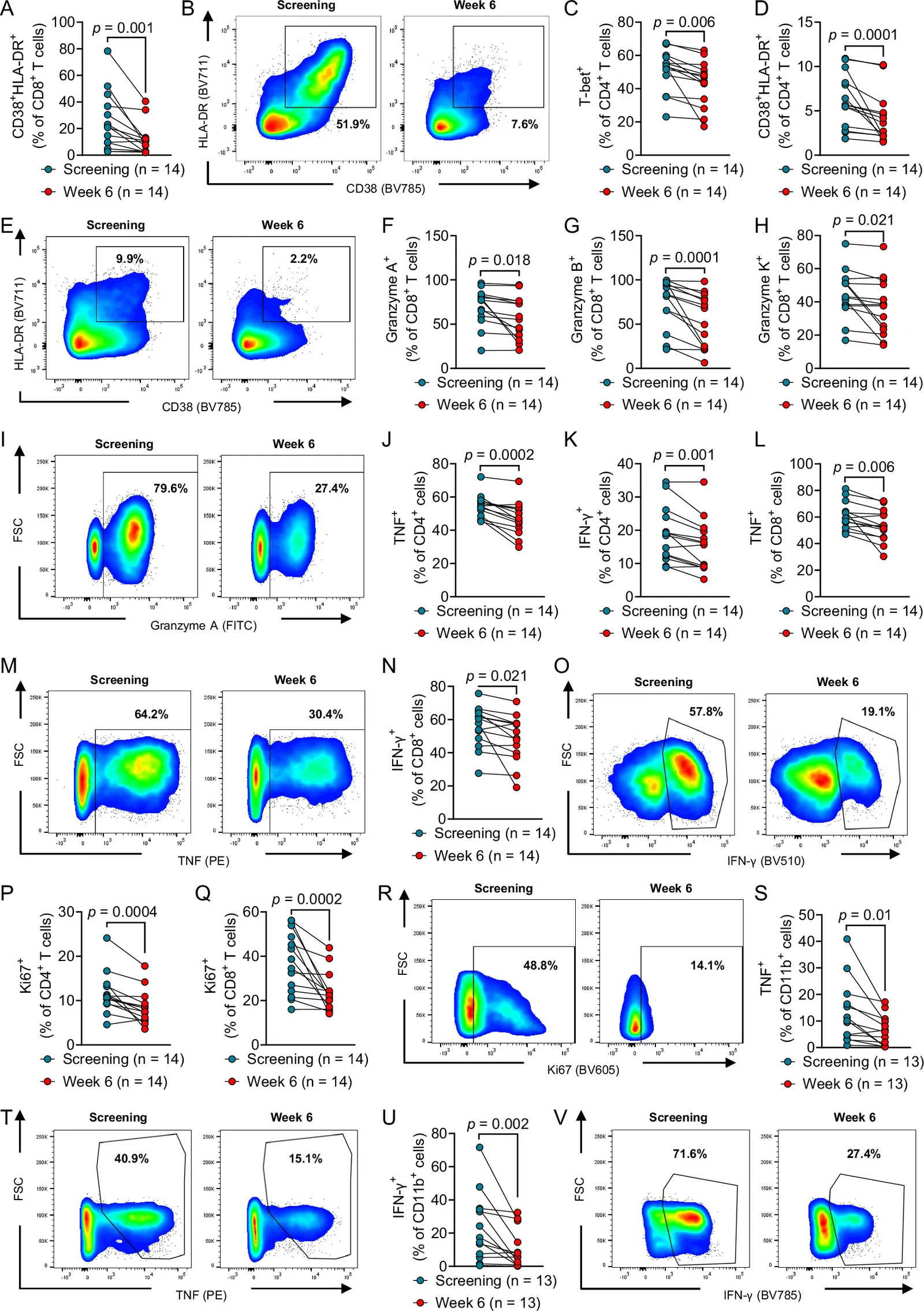
Immune cell phenotyping in peripheral blood samples of irAE patients before and after ECP therapy. **A**, **B** Frequency and representative FACS plots of CD38^+^HLA-DR^+^ cells as a percentage of CD8^+^ T cells in the peripheral blood of irAE patients at screening and week 6 of ECP therapy. **C** Frequency of T-bet^+^ cells as percent of all CD4^+^ T cells in the peripheral blood of irAE patients at screening and at week 6 of ECP therapy. **D**, **E** Frequency and representative FACS plots of CD38^+^HLA-DR^+^ cells as percentage of CD4^+^ T cells in the peripheral blood of irAE patients at screening and week 6 of ECP therapy. **F**–**H** Frequency of Granzyme A, Granzyme B, and Granzyme K expressing cells as percent of CD8^+^ T cells in the peripheral blood of irAE patients at screening and week 6 of ECP therapy. **I** Representative FACS plots of Granzyme A in CD8^+^ T cells in the peripheral blood of irAE patients at screening and week 6 of ECP therapy. **J**, **K** Frequency of TNF and IFN-γ-expressing cells as percent of CD4^+^ T cells in the peripheral blood of irAE patients at screening and week 6 of ECP therapy. **L**, **M** Frequency and representative flow cytometry plots of TNF-expressing cells as percent of CD8^+^ T cells in the peripheral blood of irAE patients at screening and week 6 of ECP therapy. **N**, **O** Frequency and representative flow cytometry plots of IFN-γ-expressing cells as percent of CD8^+^ T cells in the peripheral blood of irAE patients at screening and week 6 of ECP therapy. **P**–**R** Frequency of Ki67^+^ cells as percent of all CD4^+^ or CD8^+^ T cells in the peripheral blood of irAE patients at screening and at week 6 of ECP therapy. Representative FACS plots of Ki67 in CD8^+^ T cells. **S**–**V** Frequency and representative flow cytometry plots of TNF^+^ or IFN-γ^+^ cells as percent of CD11b^+^ cells in the peripheral blood of irAE patients at screening and week 6 of ECP therapy. The number of analyzed irAE patients is included in each graph. The *p*-values were calculated using a two-tailed Wilcoxon matched-pairs signed-rank test (**A**, **D**, **G**, **P**, **Q**, **U**) or a two-tailed Student’s paired t-test (**C**, **F**, **H**, **J**–**L**, **N**, **S**).

Aiming to further understand the global effect of ECP on different immune cells, we analyzed peripheral blood mononuclear cells (PBMC) of healthy donors and PBMCs of irAE patients (Supplementary Tables 1 and 5) before and after ECP by flow cytometry. Previous studies reported high frequencies of highly activated CD38^+^HLA-DR^+^CD8^+^ T cells and Th1 cells in intestinal biopsies and peripheral blood of ICI-colitis patients^13,42,45^. Consistent with these data, we found high frequencies of CD38^+^HLA-DR^+^CD8^+^ T cells and Th1 cells in the peripheral blood of irAE patients, irrespective of the affected target organ. We found that irAE-associated T cells were reduced during the course of ECP therapy (Fig. 4A–C and Supplementary Fig. 3A–H). Besides the effect on CD8^+^ T cells, the frequency of activated CD38^+^HLA-DR^+^ in CD4^+^ T cells was increased in irAE patients, whereas these cells decreased upon ECP therapy (Fig. 4D, E, and Supplementary Fig. 3I, J). In line with a highly activated phenotype of cytotoxic T cells, CD8^+^ T cells were found to produce high levels of Granzyme A, Granzyme B, and Granzyme K, which was reduced during ECP therapy (Fig. 4F–I and Supplementary Fig. 3K–R). Confirming increased frequencies of Th1 and cytotoxic T cells, the frequency of TNF and IFN-γ-producing cells was increased in both the CD4^+^ and CD8^+^ T cell compartments. ECP therapy was associated with declined the frequencies of TNF and IFN-γ-producing T cells (Fig. 4J–O and Supplementary Fig. 4A–J). Comparable to previous reports^13,42,46^, proliferating Ki67^+^ CD4^+^ and CD8^+^ T cells in the peripheral blood were characteristic for irAE patients, and decreased upon treatment with ECP (Fig. 4P–R and Supplementary Fig. 4K–U). Others had reported an enrichment of neutrophils and activated myeloid cells in the intestinal tract of ICI-colitis patients^42,47,48^. In line with these findings, we also found higher frequencies of activated proinflammatory myeloid cells in the peripheral blood of irAE patients, characterized by the production of pro-inflammatory cytokines. The frequency of TNF and IFN-γ-producing CD11b^+^ myeloid cells declined during ECP therapy (Fig. 4S–V and Supplementary Fig. 4V–Y).

Overall, we found a proinflammatory immune cell signature in intestinal tissue of ICI-colitis patients and in the peripheral blood of irAE patients, irrespective of the affected tissue. In agreement with the therapeutic effect of ECP, all inflammatory markers declined during ECP therapy.

### ECP was associated with reduced alanine aminotransferase levels in patients with ICI-hepatitis

**Fig. 5:**
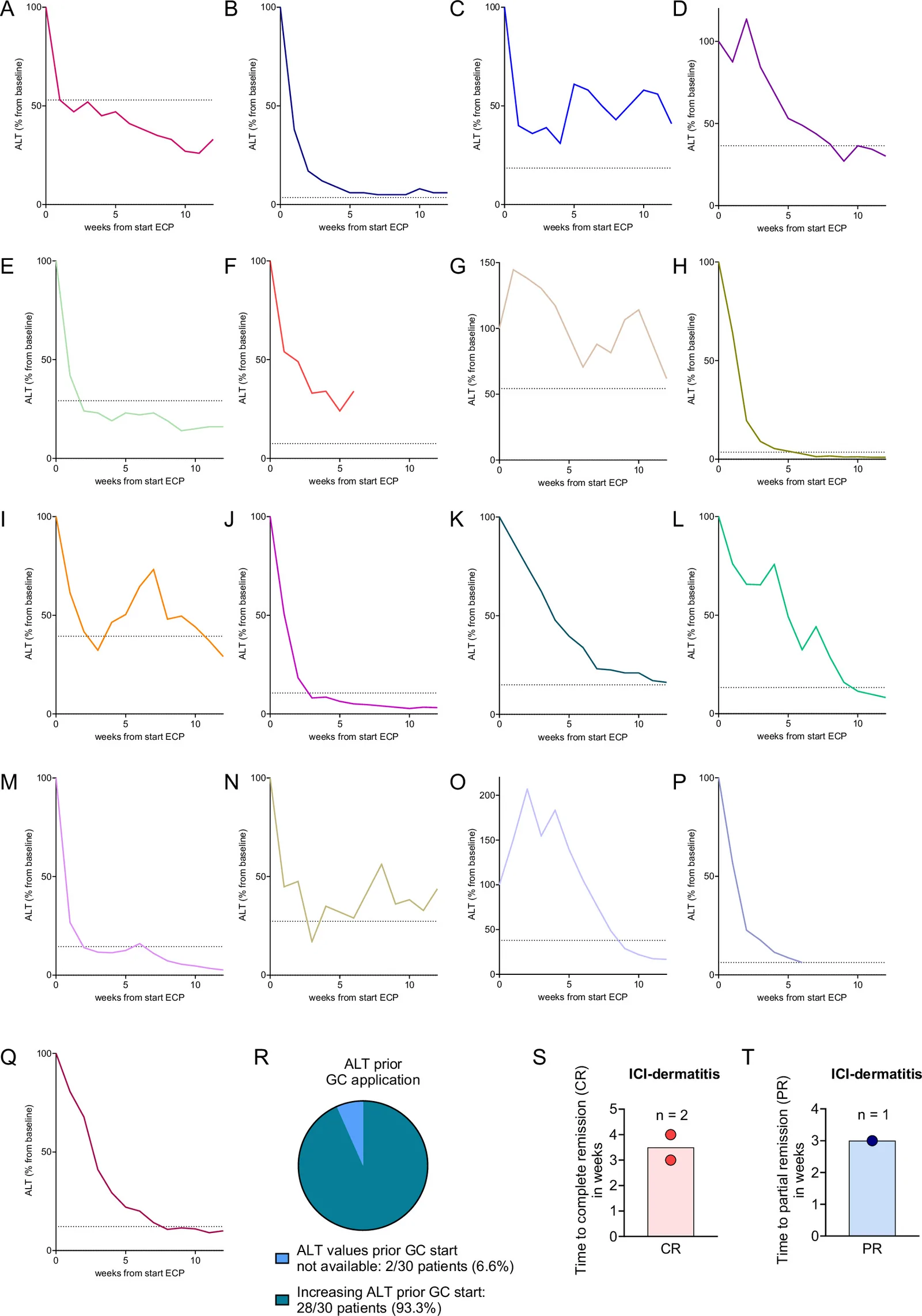
Response to ECP in patients with ICI-hepatitis or ICI-dermatitis. **A**–**Q** Time course of ALT levels of individual patients with ICI-hepatitis during ECP treatment. Relative blood ALT levels of patients included in the prospective clinical phase Ib/II trial are represented as a percent of baseline ALT level before start of ECP therapy as surrogate parameter for irAE hepatitis severity. Each graph represents an individual patient. ALT levels were assessed weekly until the end of treatment (EOT) in week 12. The dotted horizontal line represents the ALT level as a percentage from baseline. **R** Percentage of patients with stable vs increasing transaminases prior to initiation of steroids. **S**, **T** Time to complete remission and partial remission in ICI-dermatitis. The plot shows the time to complete remission (**S**) and partial remission (**T**) after ECP therapy in weeks (mean ± SEM). Each dot represents one patient.

Aiming to understand the effect of ECP on ICI-hepatitis individual patients, we measured alanine aminotransferase (ALT) as a surrogate for ICI-hepatitis severity in serum. ALT levels were determined at the screening timepoint and during the course of ECP therapy at each clinical visit. The ALT levels were plotted over time in percent relative to the baseline level, which was measured at the screening timepoint before initiation of ECP therapy. During ECP therapy, we observed that the relative ALT levels decreased in all ICI-hepatitis patients compared to baseline (Fig. 5A–Q). We analyzed ALT levels because ALT is more liver-specific than aspartate aminotransferase (AST) and because its measurement is recommended by the ASCO/ESMO guidelines^8,9^.

ALT reached the normal range in 13/17 patients (CR), while it declined but did not reach normal values in 4/17 patients (PR) (Fig. 5B, C, F, G). The dotted line in each graphic represents the percentage value of the ALT level at visit 1 relative to the reference value (Fig. 5A–Q). Median time to CR was 4 weeks (range 1–10) and median time to PR was 1 week (range 1–3) in patients with ICI-hepatitis. Absolute ALT, AST, and bilirubin values in all patients over time are shown in Supplementary Fig. 5A–C. There was no evidence for a decrease of ALT prior to ECP in the majority of the patients (Supplementary Fig. 5D). In a post-hoc analysis, we observed that the ALT values measured prior to the initiation of GC were increasing in 28/30 (93.3%) patients, while they were not available in 2/30 (6.6%) patients (Fig. 5R). Two patients with ICI-hepatitis underwent liver biopsy; for all others, hepatitis was defined biochemically, which is in agreement with the recommendations in the ESMO^8^ and ASCO guidelines^9^. Other guidelines, for example, GI society guidelines, look more favorably on liver biopsy^10^. We had ruled out other causes for increased ALT.

### ECP was associated with reduced severity of ICI-dermatitis

The severity of skin involvement was graded using the clinical dermatitis CTC-AE scoring system. We observed that in all ICI-dermatitis patients the CTC-AE score decreased during ECP therapy as compared to baseline (Supplementary Fig. 6A–C). 2/3 (66.7%) of ICI-dermatitis patients achieved complete remission. Time to complete remission (CR) ranged from 3 to 4 weeks (Fig. 5S). Time to partial remission is shown in Fig. 5T. Photographic pictures of one representative patient with ICI-dermatitis show a decrease in erythema and desquamation after ECP therapy (Supplementary Fig. 6D).

### Tumor staging, molecular remission, and survival

**Fig. 6:**
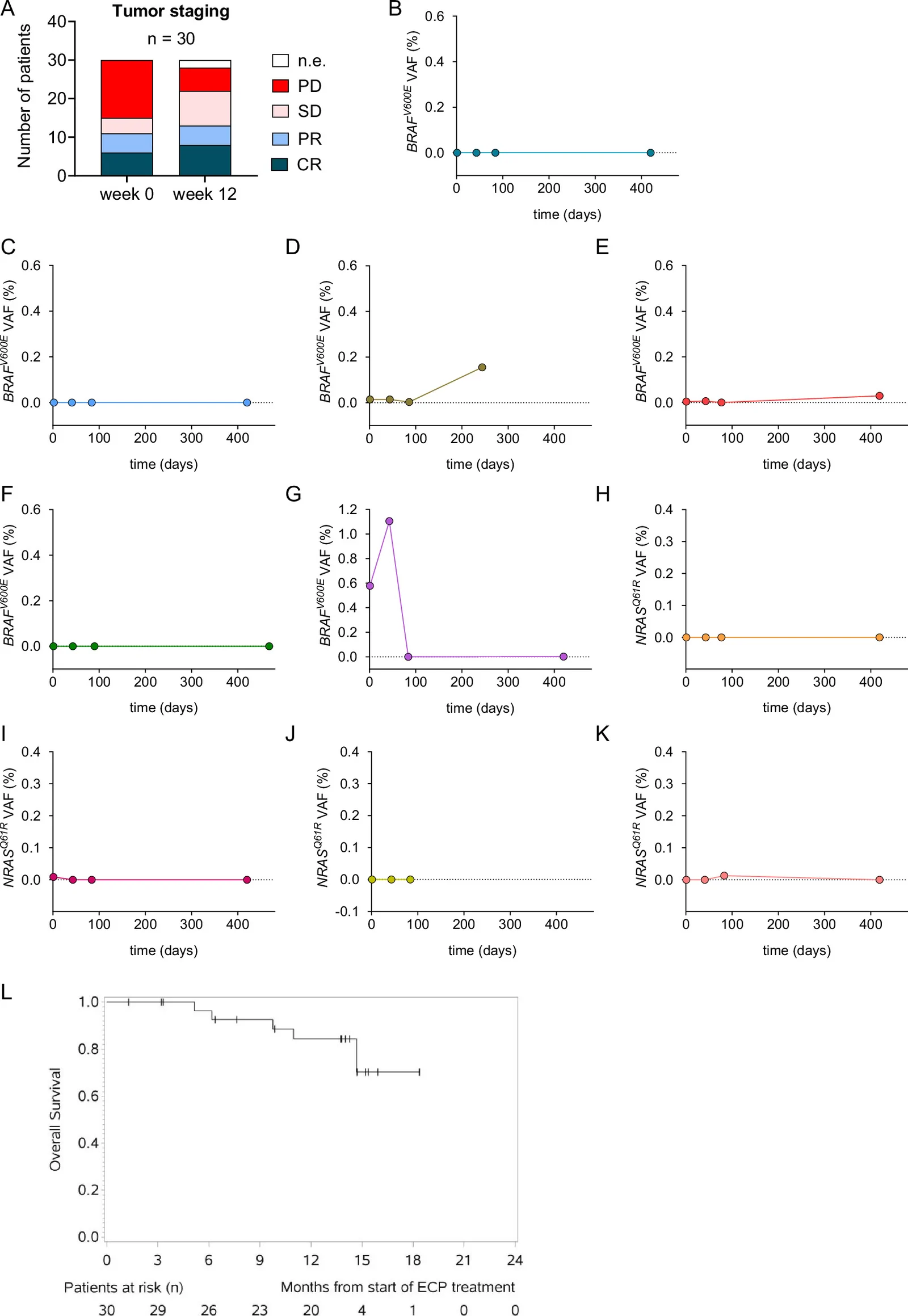
Tumor staging and circulating tumor DNA (ctDNA) analysis. **A** The graph shows the tumor response assessment of *n* = 30 irAE patients treated in the prospective phase Ib/II clinical trial before ECP (week 0) and after termination of ECP treatment (week 12). The tumor response was classified as complete response (CR), partial response (PR), stable disease (SD), and progressive disease (PD), according to RECIST criteria and based on radiological findings. Each tumor response category is represented by a specific color. Two patients were not eligible (n.e.) for tumor response assessment after ECP treatment (week 12) due to premature end of study (EOS). **B**–**K** The variable allele frequency (VAF) for *BRAF*^*V600E*^ and *NRAS*^*Q61R*^ was determined at multiple time points in patients during ECP in serum samples. **L** Overall survival from the first ECP intervention until the last follow-up visit or death from any cause was estimated with the Kaplan–Meier method.

All patients underwent tumor staging at study inclusion in week 0 and at EOT in week 12. While the majority of the patients suffered from advanced metastatic disease (progressive disease, PD) at trial inclusion tumor staging at week 12 showed no negative impact of ECP on primary disease control, with no increase of PD after ECP therapy (Fig. 6A). This was further supported by sensitive ctDNA monitoring during ECP in a subset of ten patients with melanoma and known *BRAF*^V600E^ or *NRAS*^Q61R^ mutations (Fig. 6B–K). The ctDNA monitoring was performed as an additional non-diagnostic readout, and ctDNA levels remained undetectable for nearly all patients throughout the observation period. Forty percent (4/10) of the patients shown in ctDNA testing received targeted therapy during the ECP treatment period. Two of these patients showed a decrease in tumor size in corresponding imaging methods, while one patient showed a stable tumor control according to RECIST 1.1 (SD in corresponding imaging before and after ECP). One patient (Fig. 6D) showed increasing ctDNA levels during ECP (10%, 1/10), which correlated with a progressive disease in imaging according to RECIST 1.1 before and after ECP treatment. The burden of disease remained undetectable throughout the study for most patients (90%, 9/10). Twelve-month OS was 84.3% (CI: 63.4–93.8%) (Fig. 6L). A major toxicity of GC is a decrease of lymphocyte counts during treatment^49^. We found that frequencies of T cells, B cells, and NK cells in PB were not reduced after ECP therapy compared to the immune status before ECP therapy (Supplementary Fig. 7A–H).

## Discussion

In this single-arm trial, we observed safety of ECP without an obvious negative impact on antitumor immunity, though these findings are limited by a small sample size with heterogenous tumors. Severe irAEs of ≥grade 3 involving the intestines, liver, and skin can negatively impact the outcome of patients treated with ICIs. Current therapeutic strategies for irAEs include GCs and long-term interruption of immunotherapy^8,9^. However, it was shown that GC therapy reduced anti-tumor immunity in cancer patients^14,17,25,50–53^ and abrogated ICI-induced antitumor immunity in preclinical mouse models^12,13^. Therefore, immunosuppressive therapies that help to avoid GCs while allowing for ICI-mediated antitumor immunity are an unmet medical need. Based on our previous studies in which we successfully treated patients with steroid-refractory (SR)-irAEs with ECP^13,31^, the ECIR trial received approval for a follow-up study with an expanded patient cohort of *n* = 30 patients.

The primary endpoint of the trial was safety. We observed low ECP-related toxicity without severe ECP-related adverse events (AEs). Only 6/105 (5.7%) AEs were ECP-related, and all were CTC-AE grade 1. AEs were mild in most cases (79.0% with ≤ CTC-AE grade 2) and self-limiting. Tumor staging revealed less patients in PD at week 12 compared to week 0 at the start of ECP therapy. Monitoring of ctDNA in the serum of melanoma patients harboring *BRAF*^V600E^ or *NRAS*^Q61R^ mutations revealed that most patients had undetectable ctDNA levels at all points throughout the analysis.

We found evidence of efficacy in most patients treated with ECP. The overall response rate for all irAEs was 96.3%. Colitis-, hepatitis-, and dermatitis-specific response rates were 100%, 86.7%, and 100%, respectively, and GCs could be reduced in all patients after ECP therapy. The treatment with ECP was associated with reduced expression of pro-inflammatory genes in the intestinal tract of ICI-colitis patients, including genes encoding for granzyme A, interferon-γ, and T-bet. In line, highly activated T cells were reduced in the peripheral blood of all irAE patients during ECP therapy, irrespective of the affected tissue. Similar to our previous analyses^13^, ECP therapy was associated with *ADIPOQ* expression in the intestinal tract of ICI-colitis patients, while reducing myeloid cell-associated activation markers.

The median duration of 4 weeks of corticosteroid treatment of the study patients prior to ECP start is within the expected range reported by other groups. The reported median duration of steroid treatment for ICI-colitis is 64 days^54^, 59 days^55^, or 6 weeks^56^. In some patients, the corticosteroids given as first-line therapy could have contributed to the clinical response under ECP.

In contrast to the long steroid treatment in the patients included in this trial, contemporary treatment guidance at most high-volume centers is to start secondary immune suppression at day 3 for non-responders to steroids and within a week for partial responders. Only a randomized trial can answer if there is a biological reason for some patients being treated longer with steroids and if that has impacted the response to ECP versus how this would impact the response to other second-line treatments.

Novel therapeutic modalities besides GCs for the treatment of irAEs are an unmet medical need. While one prospective trial on IL-6 blockade for ICI-colitis reported the best response at any timepoint during a period of 8 weeks after the start of treatment, it did not report any patient with a complete response^57^. Response to both infliximab and vedolizumab is typically high, as reported by the group of M. Wang^55,58^. A retrospective analysis on ICI-colitis reported 89% and 88% response rates to the α4β7-integrin inhibitor vedolizumab and the anti-TNF antibody infliximab, respectively^26^.

Endoscopic remission rates for ICI-colitis patients refractory to GC and/or infliximab were 54% after vedolizumab-treatment^27^, and retrospective analyses applying infliximab in ICI-colitis showed CR rates of 31 and 29%^28,29^. The organ-specific CR rates in patients treated with ECP were 11/13 (84.6%), 9/13 (69.2%), and 2/3 (66.6%) for ICI-colitis, ICI-hepatitis, and ICI-dermatitis, respectively at week 12.

ECP-treated patients did not experience any severe infection, which is consistent with our observation that T cell, B cell, and NK cell frequencies in the PB of the patients remained stable despite ECP treatment. In contrast, severe infections have been reported for inflammatory bowel disease patients receiving infliximab or vedolizumab^59,60^. ECP relies on the generation of maturationally synchronized human dendritic cells (DC)^61^ and therefore differs from other immunotherapies. DCs assist the immune response as key professional antigen-presenting cells, processing and presenting antigens to T cells. The capacity of ECP to promote this differentiation and to generate antigen-specific immunity supports the concept that ECP contributes to anti-tumor immunity^62,63^.

In addition to clinical and histological response assessment, we also monitored previously described immune cell phenotypes in the peripheral blood of ICI-colitis patients^42,45^ and observed that ECP therapy was associated with reduced disease-associated activated CD4^+^ and CD8^+^ T cells.

The limitations of our trial include the small sample size as well as the study design of a non-blinded single-arm study. This phase I/II study has safety as the primary endpoint, and efficacy inferences are ultimately exploratory. A randomized trial comparing ECP to best available therapy is needed to determine the efficacy of ECP compared to other available therapies.

In summary, our study extends prior clinical results on ECP as an immunomodulatory approach to control ICI-colitis, ICI-hepatitis, and ICI-dermatitis, showing safety and providing longitudinal intestinal gene expression analysis under a therapeutic intervention. To determine the efficacy of ECP in comparison to other second-line irAE therapies, ECP needs to be compared to the best available therapy in a prospective randomized trial.

## Methods

### Study design

The ECIR trial is an investigator-initiated, open-label, non-randomized, multi-centered single-arm phase Ib/II clinical trial (registered at European Union Drug Regulating Authorities Clinical Trials Database; EudraCT-No.: 2021-002073-26) conducted by three medical centers including the University Medical Center Freiburg, University Medical Center Essen and University Medical Center Regensburg. It was approved by the Federal Institute for Drugs and Medical Devices (BfArM) and the corresponding Ethic Committees in Germany 2022 (ethic committee approval numbers: 22-1052-AMG-ff multi; 23-1485-S1; Albert-Ludwigs-Universität Freiburg, Germany). It was conducted in line with the guidelines for GCP of the International Council for Harmonization, the principles of the Declaration of Helsinki, and local regulations. All patients and healthy donors signed an informed consent form prior to inclusion into the study. The trial was designed for an intervention period of 12 weeks, and a follow-up period 12 months after end of ECP treatment.

The trial was performed in two recruitment phases. The initial study population was set to 14 included patients. Based on promising preliminary results, the trial was granted an additional recruitment of 16 patients, which resulted in an overall recruitment of 30 patients. The trial was initiated on July 15th 2022; the first patient was included on August 1st 2022, and the last patient on September 2nd 2024. The trial ended when the last patient reached EOS (10/2025).

### Patient population

Patients ≥18 years were eligible if they were diagnosed with either ICI-colitis, ICI-hepatitis, ICI-dermatitis, or ICI-pneumonitis (or a combination of these) following anti-PD-1, anti-PD-L1, anti-CTLA-4 antibody, or any combination of these as treatment for any malignancy in the last 24 months before screening. Exclusion of other possible causes for the symptoms were necessary. Further, these patients needed to be refractory to GC-treatment, defined as no improvement with regard to CTC-AE grade after 72 h treatment with at least 1 mg/kg BW/day prednisolone equivalent, with up to one additional second-line therapy after GC-treatment. The entire set of inclusion and exclusion criteria is provided in the study protocol (Supplementary Information or on https://www.clinicaltrialsregister.eu/ under EudraCT-No. 2021-002073-26).

All patients were discussed in the Board for immune related adverse events at Freiburg University Medical Center or Essen Medical Center.

### Extracorporeal photopheresis

ECP was performed on 2 consecutive days per week for the first 4 weeks and then every other week until the end of treatment (EOT) in week 12. The ECP procedure was performed using the THERAKOS CELLEX Photopheresis System. For the procedure, 1500 ml of patient blood was withdrawn, processed via leukapheresis, exposed to Methoxsalen (Uvadex) and UVA irradiation. The processed blood was reinfused intravenously into the patient’s body. Additional information is provided in the study protocol (Supplementary Information).

### Response assessment

IrAE grade was determined at screening and every clinical visit according to the Common Terminology Criteria for Adverse Events (CTC-AE v5.0)^64^. Response was assessed by comparing the irAE grade at week 6 and 12 after initiation of ECP therapy with the initial irAE grade at visit one directly prior the first ECP treatment.

A complete remission (CR) was defined as a complete resolution of irAE-related symptoms, while partial remission (PR) was defined as a reduction of at least one irAE grade of at least one organ manifestation. In case of more than one organ involvement, the organ manifestation with the worst irAE grade was used for assessment of the overall response rate (ORR). Response to treatment was defined as CR or PR according to CTC-AE v5.0 grading system. The organ-specific response rate reflects all CTC-AE of all irAEs.

Stable disease (SD) was defined as no change in irAE grade, while progressive disease (PD) was defined as an increase of at least one CTC-AE grade. CR, PR, and PD were diagnosed only if the symptom severity remained stable for at least 5 consecutive days. When these terms (CR, PR, SD, and PD) were used for cancer outcomes this was explicitly stated.

### Safety assessment

Adverse events (AE) and serious adverse events (SAE) were evaluated, documented, and monitored at every clinical visit based on physical and laboratory examination. The severity of AEs and SAEs was determined according to the Common Terminology Criteria for Adverse Events (CTC-AE v5) scoring system^64^ and relevant measures where established if necessary (e.g., substrate substitution in case of deficiency). SAEs were reported to the responsible Pharmacovigilance Clinical Trial Unit according to protocol within 24 h after occurrence. Each AE and SAE was assessed for causal relationship to ECP by the investigator.

The adverse events were reported by the treating physicians of the patients, and their relation to treatment was also judged by these physicians, which is according to GCP.

### Outcomes

The main goal of the trial was the assessment of the efficacy and safety of ECP for steroid-refractory ICI-colitis, ICI-hepatitis, ICI-dermatitis, or ICI-pneumonitis. Therefore, the primary endpoint was the rate of ECP-related AEs and SAEs.

The secondary endpoint of the study was to assess the efficacy of ECP based on the overall response rates at weeks 6 and 12. Other secondary endpoints included the assessment of time to treatment response (time to first documented PR or CR), duration of response, overall survival, time to complete discontinuation of GC and other immunosuppressive therapies, changes in the immune cell phenotype in peripheral blood and intestinal tract biopsies, relapse rate of irAEs after end of treatment with ECP and upon re-exposure to ICI therapy, as well as incidence of tumor progression or relapse. All secondary endpoints are provided in the study protocol (Supplementary Information).

### Statistical analysis

Statistical analysis plan: as this is a phase Ib/II trial without the intention of a confirmatory proof of efficacy, no formal sample size calculation is performed. The determination to include 30 evaluable patients is mainly based on feasibility considerations. It is expected that only few SAEs will occur. If the probability of any ECP-related SAE for a patient is 20% (*p* = 0.2), the probability to observe at least one patient with a treatment-related SAE is 0.956.

Clinical data were managed and analyzed using Prism 10 (GraphPad), and SAS 9.4. The full analysis data set (FAS) and the safety set (SAF) included all the patients who received at least one administration of ECP. Descriptive statistics were employed for reporting continuous variables and frequency and percentages of discrete variables. Exact two-sided 95% confidence intervals for response rates were calculated, based on the binomial distribution. Overall survival (OS) was defined as time from start of treatment with ECP until death, treating observation times where the event of interest did not occur as censored. Analyses of OS was performed with the Kaplan–Meier method. Time to response was assessed for responders only as days from initiation of ECP treatment until the first documentation of a reduction of at least one irAE grade of at least one organ manifestation. Duration of response (DOR) was calculated for responders only as time from first response to the first observation of irAE relapse/progression/death or the date of additional systemic treatment for irAE. DOR was analyzed with the Kaplan–Meier method.

Flow cytometry and gene expression data were tested for normality applying the Shapiro–Wilk test. An unpaired or paired two-tailed Student’s *t* test was applied to compare two groups. If the data did not meet the criteria of normality, the Mann–Whitney test or Wilcoxon signed-rank test was applied for unpaired or paired analyses, respectively. In case multiple groups were compared, the Friedman test (paired data) with Dunn’s multiple testing was chosen if the data did not meet the criteria of normality. Statistical differences between multiple groups of normally distributed data were analyzed using a repeated measures (paired data) one-way ANOVA with Tukey’s multiple comparison test. The data were corrected for multiple testing.

### Endoscopy

In patients with ICI-colitis, endoscopic examination was performed by a specialized gastroenterologist of the Department of Internal Medicine II—Gastroenterology, Hepatology, Endocrinology, University Medical Center Freiburg prior to ECP initiation and at response assessment in week 6 or later. Intestinal biopsy specimens from small and large intestine were embedded in paraffin for histopathological analyses. Gene expression was measured by qPCR on mRNA isolated from the paraffin-embedded intestinal tissue biopsies.

### Histopathology scoring of irAE in patient colon tissue

Intestinal biopsy specimens (small intestine, large intestine) were prepared according to standardized procedures for formalin-fixed and paraffin-embedded (FFPE) tissues at the Institute of Surgical Pathology, University Medical Center Freiburg. In brief, 2 µm sections were generated, and H&E stains were performed. Scoring of respective specimens relied on the following parameters: eosinophilia, apoptosis, ulcer, cryptitis, or crypt abscess formation, lympho-plasmocytic infiltration, and signs of regeneration, focal or diffuse disease presentation; each parameter was evaluated for absence (0) or presence (1). A sum score (0–9) for each individual sample was calculated in a blinded fashion by an experienced pathologist. The histopathological scoring according to the Nancy score was performed as previously described^41^.

### Multiplex immunofluorescence of patient colon tissue

The OPAL (Opal 6-Plex Manual Detection Kit, Cat. No.: NEL811001KT; Akoya Bioscience) method was employed for multiplex immunofluorescence of 2 µm sections. Antigen retrieval and OPAL staining were performed as per manufacturer’s protocol. Slides were stained with antibodies against CD3 (Dako, Cat. No.: IR503; RRID:AB_3094578), CD4 (Abcam, Cat. No.: ab133616; RRID:AB_2750883), CD8 (Dako, Cat. No.: M7103; RRID:AB_2075537), CD103 (Atlas Antibodies, Cat. No.: HPA036313; RRID:AB_10795355), PanCK (Dako, Cat. No.: IR053; RRID:AB_2868599) and Ki-67 (Dako, Cat. No.: IR626; RRID:AB_2890068) as described previously^13^. Image acquisition was performed using the PhenoCycler Fusion microscope (Akoya Biosciences). The Phenochart v.2.0.0 (Akoya Biosciences) and InForm v.3.0 (Akoya Biosciences) software were used for spectral unmixing and background subtraction.

### Flow cytometry

For analysis of lymphocyte subpopulations in patient peripheral blood, 50 µl EDTA blood was incubated with 5 µl of the respective antibody (anti-CD3 PE-Cy7, anti-CD4 FITC, anti-CD8 APC, anti-CD16 PE, anti-CD19 FITC, anti-CD6 PE) for 30 min. Characteristics of healthy donors are shown in Supplementary Table 5. Two point five milliliters Cytofix/Cytoperm (BD Biosciences, Cat. No.: 554714) was added, and the samples were incubated for additional 10 min. The samples were centrifuged for 5 min (2000 rpm), and the supernatant was removed before resuspension in FACS Flow (BD Biosciences, Cat. No.: 342003). Starting with the 12th patient, analysis of lymphocyte subpopulations was done according to manufacturer´s protocol (BD Biosciences) using the BD Multitest 6-color TBNK (anti-CD3 FITC, anti-CD16 PE, anti-CD56 PE, anti-CD45 PerCP-Cy5.5, anti-CD4 PE-Cy7, anti-CD19 APC, anti-CD8 APC-Cy7; BD Biosciences, Cat. No.: 662967) together with BD Trucount tubes (BD Biosciences, Cat. No.: 663028). All data were measured with a BD FACS Canto II flow cytometer (BD Biosciences) and analyzed using the FACSDiva Software V6 (BD Biosciences). Antibodies used are summarized in Supplementary Table 6 containing all information on clones, vendors, catalog numbers, and unique identifiers. Blood samples were collected before and after the first ECP procedure, and at two variable timepoints during ECP therapy.

For immune cell characterization in irAE patients and healthy donor samples, PBMCs were isolated by density-gradient separation (Lymphocyte separation medium; Anprotec, Cat. No.: AC-AF-0018) as described previously at screening before ECP therapy, and weeks 6 and 12 of ECP therapy^13^. Isolated PBMCs were frozen in 90% Fetal Constance (Anprotec, Cat. No.: AC-SM-0190) with 10% DMSO (Roth, Cat. No.: A994.2), and all samples were thawed and analyzed at the same time. Viability was analyzed by Zombie NIR fixable dye (BioLegend, Cat. No.: 423106; 1:500 in PBS) staining, and Fc receptors were blocked using FcR blocking reagent (Miltenyi, Cat. No.: 130-059-901) for 10 min at RT. Surface markers were stained with fluorochrome conjugated antibodies for 30 min at 4 °C. For the analysis of intracellular proteins, cells were fixed and permeabilized for 45 min at 4 °C using the Transcription Factor Staining Buffer Set (eBioscience, Cat. No.: 00-5523-00) or the Fixation/Permeabilization Kit (BD Biosciences, Cat. No.: 554714) for staining of intranuclear or cytoplasmatic proteins and cytokines, respectively. For staining of TNF and IFN-γ, cells were stimulated in the presence of Cell Stimulation Cocktail with protein transport inhibitor (1:500; eBioscience, Cat. No.: 00-4975-93) for 4 h at 37 °C before surface and intracellular cytokine staining. For the analysis of Granzymes, cells were incubated in the presence of GolgiPlug (1:1000; BD Biosciences, Cat. No.: 555029) and GolgiStop (1:1500; BD Biosciences, Cat. No.: 554724) for 4 h at 37 °C, before surface and intracellular staining. Stimulation and incubations with GolgiPlug/GolgiStop were done in RPMI 1640 medium (ThermoFisher Scientific, Cat. No.: 21875-034) supplemented with 10% FCS (Fetal Constance, Anprotec Cat. No.: AC-SM-0190) and 1% P/S (ThermoFisher Scientific, Cat. No.: 15140-122). The samples were acquired using a BD LSR Fortessa equipped with four lasers, and analyzed using the FlowJo V10 software. Gating strategies are shown in Supplementary Figs. 8–11; antibodies are listed in Supplementary Table 6, containing all information on clones, vendors, catalog numbers, and unique identifiers. The data is presented as mean ± SEM or individual paired comparisons between patients.

The following panels were used for PBMC analysis:

Panel 1 (activated T cells and Th1): anti-CD45 FITC, anti-CD3 BV650, anti-CD8 PB, anti-CD4 BV510, anti-CD103 PE, anti-CD69 PE-Cy7, anti-CD38 BV785, anti-HLA-DR BV711, anti-KI67 BV605, anti-T-bet APC; Panel 2 (IFN-γ and TNF in T cells): anti-CD3 BV650, anti-CD8 FITC, anti-CD4 PerCP-Cy5.5, anti-CD38 BV785, anti-HLA-DR BV711, anti-TNF PE, anti-IFN-γ BV510, anti-Perforin PB, anti-Granulysin PE-Cy7, anti-KI67 BV605, anti-T-bet APC; Panel 3 (granzymes in T cells): anti-CD3 PE-Cy7, anti-CD8 APC, anti-CD4 BV510, anti-CD38 BV785, anti-HLA-DR PB, anti-Granzyme A FITC, anti-Granzyme B PE, anti-Granzyme K PerCP-Cy5.5, anti-KI67 BV605; Panel 4 (cytokines in myeloid cells): anti-CD45 FITC, anti-CD11b PB, anti-CD3 BV650, anti-CD4 BV510, anti-CD8 APC, anti-CD19 PE-Cy7, anti-TNF PE, anti-IFN-γ BV785, Rat IgG2bκ Isotype Ctrl Antibody (PB), Mouse IgG1κ Isotype Ctrl Antibody (PE), Mouse IgG1κ Isotype Ctrl Antibody (BV785).

### Calprotectin analysis in serum samples

For isolation of serum, whole blood of irAE patients was collected using S-Monovette Serum Gel CAT tubes (Sarstedt, Cat. No.: 06.1667.001). Serum samples of irAE patients were collected before initiation of ECP therapy (Screening) and at week 6 and 12 of therapy. Samples were centrifuged at 2000 × *g* for 10 min at RT to separate the serum and stored at −80 °C until further use. Serum samples of healthy controls were prepared comparably. Serum calprotectin levels were analyzed using the Human Calprotectin L1/S100-A8/A9 Complex ELISA Kit (Invitrogen, Cat. No.: EH62RB) according to manufacturer’s instructions. All samples were diluted 1:4000 and measured in duplicate. Data are displayed as mean ± SEM.

### RNA isolation, reverse transcription, and qPCR of FFPE samples

Total RNA from formalin-fixed paraffin-embedded (FFPE) patient specimens was isolated as described before^13^. In brief, the miRNeasy FFPE Kit (QIAGEN, Cat. No.: 217504) was used according to manufacturer’s instructions with modifications. Twice the suggested volume of Deparaffinization Solution (QIAGEN, Cat. No.: 19093) was used to remove paraffin. Proteinase K digestion was done for 60 min with constant shaking at 700 rpm. Up to 1 µg of total RNA was reverse transcribed into cDNA using the High-Capacity cDNA Reverse Transcription Kit (ThermoScientific Cat. No.: 4374967) in the presence of 15 ng Yeast RNA (ThermoScientific, Cat. No.: AM7120G)^65^. The quantitative PCR (qPCR) was done using the LightCycler 480 SYBR Green I Master Mix (Roche, Cat. No.: 04887352001) according to manufacturer’s instructions. In brief, a total of 10–40 ng cDNA and 5 µM forward and reverse primer were used in a 10 µl reaction volume. PCR was done on a LightCycler 480 machine (Roche). Primer sequences were designed to span exon-exon junctions, a length of 18–23 bp and with an anticipated product length of 100–200 bp. If multiple transcript variants of a gene were known, primers were designed to identify all known variants. The sequences were ordered from Eurofins Genomics (Germany). The expression of specific genes was normalized on reference gene expression and calculated relative to control using the 2^−ΔΔCT^ method. Oligonucleotides (qPCR primer sequences) used for gene expression analysis are listed in Supplementary Table 7.

### Monitoring of circulating tumor DNA by digital droplet PCR

In melanoma patients with known *BRAF*^V600E^ or *NRAS*^Q61R^ tumor mutations, digital droplet PCR (ddPCR) was performed to monitor circulating tumor DNA (ctDNA) in serum samples during ECP. We used two ddPCR assays that were developed and validated for sensitive noninvasive detection of ctDNA in clinical routine practice, as described before^66^. In brief, cell-free DNA was isolated from all serum samples (*n* = 39) using the QIAsymphony workflow (Qiagen) according to the manufacturer’s instructions. After isolation, cfDNA was processed and analyzed using a customized workflow on the Bio-Rad ddPCR technology, for which we optimized primer and probe design as well as PCR conditions and defined thresholds for positivity/negativity, as described before^66^. For each serum sample, mutant copies and wildtype copies were quantified, and the ctDNA concentrations were calculated as variant allele frequencies and expressed in percent (%)^66^.

### Reporting summary

Further information on research design is available in the Nature Portfolio Reporting Summary linked to this article.

## Supplementary information

Supplementary Information

Reporting Summary

Transparent Peer Review File

## Source data

Source Data

## Data Availability

All data are included in the Supplementary Information or available from the authors, as are unique reagents used in this Article. The raw numbers for charts and graphs are available in the Source Data file whenever possible. Source data are provided with this paper.

## References

[1] Vaddepally, R. K., Kharel, P., Pandey, R., Garje, R. & Chandra, A. B. Review of indications of FDA-approved immune checkpoint inhibitors per NCCN guidelines with the level of evidence. *Cancers* **12**, 738 (2020). 10.3390/cancers12030738 PMC7140028 32245016

[2] Vansteenkiste, J. et al. Current status of immune checkpoint inhibition in early-stage NSCLC. *Ann. Oncol.* **30**, 1244–1253 (2019). 10.1093/annonc/mdz175 31143921

[3] Gaynor, N., Crown, J. & Collins, D. M. Immune checkpoint inhibitors: key trials and an emerging role in breast cancer. *Semin. Cancer Biol.* **79**, 44–57 (2022). 10.1016/j.semcancer.2020.06.016 32623044

[4] Postow, M. A., Sidlow, R. & Hellmann, M. D. Immune-related adverse events associated with immune checkpoint blockade. *New Engl. J. Med.* **378**, 158–168 (2018). 10.1056/NEJMra1703481 29320654

[5] Johnson, D. B., Nebhan, C. A., Moslehi, J. J. & Balko, J. M. Immune-checkpoint inhibitors: long-term implications of toxicity. *Nat. Rev. Clin. Oncol.* **19**, 254–267 (2022). 10.1038/s41571-022-00600-w PMC8790946 35082367

[6] Choi, J. & Lee, S. Y. Clinical characteristics and treatment of immune-related adverse events of immune checkpoint inhibitors. *Immune Netw.* **20**, e9 (2020). 10.4110/in.2020.20.e9 PMC7049586 32158597

[7] Martins, F. et al. Adverse effects of immune-checkpoint inhibitors: epidemiology, management and surveillance. *Nat. Rev. Clin. Oncol.* **16**, 563–580 (2019). 10.1038/s41571-019-0218-0 31092901

[8] Haanen, J. et al. Management of toxicities from immunotherapy: ESMO Clinical Practice Guideline for diagnosis, treatment and follow-up. *Ann. Oncol.* **33**, 1217–1238 (2022). 10.1016/j.annonc.2022.10.001 36270461

[9] Schneider, B. J. et al. Management of immune-related adverse events in patients treated with immune checkpoint inhibitor therapy: ASCO guideline update. *J. Clin. Oncol.* **39**, 4073–4126 (2021). 10.1200/JCO.21.01440 34724392

[10] Li, M. et al. Utility of liver biopsy in diagnosis and management of high-grade immune checkpoint inhibitor hepatitis in patients with cancer. *JAMA Oncol.* **7**, 1711–1714 (2021). 10.1001/jamaoncol.2021.4342 PMC8461544 34554206

[11] Yang, H. et al. Stress-glucocorticoid-TSC22D3 axis compromises therapy-induced antitumor immunity. *Nat. Med.* **25**, 1428–1441 (2019). 10.1038/s41591-019-0566-4 31501614

[12] Hu, X. et al. Inhibition of IL-25/IL-17RA improves immune-related adverse events of checkpoint inhibitors and reveals antitumor activity. *J. Immunother. Cancer* **12**, e008482 (2024). 10.1136/jitc-2023-008482 PMC10961528 38519059

[13] Braun, L. M. et al. Adiponectin reduces immune checkpoint inhibitor-induced inflammation without blocking anti-tumor immunity. *Cancer Cell* **43**, 269–291 (2025). 10.1016/j.ccell.2025.01.004 39933899

[14] Arbour, K. C. et al. Impact of baseline steroids on efficacy of programmed cell death-1 and programmed death-ligand 1 blockade in patients with non-small-cell lung cancer. *J. Clin. Oncol.* **36**, 2872–2878 (2018). 10.1200/JCO.2018.79.0006 30125216

[15] Kostine, M. et al. Baseline co-medications may alter the anti-tumoural effect of checkpoint inhibitors as well as the risk of immune-related adverse events. *Eur. J. Cancer* **157**, 474–484 (2021). 10.1016/j.ejca.2021.08.036 34649118

[16] Bai, X. et al. Early use of high-dose glucocorticoid for the management of irAE is associated with poorer survival in patients with advanced melanoma treated with anti-PD-1 monotherapy. *Clin. Cancer Res.* **27**, 5993–6000 (2021). 10.1158/1078-0432.CCR-21-1283 PMC9401488 34376536

[17] Faje, A. T. et al. High-dose glucocorticoids for the treatment of ipilimumab-induced hypophysitis is associated with reduced survival in patients with melanoma. *Cancer* **124**, 3706–3714 (2018). 10.1002/cncr.31629 29975414

[18] Coutinho, A. E. & Chapman, K. E. The anti-inflammatory and immunosuppressive effects of glucocorticoids, recent developments and mechanistic insights. *Mol. Cell. Endocrinol.* **335**, 2–13 (2011). 10.1016/j.mce.2010.04.005 PMC3047790 20398732

[19] Oray, M., Abu Samra, K., Ebrahimiadib, N., Meese, H. & Foster, C. S. Long-term side effects of glucocorticoids. *Expert Opin. Drug Saf.* **15**, 457–465 (2016). 10.1517/14740338.2016.1140743 26789102

[20] Machado, A. P. et al. The safety of immunosuppressants used in the treatment of immune-related adverse events due to immune checkpoint inhibitors: a systematic review. *J. Cancer* **14**, 2956–2963 (2023). 10.7150/jca.87335 PMC10583582 37859810

[21] Hoisnard, L. et al. Adverse events associated with JAK inhibitors in 126,815 reports from the WHO pharmacovigilance database. *Sci. Rep.* **12**, 7140 (2022). 10.1038/s41598-022-10777-w PMC9065106 35504889

[22] Kamanamool, N. et al. Efficacy and adverse events of mycophenolate mofetil versus cyclophosphamide for induction therapy of lupus nephritis: systematic review and meta-analysis. *Medicine* **89**, 227–235 (2010). 10.1097/MD.0b013e3181e93d00 20616662

[23] Busuttil, R. W. & Klintmalm, G. B. *Transplantation of the Liver* (W.B. Saunders, 2015).

[24] Verheijden, R. J. et al. Association of anti-TNF with decreased survival in steroid refractory ipilimumab and anti-PD1-treated patients in the Dutch Melanoma Treatment Registry. *Clin. Cancer Res.* **26**, 2268–2274 (2020). 10.1158/1078-0432.CCR-19-3322 31988197

[25] van Not, O. J. et al. Association of immune-related adverse event management with survival in patients with advanced melanoma. *JAMA Oncol.* **8**, 1794–1801 (2022). 10.1001/jamaoncol.2022.5041 PMC9614679 36301521

[26] Zou, F. et al. Efficacy and safety of vedolizumab and infliximab treatment for immune-mediated diarrhea and colitis in patients with cancer: a two-center observational study. *J. Immunother. Cancer* **9**, e003277 (2021). 10.1136/jitc-2021-003277 PMC8601082 34789551

[27] Abu-Sbeih, H. et al. Outcomes of vedolizumab therapy in patients with immune checkpoint inhibitor-induced colitis: a multi-center study. *J. Immunother. Cancer* **6**, 142 (2018). 10.1186/s40425-018-0461-4 PMC6280383 30518410

[28] Hillock, N. T., Heard, S., Kichenadasse, G., Hill, C. L. & Andrews, J. Infliximab for ipilimumab-induced colitis: a series of 13 patients. *Asia Pac. J. Clin. Oncol.* **13**, e284–e290 (2017). 10.1111/ajco.12651 27981760

[29] Kadokawa, Y. et al. Efficacy and safety of infliximab for steroid-resistant immune-related adverse events: a retrospective study. *Mol. Clin. Oncol.* **14**, 65 (2021). 10.3892/mco.2021.2227 PMC7890436 33680456

[30] Vieyra-Garcia, P. A. & Wolf, P. Extracorporeal photopheresis: a case of immunotherapy ahead of its time. *Transfus. Med. Hemotherapy* **47**, 226–235 (2020). 10.1159/000508479 PMC7315210 32595427

[31] Apostolova, P. et al. Extracorporeal photopheresis for colitis induced by checkpoint-inhibitor therapy. *New Engl. J. Med.* **382**, 294–296 (2020). 10.1056/NEJMc1912274 31940706

[32] Ruf, T. et al. Extracorporeal photopheresis as a treatment option for immune-related adverse events: two case reports and a prospective study. *J. Immunother.* **47**, 227–231 (2024). 10.1097/CJI.0000000000000510 38483178

[33] Gavriilaki, E. et al. Safety and efficacy of extracorporeal photopheresis for acute and chronic graft-versus-host-disease. *Blood* **142**, 7006–7006 (2023). 10.3390/ph17101279 PMC11510389 39458920

[34] Asensi Cantó, P., Sanz Caballer, J., Solves Alcaína, P., de la Rubia Comos, J. & Gómez Seguí, I. Extracorporeal photopheresis in graft-versus-host disease. Transplant. *Cell. Ther.* **29**, 556–566 (2023). 10.1016/j.jtct.2023.07.001 37419324

[35] Greinix, H. T., Ayuk, F. & Zeiser, R. Extracorporeal photopheresis in acute and chronic steroid‑refractory graft-versus-host disease: an evolving treatment landscape. *Leukemia* **36**, 2558–2566 (2022). 10.1038/s41375-022-01701-2 PMC9613461 36153436

[36] Dong, N. et al. A retrospective study of extracorporeal photopheresis (ECP) in treatment of cutaneous T-cell lymphoma (CTCL) evaluating global, skin and blood responses. *Blood* **142**, 3078–3078 (2023).

[37] Neustadter, J. H., Samarin, F., Carlson, K. R. & Girardi, M. Extracorporeal photochemotherapy for generalized deep morphea. *Arch. Dermatol.* **145**, 127–130 (2009). 10.1001/archdermatol.2008.547 PMC4075155 19221256

[38] Alfred, A. et al. The role of extracorporeal photopheresis in the management of cutaneous T-cell lymphoma, graft-versus-host disease and organ transplant rejection: a consensus statement update from the UK Photopheresis Society. *Br. J. Haematol.* **177**, 287–310 (2017). 10.1111/bjh.14537 PMC5412836 28220931

[39] Ertl, C. et al. Extracorporeal photopheresis vs. systemic immunosuppression for immune-related adverse events: interim analysis of a prospective two-arm study. *Eur. J. Cancer* **212**, 115049 (2024). 10.1016/j.ejca.2024.115049 39383612

[40] Thielmann, C. M. et al. Extracorporeal photopheresis as a therapeutic approach for treatment resistant immune-related adverse events in anti-PD-1-treated melanoma patients. *Front. Immunol.* **16**, 1727312 (2025). 10.3389/fimmu.2025.1727312 PMC12748251 41476990

[41] Marchal-Bressenot, A. et al. Development and validation of the Nancy histological index for UC. *Gut* **66**, 43–49 (2017). 10.1136/gutjnl-2015-310187 26464414

[42] Luoma, A. M. et al. Molecular pathways of colon inflammation induced by cancer immunotherapy. *Cell* **182**, 655–671 (2020). 10.1016/j.cell.2020.06.001 PMC7415717 32603654

[43] Stallhofer, J. et al. Lipocalin-2 is a disease activity marker in inflammatory bowel disease regulated by IL-17A, IL-22, and TNF-α and modulated by IL23R genotype status. *Inflamm. Bowel Dis.* **21**, 2327–2340 (2015). 10.1097/MIB.0000000000000515 26263469

[44] Zou, F. et al. Fecal calprotectin concentration to assess endoscopic and histologic remission in patients with cancer with immunemediated diarrhea and colitis. *J. Immunother. Cancer* **9**, e002058 (2021). 10.1136/jitc-2020-002058 PMC7805368 33436487

[45] Sasson, S. C. et al. Interferon-gamma-producing CD8^(+)^ tissue resident memory T cells are a targetable hallmark of immune checkpoint inhibitor-colitis. *Gastroenterology* **161**, 1229–1244 (2021). 10.1053/j.gastro.2021.06.025 PMC8527886 34147519

[46] Nunez, N. G. et al. Immune signatures predict development of autoimmune toxicity in patients with cancer treated with immune checkpoint inhibitors. *Med* **4**, 113–129 (2023). 10.1016/j.medj.2022.12.007 36693381

[47] Beck, K. E. et al. Enterocolitis in patients with cancer after antibody blockade of cytotoxic T-lymphocyte-associated antigen 4. *J. Clin. Oncol.* **24**, 2283–2289 (2006). 10.1200/JCO.2005.04.5716 PMC2140223 16710025

[48] Powell, N. et al. British Society of Gastroenterology endorsed guidance for the management of immune checkpoint inhibitor-induced enterocolitis. *Lancet Gastroenterol. Hepatol.* **5**, 679–697 (2020). 10.1016/S2468-1253(20)30014-5 32553146

[49] Planey, S. L. & Litwack, G. Glucocorticoid-induced apoptosis in lymphocytes. *Biochem. Biophys. Res. Commun.* **279**, 307–312 (2000). 10.1006/bbrc.2000.3922 11118283

[50] Luo, J. et al. Beyond steroids: immunosuppressants in steroid-refractory or resistant immune-related adverse events. *J. Thorac. Oncol.* **16**, 1759–1764 (2021). 10.1016/j.jtho.2021.06.024 PMC8464489 34265432

[51] Horvat, T. Z. et al. Immune-related adverse events, need for systemic immunosuppression, and effects on survival and time to treatment failure in patients with melanoma treated with ipilimumab at Memorial Sloan Kettering Cancer Center. *J. Clin. Oncol.* **33**, 3193–3198 (2015). 10.1200/JCO.2015.60.8448 PMC5087335 26282644

[52] Marschner, D. et al. MicroRNA-146a regulates immune-related adverse events caused by immune checkpoint inhibitors. *JCI Insight* **5**, e132334 (2020). 10.1172/jci.insight.132334 PMC7213806 32125286

[53] Petrelli, F. et al. Association of steroids use with survival in patients treated with immune checkpoint inhibitors: a systematic review and meta-analysis. *Cancers* **12**, 546 (2020). 10.3390/cancers12030546 PMC7139305 32120803

[54] Burla, J. et al. Retrospective analysis of treatment and complications of immune checkpoint inhibitor-associated colitis: histological ulcerations as potential predictor for a steroid-refractory disease course. *Inflamm. Intest. Dis.* **5**, 109–116 (2020). 10.1159/000507579 PMC7506225 32999883

[55] Wang, Y. et al. Immune-checkpoint inhibitor-induced diarrhea and colitis in patients with advanced malignancies: retrospective review at MD Anderson. *J. Immunother. Cancer* **6**, 37 (2018). 10.1186/s40425-018-0346-6 PMC5946546 29747688

[56] Abu-Sbeih, H. et al. Early introduction of selective immunosuppressive therapy associated with favorable clinical outcomes in patients with immune checkpoint inhibitor-induced colitis. *J. Immunother. Cancer* **7**, 93 (2019). 10.1186/s40425-019-0577-1 PMC6444537 30940209

[57] Holmstroem, R. B. et al. COLAR: open-label clinical study of IL-6 blockade with tocilizumab for the treatment of immune checkpoint inhibitor-induced colitis and arthritis. *J. Immunother. Cancer* **10**, e005111 (2022). 10.1136/jitc-2022-005111 PMC9472120 36096534

[58] Harvey, C. et al. Management of infliximab refractory immune checkpoint inhibitor gastrointestinal toxicity: a multicenter case series. *J. Immunother. Cancer* **12**, e008232 (2024). 10.1136/jitc-2023-008232 PMC10831444 38296594

[59] Andersen, N. N. & Jess, T. Risk of infections associated with biological treatment in inflammatory bowel disease. *World J. Gastroenterol.* **20**, 16014–16019 (2014). 10.3748/wjg.v20.i43.16014 PMC4239487 25473153

[60] Karlqvist, S. et al. Comparative risk of serious infection with vedolizumab vs anti-tumor necrosis factor in inflammatory bowel disease: results from Nationwide Swedish Registers. *Am. J. Gastroenterol.* **119**, 2480–2492 (2024). 10.14309/ajg.0000000000002961 PMC11608631 38994835

[61] Berger, C. et al. Rapid generation of maturationally synchronized human dendritic cells: contribution to the clinical efficacy of extracorporeal photochemotherapy. *Blood* **116**, 4838–4847 (2010). 10.1182/blood-2009-11-256040 PMC3321745 20720185

[62] Schmitt, I. M. et al. Increased surface expression of class I MHC molecules on immunogenic cells derived from the xenogenization of P815 mastocytoma cells with 8-methoxypsoralen and long-wavelength ultraviolet radiation. *Tissue Antigens* **46**, 45–49 (1995). 10.1111/j.1399-0039.1995.tb02474.x 7482495

[63] Ventura, A. et al. Extracorporeal photochemotherapy drives monocyte-to-dendritic cell maturation to induce anticancer immunity. *Cancer Res.* **78**, 4045–4058 (2018). 10.1158/0008-5472.CAN-18-0171 29764863

[64] National Cancer Institute, U.S. Department of Health and Human Services. *Common Terminology Criteria for Adverse Events (CTCAE) v5.0.* (National Cancer Institute, U.S. Department of Health and Human Services, 2017).

[65] Gerlach, U. et al. Centrosome-, chromosomal-passenger- and cell-cycle-associated mRNAs are differentially regulated in the development of sporadic colorectal cancer. *J. Pathol.* **208**, 462–472 (2006). 10.1002/path.1914 16402339

[66] Wehrle, J. et al. Personalized treatment selection and disease monitoring using circulating tumor DNA profiling in real-world cancer patient management. *Diagnostics* **10**, 550 (2020). 10.3390/diagnostics10080550 PMC7459590 32748806

