# Supplementary material for: Extracorporeal photopheresis for the treatment of steroid-refractory immune related adverse events from immune checkpoint inhibitor therapy: a multi-center phase Ib/II clinical trial
Source: Nat Commun. 2026 Oct 23;17:11078. doi: 10.1038/s41467-026-78897-9 (PMC13694692; doi:10.1038/s41467-026-78897-9)
Supplement: Supplementary file 1 [file 41467_2026_78897_MOESM1_ESM.pdf]

Supplementary Information

Extracorporeal photopheresis for the treatment of steroid-refractory immune related adverse events from immune checkpoint inhibitor therapy: a multi-center phase Ib/II clinical trial.

Roxane Riemer[1,*], Lukas M. Braun[1,2,*], Sophie Giesler[3,4], David Rafei-Shamsabadi[5], Sebastian Haferkamp[6], Alina Hartmann[1], Wolfgang Melchinger[1], Matthias Weiss[1], Stefanie Birth[1], Ulrike Philipp[1], Inga Gebhard[1], Carsten Schwänen[7], James A. Hutchinson[8], Nikolaos Karantzelis[9], Till Wallrabenstein[10], Theresa Lowinus[1], Niklas Brehm[1], Justyna Rawluk[1], Dietmar Pfeifer[1], Natascha Osswald[1], Peter Bronsert[11], Manuel Rogg[11], Grigor Andreev[11], Melanie Boerries[2,12], Maximilian Lammer[13], Van Anh Nguyen[13], Matthias Schmuth[13], Natalie Köhler[1,14], Justus Duyster[1,2], Gabriele Ihorst[15], Philip Keye[16], Susana Minguet[17], Dirk Schadendorf[18,19], Selma Ugurel[20], Robert Thimme[21], Peter Hasselblatt[21], Erika L. Pearce[22], Burkhard Becher[23], Carolin Funke-Lorenz[18], Florian Scherer[1], Bertram Bengsch[2,21,24], Christoph Schell[11], Jan-Malte Placke[18,$], Frank Meiss[5,$], Petya Apostolova[1,2,25,26,$], Robert Zeiser[1,2,24,$,#]

[1] Department of Internal Medicine I, Medical Center - University of Freiburg, Faculty of Medicine, University of Freiburg, Freiburg, Germany

[2] German Cancer Consortium (DKTK), Partner site Freiburg, a partnership between DKFZ and Medical Center - University of Freiburg

[3] Department of BioMedical Research, Tumor Immunology, University of Bern, Bern, Switzerland

[4] Department of Medical Oncology, Inselspital, Bern University Hospital, University of Bern, Bern, Switzerland

[5] Department of Dermatology, Medical Center – University of Freiburg, Faculty of Medicine, University of Freiburg, Freiburg, Germany

[6] Department of Dermatology, University Hospital Regensburg, Regensburg, Germany

[7] Division of Hematology, Oncology, Palliative care, Ortenau Hospital Offenburg-Kehl, Offenburg Kehl, Germany

[8] Department of Surgery, University Hospital Regensburg, Regensburg, Germany

[9] Department of Medical Oncology and Hematology, University Hospital of Zurich, Zurich, Switzerland

[10] Center for Hematology and Oncology, Kantonspital Baselland, Bruderholz, Switzerland

[11] Institute of Surgical Pathology, Medical Center - University of Freiburg, Faculty of Medicine, University of Freiburg, Freiburg, Germany

[12] Institute of Medical Bioinformatics and Systems Medicine, Medical Center - University of Freiburg, Faculty of Medicine, University of Freiburg, Freiburg, Germany

[13] Department of Dermatology, Venereology and Allergy, Medical University Innsbruck, Innsbruck, Austria

[14] CIBSS – Centre for Integrative Biological Signalling Studies, University of Freiburg, Freiburg, Germany

[15] Clinical Trials Unit, Medical Center – University of Freiburg, Faculty of Medicine, University of Freiburg, Freiburg, Germany

[16] Eye Center, Medical Center – University of Freiburg, Faculty of Medicine, University of Freiburg, Freiburg, Germany

[17] Signalling Research Centres BIOSS and CIBSS, Freiburg. Germany. Department of Synthetic Immunology, Faculty of Biology and Centre for Chronic Immunodeficiency (CCI), Faculty of Medicine, University of Freiburg, Freiburg, Germany

[18] Department of Dermatology, University Hospital Essen, University of Duisburg-Essen, Essen, and German Cancer Consortium (DKTK), Partner Site Essen/Duesseldorf, Germany

[19] National Center for Tumor Diseases (NCT)-West, Campus Essen, & Research Alliance Ruhr, Research Center One Health, University Duisburg-Essen, Essen, Germany

[20] Department of Dermatology, Bielefeld University, Medical School and University Medical Center OWL, Klinikum Bielefeld Rosenhöhe, Bielefeld, Germany

[21] Department of Internal Medicine II (Gastroenterology, Hepatology, Endocrinology and Infectious Diseases), Medical Center - University of Freiburg, Faculty of Medicine, University of Freiburg, Freiburg, Germany

[22] Department of Oncology, The Bloomberg-Kimmel Institute for Cancer Immunotherapy, Johns Hopkins School of Medicine, Baltimore, MD 21287, USA

[23] Institute of Experimental Immunology, University of Zurich, Zurich, Switzerland

[24] Signalling Research Centres BIOSS and CIBSS – Centre for Integrative Biological Signalling Studies, University of Freiburg, Freiburg, Germany

[25] Department of Biomedicine, Faculty of Medicine, University Hospital Basel and University of Basel, Basel, Switzerland

[26] Division of Hematology, University Hospital Basel, Basel, Switzerland

* These authors contributed equally: Roxane Riemer, Lukas M. Braun

[$] These authors jointly supervised this work: Jan-Malte Placke, Frank Meiss, Petya Apostolova, Robert Zeiser

#Corresponding author:

Robert Zeiser, MD
Department of Hematology, Oncology and Stem Cell Transplantation, University Medical Center Freiburg, 79106 Freiburg, Germany
Tel: +49-761-270-34580, Fax: +49-761-270-73570
robert.zeiser@uniklinik-freiburg.de

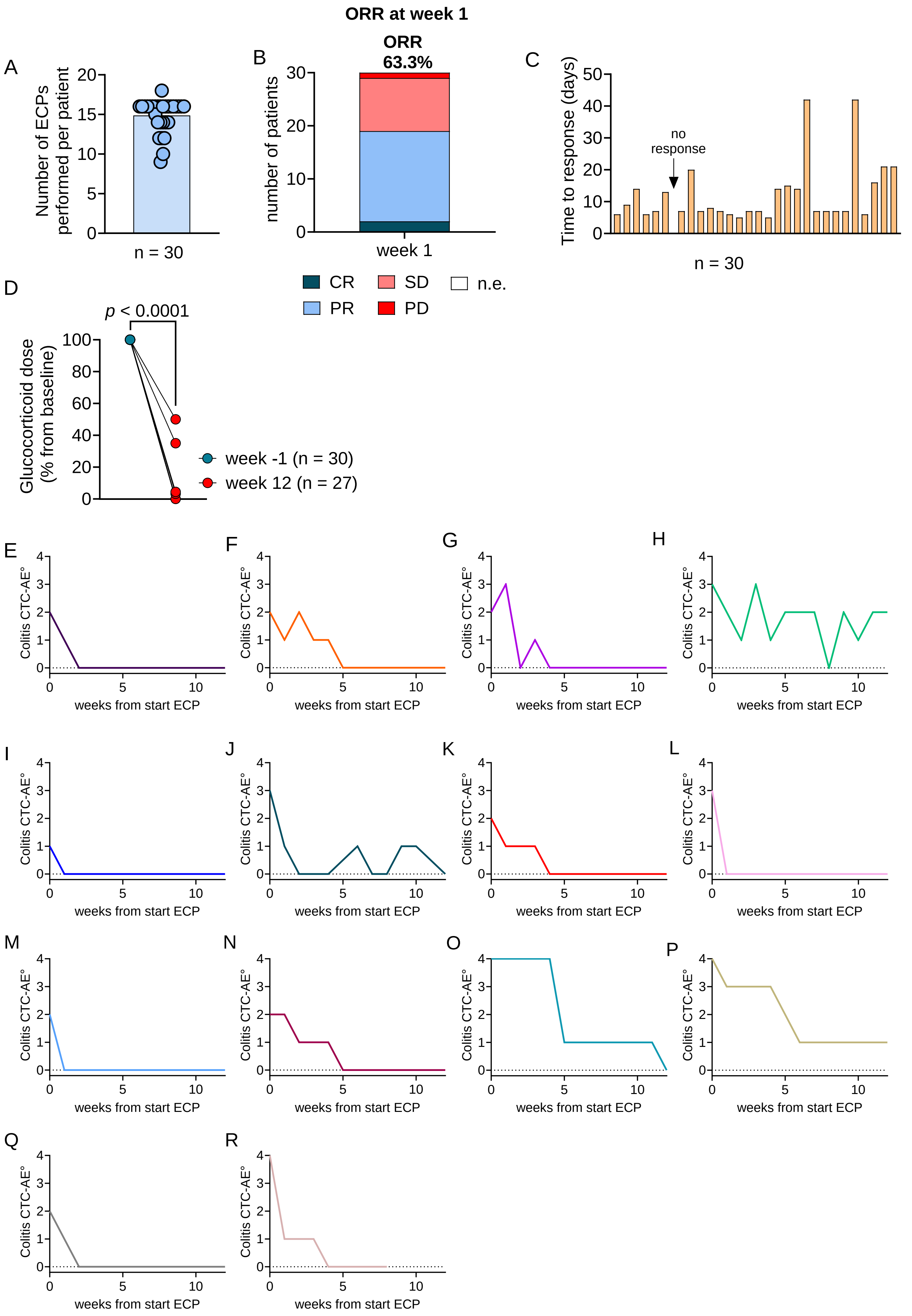

ORR at week 1
A
Number of ECPs performed per patient
n = 30
B
ORR 63.3%
number of patients
week 1
CR
SD
n.e.
PR
PD
C
Time to response (days)
no response
n = 30
D
p < 0.0001
Glucocorticoid dose (% from baseline)
week -1 (n = 30)
week 12 (n = 27)
E
F
G
H
I
J
K
L
M
N
O
P
Q
R
Colitis CTC-AE°
weeks from start ECP

Suppl. Figure 1: ECP interventions performed

A) The scatter bar graph indicates mean numbers of ECP sessions (±SEM) of n=30 patients treated in the prospective phase Ib/II clinical trial during the study period (range 9-18, median 16). Each dot represents one patient. Some patients received a reduced number of ECPs because of infections (n=2), premature EOS (n=2), premature EOT (n=1), hospitalization (n=1), poor peripheral venous access (n=1) or personal reasons (n=4).

B) Response assessment of immune-related adverse events (irAE) after the first ECP (1 × 2 ECP sessions) in week 1. The graph shows absolute numbers of patients with complete response (CR), partial response (PR), stable disease (SD) and progressive disease (PD) of n=30 patients included in the prospective clinical phase Ib/II trial. IrAEs were graded according to CTC-AE V5.0. Overall response rate (ORR) in percent at week 1 after start of the ECP treatment is indicated above the bar.

C) The plot shows the time to response after ECP-therapy in days per patient. A response to therapy was counted if patients achieved a reduction of at least one CTC-AE grade of at least one organ system involved compared to CTC-AE grade prior to the first ECP. Each bar represents one patient.

D) The graph shows the relative reduction of GC as percentage from baseline after 12 weeks of ECP-therapy (week 12) compared to steroid dose before ECP (week -1). Each dot represents an individual patient. Only patients with values in week -1 and 12 are shown (n=27). In three patients, steroid dose was not available in week 12 (n=2 premature EOS, n=1 premature EOT). The *p*-value was calculated using a two-tailed one sample Wilcoxon signed-rank test.

E-R) Time course of ICI-colitis grades of individual patients during ECP treatment. Colitis severity of patients included in the trial was graded according to CTC-AE V5.0 based on diarrhea frequency at weekly timepoints until end of treatment in week 12. Each graph represents one individual patient with ICI-colitis.

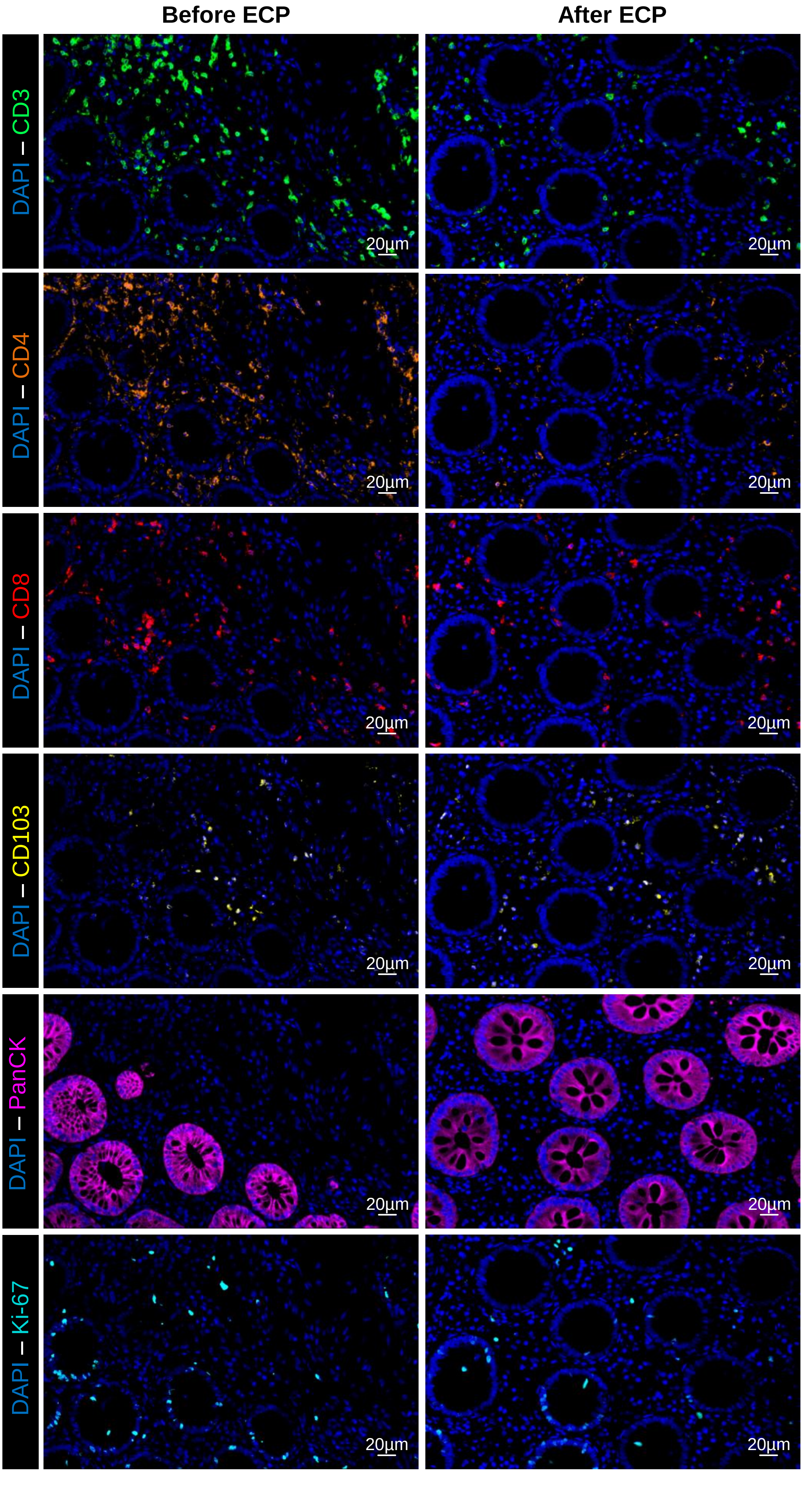

Suppl. Figure 2: Multiplex immunofluorescence of colon tissue from an ICI-colitis patient before and after ECP-therapy. Individual staining before (left) and after (right) ECP-therapy is shown. Staining shows CD3, CD4, CD8 and CD103 expressing immune cells, expression of Ki67, and cells positive for cytokeratin (pan-CK). The scale bar is 20µm.

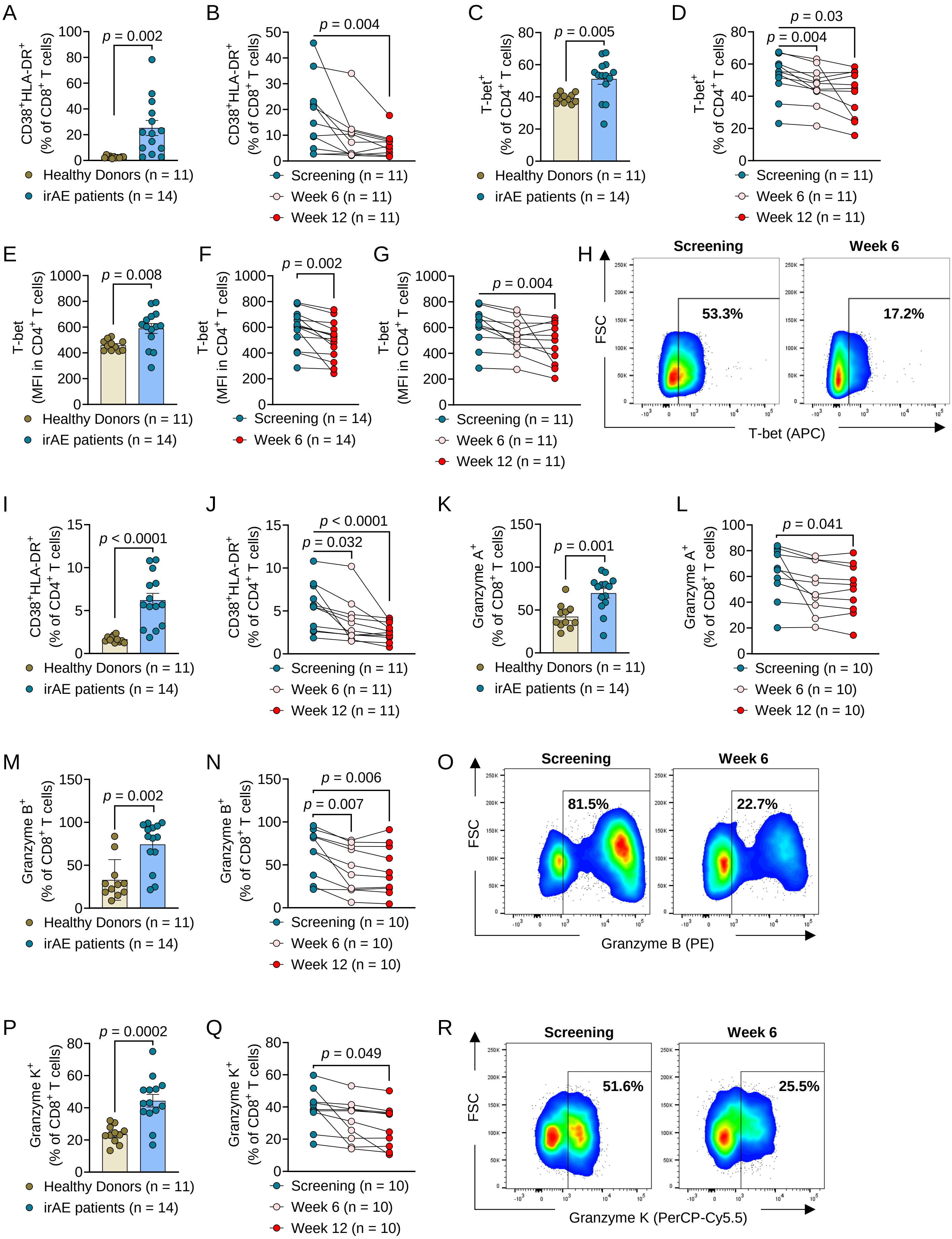

A
p = 0.002
CD38+HLA-DR+ (% of CD8+ T cells)
Healthy Donors (n = 11)
irAE patients (n = 14)
B
p = 0.004
CD38+HLA-DR+ (% of CD8+ T cells)
Screening (n = 11)
Week 6 (n = 11)
Week 12 (n = 11)
C
p = 0.005
T-bet+ (% of CD4+ T cells)
Healthy Donors (n = 11)
irAE patients (n = 14)
D
p = 0.03
p = 0.004
T-bet+ (% of CD4+ T cells)
Screening (n = 11)
Week 6 (n = 11)
Week 12 (n = 11)
E
p = 0.008
T-bet (MFI in CD4+ T cells)
Healthy Donors (n = 11)
irAE patients (n = 14)
F
p = 0.002
T-bet (MFI in CD4+ T cells)
Screening (n = 14)
Week 6 (n = 14)
G
p = 0.004
T-bet (MFI in CD4+ T cells)
Screening (n = 11)
Week 6 (n = 11)
Week 12 (n = 11)
H
Screening
Week 6
53.3%
17.2%
FSC
T-bet (APC)
I
p < 0.0001
CD38+HLA-DR+ (% of CD4+ T cells)
Healthy Donors (n = 11)
irAE patients (n = 14)
J
p < 0.0001
p = 0.032
CD38+HLA-DR+ (% of CD4+ T cells)
Screening (n = 11)
Week 6 (n = 11)
Week 12 (n = 11)
K
p = 0.001
Granzyme A+ (% of CD8+ T cells)
Healthy Donors (n = 11)
irAE patients (n = 14)
L
p = 0.041
Granzyme A+ (% of CD8+ T cells)
Screening (n = 10)
Week 6 (n = 10)
Week 12 (n = 10)
M
p = 0.002
Granzyme B+ (% of CD8+ T cells)
Healthy Donors (n = 11)
irAE patients (n = 14)
N
p = 0.006
p = 0.007
Granzyme B+ (% of CD8+ T cells)
Screening (n = 10)
Week 6 (n = 10)
Week 12 (n = 10)
O
Screening
Week 6
81.5%
22.7%
FSC
Granzyme B (PE)
P
p = 0.0002
Granzyme K+ (% of CD8+ T cells)
Healthy Donors (n = 11)
irAE patients (n = 14)
Q
p = 0.049
Granzyme K+ (% of CD8+ T cells)
Screening (n = 10)
Week 6 (n = 10)
Week 12 (n = 10)
R
Screening
Week 6
51.6%
25.5%
FSC
Granzyme K (PerCP-Cy5.5)

Suppl. Figure 3: Immune cell phenotyping in peripheral blood samples of healthy donors and irAE patients during ECP-therapy (activation and granzymes)

A, B) Frequency of $CD38^{+}HLA\text{-}DR^{+}$ cells as percent of all $CD8^{+}$ T cells **(A)** in the peripheral blood of healthy donors and irAE patients (mean±SEM), **(B)** or irAE patients at screening, and at week 6 and week 12 of ECP-therapy.

C-H) Frequency of T-bet^{+} cells as percent of all $CD4^{+}$ T cells **(C)** in the peripheral blood of healthy donors and irAE patients (mean±SEM), **(D)** or irAE patients at screening, and at week 6 and week 12 of ECP-therapy. MFI of T-bet in $CD4^{+}$ T cells **(E)** in the peripheral blood of healthy donors and irAE patients (mean±SEM), **(F)** irAE patients at screening and at week 6 of ECP-therapy, **(G)** or irAE patients at screening, and at week 6 and week 12 of ECP-therapy. **(H)** Representative flow cytometry plots of T-bet expression in $CD4^{+}$ T cells.

I, J) Frequency of $CD38^{+}HLA\text{-}DR^{+}$ cells as percent of all $CD4^{+}$ T cells **(I)** in the peripheral blood of healthy donors and irAE patients (mean±SEM), **(J)** or irAE patients at screening, and at week 6 and week 12 of ECP-therapy.

K, L) Frequency of Granzyme A expressing cells as percent of all $CD8^{+}$ T cells **(K)** in the peripheral blood of healthy donors and irAE patients (mean±SEM), **(L)** or irAE patients at screening, and at week 6 and week 12 of ECP-therapy.

M-O) Frequency of Granzyme B expressing cells as percent of all $CD8^{+}$ T cells **(M)** in the peripheral blood of healthy donors and irAE patients (mean±SEM), **(N)** or irAE patients at screening, and at week 6 and week 12 of ECP-therapy. **(O)** Representative flow cytometry plots of Granzyme B in $CD8^{+}$ T cells.

P-R) Frequency of Granzyme K expressing cells as percent of all $CD8^{+}$ T cells **(P)** in the peripheral blood of healthy donors and irAE patients (mean±SEM), **(Q)** or irAE patients at screening, and at week 6 and week 12 of ECP-therapy. **(R)** Representative flow cytometry plots of Granzyme K in $CD8^{+}$ T cells.

The number of analyzed healthy controls or irAE patients is included in each graph.

The *p*-values were calculated using a two-tailed Student's unpaired *t*-test (A, C, E, I, K, P), a two-tailed Student's paired *t*-test (F), a Friedman test with Dunn's multiple testing (B, J), a repeated measures one-way ANOVA with Tukey's multiple comparison test (D, G, L, N, Q), or a two-tailed Mann-Whitney test (M).

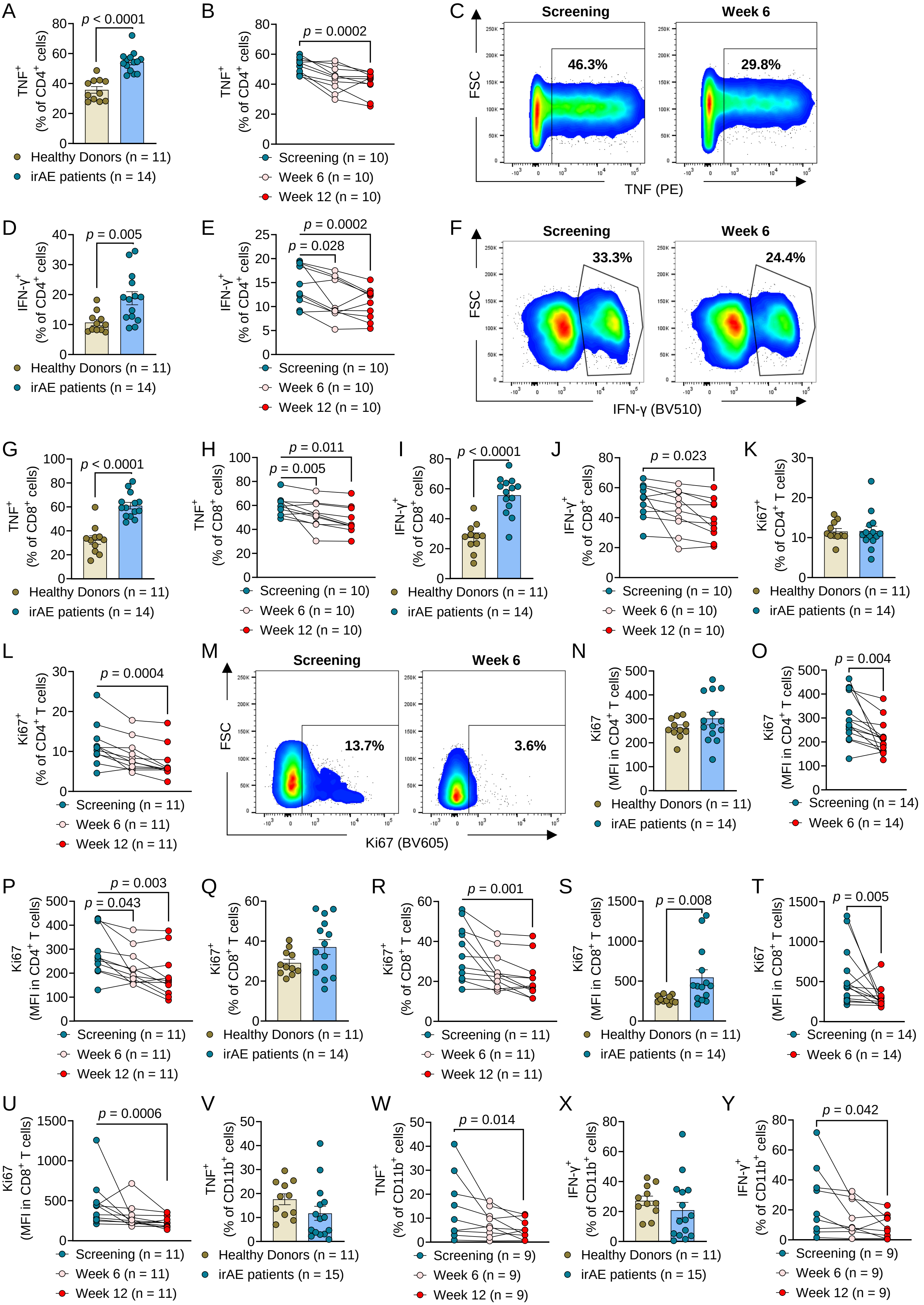
A
TNF+ (% of CD4+ cells)
p < 0.0001
Healthy Donors (n = 11)
irAE patients (n = 14)
B
TNF+ (% of CD4+ cells)
p = 0.0002
Screening (n = 10)
Week 6 (n = 10)
Week 12 (n = 10)
C
Screening
Week 6
46.3%
29.8%
FSC
TNF (PE)
D
IFN-γ+ (% of CD4+ cells)
p = 0.005
Healthy Donors (n = 11)
irAE patients (n = 14)
E
IFN-γ+ (% of CD4+ cells)
p = 0.0002
p = 0.028
Screening (n = 10)
Week 6 (n = 10)
Week 12 (n = 10)
F
Screening
Week 6
33.3%
24.4%
FSC
IFN-γ (BV510)
G
TNF+ (% of CD8+ cells)
p < 0.0001
Healthy Donors (n = 11)
irAE patients (n = 14)
H
TNF+ (% of CD8+ cells)
p = 0.011
p = 0.005
Screening (n = 10)
Week 6 (n = 10)
Week 12 (n = 10)
I
IFN-γ+ (% of CD8+ cells)
p < 0.0001
Healthy Donors (n = 11)
irAE patients (n = 14)
J
IFN-γ+ (% of CD8+ cells)
p = 0.023
Screening (n = 10)
Week 6 (n = 10)
Week 12 (n = 10)
K
Ki67+ (% of CD4+ T cells)
Healthy Donors (n = 11)
irAE patients (n = 14)
L
Ki67+ (% of CD4+ T cells)
p = 0.0004
Screening (n = 11)
Week 6 (n = 11)
Week 12 (n = 11)
M
Screening
Week 6
13.7%
3.6%
FSC
Ki67 (BV605)
N
Ki67 (MFI in CD4+ T cells)
Healthy Donors (n = 11)
irAE patients (n = 14)
O
Ki67 (MFI in CD4+ T cells)
p = 0.004
Screening (n = 14)
Week 6 (n = 14)
P
Ki67 (MFI in CD4+ T cells)
p = 0.003
p = 0.043
Screening (n = 11)
Week 6 (n = 11)
Week 12 (n = 11)
Q
Ki67+ (% of CD8+ T cells)
Healthy Donors (n = 11)
irAE patients (n = 14)
R
Ki67+ (% of CD8+ T cells)
p = 0.001
Screening (n = 11)
Week 6 (n = 11)
Week 12 (n = 11)
S
Ki67 (MFI in CD8+ T cells)
p = 0.008
Healthy Donors (n = 11)
irAE patients (n = 14)
T
Ki67 (MFI in CD8+ T cells)
p = 0.005
Screening (n = 14)
Week 6 (n = 14)
U
Ki67 (MFI in CD8+ T cells)
p = 0.0006
Screening (n = 11)
Week 6 (n = 11)
Week 12 (n = 11)
V
TNF+ (% of CD11b+ cells)
Healthy Donors (n = 11)
irAE patients (n = 15)
W
TNF+ (% of CD11b+ cells)
p = 0.014
Screening (n = 9)
Week 6 (n = 9)
Week 12 (n = 9)
X
IFN-γ+ (% of CD11b+ cells)
Healthy Donors (n = 11)
irAE patients (n = 15)
Y
IFN-γ+ (% of CD11b+ cells)
p = 0.042
Screening (n = 9)
Week 6 (n = 9)
Week 12 (n = 9)

Suppl. Figure 4: Immune cell phenotyping in peripheral blood samples of healthy donors and irAE patients during ECP-therapy (cytokines and proliferation)

A-C) Frequency of TNF expressing cells as percent of all $CD4^+$ T cells **(A)** in the peripheral blood of healthy donors and irAE patients (mean±SEM), **(B)** or irAE patients at screening, and at week 6 and week 12 of ECP-therapy. **(C)** Representative flow cytometry plots of TNF in $CD4^+$ T cells.

D-F) Frequency of IFN-γ expressing cells as percent of all $CD4^+$ T cells **(D)** in the peripheral blood of healthy donors and irAE patients (mean±SEM), **(E)** or irAE patients at screening, and at week 6 and week 12 of ECP-therapy. **(F)** Representative flow cytometry plots of IFN-γ in $CD4^+$ T cells.

G, H) Frequency of TNF expressing cells as percent of all $CD8^+$ T cells **(G)** in the peripheral blood of healthy donors and irAE patients (mean±SEM), **(H)** or irAE patients at screening, and at week 6 and week 12 of ECP-therapy.

I, J) Frequency of IFN-γ expressing cells as percent of all $CD8^+$ T cells **(I)** in the peripheral blood of healthy donors and irAE patients (mean±SEM), **(J)** or irAE patients at screening, and at week 6 and week 12 of ECP-therapy.

K-M) Frequency of $Ki67^+$ cells as percent of all $CD4^+$ T cells **(K)** in the peripheral blood of healthy donors and irAE patients (mean±SEM), **(L)** or irAE patients at screening, and at week 6 and week 12 of ECP-therapy. **(M)** Representative flow cytometry plots of Ki67 expression in $CD4^+$ T cells.

N-P) MFI of Ki67 in $CD4^+$ T cells **(N)** in the peripheral blood of healthy donors and irAE patients (mean±SEM), **(O)** irAE patients at screening and week 6 of ECP-therapy, **(P)** or irAE patients at screening, and at week 6 and week 12 of ECP-therapy.

Q, R) Frequency of $Ki67^+$ cells as percent of all $CD8^+$ T cells **(Q)** in the peripheral blood of healthy donors and irAE patients (mean±SEM), **(R)** or irAE patients at screening, and at week 6 and week 12 of ECP-therapy.

S-U) MFI of Ki67 in $CD8^+$ T cells **(S)** in the peripheral blood of healthy donors and irAE patients (mean±SEM), **(T)** irAE patients at screening and week 6 of ECP-therapy, **(U)** or irAE patients at screening, and at week 6 and week 12 of ECP-therapy.

V, W) Frequency of TNF expressing cells as percent of all $CD11b^+$ T cells **(V)** in the peripheral blood of healthy donors and irAE patients (mean±SEM), **(W)** or irAE patients at screening, and at week 6 and week 12 of ECP-therapy.

X, Y) Frequency of IFN-γ expressing cells as percent of all $CD11b^+$ T cells **(X)** in the peripheral blood of healthy donors and irAE patients (mean±SEM), **(Y)** or irAE patients at screening, and at week 6 and week 12 of ECP-therapy.

The number of analyzed healthy controls or irAE patients is included in each graph.

Significance was calculated using a two-tailed Student's unpaired *t*-test (A, D, G, I, N, Q), a Friedman test with Dunn's multiple testing (B, L, P, R, U, W), a repeated measures one-way ANOVA with Tukey's multiple comparison test (E, H, J, Y), a two-tailed Mann-Whitney test (K, S, V, X), a two-tailed Student's paired *t*-test (O), or a two-tailed Wilcoxon matched-pairs signed-rank test (T).

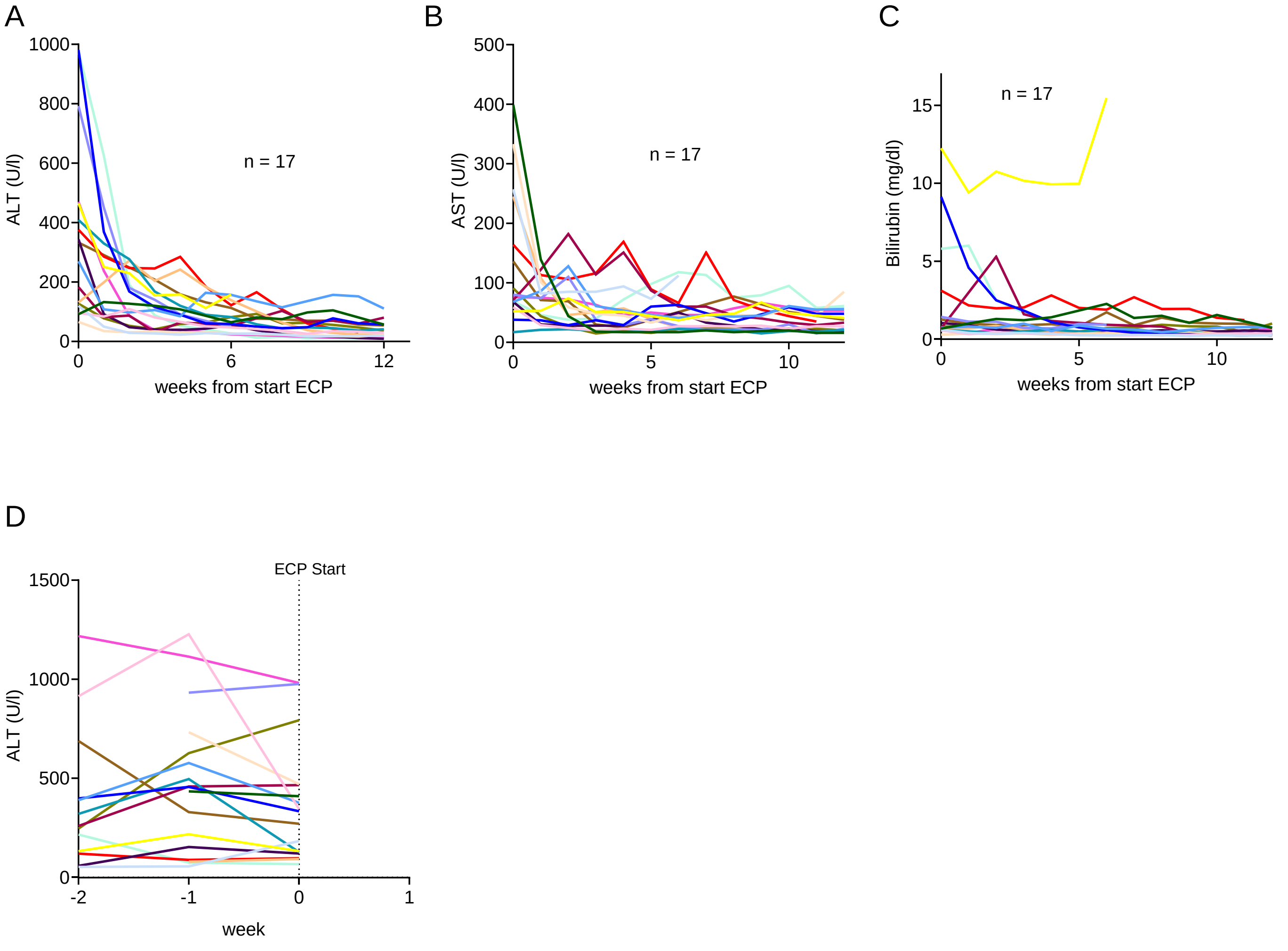

Suppl. Figure 5: Time course of ALT in patients treated with ECP for ICI-hepatitis

A) Time course of absolute ALT values in Units per liter (U/l) of individual patients with ICI-hepatitis during ECP treatment. Each colored line in the graph represents an individual patient. ALT levels were assessed weekly until end of treatment (EOT) in week 12.

B) Time course of absolute AST values in Units per liter (U/l) of individual patients with ICI-hepatitis during ECP treatment. Each colored line in the graph represents an individual patient. AST levels were assessed weekly until end of treatment (EOT) in week 12.

C) Time course of absolute bilirubin values in mg per deciliter (mg/dl) of individual patients with ICI-hepatitis during ECP treatment. Each colored line in the graph represents an individual patient. Bilirubin levels were assessed weekly until end of treatment (EOT) in week 12.

D) Time course of absolute ALT values in Units per liter (U/l) of individual patients with ICI-hepatitis two weeks prior to ECP start. Week minus 1 corresponds with screening visit and week 0 corresponds with the visit in week 1 just prior to the first ECP. Each colored line in the graph represents an individual patient. ALT levels in week -2 were assessed in a post-hoc analysis.

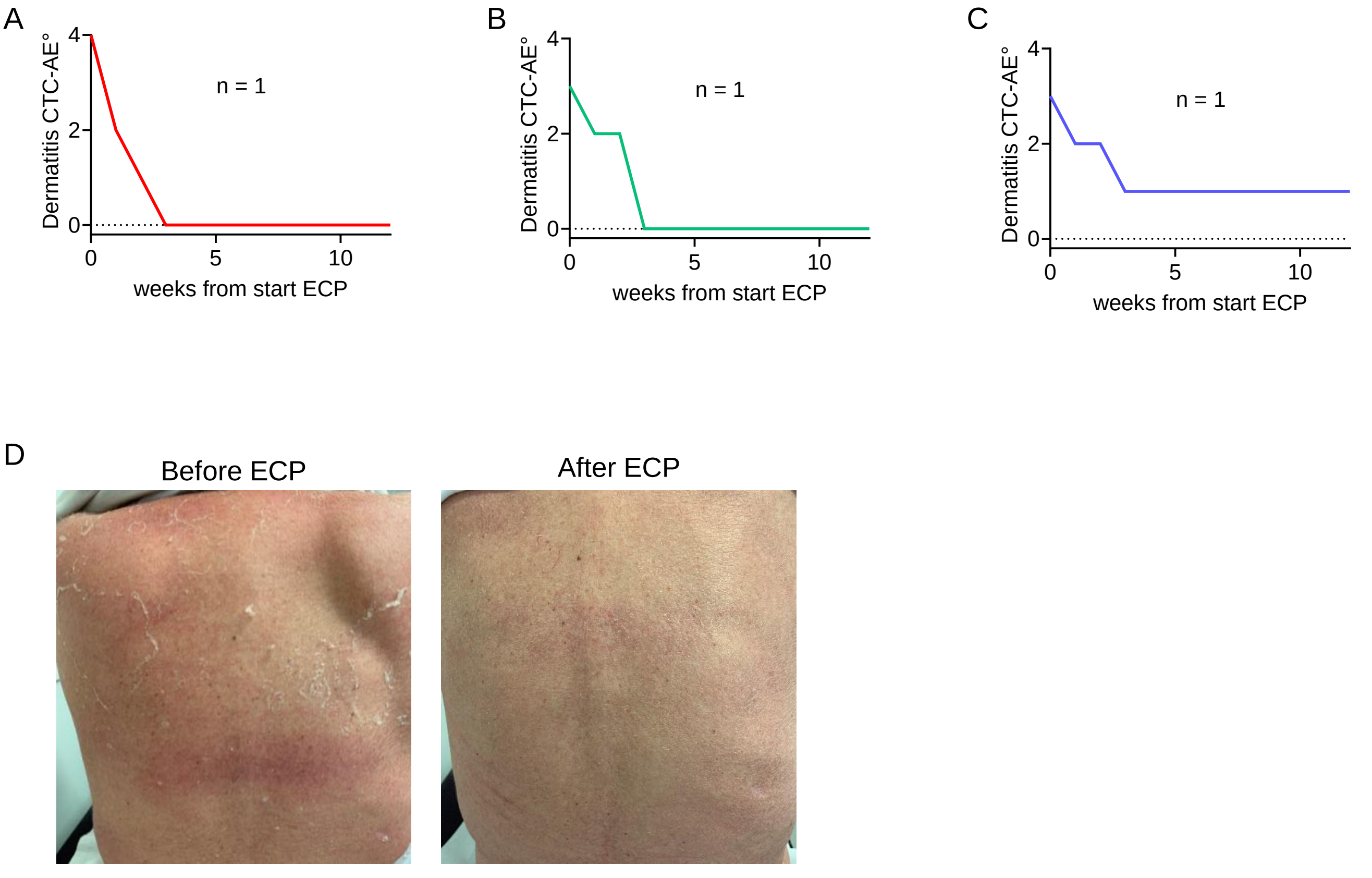

Suppl. Figure 6: Clinical results of ECP in ICI-dermatitis

A-C) Time course of ICI-dermatitis severity during ECP treatment. Dermatitis severity of patients included in the prospective clinical phase Ib/II trial was graded according to CTC-AE V5.0 based on body surface area involved and symptoms at weekly timepoints until end of treatment (EOT) in week 12. Each graph **(A-C)** represents one patient.

D) Representative photographic image of one patient with ICI-dermatitis before and after ECP-therapy. The images were captured before initiation of ECP-therapy (before ECP) and at end of treatment (EOT) in week 12 (after ECP).

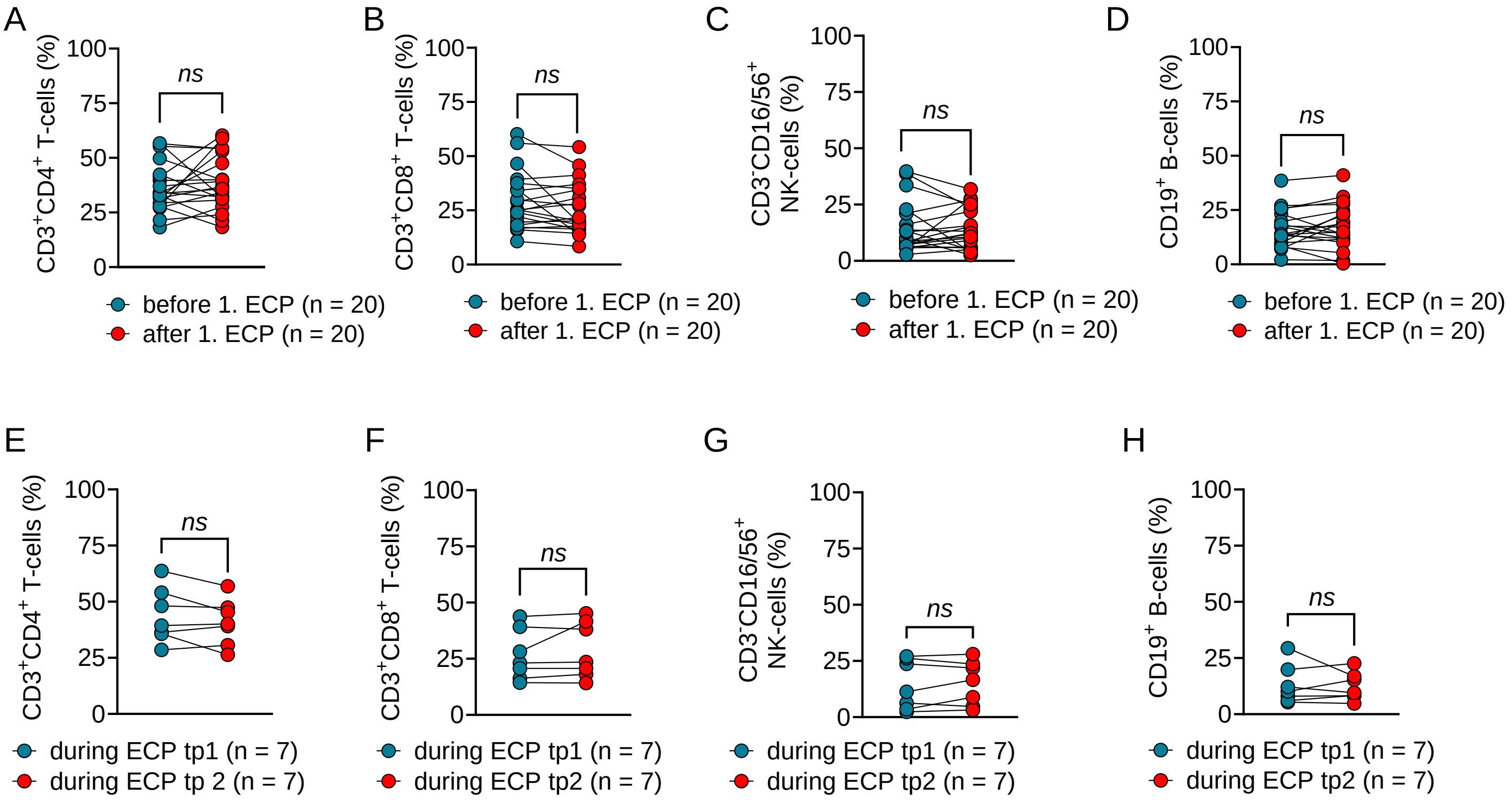

Suppl. Figure 7: Immune cell populations in the peripheral blood during ECP-therapy
A-D) Quantification of peripheral blood lymphocyte subpopulations before the first ECP treatment and after the second ECP treatment (ECP on two consecutive days) using flow cytometry in 20 patients (n=20). Lymphocyte frequencies are shown as percentage of peripheral blood leukocytes in patients included in the prospective clinical phase Ib/II trial. Each dot represents one patient. $CD4^+$ **(A)** and $CD8^+$ T cells **(B)**, NK cells (**C)** and B cells **(D)** were analyzed individually. Significance was calculated using a two-tailed Student's paired *t*-test or a two-tailed Wilcoxon signed-rank test.
E-H) Quantification of peripheral blood lymphocyte subpopulations during two different time points with intervals of at least 1 week between the 2 timepoints of sample collection while ECP-therapy was ongoing using flow cytometry. Analysis was performed in 7 patients treated in the prospective clinical phase Ib/II trial. Relative lymphocyte frequencies of peripheral blood leukocytes are shown. Each dot represents one patient. CD4+ **(E)** and CD8+ T cells **(F)**, NK cells **(G)** and B cells **(H)** were analyzed individually. Significance was calculated using a two-tailed Student's paired *t*-test or a two-tailed Wilcoxon signed-rank test.

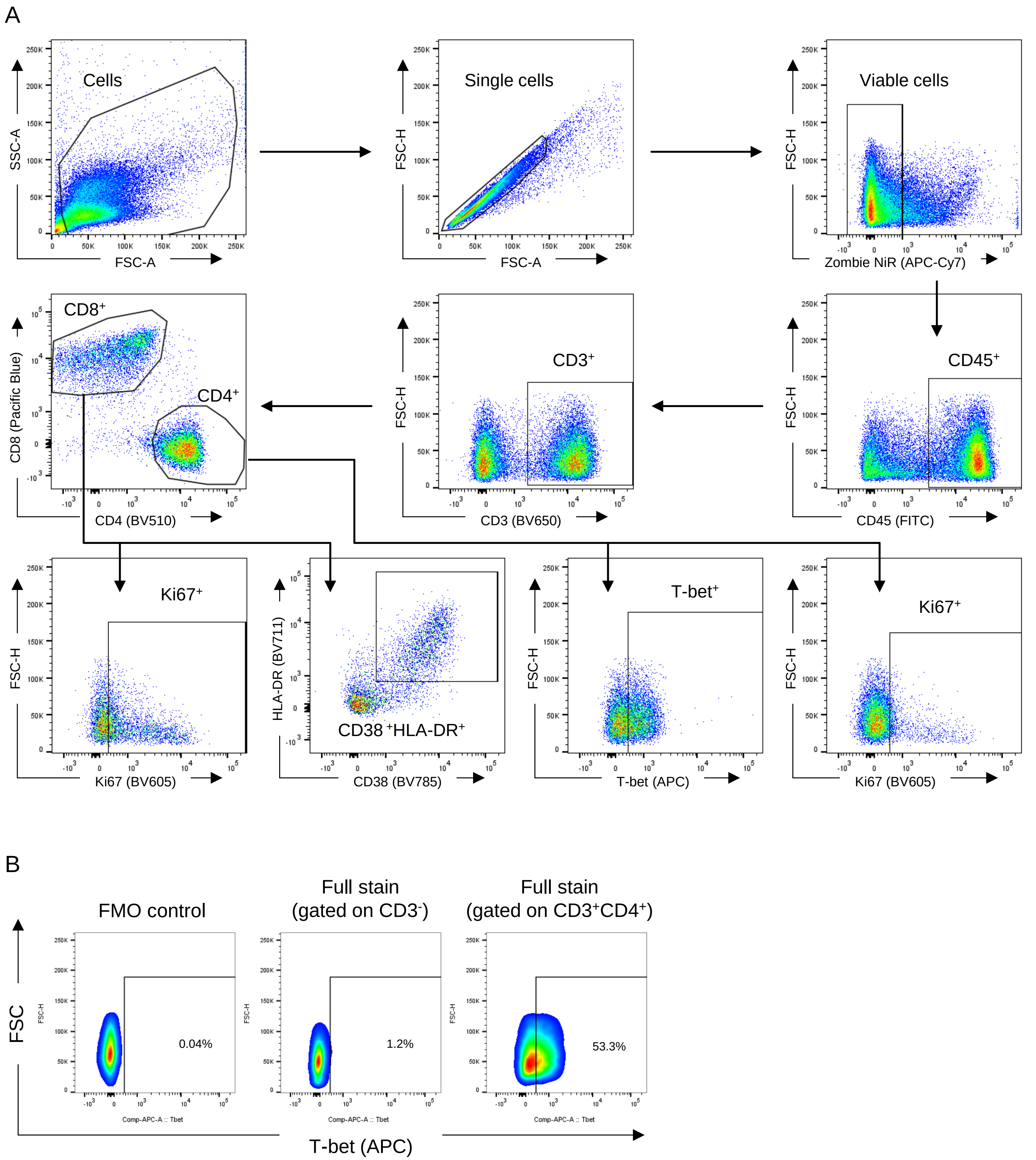

Suppl. Figure 8: Gating strategy for the analysis of activated T cells and Th1 cells

A) Gating strategy for the analysis of $CD4^+$ Th1 cells and $CD38^+HLA\text{-}DR^+$ $CD8^+$ and $CD4^+$ T cells in the peripheral blood of an irAE patient before ECP-therapy.

B) Gates were set using fluorescence-minus-one (FMO) controls. The gating for T-bet was further controlled including cells that do not express T-bet ($CD3^-$).

Gating strategy corresponds to data presented in Figure 4 A-E, Figure 4 P-R, Suppl. Figure 3 A-J, and Suppl. Figure 4 K-U.

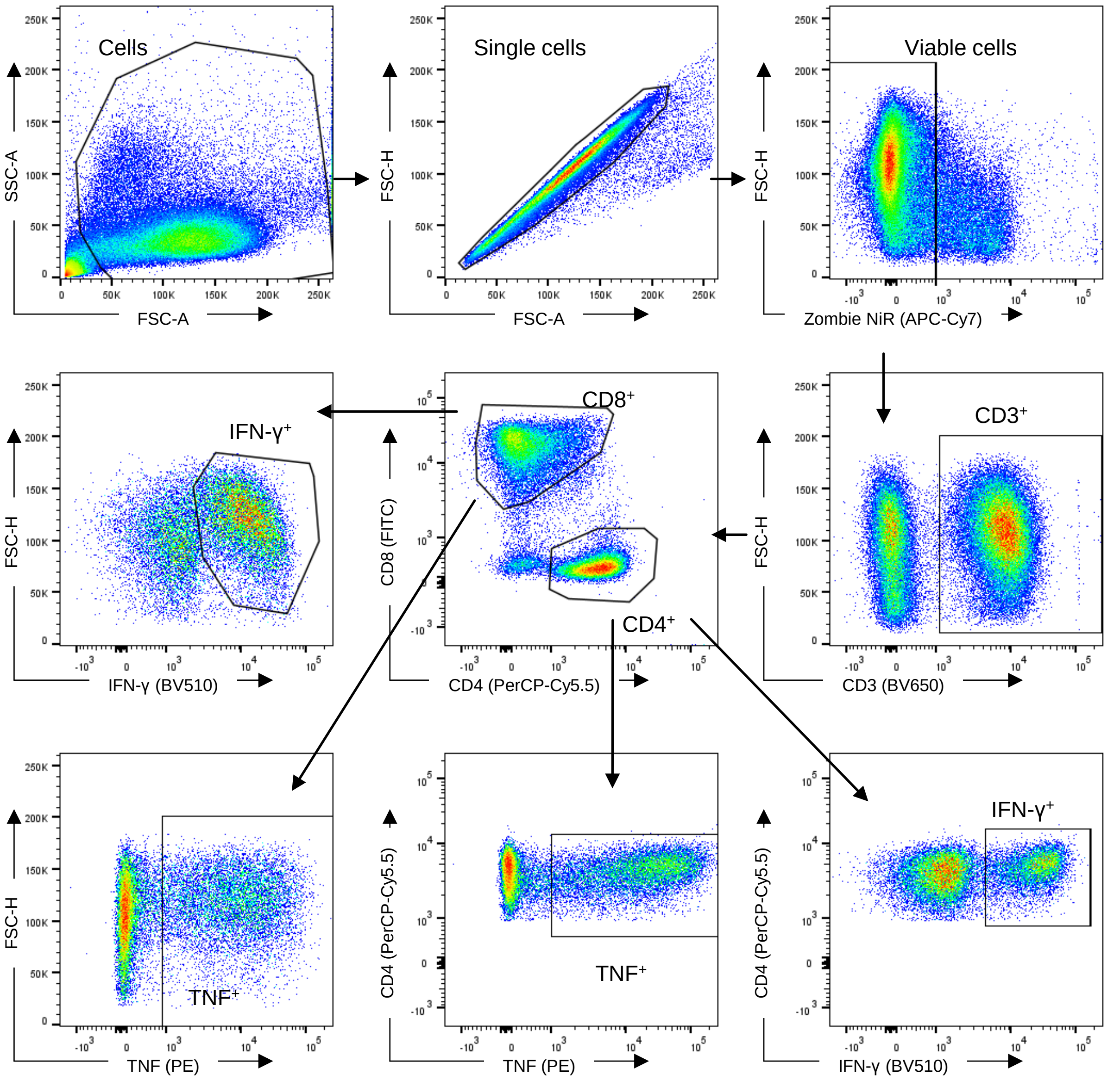

Suppl. Figure 9: Gating strategy for the analysis of IFN-γ and TNF expression in T cells
Gating strategy for the analysis of IFN-γ and TNF production by $CD4^+$ and $CD8^+$ T cells in the peripheral blood of an irAE patient before ECP-therapy. Gates were set using FMO controls. Gating strategy corresponds to data presented in Figure 4 J-O and Suppl. Figure 4 A-J.

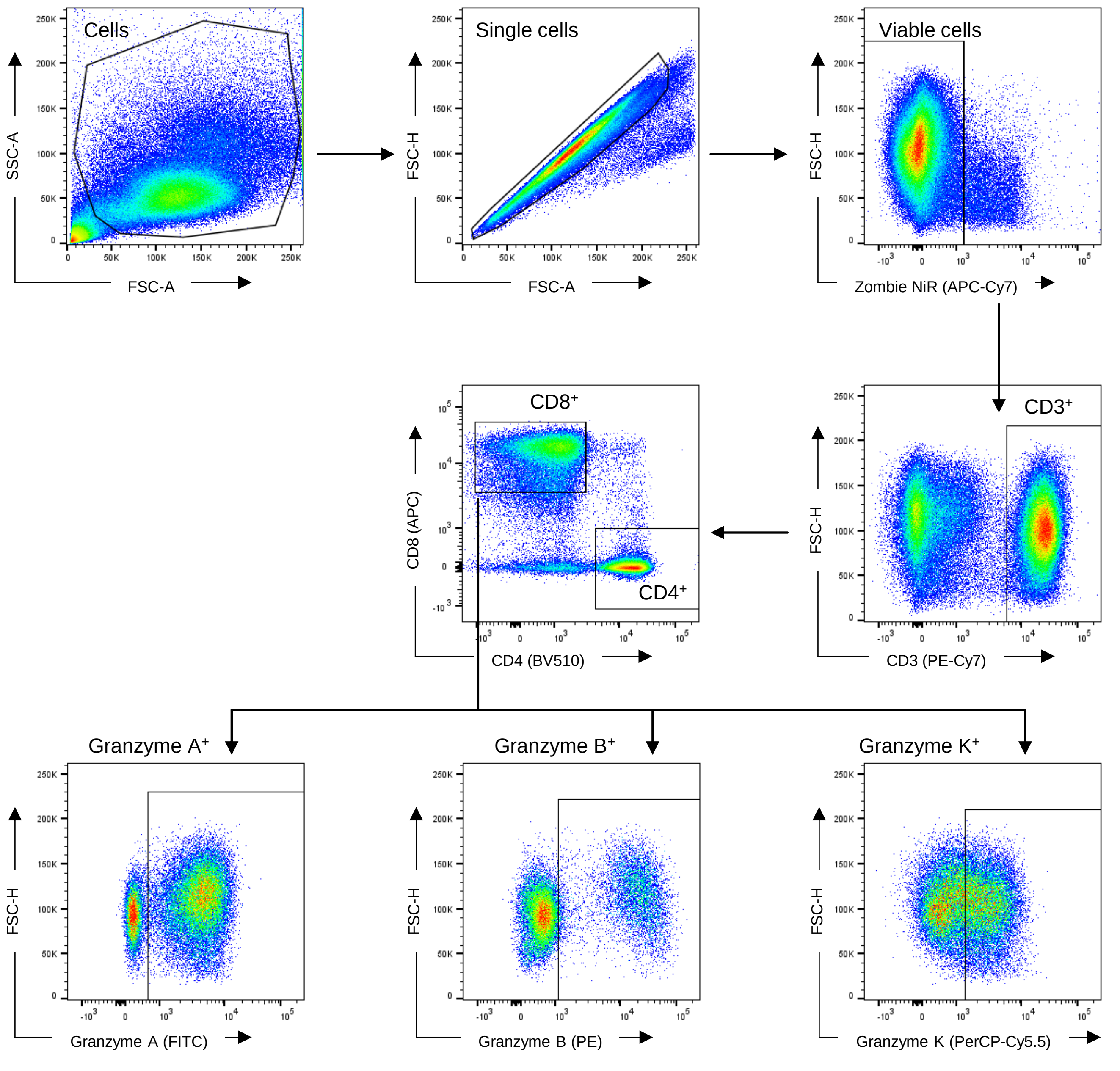

Suppl. Figure 10: Gating strategy for the analysis of granzyme expression in T cells
Gating strategy for the analysis of granzyme A, granzyme B and granzyme K expression in $CD8^{+}$ T cells in the peripheral blood of an irAE patient before ECP-therapy. Gates were set using FMO controls. Gating strategy corresponds to data presented in Figure 4 F-I and Suppl. Figure 3 K-R.

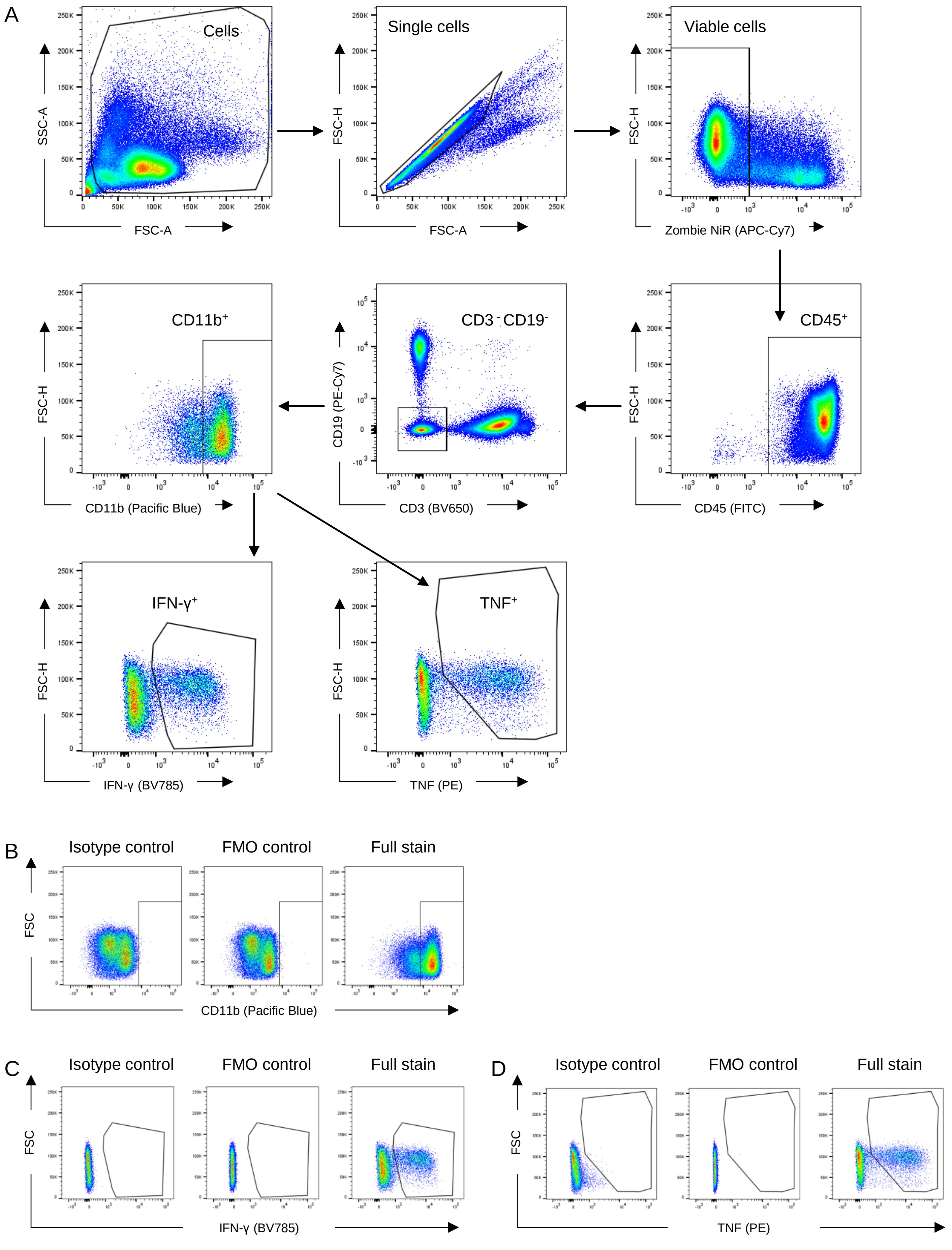

Suppl. Figure 11: Gating strategy for the analysis of cytokines produced by myeloid cells
A) Gating strategy for the analysis of IFN-γ and TNF production by CD11b⁺ myeloid cells in the peripheral blood of an irAE patient before ECP-therapy. The gate for myeloid cells was set on CD3⁻CD19⁻ cells to exclude lymphocyte contamination. **B-D)** Gates were set using FMO and isotype controls. Gating strategy corresponds to data presented in Figure 4 S-V and Suppl. Figure 4 V-Y.

Suppl. Table 1. Demographic and clinical characteristics of patients.

Variable	Value
**Total number of patients**	30
**Age**	**[years]**
- median	59.5
- range	19 - 79
**Sex**	**[absolute number (%)]**
- female	12 (40.0)
- male	18 (60.0)
**Malignant disease**	**[absolute number (%)]**
- melanoma of the skin	19 (63.3)
- uveal melanoma	1 (3.3)
- NSCLC	3 (10.0)
- SCLC	2 (6.7)
- pleural mesothelioma	1 (3.3)
- thyroid carcinoma	1 (3.3)
- Merkel cell carcinoma	1 (3.3)
- Adenocarcinoma of the breast	1 (3.3)
- cancer of unknown primary	1 (3.3)
**Primary immune checkpoint inhibitor treatment**	**[absolute number (%)]**
- double immune checkpoint inhibitor therapy (ipilimumab/nivolumab)	12 (40.0)
- monotherapy (pembrolizumab or nivolumab) followed by double immune checkpoint inhibitor therapy	5 (16.7)
- sequentially nivolumab and ipilimumab	1 (3.3)
- nivolumab	3 (10.0)
- pembrolizumab	6 (20.0)
- durvalumab/chemotherapy followed by durvalumab monotherapy	1 (3.3)
- atezolizumab/chemotherapy followed by atezolizumab monotherapy	1 (3.3)
- nivolumab/chemotherapy followed by nivolumab monotherapy	1 (3.3)
**irAE type at start of ECP (some pts had multiple irAE)**	**[absolute number (%)]**
Colitis	14 (41.2)
Hepatitis	17 (50.0)
Dermatitis	3 (8.8)
**irAE severity at ECP start**	**[absolute number (%)]**
Colitis (clinical, CTC-AE)	
G1	1 (7.1)
G2	7 (50.0)
G3	3 (21.4)
G4	3 (21.4)
	**Range**: 1-4
	**Median**: 2.0
Hepatitis	
G1	3 (17.6)
G2	3 (17.6)

G3<br>G4<br><br><br><br>Dermatitis<br>G1<br>G2<br>G3<br>G4	9 (52.9)<br>2 (11.8)<br>**Range**: 1-4<br>**Median**: 3<br><br><br>0 (0)<br>0 (0)<br>2 (66.7)<br>1 (33.3)<br>**Range**: 3-4<br>**Median**: 3
**Immunosuppressive therapy prior to ECP**<br>Corticosteroid<br>Mycophenolate mofetil<br>Infliximab	**[absolute number (%)]**<br>30 (100.0)<br>3 (10.0)<br>1 (3.3)

Suppl. Table 2. Overall frequency of AEs and SAEs.

**Type of adverse events:** iron deficiency, transferrin saturation deficiency, folic acid deficiency, anemia, fatigue, CRP increase, LDH increase, dry eyes, disorientation, itching, acneiform exanthema, exanthema, dermatitis DD BRAF/MEKi associated, dermatitis, hypopigmentation/vitiligo, hypopigmentation, weight loss, photosensitivity, rash, GGT increase, CK increase, increased body temperature, recurrent creatinine increase, hemorrhagic shock, hemorrhagic colitis, SARS-CoV2 infection, nausea, UTI, BSI, sepsis, tetraparesis, cholangitis, cholelithiasis, cholestasis due to cholelithiasis, cholestasis without cholelithiasis, gangrenous cholecystitis, rhinorrhea, throat complains, urge to urinate, thrombosis, sinusitis, systolic murmur, otitis, round lymph node suspected breast carcinoma recurrency, prostate cancer, respiratory tract infection, bronchitis, blepharitis, GIT infection, face neck edema, sleep disturbance, surface wetting disorder of both eyes, cough, icterus, hemorrhoids, lower leg edema, bacterial superinfection, oral candidiasis, conjunctivitis, loss of appetite, lower back pain, folliculitis, food drop paresis, polyneuropathy, abdominal pain, streptococcal infection, soft tissue infection, tiredness, seizure and hemiparesis due to known brain metastases and perifocal edema, headache.	
**Variable**	**Value**
**Number of events**	**[absolute number (%)]**
Adverse events	105 (100.0)
Non-serious adverse events	95 (90.5)
Serious adverse events	10 (9.5)
**CTC-AE grade**	**[absolute number (%)]**
I	56 (53.3)
II	27 (25.7)
III	18 (17.1)
IV	4 (3.8)
**Number of events with (no)relation to ECP**	**[absolute number (%)]**
Relation to ECP	6 (5.7)
No relation to ECP	99 (94.3)
**Outcome of AE**	**[absolute number (%)]**
Ongoing	29 (27.6)
Resolved	75 (71.4)
Unknown	1 (1.0)

105 AEs were documented, 6 AEs were in possible causal relationship with ECP. ECP-related AEs included hypopigmentation (1/6), exanthema (1/6), photosensitivity (3/6), and anemia (1/6).

Suppl. Table 3. Detailed frequency of individual AEs and SAEs.

AE	No. of events	CTC-AE grade (no. of events)	Relation to ECP likely	SAE	ongoing / resolved (no. of events)
folic acid deficiency	5	I (3), II (2)	no	no	resolved (3)<br>ongoing (1)<br>unknown (1)
iron deficiency	7	I (1), II (6)	no	no	resolved (4)<br>ongoing (3)
anemia	6	I (5), II (1)	no	no	resolved (4)<br>ongoing (3)
anemia	1	I (1)	yes	no	ongoing (1)
transferrin saturation deficiency	1	I (1)	no	no	ongoing (1)
respiratory tract infection	2	I (1), II (1)	no	no	resolved (2)
round lymph node suspected of breast cancer recurrency	1	II (1)	no	no	ongoing (1)
systolic murmur	1	I (1)	no	no	ongoing (1)
sinusitis	2	I (2)	no	no	resolved (2)
bacterial superinfection	1	I (1)	no	no	resolved (1)
photosensitivity	3	I (3)	yes	no	resolved (2)<br>ongoing (1)
otitis	1	I (1)	no	no	resolved (1)
lower leg edema	3	I (3)	no	no	resolved (2)<br>ongoing (1)
fatigue	3	I (3)	no	no	resolved (3)
prostate cancer	1	III (1)	no	no	ongoing (1)
oral candidiasis	2	I (2)	no	no	resolved (2)
conjunctivitis	1	II (1)	no	no	resolved (1)
cholelithiasis	1	III (1)	no	no	resolved (1)
cholestasis due to cholelithiasis	1	III (1)	no	yes	resolved (1)
cholestasis without cholelithiasis	1	III (1)	no	yes	resolved (1)
cholangitis	1	III (1)	no	no	resolved (1)
gangrenous cholecystitis	1	IV (1)	no	yes	resolved (1)
icterus	3	I (1), III (2)	no	no	resolved (1)<br>ongoing (2)
loss of appetite	1	I (1)	no	no	resolved (1)

meteorism	1	II (1)	no	no	ongoing (1)
food trop paresis	1	II (1)	no	no	ongoing (1)
polyneuropathy	1	II (1)	no	no	ongoing (1)
recurrent creatinine increase	1	IV (1)	no	no	ongoing (1)
hyperkaliemia	1	III (1)	no	no	resolved (1)
hemorrhagic shock	1	III (1)	no	yes	resolved (1)
hemorrhagic colitis	1	III (1)	no	yes	resolved (1)
sepsis	1	IV (1)	no	yes	resolved (1)
tetraparesis	1	IV (1)	no	no	ongoing (1)
streptococcal infection	1	I (1)	no	no	resolved (1)
soft tissue infection of the right calf	1	II (1)	no	no	ongoing (1)
creatinine kinase increase	1	IV (1)	no	no	resolved (1)
gamma-glutamyltransferase increase	1	III (1)	no	no	resolved (1)
itching	2	I (1), II (1)	no	no	resolved (1) ongoing (1)
LDH increase	1	I (1)	no	no	resolved (1)
seizure due to known brain metastases and perifocal edema	1	III (1)	no	yes	resolved (1)
hemiparesis due to known brain metastases and perifocal edema	1	III (1)	no	yes	resolved (1)
headache	1	III (1)	no	no	ongoing (1)
CRP increase	4	I (2), II (1), III (1)	no	no	resolved (4)
akneiform exanthema	1	II (1)	no	no	resolved (1)
exanthema	1	I (1)	yes	no	ongoing (1)
dermatitis DD BRAF/MEKi associated	1	II (1)	no	no	resolved (1)
dermatitis	1	III (1)	no	no	ongoing (1)
hypopigmentation/vitiligo	1	I (1)	no	no	ongoing (1)
hypopigmentation	1	I (1)	yes	no	ongoing (1)
rash	1	I (1)	no	no	resolved (1)

increased body temperature	1	I (1)	no	no	resolved (1)
dry eye	1	II (1)	no	no	resolved (1)
gastrointestinal infection	1	III (1)	no	yes	resolved (1)
bronchitis	1	I (1)	no	no	resolved (1)
thrombosis	1	II (1)	no	no	resolved (1)
disorientation	1	II (1)	no	no	resolved (1)
weight loss	1	I (1)	no	no	resolved (1)
SARS-CoV2 infection	1	II (1)	no	yes	resolved (1)
nausea	1	II (1)	no	no	resolved (1)
blood stream infection	1	II (1)	no	no	resolved (1)
urinary tract infection	1	II (1)	no	no	resolved (1)
rhinorrhea	1	I (1)	no	no	resolved (1)
throat complaints	1	I (1)	no	no	resolved (1)
blepharitis	1	I (1)	no	no	resolved (1)
urge to urinate	1	I (1)	no	no	resolved (1)
cough	1	I (1)	no	no	resolved (1)
face neck edema	1	II (1)	no	no	resolved (1)
sleep disturbance	1	I (1)	no	no	ongoing (1)
surface wetting disorder of both eyes	1	I (1)	no	no	ongoing (1)
hemorrhoids	1	I (1)	no	no	ongoing (1)
lower back pain	1	I (1)	no	no	resolved (1)
folliculitis	1	I (1)	no	no	resolved (1)
abdominal pain	1	I (1)	no	no	resolved (1)
increased white blood cells	1	I (1)	no	no	resolved (1)
tiredness	1	I (1)	no	no	resolved (1)

Supplementary Table 4. Assessment of colitis patients.

Colitis (clinical, CTC-AE)	Number of patients / all colitis patients	Number of patients with a biopsy **before** ECP (%)	Number of patients with a biopsy **before and after** ECP (%)	Histological grading provided **before and after** ECP (%)
G1	1	1/1 (100.0%)	1/1 (100.0%)	1/1 (100.0%)
G2	7	6/7 (85.7%)	4/7 (57.1%)	4/7 (57.1%)
G3	3	2/3 (66.7%)	2/3 (66.7%)	2/3 (66.7%)
G4	3	2/3 (66.7%)	2/3 (66.7%)	1/3 (33.3%)

Supplementary Table 5. Demographic data of healthy donors for PBMC and serum analysis.

Variable	Value
**Total number of Healthy Donors**	11
**Age**<br>- median<br>- range	**[years]**<br>31.3<br>24 - 42
**Sex**<br>- female<br>- male	**[absolute number (%)]**<br>8 (72.7)<br>3 (27.3)

Suppl. Table 6. Antibodies used for FACS analysis of immune cells in patient and healthy control derived peripheral blood.

Antigen	Fluoro-chrome	Isotype	Clone	Dilution	Vendor	Identifier
CD3	PE-Cy7	Mouse BALB/c IgG1, κ	SK7	1:10	BD Biosciences	Cat #341111; RRID:AB_2868767
CD4	FITC	Mouse BALB/c IgG1, κ	SK3	1:10	BD Biosciences	Cat #345768; RRID:AB_2868797
CD8	APC	Mouse BALB/c IgG1, κ	SK1	1:10	BD Biosciences	Cat #345775; RRID:AB_2868803
CD16	PE	Mouse BALB/c IgG1, κ	B73.1	1:10	BD Biosciences	Cat #332779; RRID:AB_2868628
CD19	FITC	Mouse IgG1, κ	HIB19	1:10	BD Biosciences	Cat #555412; RRID:AB_395812
CD56	PE	Mouse BALB/c X C57BL/6 IgG1, κ	MY31	1:10	BD Biosciences	Cat #345810; RRID:AB_286883
CD3	FITC	Mouse IgG1, κ	SK7	1:10	BD Biosciences	Cat #345763; RRID:AB_2811220
CD4	PE-Cy7	Mouse IgG1, κ	SK3	1:10	BD Biosciences	Cat #348809; RRID:AB_2783789
CD8	APC-Cy7	Mouse IgG1, κ	SK1	1:10	BD Biosciences	Cat #348813; RRID:AB_2868857
CD16	PE	Mouse IgG1, κ	B73.1	1:10	BD Biosciences	Cat #332779; RRID:AB_2868628
CD19	APC	Mouse IgG1, κ	SJ25C 1	1:10	BD Biosciences	Cat #555415; RRID:AB_398597
CD45	PerCP-Cy5.5	Mouse IgG1, κ	2D1	1:10	BD Biosciences	Cat #340953, AB_400194
CD56	PE	Mouse IgG2b, κ	NCAM 16.2	1:10	BD Biosciences	Cat #345812; RRID:AB_2629216
CD3	BV650	Mouse IgG1, κ	UCHT 1	1:25	BioLegend	Cat #300468; RRID:AB_2629574
CD3	PE/Cy7	Mouse IgG1, κ	SK7	1:25	BioLegend	Cat #344816; RRID:AB_10640737
CD4	BV510	Mouse IgG1, κ	SK3	1:25	BioLegend	Cat #344634; RRID:AB_2566017
CD4	PerCP/Cy 5.5	Mouse IgG2b, κ	OKT4	1:25	BioLegend	Cat# 317428; RRID:AB_1186122
CD8a	AF488	Mouse IgG1, κ	HIT8a	1:25	BioLegend	Cat# 300916; RRID:AB_756152

CD8a	APC	Mouse IgG1, κ	HIT8a	1:25	BioLegend	Cat #300912; RRID:AB_314116
CD8	Pacific Blue	Mouse IgG1, κ	SK1	1:25	BioLegend	Cat #344718; RRID:AB_10551438
CD11b	Pacific Blue	Rat IgG2b, κ	M1/70	1:25	BioLegend	Cat #101224; RRID:AB_755986
CD19	PE-Cy7	Mouse IgG1, κ	HIB19	1:25	BioLegend	Cat #302216; RRID:AB_314246
CD38	BV785	Mouse IgG1, κ	HIT2	1:25	BioLegend	Cat #303529; RRID:AB_2565893
CD45	FITC	Mouse IgG1, κ	HI30	1:25	BioLegend	Cat #304006; RRID:AB_314394
Granzyme A	FITC	Mouse IgG1, κ	CB9	1:25	BioLegend	Cat #507204; RRID:AB_315470
Granzyme B	PE	Rat IgG1, κ	QA18A28	1:25	BioLegend	Cat #396406; RRID:AB_2801075
Granzyme K	PerCP/Cy5.5	Mouse IgG1, κ	GM26E7	1:25	BioLegend	Cat #370514; RRID:AB_2632852
HLA-DR	BV711	Mouse IgG2a, κ	L243	1:25	BioLegend	Cat #307644; RRID:AB_2562913
IFN-γ	BV510	Mouse IgG1, κ	4S.B3	1:25	BioLegend	Cat #502544; RRID:AB_2563883
IFN-γ	BV785	Mouse IgG1, κ	4S.B3	1:25	BioLegend	Cat #502542; RRID:AB_2563882
Ki67	BV605	Mouse IgG1, κ	Ki-67	1:25	BioLegend	Cat #350522; RRID:AB_2563863
T-bet	APC	Mouse IgG1, κ	4B10	1:25	BioLegend	Cat# 644814; RRID:AB_10901173
TNF	PE	Mouse IgG1, κ	MAb11	1:25	BioLegend	Cat #502909; RRID:AB_315261
Isotype	Pacific Blue	Rat IgG2b, κ	RTK4530	1:25	BioLegend	Cat #400627; RRID:AB_493561
Isotype	PE	Mouse IgG1, κ	MOPC-21	1:25	BioLegend	Cat #400112; RRID:AB_2847829
Isotype	BV785	Mouse IgG1, κ	MOPC-21	1:25	BioLegend	Cat #400169; RRID:AB_11219601

Suppl. Table 7: Oligonucleotides used for gene expression analysis in intestinal biopsies of colitis patients.

Gene	forward	reverse
hADIPOQ	5'-TATGATGGCTCCACTGGTA-3'	5'-GAGCATAGCCTTGTCCTTCT-3'
hCXCL9	5'-CTGTTCCTGCATCAGCACCAAC-3'	5'-TGAACTCCATTCTTCAGTGTAGCA-3'
hCXCL10	5'-GGTGAGAAGAGATGTCTGAATCC-3'	5'-GTCCATCCTTGGAAGCACTGCA-3'
hGZMA	5'-CCACACGCGAAGGTGACCTTAA-3'	5'-CCTGCAACTTGGCACATGGTTC-3'
hGZMB	5'-CGACAGTACCATTGAGTTGTGCG-3'	5'-TTCGTCCATAGGAGACAATGCCC-3'
hIFNG	5'-GAGTGTGGAGACCATCAAGGAAG-3'	5'-TGCTTTGCGTTGGACATTCAAGTC-3'
hLCN2	5'-GTGAGCACCAACTACAACCAGC-3'	5'-GTTCCGAAGTCAGCTCCTTGGT-3'
hTBP	5'-GCCCGAAACGCCGAATAT-3'	5'-CCGTGGTTCGTGGCTCTCT-3'
hTBX21	5'-ATTGCCGTGACTGCCTACCAGA-3	5'-GGAATTGACAGTTGGGTCCAGG-3

Clinical Trial Protocol

Phase 1/2 Trial to determine the safety and efficacy of Extracorporeal photopheresis as treatment for steroid-refractory immune related adverse events after immune checkpoint inhibitor therapy

**Name of the study:**	ECIR (**EC**P for **ir**AE)
**EudraCT-No.:**	**2021-002073-26**
**DRKS No.:**	DRKS00025078
**Study code:**	**1125-ECIR**
**Version und Date:**	FINAL 2.0 23.04.2023
**Sponsor:**	Medical Center - University of Freiburg<br>*- represented by the Chief Medical Officer and the Chief Financial Officer-*<br>Breisacher Str. 153, 79110 Freiburg, Germany
**Coordinating Investigator:**	Prof. Dr. med. Robert Zeiser<br>Medical Center - University of Freiburg<br>Albert-Ludwigs-University Freiburg<br>Department of Medicine I<br>Specialties: Hematology, Oncology, and Stem-Cell Transplantation<br>Hugstetter Str. 55, 79106 Freiburg, Germany

This protocol contains confidential information and is intended solely for the guidance of the clinical investigation. This protocol may not be disclosed to parties not associated with the clinical investigation or used for any purpose without the prior written consent of the sponsor.

Signature

Sponsor (Representative of the Sponsor) — Date

delegated to Prof. Dr. med. Robert Zeiser

Prof. Dr. R. Zeiser — 28.11.2023

Prof. Dr. med. Robert Zeiser — Date

Coordinating Investigator

„Leiter der klinischen Prüfung“ (LKP)

according to Arzneimittelgesetz (AMG)

Gabriele Ihorst — 5.12.2023

Dr. Gabriele Ihorst — Date

Statistician

Declaration of Investigator

**Protocol Short Title:**	ECIR (**EC**P for **ir**AE)
**EudraCT No.:**	2021-002073-26
**Protocol Version No:**	V2.0 23.04.2023
**Trial Center:**	<Site>
**Investigator:**	<Investigator>

I confirm that I have read the Clinical Trial Protocol (CTP) and hereby commit to adhering to all actions and terms as specified in the relevant sections of the clinical, ethical and general paragraphs.

I confirm that I and my colleagues will comply with the local legislation (in Germany, the German Drug Law with the appropriate amendments). I further confirm that the clinical trial will be carried out in compliance with the Declaration of Helsinki and ICH-GCP guidelines.

I acknowledge that all confidential information contained in this document will not be used for any other purpose other than the evaluation or conduct of the clinical investigation without the prior written consent of the Sponsor.

Under my supervision I put copies of this CTP and possible updates as well as access to all information regarding the carrying out of this clinical trial at the disposal of my colleagues; in particular I will promptly forward all information from the Sponsor in relation to pharmaceutical safety (SUSARs and SmPC updates) to my colleagues.

I confirm that I and my colleagues were informed by a responsible scientist about the results and expected risks of the pharmacological and toxicological examination associated with the clinical trial.

I will discuss this CTP in detail with my colleagues and ensure that they are comprehensively informed about the trial compound/preparation and the execution of the trial.

I confirm that I will be responsible for supervising any individual or party to whom I delegate study tasks conducted at the trial site.

Furthermore I commit myself not to commence patient enrolment prior to approval of the competent authorities (CA) and acceptance by the responsible Independent Ethics Committee (IEC).

Investigator of the site Date

Responsibilities and addresses:

**Sponsor**	**Medical Center - University of Freiburg**<br>- represented by the Chief Medical Officer and the Chief Financial Officer-<br>Breisacher Str. 153, 79110 Freiburg, Germany
**Coordinating Investigator**	**Prof. Dr. med. Robert Zeiser**<br>Medical Center - University of Freiburg<br>Albert-Ludwigs-University Freiburg<br>Department of Medicine I<br>Specialties: Hematology, Oncology, and Stem-Cell Transplantation<br>Hugstetter Str. 55, 79106 Freiburg, Germany<br>Phone: +49 761 270-34580<br>Fax: +49 761 270-73570<br>e-mail: robert.zeiser@uniklinik-freiburg.de
**Protocol development**	**Prof. Dr. med. Robert Zeiser**<br>Medical Center - University of Freiburg<br>Albert-Ludwigs-University Freiburg<br>Department of Medicine I<br>Specialties: Hematology, Oncology, and Stem-Cell Transplantation<br>Hugstetter Str. 55, 79106 Freiburg, Germany<br>Phone: +49 761 270-34580<br>Fax: +49 761 270-73570<br>e-mail: robert.zeiser@uniklinik-freiburg.de
	**Dr. med. Petya Apostolova**<br>Medical Center - University of Freiburg<br>Albert-Ludwigs-University Freiburg<br>Department of Medicine I<br>Specialties: Hematology, Oncology, and Stem-Cell Transplantation<br>Hugstetter Str. 55, 79106 Freiburg, Germany<br>Phone: +49 761 270-72080<br>e-mail: petya.apostolova@uniklinik-freiburg.de<br><br>**PD Dr. Frank Meiß**<br>Leitender Oberarzt<br>Department of Dermatology Medical Center - University of Freiburg<br>Albert-Ludwigs-University Freiburg<br>e-mail: frank.meiss@uniklinik-freiburg.de

	**Dr. David Rafei-Shamsabadi**<br>Department of Dermatology Medical Center -<br>University of Freiburg<br>Albert-Ludwigs-University Freiburg<br>e-mail: david.rafei-shamsabadi@uniklinik-freiburg.de<br><br>**Dr. med. Enrico De Toni**<br>Medizinische Klinik und Poliklinik II<br>University of Munich (LMU)<br>Marchioninstr. 15<br>81377 München<br>+49 (0)89 4400-78829<br>e-mail: Enrico.DeToni@med.uni-muenchen.de<br><br>**Dr. med. Carolin Funke-Lorenz**<br>Klinik und Poliklinik für Dermatologie, Venerologie und Allergologie<br>Universitätsklinikum Essen<br>Hufelandstraße 55<br>45147 Essen<br>Tel. 0201 723-4939<br>Fax. 0201 723-3174<br>e-mail: carolin.funke-lorenz@uk-essen.de<br><br>**Dr. med. Jan-Malte Placke**<br>Klinik und Poliklinik für Dermatologie, Venerologie und Allergologie<br>Universitätsklinikum Essen<br>Hufelandstraße 55<br>45147 Essen<br>Tel. 0201 723-85718<br>e-mail: Jan-Malte.Placke@uk-essen.de<br><br>**PD Dr. Sebastian Haferkamp**<br>Universitätsklinikum Regensburg<br>Klinik und Poliklinik für Dermatologie<br>Franz-Josef-Strauss-Allee 11<br>93053 Regensburg<br>Tel: 0941/944 9603.<br>Fax: 0941/944 9541<br>e-mail: sekretariat.derma@klinik.uni-regensburg.de
**Special laboratory**	**Dr. rer. nat Wolfgang Melchinger**<br>ZTZ Zentrum für Translationale Zellforschung<br>3. OG / Labor Prof. Zeiser<br>Breisacher Str. 115, 79106 Freiburg, Germany<br>Phone: +49 761 270-71817<br>Fax: +49 761 270-35030

**Statistician**	**Dr. rer. nat. Gabriele Ihorst**<br>Medical Center - University of Freiburg<br>Clinical Trials Unit (Zentrum Klinische Studien)<br>Elsaesser Str. 2, 79110 Freiburg, Germany<br>Phone: +49 761 270-73750<br>Fax: +49 761 270-73730<br>e-mail: gabriele.ihorst@uniklinik-freiburg.de
**Pharmacovigilance**<br>**SAE-Management**	Medical Center - University of Freiburg<br>Clinical Trials Unit (Zentrum Klinische Studien)<br>Elsaesser Str. 2, 79110 Freiburg, Germany<br>Fax: +49 761 270-74390<br>e-mail: zks.pv@list.uniklinik-freiburg.de
**Data management / Registration**	Medical Center - University of Freiburg<br>Clinical Trials Unit (Zentrum Klinische Studien)<br>Elsaesser Str. 2, 79110 Freiburg, Germany<br>Phone: +49 761 270-77102<br>Fax: +49 761 270-74390
**Project Management**	**Dr. Matthias Weiß**<br>Early Clinical Trials Unit<br>Department of Medicine I<br>Medical Center – University of Freiburg<br>Hugstetter Str. 55, 79106 Freiburg, Germany<br>Phone: +49 761 270-32902<br>E-Mail: matthias.weiss@uniklinik-freiburg.de
**Monitoring**	Medical Center - University of Freiburg<br>Department of Medicine I<br>Specialties: Hematology, Oncology, and Stem-Cell Transplantation<br>Hugstetter Str. 55, 79106 Freiburg, Germany<br>Phone: +49 761 270-36710
**Data Monitoring Committee (DMC)**	Prof. Dr. Lutz Müller (Universitätsklinikum Jena)<br>Prof. Dr. Alexandros Spyridonidis (Universitätsklinikum Patras, Griechenland)<br>Prof. Dr. Evelyn Ullrich (Universitätsklinikum Frankfurt)

Amendment Protocol Version 2.0

Reason for this substantial Amendment:

1. Based on the results of the interim analysis, the number of patients to be recruited was increased to 30.

Summary of changes to the trial protocol (CTP):

1. Adjustment of the study design according to patient number and time schedule, section 2.
2. Adjustment of inclusion criteria and the irAE grading according to CTCAE V5.0 in synopsis, section 5.3 and Appendix 1.
3. Information about voluntary participation in a post-trial-interview described in section 7.6 and flow chart.
4. EDTA and serum samples for scientific analysis were collected exclusively in Freiburg, section 7.7.12
5. Addition of an assessment of quality of life as key secondary endpoint in synopsis, in flow chart, in section 7.7.14 and in appendices 2 and 3
6. Adjustment of SAE documentation in section 10.2 and SAE exceptions in section 10.5.
7. Correction of typos.
8. Update of internet links in Appendix 2
9. Change of participating sites from 4 to 3

List of abbreviations

AE	Adverse Event
ALT	Alanine aminotransferase
AMG	Arzneimittelgesetz (“German Drug Law”)
AST	Aspartate aminotransferase
BW	Body weight
CA	Competent authority
CI	Confidence interval
CONSORT	Consolidated Standards of Reporting Trials
CR	Complete Remission
CRA	Clinical Research Associate
CRF	Case Report Form
CTCAE	Common Terminology Criteria for Adverse Events
CTP	Clinical Trial Protocol
CTU	Clinical Trials Unit
CYP	Cytochrome P450
DCR	Disease control rate
DMC	Data Monitoring Committee
DMM	Data Management Manual
DSUR	Development safety update report
ECOG	Eastern Cooperative Oncology Group
ECP	Extracorporeal photopheresis
EORTC	European Organization for Research and Treatment of Cancer
EU	European Union
FAS	Full analysis set
GCP	Good Clinical Practice
GvHD	Graft-versus-Host-Disease
HIV	Human Immunodeficiency Virus
IB	Investigator’s Brochure
ICB	Immune checkpoint blockade
ICF	Informed Consent Form
ICH	International Conference on Harmonization
IEC	Independent Ethics Committee
IMP	Investigational Medicinal Product
IRAE	Immune-related adverse event
ISF	Investigator Site File
ITT	Intention-to-treat
IV	Intravenous/ly
LKP	Leiter der Klinischen Prüfung (Coordinating Investigator)
MedDRA	Medical dictionary for regulatory activities
MNK	Mallinckrodt Pharmaceuticals
NIH	National Institutes of Health
NRM	Non-relapse mortality
NYHA	New York Heart Association
ORR	Objective response rate
OS	Overall survival

PD	Progressive disease
PFS	Progression-free survival
PP	Per protocol
PR	Partial Remission
PT	Preferred term
PTI	Post-trial-interview
QLQ	Quality of Life Questionnaire
QLQ-HDC29	high-dose chemotherapy questionnaire module to supplement QLQ-C30
QM	Quality Management
SAE	Serious Adverse Events
SAP	Statistical analysis plan
SAS	Statistical analysis system
SD	Stable disease
SDV	Source data verification
SmPC	Summary of product characterization
SOC	System Organ Class
SOP	Standard Operating Procedure
SUSAR	Suspected Unexpected Serious Adverse Reaction
TMF	Trial Master File
ULN	Upper limit of normal
WHO	World Health Organization

Table of contents

Declaration of Investigator 3
Responsibilities and addresses: 4
List of abbreviations 8
List of Tables 13
List of Figures 13
Synopsis 14
1 Introduction and rationale for the study 18
2 References 21
3 Overall risk benefit assessment 23
3.1 Covid 19 – Risk-Benefit Assessment 23
4 Study Design 24
4.1 Number of patients 24
4.2 Participating sites 24
4.3 Time Schedule 24
5 Study Population and patient eligibility 24
5.1 Target population 24
5.2 Gender distribution 24
5.3 Inclusion Criteria 25
5.4 Exclusion Criteria 26
6 Objectives and endpoints 26
6.1 Overview objectives and endpoints 27
6.2 Response definitions related to irAE 28
6.2.1 Complete Remission (CR) 28
6.2.2 Partial Remission (PR) 28
6.2.3 Progressive disease (PD) 28
6.2.1 Stable disease (SD) 28
6.2.2 Overall response rate (ORR) 28
7 Trial Conduct 28
7.1 Patient eligibility 28
7.2 Study treatment 29
7.3 Treatment with investigational procedure including the investigational medicinal product (methoxsalen) 29
7.3.1 ECP, duration of treatment 29
7.3.2 ECP, procedure recommendations 30
7.3.3 ECP, combination with corticosteroids 30
7.3.4 ECP, combination with other drugs 30
7.4 Concomitant medication 30
7.5 Treatment after end of trial 30
7.6 Post-trial-interviews (PTI) 30
7.7 Visit schedule and assessments 31
7.7.1 Flow chart and visit schedule 31
7.7.2 Screening and registration 35

7.7.3 Data to be collected on screening failures 35
7.7.4 Assessments and specifications 36
7.7.5 Informed consent, Inclusion-/Exclusion criteria 36
7.7.6 Patient demographics 36
7.7.7 Medical history 36
7.7.8 Current disease status 36
7.7.9 Physical examination 36
7.7.10 irAE grading 36
7.7.11 Laboratory tests 37
7.7.12 EDTA and serum samples for scientific analysis (Freiburg only) 37
7.7.13 Assessment of response 37
7.7.14 Assessment of quality of life 37
7.7.15 Concomitant medication 38
7.7.16 Adverse events 38
7.7.17 Dermatological examination 38
7.7.18 Ophthalmological examination 38
8 Discontinuation criteria 38
8.1 Premature termination of the entire trial 38
8.2 Premature termination of the trial at one of the trial sites 39
8.3 Discontinuation of trial treatment or trial participation for individual patients 39
8.3.1 Premature termination of trial treatment 39
8.3.2 Premature termination of trial participation 39
9 Investigational Procedure including Investigational Medicinal Product (IMP) 40
9.1 Description of IMP 40
9.1.1 IMP background information 40
9.1.2 Pharmacology and Pharmacokinetics 41
9.2 Summary of safety profile 41
9.2.1 Expected Adverse Events reported in clinical trials or postmarketing use 41
9.2.2 Reference Safety Information for Assessment of Expectedness of Adverse Events 42
9.3 IMPs pharmaceutical characteristics 42
9.4 Packaging and Labeling 43
9.5 Supply and ordering 43
9.6 Receipt and storage 43
9.7 Destruction of IMP 43
10 Safety 44
10.1 Definition of adverse events 44
10.2 Documentation of (S)AEs 45
10.3 Laboratory Test Result Abnormalities 45
10.4 Definition and documentation of serious adverse events (SAEs) 46
10.4.1 Definition of SAE 46
10.5 Exceptions from SAE Reporting 47
10.6 Reporting of SAEs 47
10.6.1 Investigator requirements for SAE Reporting 47
10.6.2 Sponsor requirements for SAE reporting 48
10.7 Development safety update report (DSUR) 49
10.8 Pregnancies 49
10.9 Contraception 49

10.10 Administration setting 50
11 Documentation 50
11.1 Data handling and data management 50
11.1.1 Data confidentiality 50
11.2 Documentation of trial data 51
11.2.1 Documentation in medical records 51
11.2.2 Documentation in eCRF 51
11.3 Data management 51
11.3.1 Data coding 52
11.4 Investigator Site File (ISF) 52
11.5 Source documents 52
11.6 Archiving 52
11.6.1 Sponsor's Responsibility for Archiving 52
11.6.2 Investigator's Responsibility for Archiving 53
12 Quality Assurance 53
12.1 Monitoring 53
12.2 Audits and Inspections 53
13 Biostatistical planning and analysis 54
13.1 Trial design 54
13.2 Objectives and endpoints 54
13.3 Sample size estimation 54
13.4 Definition of analysis sets 54
13.5 Statistical analysis 54
13.5.1 Primary endpoint analysis 54
13.5.2 Secondary endpoint analysis 55
13.5.3 Patient demographics/other baseline characteristics 55
13.5.4 Trial treatment 55
13.5.5 Concomitant medication 55
13.5.6 Safety analysis 55
13.5.7 Interim analysis 56
14 Data Monitoring Committee (DMC) 56
15 Reporting 57
15.1 Final report 57
15.2 Publication Policy 57
16 Ethical and regulatory aspects 57
16.1 Sponsor's and Investigator's responsibilities 57
16.2 Independent Ethics Committee and Health Authorities 58
16.3 Informed Consent 58
16.4 Data Privacy Protection and Confidentiality 59
16.5 Insurance 59
16.6 Financial aspects of the study 59
17 Protocol amendments or changes in trial conduct 59
18 Appendices 60
Appendix 1 Grading of irAE according to CTCAE V5 60
Colitis/Diarrhea 60
Appendix 2 Relevant laws, guidelines and links 62

Appendix 3 EORTC QLQ – HDC29 63

List of Tables

Table 1 Objectives and related endpoints 27
Table 2 Study procedure and Flow Chart (Screening and treatment phase) 32
Table 3 Study procedure and Flow Chart (Follow Up / End of study) 33

List of Figures

Figure 1 Clinical and histological response of a patient treated with ECP for irAEs after anti-PD1 antibody treatment 20

Synopsis

<table>
<tr><td>Study title</td><td colspan="2">Phase 1/2 Trial to determine the safety and efficacy of Extracorporeal photopheresis (ECP) as treatment for steroid-refractory immune related adverse events (irAE) after immune checkpoint inhibitor therapy</td></tr>
<tr><td>Study Code</td><td colspan="2">1125-ECIR</td></tr>
<tr><td>EudraCT-Nr.</td><td colspan="2">2021-002073-26</td></tr>
<tr><td>Acronym</td><td colspan="2">ECIR (ECP for irAE)</td></tr>
<tr><td>Medical Condition</td><td colspan="2">Immune related adverse events induced by treatment with immune checkpoint inhibitors (monoclonal antibodies against PD-1, PD-L1 or CTLA-4)</td></tr>
<tr><td>Trial type</td><td colspan="2">Open-label, multicentric, non-randomized phase 1/2 clinical trial</td></tr>
<tr><td>Objectives</td><td colspan="2">Our preliminary data demonstrate that irAEs induced by immune checkpoint blockade can be successfully treated with ECP (Apostolova et al. NEJM 2020). We therefore intend to launch a clinical phase 1/2 trial to validate this finding in a prospective trial. The primary objective is evaluation of safety of ECP treatment in patients with irAEs. As a secondary objective, we will determine the efficacy of ECP as a treatment for immune-related adverse events and its effect on tumor progression.</td></tr>
<tr><td rowspan="3">Interventions</td><td><u>Experimental intervention:</u></td><td>Treatment with ECP for irAEs with 2 cycles performed on two consecutive days, for the first 4 weeks repeated every week, and from week 5 to 12 repeated every two weeks</td></tr>
<tr><td><u>Duration of intervention per patient:</u></td><td>12 weeks (might be prolonged for up to 60 weeks if patient experiences benefit from treatment)</td></tr>
<tr><td><u>Follow-up per patient:</u></td><td>12 months after end of ECP treatment</td></tr>
<tr><td>Outcome (more details see chapter 6.1)</td><td colspan="2"><u>Primary safety endpoint</u>:
To evaluate the rate of treatment-related adverse events (AEs) and serious adverse events (SAEs) in patients treated with ECP for immune-checkpoint inhibitor-induced colitis, pneumonitis, hepatitis or dermatitis. A positive result from the study is defined as ≤50% of the patients developing a treatment-related SAE.

<u>Key secondary endpoints</u>:
• To evaluate the investigator-assessed objective response rate (ORR) to treatment after 6 and 12 weeks of ECP therapy. The response criteria for each organ are described in chapter 6.2.
• To assess time to response
• To assess the duration of investigator-assessed clinical response
• To assess overall survival (OS) at 1 year after end of ECP treatment
• To measure the time to complete discontinuation of other immunosuppressive therapy
• To assess changes in immune cell phenotype and metabolism during treatment
• To measure quality of life (EORTC QLQ-C30 and QLQ-HDC29) before and during ECP treatment
• To assess the relapse rates of irAEs upon discontinuation of ECP and rechallenge with an immune checkpoint inhibitor
• To assess the incidence of tumor progression or relapse</td></tr>
</table>

Inclusion criteria (see chapter 5.3)	1. Male and female patients aged ≥18 years with adequate German written and oral language skills<br>2. Written informed consent:<br>• Subjects must have signed and dated an IRB/IEC approved written informed consent form in accordance with regulatory and institutional guidelines.<br>• Subjects must be able to understand and willing to comply with scheduled visits, treatment schedule, laboratory tests and mandatory collection of blood, and other requirements of the study.<br>• Subject Re-enrollment: This trial permits the re-enrollment of a subject that has discontinued the study as a screening failure. If re-enrolled, the subject must be re-consented.<br>3. Target population<br>• Patients who have received treatment with an anti-PD-1, anti-PD-L1 or an anti-CTLA-4 antibody or any combination of these for any type of malignancy in the last 24 months before screening. 30% of the patients that will be included should have non-skin cancer.<br>• Patients should have clinical and/or histological evidence of immune-related adverse events as follows:<br>o Colitis<br>▪ Diarrhea with increase of ≥4 stools over baseline<br>▪ No improvement after 72h treatment with at least 1 mg/kg BW/day prednisolone equivalent<br>o Hepatitis<br>▪ Alanine aminotransferase and/or aspartate aminotransferase ≥3x ULN if baseline was normal; or ≥3x baseline if baseline was abnormal and/or total bilirubin >1.5 ULN.<br>▪ No improvement after 72h treatment with at least 1 mg/kg BW/day prednisolone equivalent<br>o Pneumonitis<br>▪ Radiographic changes that involve more than one lobe of the lung or ≥25% of lung parenchyma and new symptoms such as cough, dyspnea or new oxygen therapy<br>▪ No improvement after 72h treatment with 1 mg/kg BW/day prednisolone equivalent<br>o Dermatitis<br>▪ Rash covering >30% of the body surface area and moderate or severe symptoms<br>▪ No improvement after 72h treatment with at least 1 mg/kg BW/day prednisolone equivalent<br>4. Maximum of one additional (second line) therapy after Steroid treatment before ECP starts (e.g. infliximab for colitis)<br>5. Women of childbearing potential (WOCBP) must have a negative serum pregnancy test (minimum sensitivity 25 IU/L or equivalent units of HCG) within 1 week prior to the start of study drug. Males who are sexually active with WOCBP must use during the duration of the study and up to 5 months afterwards a latex or synthetic condom during any sexual contact with females of reproductive potential, even if they have undergone a successful vasectomy (see also 10.9). Patients must abstain from donating blood, semen, or sperm during participation in the study

	6. Women must not be breastfeeding.<br>7. ECOG performance status 0, 1, or 2
Exclusion criteria (see chapter 5.4)	1. Active treatment in a clinical study of any investigational agent within 14 days prior day 0 or within 5 half-lives of the study treatment, whichever is greater.<br>2. Positive result for HIV.<br>3. Active COVID-19-infection or non-compliance with the prevailing hygiene measures regarding the COVID-19 pandemic<br>4. Prior allogeneic bone marrow transplantation or prior solid organ transplantation.<br>5. Patients who require vasopressors, and/or have NYHA class III or IV heart failure.<br>6. Uncontrolled hypertension or ventricular arrhythmias.<br>7. Previous or concurrent malignancies within the last 3 years of enrollment other than the disease for which checkpoint-inhibitor blockade was applied. Exceptions are adequately treated basal or squamous cell skin cancer, or any other cancer from which the subject has been disease-free for more than 3 years.<br>8. Any condition that would, in the investigator's judgment, interfere with full participation in the study, including administration of study drug and attending required study visits; pose a significant risk to the subject; or interfere with interpretation of study data<br>9. Known allergies, hypersensitivity, or intolerance of methoxypsoralen, excipients, or similar compounds, acid-citrate-dextrose or similar compounds<br>10. Aphakia<br>11. Sexually active men and female patients of child-bearing potential who are not willing to use highly effective methods of contraception during the trial and at least 5 months after the last ECP procedure (see also 10.9)<br>12. Inability to tolerate extracorporeal volume loss<br>13. Previous splenectomy<br>14. Pregnancy and lactation
Statistical analysis	<u>Safety</u>:<br>Adverse events and serious adverse events will be registered and reported according to the guideline of ICH/GCP. Rates of adverse events and of serious adverse events will be calculated with corresponding 95% confidence intervals.<br><br><u>Efficacy</u><br>The efficacy analyses will be based on the full analysis set (FAS) and will be considered as descriptive results.<br><br><u>Secondary endpoints</u>:<br>Descriptive analyses of the secondary endpoints will be performed.<br>OS probabilities will be estimated and displayed using the Kaplan Meier method. The analysis of secondary time-to-event endpoints with competing risks will be performed by means of the Aalen Johanson estimator for the calculation of cumulative incidence rates.<br><br>As this is a phase 1/2 trial without the intention of a confirmatory proof of efficacy, no formal sample size calculation is performed. The determination to include 30 evaluable patients is mainly based on feasibility considerations. It is expected that

	only few SAEs will occur. If the probability of any ECP-related SAE for a patient is 20% (p=0.2), the probability to observe at least one patient with a treatment-related SAE is 0.956.	
Sample size	To be assessed for eligibility:<br>To be assigned to the trial:<br>To be analysed:	n = 50<br>n = 30<br>n = 30
Trial duration	Recruitment period:<br>First patient in to last patient out<br>Duration of the entire trial:	24 months<br>39 months<br>48 months
	Treatment duration per patient:<br><br>Follow-up duration per patient:	12 weeks or as long as the patient experiences benefit from treatment (maximum of 60 weeks)<br>12 months after end of treatment
	Interim safety analysis	After the first 14 patients have completed study treatment or reached month 3 after treatment start (whichever occurs first)
Participating centers	UKF Freiburg (Lead center), UK Essen, UK Regensburg	

1 Introduction and rationale for the study

In the last decade, immune checkpoint inhibitors (ICIs) have dramatically changed the treatment algorithms of many solid tumors and hematological malignancies. These antibodies inhibit negative regulatory molecules of the immune response, including cytotoxic T lymphocyte-associated antigen 4 (CTLA-4), programmed cell death protein-1 (PD-1) and its ligand (PD-L1), resulting in an enhanced T cell response against cancer cells [1]. However, the increased immune activation can also induce immune-related adverse events (irAEs), which can affect any organ system, most commonly the skin, gastrointestinal tract, endocrine glands, liver and lung [2]. The incidence of irAEs is high, with frequencies of up to 62% and 50% in patients treated with CTLA-4 and PD-1/PD-L1, respectively [3-5]. Particularly grade 3 and grade 4 irAEs are a major concern, since patients often have to permanently discontinue ICI therapy, receive immunosuppressive therapy including corticosteroids and are at high risk of death.

ECP is an efficient treatment for acute and chronic graft-versus-host disease (GVHD) [6,7]. We could recently show that ECP was also effective in a patient with severe immune checkpoint inhibitor-induced autoimmune colitis [8]. The 29-year old male patient was treated with ipilimumab and nivolumab for metastatic melanoma. After two doses, the patient developed dermatitis, thyroiditis, hepatitis and colitis. Colitis was diagnosed based on macroscopic mucosal ulcerations and intraepithelial apoptosis and crypt loss identified in the biopsy (**Fig. 1a,b**). While dermatitis, thyroiditis and hepatitis resolved after stopping ipilimumab/nivolumab and corticosteroid treatment, the patient experienced three colitis episodes within the next 20 weeks (CTC-AE II-III°). These were treated with corticosteroids (in total 23 weeks prior to ECP), infliximab (2 single doses 18 and 15 weeks prior to ECP) and cyclosporine (14 weeks prior to ECP, **Fig. 1a**) [8]. Since there was no durable response, the patient received ECP. During the next 8 months, he underwent 2 cycles of ECP on consecutive days every 2-4 weeks. ECP was well tolerated and led to a complete response (**Fig. 1a**). Immunosuppression was tapered without symptom rebound. Continuous remission of colitis was confirmed by colonoscopy (**Fig. 1c**). Immune checkpoint-inhibitor treatment was discontinued upon first manifestation of irAE and never resumed.

We analyzed the peripheral blood leukocyte compartment at multiple time points before and during ECP treatment. We observed a 4-fold increase in NK cells (**Fig. 1d-e**), with an immuno-regulatory phenotype (**Fig. 1f,g**). Also multiple pro-inflammatory cytokines were lower in the patient compared to age-matched healthy donors [8]. Supporting the notion that NK cells regulated autoimmunity, adoptive transfer of the patient's NK cells prevented irAE in a murine irAE model triggered by human T cells and anti-PD-1 antibody-treatment in a dose-dependent manner (**Fig. 1h**). Data regarding safety and efficacy of ECP for irAE treatment have been lacking so far. Our experience implicates that ECP can be an efficient therapy for refractory checkpoint inhibitor-associated colitis through the expansion of a protective NK cell population. An increase in NK cells during ECP for extensive chronic GVHD has been previously observed [9]. Patients with acute GVHD have a higher frequency of CD56^{bri} NK subsets with stronger NKG2D and CD62L expression[10]. In the same study, CD56^{-}CD16^{+} NK cells with higher expression of CD57 and CD11b were increased in patients with chronic cGVHD. ECP shifted the NK cell populations towards a more immuno-regulatory phenotype with protection of a specialized anti-viral and anti-leukemic CD57^{+}NKG2C^{+}CD56^{dim} subset[10]. We hypothesize that similar mechanisms might be responsible for the protective effects of NK cells against irAE. When comparing the NK cell compartment in the patient with that of age- and sex-matched healthy controls, we observed a downregulation of CD16 expression, in particular on CD56^{dim}

NK cells. CD16 is the FcRγIII, an activating NK cell receptor that can induce strong cytokine production. Previous studies show that shedding of CD16 might be an immuno-regulatory mechanism to prevent autoimmunity[11]. CD16 downregulation modulates NK cell responses and contributes to maintenance of the immune homeostasis of both antibody and T cell-dependent pathways[12]. Supporting this hypothesis, expression of GM-CSF, IFN-γ, TNF and IL-2 was lower in the NK cells of the patient when compared to the control group.
The mechanism of action of ECP is not fully understood. It has been shown that after ECP the frequency of Treg, MDSC and IL-10 producing cells is increased [13].

The current trial will investigate the safety of ECP in the treatment of a specified subgroup of common irAE (colitis, hepatitis, pneumonitis and dermatitis). These irAE were selected because of their frequency and severity. To understand if ECP has activity in different types of cancer we plan to include 30% non-skin cancer patients. Patients with immune-mediated endocrine disorders will not be enrolled because endocrinopathies often require the sole supplementation of hormones and not a causal therapy. Other, more rare irAE, are excluded in order to keep the patient population homogenous.

Figure 1 Clinical and histological response of a patient treated with ECP for irAEs after anti-PD1 antibody treatment

Figure 1

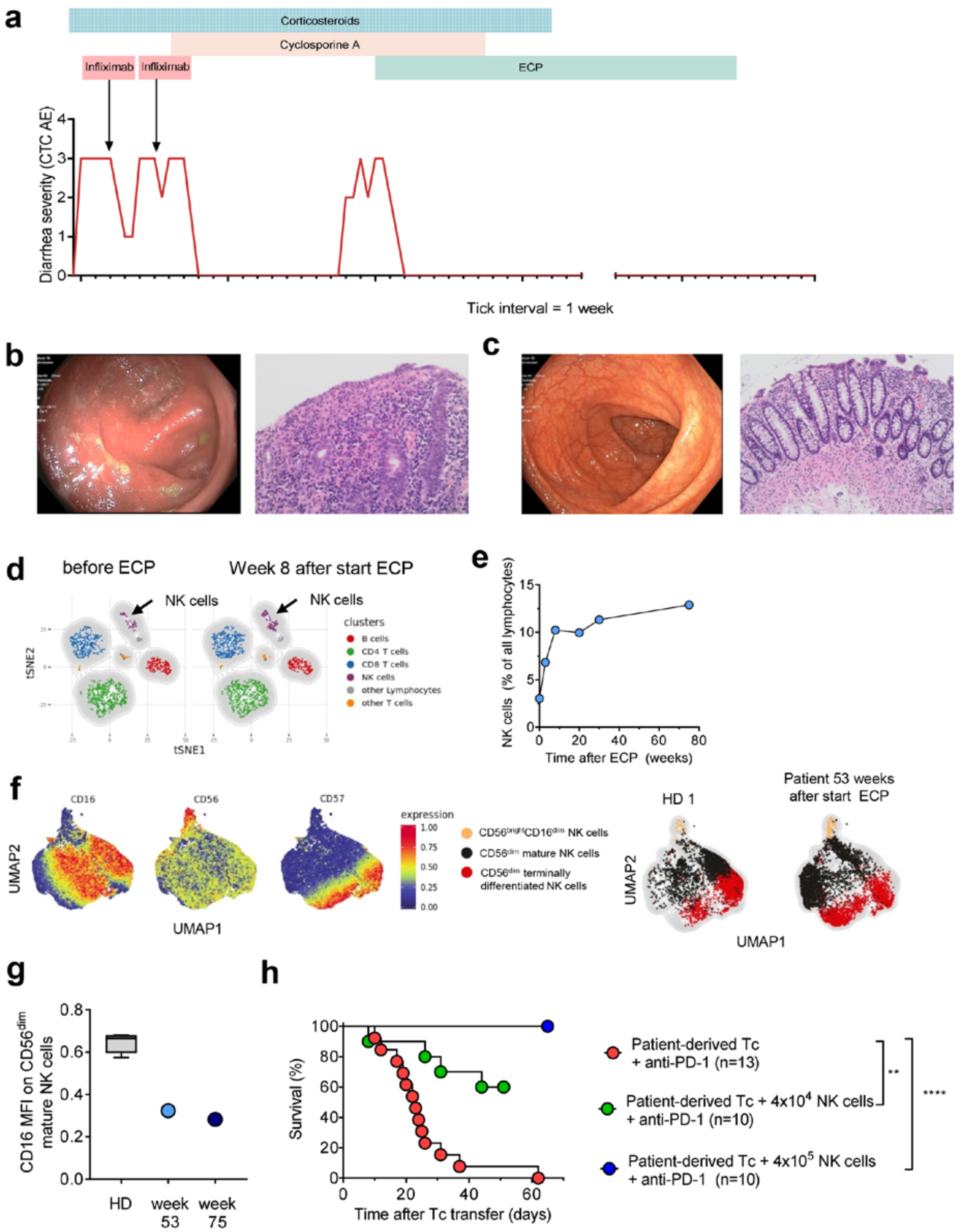

Figure 1: Refractory autoimmune colitis responding to ECP which leads to the expansion of immunoregulatory NK cells

(a) The diarrhea severity of the patient according to common toxicity criteria (y-axis) over time (x-axis) with different treatments is shown.
(b) Colonoscopy image (left panel) and H&E stained biopsy section (right panel) from the initial diagnosis of the immune checkpoint inhibitor-related colitis showing mucosal edema and ulcerations.
(c) Colonoscopy image (left panel) and H&E stained biopsy section (right panel) after successful ECP treatment showing no signs of an autoimmune colitis. Colonic crypts show regular morphology without granulocytic infiltrations, apoptosis or crypt loss.
(d) tSNE plot visualizing the peripheral lymphocyte compartment of the patient before and 8 weeks after start ECP.
(e) Relative NK cell numbers before and after ECP treatment.
(f) Expression intensity of CD16, CD56 and CD57 on NK cells (defined as single/live/$CD45^{pos}$/$CD14^{neg}$/$CD3^{neg}$/$CD19^{neg}$/$CD56^{pos}$) from healthy, age-matched donors (HD, n=5) and the patient at week 53 and week 75 after ECP start visualized by UMAP. FlowSOM clustered NK cell subsets are overlaid on the two plots on the right.
(g) MFI (median marker expression, value range: 0-1) of CD16 on $CD56^{dim}$ mature NK cells. HD (n=5), whiskers of boxplot represent the minimum and maximum values of the HD dataset.
(h) Survival of mice injected with T cells (Tc) with or without NK cells (NK dose of $4x10^4$ or $4x10^5$ per mouse) in a model of anti-PD1 antibody-induced irAE as described in Suppl. Appendix. ** p= 0.003, **** p<0.0001.

2 References

1. Mastroianni, J., Stickel, N., Andrlova, H., Hanke K, Melchinger W, Duquesne S, Schmidt D, Falk M, Andrieux G, Pfeifer D, Dierbach H, Schmitt-Graeff A, Meiss F, Boerries M, Zeiser R. miR-146a Controls Immune Response in the Melanoma Microenvironment. *Cancer Res* **79**, 183-195 (2019).
2. Johnson, D.B., Chandra, S. & Sosman, J.A. Immune Checkpoint Inhibitor Toxicity in 2018. *JAMA* **320**, 1702-1703 (2018).
3. Hodi, F.S.*, et al.* Improved Survival with Ipilimumab in Patients with Metastatic Melanoma. *New England Journal of Medicine* **363**, 711-723 (2010).
4. Topalian, S.L.*, et al.* Safety, Activity, and Immune Correlates of Anti–PD-1 Antibody in Cancer. *New England Journal of Medicine* **366**, 2443-2454 (2012).
5. Brahmer, J.R.*, et al.* Safety and Activity of Anti–PD-L1 Antibody in Patients with Advanced Cancer. *New England Journal of Medicine* **366**, 2455-2465 (2012).
6. Zeiser, R., Blazar, B.R. Acute Graft-versus-host disease - Biologic process, prevention, and therapy. *N Eng J Med* **377**, 2167-2179 (2017).
7. Zeiser, R., Blazar, B.R. Pathophysiology of Chronic Graft-versus-Host Disease and Therapeutic Targets. *N Eng J Med* **377**, 2565-2579 (2017).
8. Apostolova, P., Unger, S., Meiss, F., von Bubnoff, D., Aumann, K., Becher, B., Zeiser, R. Extracorporeal photopheresis for severe immune checkpoint inhibitor-induced autoimmune colitis. *N Eng J Med* **382**, 294-296 (2020).
9. Alcindor, T.*, et al.* Immunomodulatory effects of extracorporeal photochemotherapy in patients with extensive chronic graft-versus-host disease. *Blood* **98**, 1622-1625 (2001).
10. Ni, M.*, et al.* Shaping of CD56(bri) Natural Killer Cells in Patients With Steroid-Refractory/Resistant Acute Graft-vs.-Host Disease via Extracorporeal Photopheresis. *Front Immunol* **10**, 547 (2019).
11. Romee, R.*, et al.* NK cell CD16 surface expression and function is regulated by a disintegrin and metalloprotease-17 (ADAM17). *Blood* **121**, 3599-3608 (2013).
12. Goodier, M.R.*, et al.* Sustained Immune Complex-Mediated Reduction in CD16 Expression after Vaccination Regulates NK Cell Function. *Front Immunol* **7**, 384 (2016).

13. Braun, L.M., Zeiser R. Immunomodulatory Therapies for the Treatment of Graft-versus-host Disease. **5**, e581. (2021).
14. Postow, M.A., Sidlow R, Hellmann MD. Immune-Related Adverse Events Associated with Immune Checkpoint Blockade. *N Eng J Med* **378**, 158-168 (2018).
15. Knobler, R., Berlin G, Calzavara-Pinton P, Greinix H, Jaksch P, Laroche L, Ludvigsson J, Quaglino P, Reinisch W, Scarisbrick J, Schwarz T, Wolf P, Arenberger P, Assaf C, Bagot M, Barr M, Bohbot A, Bruckner-Tuderman L, Dreno B, Enk A, French L, Gniadecki R, Gollnick H, Hertl M, Jantschitsch C, Jung A, Just U, Klemke CD, Lippert U, Luger T, Papadavid E, Pehamberger H, Ranki A, Stadler R, Sterry W, Wolf IH, Worm M, Zic J, Zouboulis CC, Hillen U. Guidelines on the use of extracorporeal photopheresis. *J Eur Acad Dermatol Venereol.* **28**, 1-37 (2014).
16. Nygaard, M., Wichert, S., Berlin, G., Toss F. Extracorporeal photopheresis for graft-vs-host disease: A literature review and treatment guidelines proposed by the Nordic ECP Quality Group. *Eur J Haematol.* **doi: 10.1111/ejh.13381. [Epub ahead of print]**(2020).
17. Haanen, J.B.A.G., Carbonnel F, Robert C, Kerr KM, Peters S, Larkin J, Jordan K; ESMO Guidelines Committee. Management of toxicities from immunotherapy: ESMO Clinical Practice Guidelines for diagnosis, treatment and follow-up. *Ann Oncol.* **29**, iv264-iv266 (2018).

3 Overall risk benefit assessment

IrAE occur in up to 61,1% and 39-41% in patients treated with CTLA-4 and PD-1/PD-L1, respectively [3-5]. Especially with combination therapy, serious irAE (defined as at least grade III according to CTC-AE) occur in up to 50% of all patients [14]. The most commonly affected organs are the skin, the gastrointestinal system, the liver and the endocrine glands. More rare manifestation include pneumonitis, nephritis, myocarditis and CNS involvement. There have been no prospective trials regarding the management of irAE and this leads to significant variability in institutional practice [14]. Most commonly used treatments include withdrawal of immune checkpoint inhibitors, corticosteroids, mycophenolate mofetil, infliximab or cyclosporine A[16]. Immunosuppressive treatment might impair the anti-tumoral effect of immune checkpoint inhibitors and discontinuation of the treatment might increase the risk for tumor progression [14].

ECP is an established treatment for acute and chronic graft-versus-host disease, cutaneous T cell lymphoma, systemic sclerosis, transplant rejection and autoimmune diseases [15]. During ECP, mononuclear cells from the peripheral blood are collected, mixed with a photoactivating compound (methoxsalen) and exposed to ultraviolet light [16]. The mononuclear cells are then re-infused to the patient. ECP treatment leads to crosslinkage of DNA strains and apoptosis. The mechanism of action is not clearly understood, however it is well established that ECP has an immunoregulatory effect by modulating T cell and natural killer (NK) cell populations and inducing immune tolerance [16]. ECP is generally very well tolerated. The incidence of adverse events is extremely low at less than 0.003% [16] making ECP an extremely safe procedure. When considering the high morbidity and possible mortality of treatment-refractory irAE, the risk benefit assessment for ECP is highly positive. This study will assess the safety of ECP in this particular patient population with irAE after immune checkpoint inhibitor treatment.

3.1 Covid 19 – Risk-Benefit Assessment

Data on COVID-19 infections in irAE patients after Immune checkpoint inhibition (ICI) are scarce. A recent study analyzed whether patients with cancer treated with ICI are more or less vulnerable to COVID-19 (Ghosh N, Tirpack A, Chan KK, Bass AR. Impact of COVID-19 on patients with rheumatic complications of cancer immunotherapy: results of a registry survey. J Immunother Cancer. 2020 Oct;8(2):e001550). The authors reported that six patients (10%) had definite or probable COVID-19, but all recovered uneventfully, including two still on ICI and on low-to-moderate dose prednisone. Of the 25 on ICI within the last 6 months, seven (28%) had their ICI held due to the pandemic. In patients on immunosuppression for irAE, none had changes made to those medications as a result of the pandemic. The incidence of COVID-19 was no higher in patients still on ICI.

Given the fact that diagnostic and therapeutic procedures of the irAE patients require frequent hospitalization and that steroids causes immunosuppression, IRAE patients are at a higher risk for a COVID-19-infection. However, irAE is an aggressive disease, that can lead to death within a few months after initial diagnosis, when untreated. This is why prompt diagnostic and therapeutic procedures are crucial for the outcome of irAE patients. Frequent hospitalization for adequate diagnostic and therapeutic procedures combined with therapy-related-immunosuppression are inevitable for AE patients treated in curative intent, irrespective of participation in this trial. However, all prevailing hygiene measures will be strictly implemented in all diagnostic and therapeutic procedures performed in study participants regarding the

execution of this study to keep the risk of a COVID-19-infection as low as possible. Moreover, the possibility of a vaccination for COVID-19 prior or during the participation in this trial will be actively discussed with each participant based on the respective recommendations of expert associations and SmPCs. In conclusion with reference to the safety of the trial participants which is of primary importance, and risks of involvement in the trial a negative benefit-to-risk-ratio for the trial participant regarding the COVID-19 pandemic is not expected.

4 Study Design

This is an open-label, multicentric, non-randomized single arm phase 1/2 study.
A non-randomized design was chosen due to the fact that there is a high variety in the institutional practice for treating patients with corticosteroid-refractory irAE. This study aims at describing the response rates to ECP treatment on a systematic basis which has so far been used only in few case reports.

4.1 Number of patients

50 patients will be assessed for eligibility, 30patients will be included in this trial.

4.2 Participating sites

4 sites are planned in Germany, which meet the structural and personnel requirements
for performing the planned regular trial-related investigations.
If necessary, additional qualified sites can be included during trial conduct.

4.3 Time Schedule

Enrollment is expected to be completed after 24 months. Treatment duration will be 12 weeks and follow-up will be additional 12 months. Responders can receive additional treatment up to 60 weeks. This time frame was chosen based on our experience from treating patients with irAE with ECP.

First patient first visit:	Q3/2022
Last patient included:	Q3/2024
Last patient last visit:	Q4/2025

5 Study Population and patient eligibility

5.1 Target population

Patients will only be allowed to enter the trial if they provide written informed consent to their participation (following full explanation of the trial) (see sections 5.3 and 5.4).

5.2 Gender distribution

No gender ratio has been stipulated in this trial, as the results of the preclinical and clinical studies did not indicate any gender effect of the trial treatment in terms of efficacy and safety.

5.3 Inclusion Criteria

For inclusion into the trial, patients must fulfill all of the following criteria:

1. Male and female patients aged ≥18 years with adequate German written and oral language skills
2. Written informed consent:
    - Subjects must have signed and dated an IRB/IEC approved written informed consent form in accordance with regulatory and institutional guidelines. This must be obtained before the performance of any protocol related procedures that are not part of normal subject care.
    - Subjects must be able to understand and willing to comply with scheduled visits, treatment schedule, laboratory tests and mandatory collection of blood, and other requirements of the study.
    - Subject Re-enrollment: This trial permits the re-enrollment of a subject that has discontinued the study as a screening failure. A re-screening can be performed if previously non-fulfilled inclusion or reversible exclusion criteria have changed or if a previous immunosuppressive therapy (other than corticosteroids) has been stopped. If re-enrolled, the subject must be re-consented.
3. Target population
    - Patients who have received treatment with an anti-PD-1, anti-PD-L1 or an anti-CTLA-4 antibody or any combination of these for any type of malignancy in the last 24 months before screening. 30% of the patients that will be included should have non-skin cancer.
    - Patients must have diagnosis of immunogenic adverse events after exclusion of other causes of this AE as per standard guidelines.
    - Patients should have clinical or histological evidence of at least one of the following immune-related adverse events:
        - Colitis
            - Diarrhea with increase of ≥4 stools over baseline
            - No improvement after 72h treatment with at least 1 mg/kg BW/day prednisolone equivalent
        - Hepatitis
            - Alanine aminotransferase and/or aspartate aminotransferase ≥3x ULN if baseline was normal; ≥3x baseline, if baseline was abnormal and/or total bilirubin >1.5 ULN
            - No improvement after 72h treatment with at least 1 mg/kg BW/day prednisolone equivalent
        - Pneumonitis
            - Radiographic changes that involve more than one lobe of the lung or ≥25% of lung parenchyma and new symptoms such as cough, dyspnea or new oxygen therapy
            - No improvement after 72h treatment with 1 mg/kg BW/day prednisolone equivalent
        - Dermatitis
            - Rash covering >30% of the body surface area and moderate or severe symptoms
            - No improvement after 72h treatment with at least 1 mg/kg BW/day prednisolone equivalent

- Patients must have received standard-of-care treatment for irAE with corticosteroids at least 1 mg/kg body weight for at least 72 hours.
- Patients can have received maximum of one additional (second line) therapy after Steroid treatment before ECP starts

4. Maximum of one additional (second line) therapy after Steroid treatment before ECP starts (e.g. infliximab for colitis)

5. Women of childbearing potential (WOCBP) must have a negative serum pregnancy test (minimum sensitivity 25 IU/L or equivalent units of HCG) within 1 week prior to the start of study drug. Males who are sexually active with WOCBP must use during the duration of the study and up to 5 months afterwards a latex or synthetic condom during any sexual contact with females of reproductive potential, even if they have undergone a successful vasectomy (see also 10.9). .
6. Women must not be breastfeeding.
7. ECOG performance status 0, 1, or 2.

5.4 Exclusion Criteria

Patients are not eligible for this study if they fulfill one or more of the following exclusion criteria:

1. Active treatment in a clinical study of any investigational agent within 14 days prior day 0 or within 5 half-lives of the study treatment, whichever is greater.
2. Positive result for HIV.
3. Active COVID-19-infection or non-compliance with the prevailing hygiene measures regarding the COVID-19 pandemic
4. Prior allogeneic bone marrow transplantation or prior solid organ transplantation.
5. Patients who require vasopressors, and/or have NYHA class III or IV heart failure.
6. Uncontrolled hypertension or ventricular arrhythmias.
7. Previous or concurrent malignancies within the last 3 years of enrollment other than the disease for which checkpoint-inhibitor blockade was applied. Exceptions are adequately treated basal or squamous cell skin cancer, or any other cancer from which the subject has been disease-free for more than 3 years.
8. Any condition that would, in the investigator's judgment, interfere with full participation in the study, including administration and contraindications of study drug/study procedure and attending required study visits; pose a significant risk to the subject; or interfere with interpretation of study data
9. Known allergies, photosensitive disease, hypersensitivity, or intolerance of methoxypsoralen or psoralen compounds, excipients, or similar compounds
10. Aphakia
11. Sexually active men and female patients of child-bearing potential who are not willing to use highly effective methods of contraception during the trial and at least 5 months after the last ECP procedure (see also 10.9)
12. Inability to tolerate extracorporeal volume loss
13. Previous splenectomy
14. Pregnancy and lactation

6 Objectives and endpoints

The primary objective of this phase 1/2 trial is a first evaluation of the safety of ECP treatment for patients with irAE induced by treatment with an anti-CTLA-4, anti-PD-1, anti-PD-L1 antibody or a combination. We have previously employed ECP as a single-case treatment

approach in a patient with corticosteroid-, anti-TNF-antibody- and cyclosporine A-refractory colitis. In order to evaluate the response to ECP treatment in a systematical manner, we launch this multicentric phase 1/2 trial to evaluate the safety. As a secondary endpoint, response rates of irAE will be assessed by monitoring clinical and laboratory parameters as well as the overall survival.

6.1 Overview objectives and endpoints

Table 1 Objectives and related endpoints

Objective	Endpoint
**Primary endpoint**	
To evaluate the rate of treatment-related adverse events (AEs) and serious adverse events (SAEs) in patients treated with ECP for immune-checkpoint inhibitor-induced colitis, pneumonitis, hepatitis or dermatitis.	Rate of ECP-related adverse events and serious adverse events. A positive result of the study is defined as ≤50% of the patients developing a treatment-related SAE.
**Secondary Endpoints**	
To assess the efficacy of ECP in patients with irAE with immune-related colitis, hepatitis, pneumonitis, dermatitis	Objective response rate (ORR) at week 6 and 12 after start of ECP treatment assessed by the investigator according to the criteria in 6.2. IrAE severity is assessed according to CTCAE V5.0 grading (Appendix 1) to monitor response rates.
To assess time to treatment response (TTTR)	Time to response is defined as time from treatment start to the date of first documentation of PR or CR. Death without prior response will be considered to be a competing event.
To assess the duration of investigator-assessed clinical response	The duration of response is assessed for responders only by calculating the time from first response to the date of first observation of irAE relapse/progression/death or the date of additional systemic treatment for irAE.
To assess Overall Survival (OS)	OS is defined as time from treatment start to the date of death from any cause.
To measure the time to complete discontinuation of other immunosuppressive therapy	The time between start of ECP and the last day on which another immunosuppressive drug was administered for the treatment of irAE
To assess changes in immune cell phenotype and metabolism during treatment	Changes in immune cell phenotype and metabolism as described in section 13.5.2
To assess quality of life	EORTC QLQ-C30 and EORTC QLQ-HDC29.
To assess the relapse rates of irAEs upon discontinuation of ECP and rechallenge with an immune checkpoint inhibitor	Incidence of irAEs in patients after discontinuation of ECP<br>Incidence of irAEs in patients after re-challenge with an immune checkpoint inhibitor

Objective	Endpoint
To assess the incidence of tumor progression or relapse.	Incidence of tumor progression or relapse, calculated as time from start of treatment until disease progression or relapse. Death without prior tumor progression / relapse is a competing event.

6.2 Response definitions related to irAE

Response definitions are based on the ESMO Clinical Practice Guidelines for diagnosis, treatment and follow up of toxicities from immunotherapy [17]. Scoring of response will be compared with the time point directly prior to start of study treatment (Visit week 1 day 0).

6.2.1 Complete Remission (CR)

CR is defined by:

- Complete resolution of clinical and laboratory parameters of the irAE present at study enrolment
- Corticosteroids should be discontinued without symptom rebound for at least 1 week.

6.2.2 Partial Remission (PR)

PR is defined by:

- Decrease by at least one grade according to CTC-AE V5.0 (Appendix 1):
  - o Colitis: Diarrhea with increase < 4 stools over baseline
  - o Hepatitis: AST and ALT < 3x ULN if baseline was normal and <3x baseline if baseline was abnormal
  - o Pneumonitis: partial resolution of clinical symptoms
  - o Dermatitis: < 30% of body surface area affected
- In patients with >1 irAE at the time point of enrolment, PR will be defined by a decrease of at least one grade in at least one irAE.

6.2.3 Progressive disease (PD)

PD corresponds to an increase of any irAE by at least one grade according to CTC-AE V5.0.

6.2.1 Stable disease (SD)

SD is defined by no change in the severity of irAE according to CTC-AE V5.0.

6.2.2 Overall response rate (ORR)

ORR is defined as the proportion of patients achieving CR or PR.

7 Trial Conduct

7.1 Patient eligibility

If a patient appears to be eligible for the trial, the investigator will inform the patient about the trial and ask the patient for his/her written consent. It is imperative that written consent is obtained prior to any trial-specific procedures. The investigator will then record the details of these trial patients on the following trial-specific lists:

- **Subject Screening log:** for the documentation of the trial patients who were checked for eligibility before the clinical trial. The following will be entered: the identification code, the dates of written consent, screening and mode of enrolment as well as details of whether the patient was enrolled in the trial and, if not, the reason for not enrolling the patient.

- **Subject Identification log:** A confidential log of the names of all trial patients with the identification code assigned to each patient at the time of enrolment in the clinical trial. With this list, the identity of each patient can be revealed. The list must be kept confidential and must not leave the institution. It must remain at the trial centre and must not be copied or otherwise passed on. Monitors, auditors and representatives of authorities must be allowed to inspect the list on request.

7.2 Study treatment

This trial will include patients with corticosteroid-refractory irAE (colitis, hepatitis, pneumonitis or dermatitis) after treatment with an anti-PD-1, anti-PD-L1 or anti-CTLA-4 antibody or a combination of those. All patients will be treated with ECP as described below. The ECPs will be performed in the leukapheresis outpatient ward by a specialized team which is trained for the handling of the ECP device.

7.3 Treatment with investigational procedure including the investigational medicinal product (methoxsalen)

7.3.1 ECP, duration of treatment

- Study treatment will be administered (see section 10.10) for twelve weeks or longer if the patient experiences benefit from treatment with ECP. In this case treatment might be prolonged at the discretion of the investigator for a total of 60 weeks. In patients who achieve CR, ECP treatment might be discontinued before week 12 after discussion with the coordinating investigator. In case of persisting irAE after 60 weeks with response to ECP therapy, f.e. after ICI re-exposition, the continuation of ECP might be reasonable and can be performed at the discretion of the investigator.
- Patients who have malignant disease progress during treatment will discontinue ECP and will be treated at the discretion of the investigator.
- In case of deterioration of the irAE within the first four weeks of treatment with ECP, study treatment may be permanently discontinued due to lack of activity at the discretion of the investigator. In this case, an individual case-by-case discussion with the coordinating investigator is mandatory.
- In case of irAE relapse after discontinuation of ECP, the treatment may be reinstituted. Other possible treatment options will be considered according to the current state of the art and the patient will be informed about expected benefit from reinstitution of ECP treatment as well as possible benefit from other treatments.
- In case of a treatment-related adverse event ≥ 3, the study treatment will be permanently discontinued.
- In the case of pregnancy, the study treatment will be permanently discontinued.

7.3.2 ECP, procedure recommendations

- Patients will be treated with two cycles of ECP carried out on two consecutive days. During each treatment, 1.5 l blood will be processed using an ECP device. Treatment will be repeated every week for the first 4 weeks, from week 5 to 12 every two weeks (on 2 consecutive days). Patients who achieve CR, might discontinue treatment prior to week 12 after discussion with the coordinating investigator. Patients who have benefit from the treatment until week 12, but do not achieve a CR, might continue treatment for a maximum total of 60 weeks.
- Hematological adverse events will be recorded but however, dose interruptions and dose reductions during treatment due to hematological adverse events are optional as required in the investigators opinion. Also, baseline cytopenias are not prohibitive for inclusion.
- A non-hematologic adverse event **NOT** suspected to be drug-related **BUT** suspected to be related to irAE or the underlying malignancy, does not require interruption or reduction of ECP.
- In case of an adverse event not allowing retreatment within 4 weeks after interruption, the coordinating investigator should be contacted to discuss subsequent procedures (e.g. discontinuation of study treatment).

7.3.3 ECP, combination with corticosteroids

Corticosteroids started before treatment with ECP might be continued at the discretion of the investigator. Upon response to corticosteroids+ECP, it is recommended to discontinue corticosteroids first, followed by ECP.
Please note that the addition of any new systemic treatment for irAE until the end of month 3 after start of treatment will be considered as a progressive disease. These patients will be followed up to understand if they benefit from a change in therapy.

7.3.4 ECP, combination with other drugs

ECP is a procedure based on the application of 8-methoxypsoralen and the extracorporeal UV-A light exposure of peripheral blood leukocytes. 8-methoxypsoralen is a known inhibitor of CYP2A6.
The use of systemic immunosuppressants other than corticosteroids at screening or after start of ECP treatment is not allowed.

The ICI-therapy might be continued during the course of the study in patients with controlled irAE symptoms and after careful assessment of risks and benefits by an interdisciplinary team.

7.4 Concomitant medication

Concomitant medication has to be thoroughly documented in the Case Report Forms (CRF) as described in section 7.7.15.

7.5 Treatment after end of trial

After end of the trial, the therapy of the given indications will be performed according to investigators choice.

7.6 Post-trial-interviews (PTI)

At the end of the treatment phase, the patients receive information on voluntary participation in a post-trial-interview. For this purpose, a flyer from Mallinckrodt (MNK) Pharmaceuticals can be handed out by the study personnel at EOT visit or later, including the follow up phase up to the EOS visit. As a subsidiary of MNK, Therakos is the IMP manufacturer and provides the ECP devices.
This process and the PTI itself are not part of the eCRF and/or the clinical monitoring and will be coordinated exclusively from MNK.

7.7 Visit schedule and assessments

7.7.1 Flow chart and visit schedule

The schedule of assessment (Table 2 and
Table 3) lists all of the assessments and indicates with an "X" when they have to be performed. All data obtained from these assessments must be available in the patient's source documentation.
Patients will have regular visits once per week.
Treatment with ECP for irAEs will include 2 ECP cycles performed on consecutive days every week for 4 weeks, from week 5 to 12 every other week ECP (2 consecutive days).

Table 2 **Study procedure and Flow Chart (Screening and treatment phase)**

Assessments	Timepoint / Section	Screening Examination -28 days to day -1	Treatment period from day 1 to week 12 *(± 2 days)*												End of Treatment (EOT) = week 12, day 84 (± 2 days) or premature	Additional treatment phase[1] *(± 2 days)*	
			Week 1, day 1	Week 2, day 8	Week 3, day 15	Week 4, day 22	Week 5, day 29	Week 6, day 36	Week 7, day 43	Week 8, day 50	Week 9, day 57	Week 10, day 64	Week 11, day 71	Week 12, day 78		Week 13–59	Week 14–60
																Alternating biweekly	
Informed Consent	16.3	x															
Patient eligibility	5, 7.1	x															
Demographic data	7.7.6	x															
Medical history	7.7.7	x															
Current disease status[2]	7.7.8	x															
Registration	7.7.2	x															
ECP (always 2 consecutive days)	7.3		x	x	x	x	x		x		x		x			x	
Physical examination incl. Weight/Height and ECOG	7.7.9	x	x	x	x	x	x		x		x		x		x	x	
Clinical irAE Grading[3]	7.7.10	x	x	x	x	x	x	x	x	x	x	x	x	x	x	x	x
Laboratory (Hematology, clinical chemistry, coagulation parameters, pregnancy test)[4]	7.7.11	x	x	x	x	x	x	x	x	x	x	x	x	x	x	x	x
Hepatitis B and C and HIV screening		x															
EDTA and serum samples for scientific analysis (only Freiburg)	7.7.12	x	x		x		x		x						x		
EORTC QLQ-C30 and QLQ-HDC29	7.7.14	x		x				x						x	x		
Concomitant Medication related to tumor therapy or immunosuppression[5]	7.7.15	x	x	x	x	x	x	x	x	x	x	x	x	x	x	x	x
(Serious) Adverse Events[6]	7.7.16,10.1	x	x	x	x	x	x	x	x	x	x	x	x	x	x	x	x
Dermatological examination	7.7.17	x							x						x		
Ophthalmological examination	7.7.18	x							x						x		

Assessments	Timepoint / Section	Screening Examination -28 days to day -1	Treatment period from day 1 to week 12 *(± 2 days)*												End of Treatment (EOT) = week 12, day 84 (± 2 days) or premature	Additional treatment phase[1] *(± 2 days)*	
			Week 1, day 1	Week 2, day 8	Week 3, day 15	Week 4, day 22	Week 5, day 29	Week 6, day 36	Week 7, day 43	Week 8, day 50	Week 9, day 57	Week 10, day 64	Week 11, day 71	Week 12, day 78		Week 13–59	Week 14–60
																Alternating biweekly	
Response assessment[7]	7.7.13								x						x	(x)[7]	(x)[7]
Post Trial Interview[8] (optional)	7.6														x[8]	(x)[8]	(x)[8]

Table 3 Study procedure and Flow Chart (Follow Up / End of study)

Assessments	Timepoint / Section	Follow Up starting with the last ECP procedure until end of month 12					
		Visits in FU phase *(± 14 days)*					End of Study
		Month 2	Month 4	Month 6	Month 8	Month 10	Month 12
Physical examination incl. weight/height and ECOG	7.7.9	x	x	x	x	x	x
Clinical irAE grading[3]	7.7.10	x	x	x	x	x	x
Laboratory(Hematology, clinical chemistry)[4], pregnancy test – Only until Month 6	7.7.11	x	x	x	x	x	x
EDTA and serum samples for scientific analysis (only Freiburg)	7.7.12			x			x
EORTC QLQ-C30 and QLQ-HDC29	7.7.14		x				x
Concomitant Medication[5]	7.7.15	x					
Dermatological examination	7.7.17	x	x	x	x	x	x
Ophthalmological examination	7.7.18			x			x
Post Trial Interview[8] (optional)	7.6	(x)[8]	(x)[8]	(x)[8]	(x)[8]	(x)[8]	(x)[8]

[1] Study treatment with ECP will be performed for 12 weeks or as long as the patient experiences benefit from treatment with ECP. In this case treatment might be prolonged at the discretion of the investigator (maximal 60 weeks). In case of reappearance of irAE after ECP has been discontinued, treatment with ECP may be reinstituted. At end of treatment the same assessments will be done as week 12.
[2] Current disease status: Previous treatment of the underlying disease including treatment duration, dose and response and earlier infectious or immune-related complications have to be documented.
[3] According to the ESMO guidelines for management of toxicities from immunotherapy, Appendix 1. IrAE grading should be performed at every visit in the case of a premature end of treatment or in the case where the additional treatment phase is performed.
[4] Local lab: hematology and clinical chemistry will be performed according to clinical routine. In the CRF only the following parameters will be documented: hemoglobin, leukocytes, platelets, coagulation parameters (INR, PTT), renal function (creatinine) and liver function (AST, ALT, bilirubin). Pregnancy test 1x every month during the treatment phase. Laboratory data have to be checked by the investigator and in case of a clinically relevant abnormality an AE has to be recorded on the AE-page in the CRF.
[5] Please check possible drug interactions before prescribing/administration of any new medication. All concomitant medication will be documented in the electronic chart of the patient (e.g, Meona system). CM will be documented in the CRF as described in section 7.5.14.
[6] (Serious) Adverse events will be reported as described in section 7.5.15 and 10.1. The reporting period begins with ICF signature and ends 30 days after the last ECP procedure.
[7] Response assessments will be performed after 6 and 12 weeks of treatment have been completed (i.e. on the first day of week 7 and on the last day of week 12). These response assessments should be performed at these designated time points also in the case of a premature end of treatment. In the case of premature end of treatment, response will be assessed during the EOT visit as well. In the case of an additional treatment beyond the 12-week phase, a response assessment will be performed again at end of treatment. Lung function testing must be performed for evaluation of the response in patients with pneumonitis.
[8] At the end of the treatment phase, the patients receive information on voluntary participation in a post-trial-interview. For this a flyer from MNK can be handed out at EOT visit or later. The post-trial interviews are not documented in the eCRF and are not part of the clinical monitoring.

7.7.2 Screening and registration

The investigator is obliged to give the patient thorough information about the trial and the trial related assessments, and the patient should be given ample time to consider his or her participation. The investigator must not start any trial-specific procedure before Informed Consent Form (ICF) is signed and dated by both the patient and the investigator. The investigator must keep the original signed ICF (a signed copy is given to the patient) (see section 16.3).
After being informed about the trial and after giving their written Informed Consent, patients have to undergo the examinations listed in Table 2 prior to registration. Results of examinations routinely performed will be accepted, if they were done within day -28 to day -1 prior to study treatment start.
Patients must meet all inclusion criteria and none of the exclusion criteria to be considered eligible.
Patients considered eligible by the investigator should be registered to the trial.

The registration will be carried out with a patient code at the study site on the electronic registration form in secuTrial® - an electronic data capture (EDC) software.

The patient identification code[a] assigned for the trial will be entered into a registration form.
Subsequently, questions on eligibility (inclusion criteria met, exclusion criteria not met, informed consent present), year of birth and gender must be answered on a designated form. This form has to be complete and correct. Otherwise, it cannot be saved.
An automatic confirmation email will be sent to the Clinical Trials Unit (CTU), responsible data manager and project manager.
If questions on the patient eligibility are answered with a “no”, then the remaining screening visit forms will not be editable.

7.7.3 Data to be collected on screening failures

Trial sites are required to document all screened patients on the screening log (see section 7.1). If a screened subject is not included/registered, the reason(s) should be recorded in the source documents and on the screening log.
Screening failures are defined as patients who signed an ICF but failed to be registered in the study for any reason. For these patients, only the reason for screening failure and AE/SAE (if applicable) will be documented in the eCRF.

[a] Patient identification code: a unique trial-specific identification number which identifies the patient and consists of two parts: The first 2 digits correspond to the site number, the next 3 digits stand for a consecutive number of the patient enrolled at a particular site, for example: 01-002 (Site No. 1, Patient No. 2) so that each patient is numbered uniquely across the entire database. The site number is a consecutive number assigned centrally at the CTU, The patient number is assigned at the trial site. A PID Generator will not be used. The patient identification numbers on the registration form will be checked during the registration process by staff members of the central registration office (CTU). Any ambiguities or missing answers on the registration form will be requested. Only unique patient identification numbers will be accepted.

7.7.4 Assessments and specifications

Screening evaluations have to be performed within 28 days prior to the trial treatment procedure.
Following inclusion in the trial and initiation of trial treatment (Day 0), the patient should visit the trial site every week (until week 12 respectively for up to 60 weeks depending on the benefit from the treatment). During the course of the trial, visits and test procedures should occur on schedule whenever possible; visits that occurs ± 2 days (treatment phase) or ± 14 days (follow up) from the scheduled date will not constitute any protocol deviation.
The following assessments will be done according the Flow Chart (see Table 2 and Table 3).

7.7.5 Informed consent, Inclusion-/Exclusion criteria

The investigator is obliged to give the patient thorough information about the trial and the trial related assessments, and the patient should be given ample time to consider his or her participation. The investigator must not start any trial-specific procedure before Informed Consent Form (ICF) is signed and dated by both the patient (and impartial witness, if applicable) and the investigator. The investigator must keep the original signed ICF and a signed copy is given to the patient (see section 16.3). Patient eligibility is to be assessed including serum pregnancy test in females of child-bearing potential.

7.7.6 Patient demographics

Patient’s demographics comprise year of birth, sex and childbearing potential.

7.7.7 Medical history

The medical history includes the following items and has to be documented in the eCRF:

- earlier relevant medical conditions
- or/and e.g. surgeries

7.7.8 Current disease status

Previous treatment of the underlying disease has to be documented in the eCRF and include:

- any treatment for irAE after first diagnosis with dose and duration
- response (CR yes/no)

7.7.9 Physical examination

Thorough physical/medical examination includes, but is not limited to vital signs, cardiovascular, gastrointestinal, hepatobiliary, respiratory, musculoskeletal, genitourinary/renal and other organ systems. Physical examination incl. height (only at screening), weight and the ECOG status. Clinical significant findings during screening examination will be documented as medical history.
Clinical significant findings not present at screening will be documented as adverse events. For documentation of (S)AE please refer to section 10.6.

7.7.10 irAE grading

irAE grading will be performed for each irAE according to CTCAE V5.0 (Appendix 1.)

7.7.11 Laboratory tests

All needed laboratory examinations will be performed according to clinical routine. Following laboratory tests will be documented in the eCRF

- hemoglobin, leukocyte, platelet count
- coagulation parameters (INR, PTT)
- renal function (creatinine)
- liver function (AST, ALT, bilirubin)
- Pregnancy test 1x every month during the treatment phase and 5 months after the last ECP procedure.

All laboratory data have to be checked by the investigator and in case of a clinically relevant abnormality an AE has to be recorded on the AE-page in the eCRF. For details on reporting period please refer to section 10.2.

7.7.12 EDTA and serum samples for scientific analysis (Freiburg only)

Blood has to be collected before treatment start (day -30 to 0) and day 1, day 15, day 29, day 43 and day 85 of treatment start as well as 6 and 12 months after end of ECP treatment for local assessment of immune cell metabolism and phenotype according to the study specific sample handling manual. EDTA and serum samples should be drawn at Screening and on indicated timepoints before start of the ECP treatment on first day of ECP and serum samples should be drawn additionally on the consecutive second day of ECP after ECP treatment.

The analyzed parameters will include: number of T cells, number of NK cells, cytokine expression by T cells and NK cells, CD16 expression by NK cells, serum level of pro-inflammatory mediators.

7.7.13 Assessment of response

Assessment of response will be performed using an appropriate diagnostic method depending on the manifestation of irAE:

- Colitis: clinical assessment of the number of stools per day, abdominal pain or nausea
- Hepatitis: laboratory measurement of AST and ALT, bilirubin
- Pneumonitis: clinical evaluation of symptoms cough, dyspnea, lung function, oxygen saturation
- Dermatitis: clinical evaluation of the body surface area affected

Assessment of irAE has to be performed at any time point prior to screening and documented in the eCRF. Thus, time frame is NOT limited to day -30 to day -1. Thereafter it has to be performed according to the Flow chart. Assessment of response at week 6 and 12 after first ECP procedure will be performed in all patients including patients who have discontinued treatment prematurely.

7.7.14 Assessment of quality of life

The Quality of life of the patients will be evaluated using the EORTC QLQ-C30 and the QLQ-HDC29 Quality of Life Questionnaires (see Appendix 2 and Appendix 3).
These questionnaires have been developed by the European Organization for Research and Treatment of Cancer (EORTC) and are a validated and well established scoring instrument to

measure the quality of life in patients within clinical trials. It is important that the investigator is not influencing the patient in any way.

7.7.15 Concomitant medication

All concomitant mediation administered at any time during the period starting with the signature of the ICF until 14 days after the last ECP procedure, has to be thoroughly documented in the eCRF.

7.7.16 Adverse events

For details on (S)AE reporting and documentation see section 10.

7.7.17 Dermatological examination

Skin examination will be performed at screening and then at week 7 during the treatment phase, as well as at the end of treatment visit and at every follow-up. In case of suspicious findings, the patient will be referred to a specialist in dermatology.

7.7.18 Ophthalmological examination

Ophthalmological examination with a focus on potential damage of the eye lens will be performed at screening, at week 7 during the treatment phase, at end of treatment, and on month 6 and 12 of follow-up.

8 Discontinuation criteria

8.1 Premature termination of the entire trial

The sponsor/coordinating investigator is under obligation to monitor the progress of the clinical trial with regard to safety-relevant developments and, if necessary, initiate the premature termination of the entire clinical trial. The sponsor/coordinating investigator will be supported in this responsibility by the DMC, if necessary.

The entire clinical trial must be terminated prematurely if:

- the benefit-to-risk ratio for the patients changes markedly,
- the occurrence of ECP related SAEs in 50% of the cases when 15 of 30 patients are enrolled. The SAEs have to be clearly not related to the malignancy.
- the sponsor/coordinating investigator or the DMC considers that the termination of the trial is necessary,
- indications arise that the trial patients' safety is no longer guaranteed,
- the question(s) addressed in the trial can be clearly answered on the basis of an interim analysis,
- the questions(s) addressed in the trial can be clearly answered on the basis of results of another trial on the same subjects,
- an insufficient recruitment rate makes a successful conclusion of the clinical trial unrealisable/no longer feasible.

If the clinical trial is prematurely terminated or suspended for any reason, the investigator should promptly inform the trial patients and ensure appropriate therapy and follow-up for the

patients. Where required by the applicable regulatory requirements, the CA(s) and the IEC(s) will also be informed (this is usually done by the sponsor).

An unexpected death directly related to ECP is a SUSAR (not SAE) and leads to interruption of the trial followed by a review by the independent DMC.

8.2 Premature termination of the trial at one of the trial sites

Both the investigator and the sponsor have the right to terminate the trial at one of the sites.
The clinical trial can be terminated prematurely at his site by the investigator if, for instance unforeseeable circumstances have arisen at the trial site which preclude the continuation of the clinical trial, the investigator considers that the resources for continuation are no longer available, the investigator considers that the continuation of the trial is no longer ethically or medically justifiable.
The sponsor/coordinating investigator can initiate the exclusion of a site from further participation if, for instance, patient recruitment is inadequate, serious problems arise with regard to the quality of the collected data which cannot be resolved.
Premature termination at one of the trial sites does not automatically mean a termination of the trial for already enrolled trial patients. A separate decision on further treatment must be made for each patient, depending on the overall situation. Adequate further treatment and follow-up of already enrolled trial patients must be ensured. The documentation of already enrolled trial patients will be reviewed for completeness and plausibility. Queries may be raised for further clarification before the site is closed. These queries must be answered properly by the site. The CA(s) and IEC(s) must be duly notified of the site´s closure, including reasons, within the specified period. The trial site concerned will be closed in stages by the CRA when a decision has been made on the further treatment of the patients concerned.

8.3 Discontinuation of trial treatment or trial participation for individual patients

8.3.1 Premature termination of trial treatment

All patients who prematurely terminate study treatment will be followed up for 12 months excluding patients withdrawing their consent, who will not be followed up.
In the case of pregnancy, irAE progress during ECP treatment and occurrence of a treatment-related AE grade ≥3, the study treatment will be permanently discontinued. Other potential reasons for discontinuing the patient from the trial treatment may include:

- Adverse events (including intercurrent illnesses) which preclude further treatment with the IMP or make further participation in the clinical trial inadvisable because the informational value of the trial results is impaired.
- Uncontrolled bacterial, viral or fungal infection
- Logistical reasons (patient changes his/her doctor or hospital or moves to another location)

8.3.2 Premature termination of trial participation

The patient is free to withdraw from the study for any reason and at any time without giving reason for doing so and without penalty or prejudice.
In the case of withdrawal, patients will be encouraged to explain why she/he has withdrawn consent but must not to be compelled to do so.

The date of withdrawal and reasons surrounding the exit at the moment of withdrawal must be documented on the CRF. All documentation concerning the patient must be as complete as possible.
The responsible investigator may only withdraw a patient from participation in the trial for the following reasons: extreme circumstances arise which make any trial-relevant follow-up impossible.

9 Investigational Procedure including Investigational Medicinal Product (IMP)

The following medication is defined as IMP in this study:
Methoxsalen sterile solution for the indication of extracorporeal administration with the therakos® uvar xts® or therakos® cellex® photopheresis system in the palliative treatment of the skin manifestations of cutaneous t-cell lymphoma (CTCL) that is unresponsive to other forms of treatment

9.1 Description of IMP

Methoxsalen Sterile Solution was developed exclusively for extracorporeal photopheresis (ECP) with the UVAR® or UVAR XTS® Photopheresis. UVAR and UVAR XTS are first and second generation photopheresis systems. UVAR or UVAR XTS Photopheresis System in conjunction with Methoxsalen (UVADEX) Sterile Solution is currently approved for the palliative treatment of the skin manifestations of cutaneous T-cell lymphoma (CTCL) not responsive to other therapies.
For further characteristics please refer to the current version of the SmPCfor Methoxsalen.

9.1.1 IMP background information

Methoxsalen (8-methoxypsoralen) is a member of the group of compounds known as psoralens or furocoumarins. Methoxsalen is a naturally occurring compound with the chemical name 9-methoxy-7H-furo{3,2-g}[1]-benzopyran-7-one. Methoxsalen is biologically inactive in the absence of photoactivation; approximately 95% is eliminated from the body within 24 hours without demonstrable effect.
Methoxsalen is a photosensitizing agent that preferentially accumulates in epidermal cells.
Methoxsalen is usually used in combination with UV light activation, a treatment known as psoralen plus UV light A (PUVA). In the dark, methoxsalen intercalates with DNA, but does not form covalent bonds. With exposure to long-wave UV energy (UVA), methoxsalen becomes an antimitotic agent with a short half-life. This inhibition of DNA synthesis results in a decrease in cell division and epidermal turnover; both are favorable responses in the treatment of proliferative skin diseases. Photoactivated methoxsalen is also useful in increasing melanin pigmentation of functioning melanocytes in the treatment of vitiligo.

Extracorporeal Photopheresis:
ECP is a procedure that involves collecting blood outside the patient's body and exposing extracorporeally circulating leukocyte-enriched blood to ultraviolet light A (UVA) in the presence of the photoactive drug methoxsalen. In the photopheresis process, the patient is connected to a photopheresis system via a venous catheter interface. Red blood cells are separated from the white blood cells and plasma by centrifugation. Red blood cells and excess plasma are returned to the patient while the leukocyte-enriched blood (buffy coat) and some

plasma are collected in the photoactivation bag. The dose of Methoxsalen (UVADEX) is calculated by the ECP instrument based on the treatment volume. Methoxsalen Sterile Solution (20 µg / mL) is added according the formular defined in the SmPC (treatment volume x 0,017 ml) to the buffy coat fraction in the photoactivation bag. The Methoxsalen--treated buffy coat is then exposed to UVA and re-infused into the patient. UVAR, UVAR XTS, and THERAKOS CELLEX are the first, second and third generation in-line photopheresis systems which perform all steps of the ECP (blood collection, centrifugation, addition of the photosensitizer and photoactivation to the buffy coat fraction and reinfusion of all blood components) in a fully automated way in a closed system.

9.1.2 Pharmacology and Pharmacokinetics

Methoxsalen sterile solution was developed for ECP to eliminate the substantial inter- and intrapatient variability in methoxsalen pharmacokinetics associated with administration by the oral route in humans. Because of the 6- to 15-fold variability in methoxsalen concentrations after oral administration, the minimum effective concentration (50 ng/mL) was difficult to achieve in subjects and often resulted in inadequate clinical responses.
Extracorporeal use of UVADEX Sterile Solution in clinical studies is associated with a much lower systemic exposure to methoxsalen compared with clinical studies using an oral formulation. The ex vivo administered dose is approximately 1/200th of the administered oral dose.

9.2 Summary of safety profile

9.2.1 Expected Adverse Events reported in clinical trials or postmarketing use

The following frequent AEs (1-10%), which might apply to the patient cohort of the present study: :
Hypotension, Dizziness during ECP
Nausea, Vomiting
Fatigue, headache and pyrexia after ECP
Hypertension
Complications of the venous access (dislocation, infection, pain, ecchymosis)
Intolerance of volume dysbalances
electrolyte dysbalances
Anemia, thrombocytopenia after frequent ECP
Infection

Relevant AEs with rare (<0.1%) or unknown frequency are:
Cataract, Chorioretinitis
Thrombosis, Hemolysis
Phototoxic reactions of the skin

SAEs were generally very rare. The following individual cases of SAEs which could be related to ECP::
Hypotension, status epilepticus, infection, electrolyte dysbalances, peripheral edema, hypogammaglobulinemia, anemia, allergic reaction.

Except one case of disease progression of cGvHD, no deaths have been clearly been associated with ECP.

Few post-marketing reports of allergic/hypersensitivity reactions, photosensitivity reactions, dysgeusia and nausea were received, which may have a plausible causal relationship to the use of Methoxsalen. These events are considered expected.

Thus, the ECP can be considered as very safe, especially when comparing with the AE profile of systemic immunosuppression with corticosteroids, cyclosporine A or mycophenolate mofetil and infliximab. For the treatment of GvHD, ECP is considered as a treatment option with few side effects. The morbidity of the irAEs outweighs the possible side effects of ECP.

9.2.2 Reference Safety Information for Assessment of Expectedness of Adverse Events

Please see Reference Safety chapter of the current version of SmPC.

9.3 IMPs pharmaceutical characteristics

The IMP used in this trial is characterised as follows:

Proprietary name:	UVADEX
Name of substance:	Methoxsalen
Manufacturer:	Therakos, Inc., a Mallinckrodt company<br>1425 US Route 206<br>Bedminster, NJ 07921
Approved indications (UVADEX®):	• UVADEX Sterile Solution is indicated for extracorporeal administration with the THERAKOS CELLEX System in the palliative treatment of the skin manifestations of CTCL that are unresponsive to other forms of treatment<br>• UVADEX Sterile Solution is indicated for extracorporeal administration with the THERAKOS CELLEX Photopheresis System for the treatment of steroid-refractory and steroid-intolerant chronic Graft versus Host Disease (cGvHD) in adults following allogeneic hematopoietic stem cell transplantation in Australia<br><br>UVADEX is currently approved for use in conjunction with the CELLEX Photopheresis Systems in the United States, in several European countries via the Mutual Recognition procedure, including the United Kingdom, Germany, Austria, France, Netherlands, Spain, Italy, Portugal, Sweden, Norway, Denmark, Poland, Hungary and the Czech Republic; as well as in Canada, Australia, and several other countries.
Dosage form:	UVADEX Sterile Solution is a liquid methoxsalen for ex vivo use in ECP therapy. With this formulation, UVADEX Sterile Solution is introduced ex vivo into the buffy coat bag during the ECP collection process.
Strength:	UVADEX Sterile Solution is formulated as a sterile solution containing 200 µg methoxsalen per 10 mL.
Over dose:	There are no known reports of overdosage with extracorporeal administration of methoxsalen.

	However, in the event of overdosage, the patient should be kept in a darkened room for at least 24 hours.
Total daily dose:	UVADEX Sterile Solution is added to the UVAR
	XTS Photoactivation Bag using the following formula: 0.017 × buffy coat volume

9.4 Packaging and Labeling

Therakos EMEA Ltd., a Mallinckrodt company provides two Therakos ECP devices (each for one study site) and Uvadex vials and kits free of charge to the lead center Medical center – university of Freiburg, Department of Medicine I and the participating sites.

9.5 Supply and ordering

Therakos EMEA Ltd., a Mallinckrodt company will provide the Therakos ECP devices to each site and the site will order UVADEX vials and kits as described in the drug order instruction/ IMP handling manual.

9.6 Receipt and storage

Trial medication must be received by a designated person at the investigational site/site pharmacy, handled and stored safely and properly, and kept in a secured location to which only the investigator/pharmacists and/or designated assistants have access.

Upon receipt, IMP must be stored according to applicable SmPCs. The investigator will be responsible for ensuring the correct storage and sufficient stocks of the IMP at the site. Where allowed/required, the investigator may/should entrust the IMP, in whole or in part, to an appropriate pharmacist (to be designated in advance) or another appropriate individual who is under the supervision of the investigator.

The investigator or a pharmacist, or another appropriate individual who is designated by the investigator, should maintain records of the delivery of the IMP and the stocks at the study site.

9.7 Destruction of IMP

The IMP stock can be destroyed at the study site as appropriate according to SOP and/or local regulations and according to the study specific IMP manual

10 Safety

10.1 Definition of adverse events

An Adverse Event (AE) is defined as any new untoward medical occurrence or worsening of a preexisting medical condition in a subject participating in a clinical investigation. The AE does not necessarily need to have a causal relationship with the administered study drug. An AE can therefore be any unfavorable and unintended sign (such as an abnormal laboratory finding), symptom, or disease temporally associated with the use of investigational product, whether or not considered related to the investigational product.
The causal relationship to study drug is determined by a physician and should be used to assess all adverse events (AE). For definition of relationship to study medication please refer to section 9.2.
Adverse events can be spontaneously reported or elicited during open-ended questioning, examination, or evaluation of a subject. In order to prevent reporting bias, subjects should not be questioned regarding the specific occurrence of one or more AEs.
An overdose without clinical sequelae is not to be considered an AE. For the purposes of this study, an overdose is defined as a single dose of IMP that exceeds the prescribed dose.

Irrespective of any causal relationship, all AEs spontaneously reported by the patient or observed by the investigator will be continuously documented in the medical record and on the designated case report form (AE eCRF page).

All AEs must be described by diagnosis or, if an underlying diagnosis is not known, by symptoms or medically significant laboratory or instrumental abnormalities. The AEs will be documented as shown in the section below.

Symptoms of a pre-existing disease, medically significant laboratory, or instrumental (e.g. electrocardiographic) abnormalities at baseline will not be considered an AE. However, occurrences of new or worsening of pre-existing symptoms, new laboratory or instrumental abnormalities will be considered an AEs

All AEs, no matter how intense, are to be followed up by the investigator in accordance with good clinical practice until resolved or judged no longer clinically relevant, or in case of a chronic condition, until it is fully characterized.

Non-serious AEs should be followed to resolution or stabilization, or reported as SAEs if they become serious. Follow-up is required for non-serious AEs that cause interruption or discontinuation of study drug and for those present at the end of study treatment as appropriate. All identified non-serious AEs must be recorded and described on the AE eCRF page.

Completion of supplemental eCRFs may be requested for AEs and/or laboratory abnormalities that are reported/identified during the course of the study.

All AEs should be treated appropriately. Such treatment may include possible interruption or discontinuation of ECP, starting or stopping concomitant treatments, changes in the frequency or nature of assessments, hospitalization, or any other medically required intervention. Once an AE is detected, it should be followed until its resolution, an assessment should be made at

each visit (or more frequently, if necessary) of any changes in its severity, its suspected relationship to the study medication, any of the interventions required to treat it, and its outcome.

10.2 Documentation of (S)AEs

The AE and SAE reporting and documentation period begins with the ICF signature and ends 30 days after last ECP procedure.
After this period and until end of the study only SAEs related to IMP as per investigator's judgment will be documented on the eCRF and reported to sponsor.
After end of the study only SAEs related to IMP as per investigator's judgment will be reported to sponsor.

Each AE should be characterized by:

- Diagnosis/symptom
- Severity (according to CTCAE Version 5.0,)
- Relationship to ECP (reasonable possibility that AE is related to investigational product yes / no; see definition below)
- Duration (start and end dates)
- Action taken regarding study drug
- Outcome
- Seriousness (yes/no)

Causality
According to EU guideline 2011/C 172/01 ("CT-3") investigator should assess "whether there is a reasonable possibility" of causal relationship between investigational product and adverse event (article 7.3.2 EU guideline). The definition "reasonable possibility" means that there are facts (evidence) or arguments to suggest a causal relationship (article 7.2.1 EU guideline). Specified more the expression "related" means, that there is evidence or argument to suggest a reasonable causal relationship between the event and the administration of the study drug or the study treatment, e.g. close temporal connection, exclusion of other causes. In this study causality related to the UVADEX drug is captured separately.
The assessment "not related" is appropriate, if the SAE is clearly or most likely explained by other causes even if a potential relationship between study drug and the SAE cannot be completely excluded.

10.3 Laboratory Test Result Abnormalities

The following laboratory test result abnormalities should be captured on the AE eCRF page and if applicable on SAE Report Form:

- Any laboratory test result that is clinically significant or meets the definition of a SAE
- Any laboratory test result abnormality that required the subject to have study procedure discontinued or interrupted
- Any laboratory test result abnormality that required the subject to receive specific corrective therapy.

It is expected that wherever possible, the clinical rather than laboratory term would be used by the reporting investigator (e.g., anemia versus low hemoglobin value).

10.4 Definition and documentation of serious adverse events (SAEs)

SAEs have to be reported and documented in eCRF for the timeperiod stated in section 10.2. That refers to all SAEs, whether related or not related to IMP including those thought to be associated with protocol specified procedures.

If the investigator believes that an SAE is not related to study drug/treatment, but is potentially related to the conditions of the study (such as withdrawal of previous therapy or a complication of a study procedure), the relationship to these factors should be specified in the SAE Report Form.

10.4.1 Definition of SAE

An SAE is any untoward medical occurrence that at any dose:

- results in death
- is life-threatening (defined as an event in which the participant was at risk of death at the time of the event; it does not refer to an event which hypothetically might have caused death if it were more severe)
- requires inpatient hospitalization or causes prolongation of existing hospitalization (see **NOTE** below)
- results in persistent or significant disability/incapacity
- is a congenital anomaly/birth defect
- is an **important medical event** (defined as a medical event(s) that may not be immediately life-threatening or result in death or hospitalization but, based upon appropriate medical and scientific judgment, may jeopardize the subject or may require intervention [eg, medical, surgical] to prevent one of the other serious outcomes listed in the definition above). Examples of such events include, but are not limited to, intensive treatment in an emergency room or at home for allergic bronchospasm; blood dyscrasias or convulsions that do not result in hospitalization.

Any component of a study endpoint that is considered related to study therapy should be reported as an SAE (eg, if death is an endpoint, if death occurred due to anaphylaxis, anaphylaxis must be reported).

NOTE: A hospitalization meeting the regulatory requirement for the “serious” criteria is any inpatient hospital admission that includes a minimum of an overnight stay in a healthcare facility, unless hospitalization is for:

- Routine treatment or monitoring of the studied indication, not associated with any deterioration in condition
- Elective or pre-planned treatment for a pre-existing condition that is unrelated to the indication under trial and has not worsened since signing the informed consent
- Social reasons and respite care in the absence of any deterioration in the patient’s general condition

- Elective hospitalizations for performing ECP or any other trial assessment

Unlike routine safety assessments, SAEs are monitored continuously and have special reporting requirements

10.5 Exceptions from SAE Reporting

Progression of the underlying malignancy (including fatal outcome) and irAE will be collected on specific CRFs pages and should not be notified to the Sponsor as SAEs.

A study patient's relapse or progression or irAE will be documented on specific eCRFs pages and should not be communicated to the Sponsor as SAEs. Nevertheless, the investigator must complete the eCRF page designated for these events within 3 working days after knowledge.

10.6 Reporting of SAEs

Each AE must be assessed and recorded in eCRF. If an AE fulfills one or more of the seriousness criteria, a SAE form has to be filled out. The SAE information must contain at least the minimum criteria for a valid report:

- patient identification (patient number, sex and age).
- serious adverse event (description incl. onset date and outcome)
- relationship to IMP
- reporting source (investigator).

The investigator is also required to complete missing or requested information and to submit follow-up reports per fax to the number mentioned below until the SAE has resolved or, in the case of permanent impairment, until the SAE has stabilized. All documents must be blinded with respect to the patient's name. The confidentiality of patient information is ensured by revealing only a patient number and age.

10.6.1 Investigator requirements for SAE Reporting

All SAEs must be reported by fax to the following address within **24 hours** after knowledge by the investigator:

Pharmacovigilance Clinical Trials Unit
Medical Center - University of Freiburg
Elsaesser Str. 2, 79110 Freiburg

SAE Fax No.
+49 761 270-74 390

The investigator must complete the SAE-report form in English and sign it. The original SAE report form and the fax confirmation must be kept in the investigator site file.

According to section 12, subsection 6 GCP-V, in the event of the death of a patient the investigator must submit all information to the sponsor that put the sponsor in a position to

fulfill his duty in informing the competent ethics committee and the competent authority (note that personal data must not be transmitted using the trial-specific patient identification number, i.e. in pseudonymised form).
If only limited information is initially available, follow-up reports are required. (Note: Follow-up SAE reports are sent using a new SAE form. Follow-up should include the same investigator term(s) initially reported, if the terms has changed, please include the new term starting with "change of term:".)
If an ongoing SAE changes in its intensity or relationship to study drug or if new information becomes available, a follow-up SAE report should be sent within 24 hours using the same procedure used for transmitting the initial SAE report (details will be provided in SAE reporting Manual).
All SAEs should be followed to resolution or stabilization.

Patient's death:
Please note that "death" is usually an SAE outcome and not an SAE per se. Only in cases where the clinical circumstances before the death are unknown (i.e. patient died without a determinable cause of death), then the diagnosis "death" itself has to be reported as an SAE. In case of fatal outcome of an already-reported SAE, a follow-up notification must be done.
In case of patient's death the eCRF-pages designated for death documentation, end of treatment documentation and end of study documentation must be completed within 3 working days after knowledge.

10.6.2 Sponsor requirements for SAE reporting

The SAEs must be reported to the sponsor within the time period determined in the section 10.2.
The sponsor's reporting requirements are divided into **expedited** reporting and reporting that must be performed on request or annually. The sponsor's expedited reporting requirements comprise the following:

- All SUSARs must be reported within 15 days after knowledge (section 13, subsection 2 GCP-V),
- All SUSARs that are life-threatening or result in death must be reported within 7 days after knowledge (section 13, subsection 3 GCP-V),
- All circumstances requiring a review of the benefit/risk evaluation of ECP must be reported within 15 days after knowledge (e.g. expected serious adverse reaction with unexpected outcome, increased incidence of expected serious adverse reactions, SUSARs after the end of the patient's participation in the clinical trial, events in connection with the trial conduct which may affect the safety of the trial patients) (section 13, subsection 4 GCP-V).

SUSARs:
The sponsor's expedited reporting requirements are particularly relevant to suspected unexpected serious adverse reactions (SUSARs). The definition is a combination of the definitions of serious adverse reaction (adverse reaction that results in death, is life-threatening, requires inpatient hospitalization or prolongation of hospitalization, results in persistent or significant disability/incapacity, causes congenital anomaly/birth defect) and unexpected adverse reaction (an adverse reaction, the nature or severity of which is not consistent with the SmPC for methoxsalen).

SUSARs will be collected and reported by the sponsor to the competent authorities and relevant ethics committees in accordance with Directive 2001/20/EC.

10.7 Development safety update report (DSUR)

In addition to the expedited reporting, the Pharmacovigilance CTU shall submit, once a year throughout the whole duration of the clinical trial or on request a safety report to the Competent Authority and the Ethics Committee, according to protocol section 16.2, subsection 6 GCP-V and ICH guideline E2F. As this is an investigator initiated trial a study specific DSUR will be prepared. The aim of the DSUR is to concisely describe all relevant new safety information, to assess the safety conditions of subjects included in the concerned trial and to evaluate whether the benefit / risk ratio is still favorable.
To fulfill these requirements the investigator must complete the eCRF page designated for premature treatment/study discontinuation within 3 working days after knowledge. The DSUR will be written by the CTU and the study center in Freiburg.

10.8 Pregnancies

Any pregnancy that occurs during trial participation must be reported. To ensure patient safety each pregnancy must be reported to Sponsor on the pregnancy-reporting form within 24 hours of learning of its occurrence. The pregnancy should be followed up to determine outcome, including spontaneous or voluntary termination, details of birth, and the presence/absence of any birth defects, congenital abnormalities or maternal and newborn complications. Pregnancies that occur in a female partner of a study participant will also be reported. . All pregnancy reports must be reported by fax to the following address within **24 hours** after knowledge by the investigator:

Pharmacovigilance Clinical Trials Unit<br>Medical Center - University of Freiburg<br>Elsaesser Str. 2, 79110 Freiburg<br><br>**SAE Fax No.**<br>**+49 761 270-74 390**

10.9 Contraception

The following definitions are based on the recommendations of the Clinical Trial Facilitation Group (CTFG).
A woman is considered of childbearing potential, i.e. fertile, following menarche and until becoming post-menopausal unless permanently sterile. Permanent sterilization methods include hysterectomy, bilateral salpingectomy and bilateral oophorectomy. A postmenopausal state is defined as no menses for 12 months without an alternative medical cause.
Following birth control methods will be regarded as highly effective:

- combined (estrogen and progesterone containing) hormonal contraception associated with inhibition of ovulation (oral, intravaginal, transdermal)

- progesterone-only hormonal contraception associated with inhibition of ovulation (oral, injectable, implantable)
- intrauterine device
- intrauterine hormone-releasing system
- bilateral tubal occlusion
- vasectomized partner
- sexual abstinence

Methods of highly effective contraception should be used during the treatment with ECP until the end of relevant systemic exposure (5 months after the ECP procedure). Pregnancy tests will be conducted on a monthly basis during this period.
Males who are sexually active with WOCBP must use during the duration of the study and up to 5 months afterwards a latex or synthetic condom during any sexual contact with females of reproductive potential, even if they have undergone a successful vasectomy.
Patients must abstain from donating blood, semen, or sperm during participation in the study.

10.10 Administration setting

ECP will be performed in a specialized leukapheresis unit (outpatient facility). Specially-trained staff including physicians and study nurses will monitor the patient during ECP. Emergency medication and equipment will be available in the treatment room.

11 Documentation

11.1 Data handling and data management

11.1.1 Data confidentiality

Information about trial patients will be kept confidential and managed under the applicable laws and regulations. Those regulations require a signed patient authorization informing the patient of the following:

- what protected health information (PHI) will be collected from patients in this trial;
- who will have access to that information and why;
- who will use or disclose that information;
- right of trial patients to revoke their authorization for use of their PHI.

In the event that a patient revokes authorization to collect or use PHI, the investigator, by regulation, retains the ability to use all information collected prior to the revocation of patient authorization. For patients who have revoked authorization to collect or use their PHI, attempts should be made to obtain permission to collect at least their vital status (i.e. that the patient is alive) at the end of their scheduled trial phase.

The data collection system for this trial uses built-in security features to prevent unauthorized access to confidential participant information. Access to the system will be controlled by individually-assigned user identification codes and passwords made available only to authorized personnel who have completed prerequisite training.

11.2 Documentation of trial data

11.2.1 Documentation in medical records

The investigator will record participation in the trial, the frequency of trial visits, relevant medical data, concomitant treatment/s and the occurrence of adverse events in each trial patient's medical record.

11.2.2 Documentation in eCRF

The investigator or anyone designated by the investigator shall document the trial data on a trial-specific electronic case report form (eCRF) as promptly as possible. The investigator is responsible for ensuring the accuracy, completeness, and timeliness of the data reported to the sponsor in the eCRFs and in all required reports.

In order to identify authorized study personnel, a signature log is to be kept in the ISF and is to be forwarded to the sponsor for archival in the Trial Master File (TMF).

Accurate and reliable data collection will be assured by verification and cross–check of the eCRFs against the investigator's records by the study monitor (source data verification).

11.3 Data management

The data management will be performed with secuTrial® (https://www.secutrial.com/, interActive Systems GmbH). Details on data management (software, procedures, responsibilities, etc.) will be described in a data management manual prior to the trial. During the trial, the performance of data management and any deviations from the planned data management will be also documented in the data management manual. The technical specifications of the database and the structure of the e-forms (variable names, attributes, data entry and data validation checks) will be described in a corresponding database description plan.

Before any data entry is performed, the trial database and data checks of the e-forms will be validated. Data entry personnel will not be given access to the trial database until they have been trained and signed an access form.

An audit trail provides the history about data entered, changed or deleted, by whom and when. During data entry and study performance the data will be reviewed for completeness, consistency, plausibility, and regarding protocol violations and other distinctive problems (e.g., cumulative missings) using the Query Module of secuTrial® and secuTrial® Reports.

The resulting queries have to be corrected in the eCRF or verified by the investigator, respectively, in a timely manner.

All programs which can be used to influence the data or data quality will be validated (e.g., data validation programs, programs for eCRF/query tracking, programs for import of secuTrial® data into SAS, etc.).

11.3.1 Data coding

Concomitant treatments or procedures entered into the database will be coded using the WHO Drug Reference List, or the Anatomical Therapeutic Chemical classification system provided by the WHO.

All AEs or SAEs will be coded with MedDRA in its latest version.

11.4 Investigator Site File (ISF)

The investigator must maintain adequate and accurate records to enable the conduct of the study to be fully documented and the study data to be subsequently verified. These documents should be classified into two different separate categories:

- ISF
- Subject clinical source documents

The ISF will contain the protocol/amendments, Case Report and Query Forms, Ethics Committee and Competent Authority approval, sample informed consent, staff curriculum vitae and authorization forms and other appropriate documents / correspondence etc. During monitoring visits the ISF will be checked for completeness.

11.5 Source documents

The investigator shall supply the sponsor on request with any required background data from the study documentation or clinical records. This is particularly important when CRFs are illegible or when errors in data transcription are suspected. In case of special problems and/or governmental queries or requests for audit and inspections, it is also necessary to have access to the complete study records, provided that patient confidentiality is protected.
Source data as defined by ICH-GCP include original documents, data, and records such as hospital records, clinical and office charts, laboratory notes, memoranda, patients' diaries or evaluation checklists, pharmacy dispensing records, recorded data from automated instruments, copies or transcriptions certified after verification as being accurate copies, microfiches, photographic negatives, microfilm or magnetic media, X-rays, and records kept at the pharmacy, at the laboratories and at medico-technical departments involved in the clinical trial.

11.6 Archiving

11.6.1 Sponsor's Responsibility for Archiving

The Sponsor has to archive all essential documents of the clinical study after completion or discontinuation of the study for a minimum of 25 years in accordance with the legal regulations, including, but not limited to, TMF and CRF.

11.6.2 Investigator's Responsibility for Archiving

The Investigator must keep the ISF, CRFs and all information related to the clinical trial archived for at least 25 years after completion or discontinuation of the study. After that period of time the documents may be destroyed after Sponsors approval, subject to local regulations.

12 Quality Assurance

12.1 Monitoring

The monitoring is performed by Sektion Klinische Forschung, GCP und QM of the Department of Medicine 1, Medical Center - University of Freiburg. Adapted monitoring will be done according to ICH-GCP E6 and standard operating procedures (SOPs) to verify that patients' rights and wellbeing are protected, reported trial data are accurate, complete and verifiable from source documents and that the trial is conducted in compliance with the currently approved protocol/amendment, with GCP and with the applicable regulatory requirements to ensure safety and integrity of clinical trial data.
The investigator will accept monitoring visits before, during and after the clinical trial. Prior to the trial, a site initiation visits is conducted in order to train and introduce the investigators and their staff to the trial protocol, essential documents, handling of investigational product and related trial specific procedures, ICH-GCP and national/local regulatory requirements.
During the trial, the clinical research associate (CRA) will visit the site regularly during the trial depending on the recruitment rate and quality of data. During these on-site visits, the CRA verifies that the trial is conducted according to the trial protocol, trial specific procedures, ICH-GCP and national/local regulatory requirements. The presence of signed informed consents, eligibility of patients, primary endpoint and documentation/reporting of safety data (e.g. AE/SAE) will be verified by the CRA. The monitor is also performing source data verification to ensure clinical trial data are recorded and documented in the source data and CRFs are complete and accurate. Extent of source data verification (SDV) and monitor visit frequency will be adapted in case of lack of data quality or a high number of protocol violations. All trial specific monitoring procedures, monitoring visit frequency and extent of SDV will be predefined in a trial specific monitoring manual.

12.2 Audits and Inspections

As part of quality assurance according to GCP, the sponsor and the competent health authorities have the right to audit/inspect the trial sites and any other institutions involved in the trial. The aim of an audit/inspection is to verify the validity, accuracy and completeness of data, to establish the credibility of the clinical trial, and to check whether the trial subject's rights, safety and wellbeing are being maintained. The sponsor may assign these activities to persons otherwise not involved in the trial (auditors). These persons as well as inspectors are allowed access to all trial documentation (especially the trial protocol, CRFs, trial subjects' medical records, drug accountability documentation, and trial-related correspondence).
The sponsor and all trial sites involved undertake to support auditors and inspections by the competent authorities at all times and to allow the persons charged with these duties access to the necessary original documentation.
All persons conducting audits undertake to keep all trial subject data and other trial data confidential.

After each audit the investigator receives an audit confirmation from the responsible auditor. This confirmation has to be stored in the ISF in order to provide access to it in case of an inspection by the competent authorities. The audit report is provided to the sponsor for control. At the end of the study an audit certificate is added to the final report. A respective inspection report is provided to the trial centers for notification.

13 Biostatistical planning and analysis

All statistical programming for analysis will be performed with the Statistical Analysis System (SAS).

13.1 Trial design

For details on trial design see section of the protocol.

13.2 Objectives and endpoints

For details on endpoints see section 6 of the protocol.

13.3 Sample size estimation

As this is a phase 1/2 trial without the intention of a confirmatory proof of efficacy, no formal sample size calculation is performed. The initial determination to include 14 evaluable patients is mainly based on feasibility considerations. The usefulness of this sample size is assessed as follows: It is expected that only few SAEs will occur. If the probability of any ECP-treatment-related SAE for a patient is 20% (p=0.2), the probability to observe at least one patient with a treatment-related SAE is 0.956. In case probability of any ECP-related SAE for a patient is 15% (p=0.15), the probability to observe at least one patient with a treatment-related SAE is 0.897.
With an extended patient cohort of n=30 patients, a more precise estimation of AE-, SAE-rates and further endpoints is possible, and rare events can be detected with higher probability. In case that the probability of any ECP-treatment-related SAE for a patient is 10% (p=0.1), the probability to observe at least one patient with a treatment-related SAE is 0.958, and in case that the probability of any ECP-treatment-related SAE for a patient is 5% (p=0.05), the probability to observe at least one patient with a treatment-related SAE is 0.785.

13.4 Definition of analysis sets

Efficacy analyses will be performed in the full analysis set (FAS) according to the intention-to-treat (ITT) principle, safety analyses will be performed in the safety population SAF.
FAS and SAF are defined to consist of those patients for whom ECP treatment was started.

13.5 Statistical analysis

13.5.1 Primary endpoint analysis

The primary endpoint is to assess the safety of ECP in patients with immune-related colitis, pneumonitis, hepatitis, dermatitis. Rates of ECP-related AEs and SAEs will be calculated.

The trial will be considered as successful, if 50% or less of the patients experience an SAE. Feasibility aspects will however be taken into account.

13.5.2 Secondary endpoint analysis

Secondary endpoints are the ORR at 6 and 12 weeks after start of treatment. The ORR will be estimated as defined in section 6.2.1 and will be presented with a two sided 95% confidence interval. Futhermore, the time to response, the duration of response and the time between start of ECP and discontinuation of other immunosuppressive treatment will be assessed.
OS probabilities will be estimated and displayed using the Kaplan-Meier method. Other time-to-event endpoints where competing events have to be considered (time to treatment response, duration of response) are estimated as cumulative incidence rates using the Aalen Johanson estimator. Competing events are noted in section 6.1.
Changes in immune cell fehlertype, metabolism and serum pro-inflammatory markers will be analysed descriptively. The incidence of tumor progression or relapse will also be monitored as a secondary endpoint.

13.5.3 Patient demographics/other baseline characteristics

Demographic and other baseline data (including disease characteristics) will be summarized descriptively using the FAS.
Continuous data will be summarized by arithmetic mean, standard deviation, minimum, 25% quantile, median, 75% quantile, maximum, and the number of complete and missing observations. If appropriate, continuous variables can also be presented in categories.
Categorical data will be summarized by the total number of patients in each category and the number of missing values. Relative frequencies are displayed as valid % (number of patients divided by the number of patients with non-missing values).

13.5.4 Trial treatment

Duration of trial treatment exposure will be summarized. The number of patients with changes in treatment frequency/interruptions will be presented, along with reasons for the change in treatment frequency.

13.5.5 Concomitant medication

The concomitant medications will be summarized by ATC level 1/3/5. In each table, patients will be counted once, if they took at least one medication from the respective ATC level. The number of patients and the percentage of the total number of patients in the respective population will be given.

13.5.6 Safety analysis

Safety analysis will be performed for all patients for whom the treatment was started. All safety parameters will be listed by patient and center and displayed in summary tables.
The adverse events (AEs) are displayed in summary tables as follows:
The total number of AEs, the minimum, maximum and mean number of AEs per patient will be given.
The incidence of AEs defined by preferred term (PT) according to MedDRA will be calculated as the number of patients who experienced at least one AE with the respective PT in percentage of the total number of patients in the safety population. In the incidence tables the

PTs will be grouped by system organ class (SOC) according to MedDRA. Additionally, the incidence of AEs defined by SOC will be calculated as the number of patients who experienced at least one AE in the respective SOC as percentage of the total number of patients in the safety population.
Each table will be produced for the following AE-sets:

- all AEs
- AEs being at least severe
- Serious Adverse Events (SAEs)
- SAEs leading to death
- AEs possibly related to investigational product
- AEs possibly related to investigational product being at least severe
- SAEs possibly related to investigational product
- SAEs possibly related to investigational product leading to death

Incidences of AEs will be calculated with 95%-confidence intervals.
The frequency of laboratory abnormalities will be displayed by parameter.
Summary tables for vital signs will be produced by visit.

13.5.7 Interim analysis

One interim analysis with respect to safety will be performed after first 14 patients have completed their treatment phase or have reached month 3 after treatment start (whichever occurs first). The objective of this analysis is to enable early stopping of study treatment in case of an unacceptable rate of toxicities.

The following factors should be used for the decision with regard to the continuation of the study:

Incidence of SAE related to the investigational treatment in more than 50% of all patients.

This analysis will be performed in the safety population. The interim analysis and interim report will describe patient recruitment and safety for the patients in this period. Safety data will be discussed in a DMC Meeting

14 Data Monitoring Committee (DMC)

An independent Data Monitoring Committee (DMC) will be established. The members of the DMC are given in section “Responsibilities”. The function of the DMC is to monitor the course of the trial and if necessary to give a recommendation to the sponsor/coordinating investigator for discontinuation, modification or continuation of the trial. The underlying principles for the DMC are ethical and safety aspects for the patients. It is the task of the DMC to examine, whether the conduct of the trial is still ethically justifiable, whether security of the patients is ensured, and whether the process of the trial is acceptable.
The coordinating investigator will closely communicate with the DMC. The DMC will receive DSURs and all necessary data for safety review during the interim analysis. The first DMC meeting will be held at the latest to discuss the interim safety analysis. Further details on the DMC functioning will be described in the DMC charter.

15 Reporting

15.1 Final report

The Competent Authority and Ethics Committee will be informed within 90 days that the trial has officially ended.
Within one year of the completion of the trial, the Competent Authority and the Ethics Committee will be supplied with a summary of the final report on the clinical trial containing the principle results. The Coordinating Investigator is responsible for the generation of the final report.

15.2 Publication Policy

Any formal presentation or publication of data from this trial may be considered as a joint publication by the Principal Investigator and appropriate personnel who participated in data analysis and data collection of this trial. Publication of the results of the trial as a whole is intended. The trial will also be registered in the public register https://www.drks.de.Investigators participating in multicenter studies agree not to present data gathered from one center or a small group of centers before the main publication, unless formally agreed to by the Principal Investigator. Any published data will observe data protection legislation covering the trial subject and investigators.
Patient data will only be submitted within the DSGVO.
The sponsor must receive copies of any intended communication in advance of publication (at least 30 working days for an abstract or oral presentation and 45 working days for a journal submission). The sponsor will review the communications for accuracy (thus avoiding potential discrepancies with submissions to health authorities), verify that confidential information is not being inadvertently divulged and provide any relevant supplementary information.
The support by the Clinical Trials Unit Freiburg is to be mentioned in any publication. Clinical Trials Unit Freiburg-staff will be included as co-authors as applicable. A copy of all publications will be sent to the Clinical Trials Unit Freiburg.
By signing the contract to participate in this trial, the investigator declares that he or she agrees to submission of the results of this trial to national and international authorities for approval and surveillance purposes. At the same time, the investigator agrees that his or her name, address, qualifications and details of his or her involvement in the clinical trial may be made known to these bodies.

16 Ethical and regulatory aspects

16.1 Sponsor‘s and Investigator’s responsibilities

This clinical trial was designed, shall be implemented and reported in accordance with the ICH-GCP, with applicable local regulations (including European Directive 2001/20/EC), and with the ethical principles laid down in the Declaration of Helsinki.
Before initiating the clinical trial, the sponsor/coordinating investigator should submit the CTP and any required application(s) to the appropriate competent authority for review, acceptance, and/or permission, as required by the applicable regulatory requirements.
The protocol and the proposed informed consent form must be reviewed and approved by a properly constituted Independent Ethics Committee (IEC) before trial start. A signed and dated

statement that the protocol and informed consent have been approved by the IEC must be available prior to initiation of the trial.

Before the start of the trial, the investigator is required to sign a protocol signature page confirming his/her agreement to conduct the trial in accordance with these documents and all of the instructions and procedures found in this protocol and to give access to all relevant data and records to sponsor CRAs, auditors, sponsor Clinical Quality Assurance representatives, designated agents of sponsor, IECs and CA(s) as required.

16.2 Independent Ethics Committee and Health Authorities

Prior to the start of this study, the protocol, the proposed informed consent form and other information to subjects, must be reviewed by a properly constituted Ethics Committee and the Health Authority. A signed and dated statement that the protocol and informed consent have been approved by the Ethics Committee and Competent Authority must be obtained before study initiation. Any amendments to the protocol, other than administrative ones (for which the Ethics Committee and Competent Authority will merely be informed), must be reviewed and approved by the Ethics Committee and Health Authority.

Before inclusion of the first patient the investigator agrees that the federal state authorities will be informed about the study by the Clinical Trials Unit Freiburg. A copy of this report needs to be filed in the ISF and TMF.

16.3 Informed Consent

Before being admitted to the study, each patient will receive a comprehensive explanation of the proposed treatment including the nature and risks of the study, any known adverse experiences, the investigational nature of the study, and the other elements that are part of obtaining proper informed consent. Patient information will be performed in oral and written form. Patients will be allowed sufficient time to consider participation in the study, after having the nature and risks of the study explained to them.

It is the responsibility of the investigator, to obtain written informed consent from each subject participating in this study. After the subject has orally consented to participation in the trial, the witness' signature on the form will attest that the information in the consent form was accurately explained and understood. This informed consent should be given by means of a standard written statement, written in non-technical language. The investigator or designee must also explain that the subjects are completely free to refuse to enter the study or to withdraw from it at any time, for any reason. No subject can enter the study before his/her informed consent has been obtained. The date when informed consent was obtained must be documented in the patient's medical record and in the CRF. The informed consent form must be signed and dated by the patient and by the individual administering consent. One copy will be given to the patient and the original will be retained by the investigator as part of the study documentation. The investigator will not undertake any procedure specifically required for the clinical study until valid consent has been obtained.

If new safety information results in significant changes in the risk/benefit assessment, the consent form should be reviewed and updated if necessary. All subjects (including those already being treated) should be informed of the new information, given a copy of the revised form and give their consent to continue in the study.

The informed consent form is considered to be part of the protocol, and must be submitted by the sponsor together with the protocol for Ethics committee approval.

16.4 Data Privacy Protection and Confidentiality

The applicable local regulations of data privacy protection will be followed. The patients will be informed that any patient-related data and materials will be appropriately (pursuant to § 12 and § 13 of the GCP Regulations) made pseudonymous and that these data may be used for analysis and publication purposes. Furthermore, the patients will be informed that their pseudonymized data may be inspected by health authority inspectors or representatives of the sponsor or by MNK for the purpose of validation of a proper study conduct.
Patients who do not provide consent for transmission of their pseudonymized data will not be included into the clinical study.

16.5 Insurance

All trial subjects enrolled are insured against injury caused by their participation in the study in accordance with § 40 AMG under the group insurance contract of the Albert-Ludwigs-Universität Freiburg. The name of the insurance company:

HDI Global SE
Am Schönenkamp 45, 40599 Düsseldorf
Insurance Number: 57 010313 03014

16.6 Financial aspects of the study

Before the trial commences, the Sponsor (or its designee) and the Investigator (or the institution, as applicable) will agree on the costs necessary to perform the trial. This agreement will be documented in a financial agreement that will be signed by the Investigator (or the institution signatory) and the Sponsor (or its designee).

17 Protocol amendments or changes in trial conduct

In order to ensure comparable conditions in all trial centers and in the interest of standardized evaluations of the trial, changes in this protocol are not planned.
However changes in trial conduct are possible. Any change (besides administrative changes) to this protocol requires a written protocol amendment that must be reviewed by the sponsor and the Principal Investigator before implementation and consent needs to be obtained by the principal investigator of participating centers.
Amendments significantly affecting the safety of subjects, the scope of the investigation or the scientific quality of the study additionally require approval of the Ethics Committee and Health Authority. A copy of the written approval of these amendments must be provided to the sponsor and each investigator. Examples of amendments requiring such approval are:

- significant changes in the study design
- increases in the number of invasive procedures

These requirements for approval should in no way prevent any immediate action from being taken by the investigator or by the sponsor in the interests of preserving the safety of all patients included in the trial. If an immediate change to the protocol is felt to be necessary by the investigator and is implemented for safety reasons the sponsor must be notified and the Ethics Committee and Health Authority must be informed immediately. Amendments affecting only administrative aspects of the study do not require formal protocol amendments or Ethics Committee and Health Authority approval but the Ethics Committee and Health Authority must be kept informed of such administrative changes.

18 Appendices

Appendix 1 Grading of irAE according to CTCAE V5

Colitis/Diarrhea

**Grade**	**Symtpoms/Laboratory parameters**
1	<4 liquid stools per day over baseline, feeding well
2	4-6 stools liquid stools per day over baseline
3	Increase of ≥7 stools per day over baseline; incontinence; hospitalization indicated; severe increase in ostomy output compared to baseline; limiting self care Activities of Daily Living (ADL)
4	Life-threatening consequences; urgent intervention indicated

Hepatitis

Grade	Symtpoms/Laboratory parameters
1	ALT or AST > ULN - 3xULN if baseline was normal and baseline - 3x baseline if baseline was abnormal and/or total bilirubin >ULN to 1.5xULN
2	ALT or AST 3-5xULN if baseline was normal and 3-5x baseline if baseline was abnormal and/or total bilirubin >1.5 to 3xULN
3	ALT or AST 5-20xULN if baseline was normal and 5-20x baseline if baseline was abnormal and/or total bilirubin 3-10xULN OR symptomatic liver dysfunction, fibrosis by biopsy, compensated cirrhosis, and reactivation of chronic hepatitis
4	ALT or AST > 20xULN if baseline was normal and >20x baseline if baseline was abnormal and/or bilirubin >10xULN OR decompensated liver function

Pneumonitis

Grade	Symtpoms/Laboratory parameters
1	Radiographic changes only: ground glass change, non-specific interstitial pneumonitis confined to one lobe of the lung or <25% of lung parenchyma
2	Mild/moderate new symptoms: dyspnea, cough, involves more than one lobe of the lung or 25-50% of lung parenchyma
3	Severe new symptoms; Hospitalization required: involves all lung lobes or >50% of lung parenchyma, oxygen indicated
4	Life-threatening difficulty in breathing, ARDS

Dermatitis

Grade	Symtpoms/Laboratory parameters
1	Skin rash, with or without symptoms, < 10% BSA
2	Skin rash, with or without symptoms, covering 10-30% BSA
3	Rash covers > 30% BSA or grade 2 with moderate or severe symptoms
4	Severe consequences requiring hospitalization or urgent intervention indicated or life-threatening consequences

Appendix 2 Relevant laws, guidelines and links

Declaration of Helsinki	https://www.wma.net/policies-post/wma-declaration-of-helsinki-ethical-principles-for-medical-research-involving-human-subjects/
ICH E6 - GCP Guideline	https://www.ema.europa.eu/en/ich-e6-r2-good-clinical-practice
ICH E8 – General considerations for clinical trials	https://www.ema.europa.eu/en/ich-e8-general-considerations-clinical-studies
ICH E2F - DSUR	https://www.ema.europa.eu/en/ich-e2f-development-safety-update-report
EMA Guidelines	https://www.ema.europa.eu/en/human-regulatory/research-development/clinical-trials-human-medicines
AMG/GCP-V	http://www.gesetze-im-internet.de
Common Terminology Criteria for Adverse Events (CTCAE) version 5.0	https://ctep.cancer.gov/protocoldevelopment/electronic_applications/docs/CTCAE_v5_Quick_Reference_5x7.pdf
Recommendations related to contraception and pregnancy testing in clinical trials- Heads of Medicines Agencies (HMA)	https://www.hma.eu/fileadmin/dateien/Human_Medicines/01-About_HMA/Working_Groups/CTFG/2020_09_HMA_CTFG_Contraception_guidance_Version_1.1_updated.pdf
EORTC QLQ-C30 (version 3.0) (specimen)	https://www.eortc.be/qol/C30/QLQ-C30%20German.pdf

Appendix 3 EORTC QLQ – HDC29

EORTC QLQ – HDC29

Patienten berichten manchmal die nachfolgend beschriebenen Symptome oder Probleme. Bitte beschreiben Sie, wie stark Sie diese Symptome oder Probleme während der letzten Woche empfunden haben. Kreisen Sie bitte die Zahl, die am besten auf Sie zutrifft.

Während der letzten Woche:	Überhaupt nicht	Wenig	Ziemlich	Sehr
31. Hatten Sie Schmerzen im Mund?	1	2	3	4
32. Hatten Sie einen trockenen Mund?	1	2	3	4
33. Hatten Sie Schwierigkeiten beim Schlucken?	1	2	3	4
34. War Ihr Geschmacksempfinden beim Essen und Trinken verändert?	1	2	3	4
35. Hatten Sie Bauchschmerzen oder Bauchkrämpfe?	1	2	3	4
36. Hatten Sie Hautprobleme (z.B.: Jucken, Trockenheit)?	1	2	3	4
37. Haben Ihnen Haarprobleme infolge der Behandlung zu schaffen gemacht?	1	2	3	4
38. Haben Sie sich Sorgen darüber gemacht, dass Ihr Gewicht zu niedrig ist?	1	2	3	4
39. Hatten Sie Fieber oder Erkältungen?	1	2	3	4
40. Mussten Sie häufig Harn lassen?	1	2	3	4
41. Hatten Sie Schmerzen in den Knochen?	1	2	3	4
42. Hatten Sie Schwierigkeiten, Dinge zu beenden, die Sie angefangen hatten?	1	2	3	4
43. Haben Sie sich Sorgen wegen der Ergebnisse von Untersuchungen und Tests gemacht?	1	2	3	4

Bitte beantworten Sie folgende Fragen, wenn Sie sich gegenwärtig noch zur Behandlung im Krankenhaus befinden. Falls dies nicht der Fall ist, gehen Sie bitte zu Frage 48.

Während der letzten Woche:	Überhaupt nicht	Wenig	Ziemlich	Sehr
44. Hatten Sie Schwierigkeiten, mit dem Krankenhausaufenthalt zurechtzukommen?	1	2	3	4
45. Hat die Isolation im Krankenhaus Ihnen zu schaffen gemacht?	1	2	3	4
46. Waren Sie besorgt, daß Ihre Blutwerte sich nicht erholen könnten?	1	2	3	4
47. Wie zufrieden waren Sie mit der Vorbereitung auf Ihre Behandlung?	1	2	3	4

Bitte wenden

GERMAN

**Während der letzten <u>vier</u> Wochen:**	**Überhaupt nicht**	**Wenig**	**Ziemlich**	**Sehr**
48. Haben Sie sich von Ihren Nächsten (d.h. der Familie oder Freunden) isoliert gefühlt?	1	2	3	4
49. Haben Sie sich Sorgen gemacht, dass Ihr Familienleben durch Ihre Behandlung gestört wird?	1	2	3	4
50. Wie belastet waren Ihrer Meinung nach Ihre Nächsten durch Ihre Erkrankung und Behandlung?	1	2	3	4
51. Haben Sie es für notwendig befunden, Ihre Ängste / Sorgen vor Ihrer Familie bzw. Freunden zu verbergen?	1	2	3	4
52. Hat Ihre Erfahrung Ihnen geholfen, zwischen wichtigen und unwichtigen Dingen im Leben zu unterscheiden?	1	2	3	4
53. Waren Sie wegen Ihres zukünftigen Gesundheitszustandes besorgt?	1	2	3	4
54. Fühlten Sie sich weniger attraktiv aufgrund Ihrer Krankheit oder Behandlung?	1	2	3	4

Bitte kreuzen Sie das Kästchen an, wenn die folgenden Fragen nicht auf Sie zutreffen ☐

55. Haben Sie sich Sorgen darüber gemacht, ob Sie noch Kinder bekommen können?	1	2	3	4

Bitte beantworten sie die folgenden Fragen nur, wenn Ihre Krankenhausbehandlung abgeschlossen ist und Sie gegenwärtig zu Hause sind.

**Während der letzten <u>vier</u> Wochen:**	**Überhaupt nicht**	**Wenig**	**Ziemlich**	**Sehr**
56. Hat das regelmäßige Einnehmen Ihrer Medikamente Ihr tägliches Leben beeinträchtigt?	1	2	3	4
57. Haben Sie sich selbst genau beobachtet in Bezug auf irgendwelche neuen Symptome?	1	2	3	4
58. Hatten Sie weniger Interesse an Sexualität?	1	2	3	4
59. Konnten Sie Sexualität weniger genießen?	1	2	3	4

© Copyright 1994 EORTC Quality of Life Group, version 1.0 All rights reserved
