# Supplementary material for: Extracorporeal photopheresis for the treatment of steroid-refractory immune related adverse events from immune checkpoint inhibitor therapy: a multi-center phase Ib/II clinical trial
Source: Nat Commun. 2026 Oct 23;17:11078. doi: 10.1038/s41467-026-78897-9 (PMC13694692; doi:10.1038/s41467-026-78897-9)
Supplement: Supplementary file 2 [file 41467_2026_78897_MOESM2_ESM.pdf]

nature portfolio

Corresponding author(s): Robert Zeiser

Last updated by author(s): Sep 15, 2026

Reporting Summary

Nature Portfolio wishes to improve the reproducibility of the work that we publish. This form provides structure for consistency and transparency in reporting. For further information on Nature Portfolio policies, see our Editorial Policies and the Editorial Policy Checklist.

Statistics

For all statistical analyses, confirm that the following items are present in the figure legend, table legend, main text, or Methods section.

n/a	Confirmed	
☐	☒	The exact sample size (*n*) for each experimental group/condition, given as a discrete number and unit of measurement
☐	☒	A statement on whether measurements were taken from distinct samples or whether the same sample was measured repeatedly
☐	☒	The statistical test(s) used AND whether they are one- or two-sided<br>*Only common tests should be described solely by name; describe more complex techniques in the Methods section.*
☐	☒	A description of all covariates tested
☐	☒	A description of any assumptions or corrections, such as tests of normality and adjustment for multiple comparisons
☐	☒	A full description of the statistical parameters including central tendency (e.g. means) or other basic estimates (e.g. regression coefficient) AND variation (e.g. standard deviation) or associated estimates of uncertainty (e.g. confidence intervals)
☐	☒	For null hypothesis testing, the test statistic (e.g. *F*, *t*, *r*) with confidence intervals, effect sizes, degrees of freedom and *P* value noted<br>*Give P values as exact values whenever suitable.*
☒	☐	For Bayesian analysis, information on the choice of priors and Markov chain Monte Carlo settings
☒	☐	For hierarchical and complex designs, identification of the appropriate level for tests and full reporting of outcomes
☒	☐	Estimates of effect sizes (e.g. Cohen's *d*, Pearson's *r*), indicating how they were calculated

Our web collection on statistics for biologists contains articles on many of the points above.

Software and code

Policy information about availability of computer code

Data collection	We used the electronic case report form (eCRF) for data collection und secuTrial® software for data management.
Data analysis	Clinical data were managed and analyzed using Prism 10 (GraphPad), and SAS 9.4. The full analysis data set (FAS) and the safety set (SAF) included all the patients who received at least one administration of ECP. Descriptive statistics were employed for reporting continuous variables and frequency and percentages of discrete variables. Exact two-sided 95% confidence intervals for response rates were calculated, based on the binomial distribution. Overall survival (OS) was defined as time from start of treatment with ECP until death, treating observation times where the event of interest did not occur as censored. Analyses of OS was performed with the Kaplan Meier method. Time to response was assessed for responders only as days from initiation of ECP treatment until the first documentation of a reduction of at least one irAE grade of at least one organ manifestation. Duration of response (DOR) was calculated for responders only as time from first response to the first observation of irAE relapse/progression/death or the date of additional systemic treatment for irAE. DOR was analyzed with the Kaplan Meier method.<br>Flow cytometry and gene expression data were tested for normality applying the Shapiro-Wilk test. An unpaired or paired two-tailed Student's t-test was applied to compare two groups. If the data did not meet the criteria of normality, the Mann-Whitney test or Wilcoxon signed-rank test was applied for unpaired or paired analyses, respectively. In case multiple groups were compared, the Friedman test (paired data) with Dunn's multiple testing was chosen if the data did not meet the criteria of normality. Statistical differences between multiple groups of normally distributed data were analyzed using a repeated measures (paired data) one-way ANOVA with Tukey's multiple comparison test. The data was corrected for multiple testing.

For manuscripts utilizing custom algorithms or software that are central to the research but not yet described in published literature, software must be made available to editors and reviewers. We strongly encourage code deposition in a community repository (e.g. GitHub). See the Nature Portfolio guidelines for submitting code & software for further information.

Data

Policy information about availability of data

All manuscripts must include a data availability statement. This statement should provide the following information, where applicable:

- Accession codes, unique identifiers, or web links for publicly available datasets
- A description of any restrictions on data availability
- For clinical datasets or third party data, please ensure that the statement adheres to our policy

All data are included in the Supplementary Information or available from the authors, as are unique reagents used in this Article. The raw numbers for charts and graphs are available in the Source Data file whenever possible. The patient data cannot be placed in a public repository at this point but we will provide all flow-cytometry and image data upon request. This study does not report original code.

Research involving human participants, their data, or biological material

Policy information about studies with human participants or human data. See also policy information about sex, gender (identity/presentation), and sexual orientation and race, ethnicity and racism.

Reporting on sex and gender	Our study is focused on testing ECP as a novel treatment for immune checkpoint inhibitor induced irAE.<br>We have randomly included both sex, male and female, into the clinical trial and analyses of biological human material without any pre-selection. We did not see differences in the outcome between male and female participants.
Reporting on race, ethnicity, or other socially relevant groupings	We do not specifically report on race, ethnicity or other socially relevant grouping.
Population characteristics	Patients ≥18 years were eligible if they were diagnosed with either ICI-colitis, ICI-hepatitis, ICI-dermatitis or ICI-pneumonitis (or a combination of these) following anti-PD-1, anti-PD-L1, anti-CTLA-4 antibody or any combination of these as treatment for any malignancy in the last 24 months before screening. Exclusion of other possible causes for the symptoms were necessary. Further, these patients needed to be refractory to GC-treatment, defined as no improvement with regard to CTC-AE grade after 72 hours treatment with at least 1 mg/kg BW/day prednisolone equivalent with up to one additional second-line therapy after GC-treatment. The entire set of inclusion and exclusion criteria is provided in the study protocol (supplemental information or on https://www.clinicaltrialsregister.eu/ under EudraCT-No. 2021-002073-26).
Recruitment	Patients were recruited in the participating Cancer Centers. Inclusion and exclusion criteria were checked and patients received informed consent forms if considered eligible for the trial. The trial was performed in two recruitment phases. The initial study population was set to 14 included patients. Based on promising preliminary results the trial was granted an additional recruitment of 16 patients, which resulted in an overall recruitment of 30 patients. The trial was initiated on July 15th 2022, the first patient was included on August 1st 2022 and the last patient on September 2nd 2024.
Ethics oversight	All patients and healthy donors signed an informed consent form prior to inclusion into the study, and approval was obtained from the local ethics committees. The trial was designed and conducted in accordance with the guidelines for Good Clinical Practice of the International Council for Harmonisation, the principles of the Declaration of Helsinki, and local regulations. An independent data monitoring committee reviewed the safety data to ensure the safety of the therapy. Ethic committee approval numbers: 22-1052-AMG-ff multi; 23-1485-S1; Albert-Ludwigs-Universität Freiburg, Germany).<br>The trial was approved by the Federal Institute for Drugs and Medical Devices (BfArM) and corresponding Ethic Committees of the University of Freiburg, Germany 2022.

Note that full information on the approval of the study protocol must also be provided in the manuscript.

Field-specific reporting

Please select the one below that is the best fit for your research. If you are not sure, read the appropriate sections before making your selection.

[x] Life sciences [ ] Behavioural & social sciences [ ] Ecological, evolutionary & environmental sciences

For a reference copy of the document with all sections, see nature.com/documents/nr-reporting-summary-flat.pdf

Life sciences study design

All studies must disclose on these points even when the disclosure is negative.

Sample size	Prospective clinical study:<br>Safety: Adverse events and serious adverse events will be registered and reported according to the guideline of ICH/GCP. Rates of adverse events and of serious adverse events will be calculated with corresponding 95% confidence intervals.<br>Efficacy: The efficacy analyses will be based on the full analysis set (FAS) and will be considered as descriptive results.<br>Secondary endpoints: Descriptive analyses of the secondary endpoints will be performed. OS probabilities will be estimated and displayed using the Kaplan Meier method. The analysis of secondary time-to-event endpoints with competing risks will be performed by means of the Aalen Johanson estimator for the calculation of cumulative incidence rates.<br>As this is a phase 1/2 trial without the intention of a confirmatory proof of efficacy, no formal sample size calculation is performed. The determination to include 30 evaluable patients in the initial study population was mainly based on feasibility considerations. It is expected that

only few SAEs will occur. If the probability of any ECP-related SAE for a patient is 20% (p=0.2), the probability to observe at least one patient with a treatment-related SAE is 0.956. Based on promising preliminary results the trial was granted an additional recruitment of 16 patients, which resulted in an overall recruitment of 30 patients.

Data exclusions	No data were excluded from the analyses.
Replication	For replication purposes, samples analyses of biological specimen included all available samples. Changes of cell populations, phenotypes, gene expression and histological scoring were analyzed for all available samples. The experiments were not repeated individually.
Randomization	There was no randomization of patients or samples before analysis. Therefore, all available samples were included in our analysis. This was a single-arm study without randomization.
Blinding	The study treatment and the experiments were performed in a non-blinded manner.

Reporting for specific materials, systems and methods

We require information from authors about some types of materials, experimental systems and methods used in many studies. Here, indicate whether each material, system or method listed is relevant to your study. If you are not sure if a list item applies to your research, read the appropriate section before selecting a response.

Materials & experimental systems

n/a	Involved in the study	
☐	☒	Antibodies
☒	☐	Eukaryotic cell lines
☒	☐	Palaeontology and archaeology
☒	☐	Animals and other organisms
☐	☒	Clinical data
☒	☐	Dual use research of concern
☒	☐	Plants

Methods

n/a	Involved in the study	
☒	☐	ChIP-seq
☐	☒	Flow cytometry
☒	☐	MRI-based neuroimaging

Antibodies

Antibodies used

Antibodies used for FACS analysis of immune cells in patient peripheral blood included in the prospective phase Ib/II trial (EudraCT-No.: 2021-002073-26) – initial cohort.
Antigen Fluoro-chrome Isotype Clone Dilution Vendor Identifier
CD3 PE-Cy7 Mouse BALB/c IgG1, κ SK7 1:10 BD Biosciences Cat #341111; RRID:AB_2868767
CD4 FITC Mouse BALB/c IgG1, κ SK3 1:10 BD Biosciences Cat #345768; RRID:AB_2868797
CD8 APC Mouse BALB/c IgG1, κ SK1 1:10 BD Biosciences Cat #345775; RRID:AB_2868803
CD16 PE Mouse BALB/c IgG1, κ B73.1 1:10 BD Biosciences Cat #332779; RRID:AB_2868628
CD19 FITC Mouse IgG1, κ HIB19 1:10 BD Biosciences Cat #555412; RRID:AB_395812
CD56 PE Mouse BALB/c X C57BL/6 IgG1, κ MY31 1:10 BD Biosciences Cat #345810; RRID:AB_286883

Antibodies used for FACS analysis of immune cells in patient peripheral blood included in the prospective phase Ib/II trial (EudraCT-No.: 2021-002073-26) – extended cohort.
Antigen Fluoro-chrome Isotype Clone Dilution Vendor Identifier
CD3 FITC Mouse IgG1, κ SK7 1:10 BD Biosciences Cat #345763; RRID:AB_2811220
CD4 PE-Cy7 Mouse IgG1, κ SK3 1:10 BD Biosciences Cat #348809; RRID:AB_2783789
CD8 APC-Cy7 Mouse IgG1, κ SK1 1:10 BD Biosciences Cat #348813; RRID:AB_2868857
CD16 PE Mouse IgG1, κ B73.1 1:10 BD Biosciences Cat #332779; RRID:AB_2868628
CD19 APC Mouse IgG1, κ SJ25C1 1:10 BD Biosciences Cat #555415; RRID:AB_398597
CD56 PE Mouse IgG2b, κ NCAM16.2 1:10 BD Biosciences Cat #345812; RRID:AB_2629216

Antibodies used for immune cell phenotyping by FACS in irAE patient and healthy control peripheral blood samples.
Antigen Fluorochrome Isotype Clone Dilution Vendor Identifier
CD3 BV650 Mouse IgG1, κ UCHT1 1:25 BioLegend Cat #300468; RRID:AB_2629574
CD3 PE/Cy7 Mouse IgG1, κ SK7 1:25 BioLegend Cat #344816; RRID:AB_10640737
CD4 BV510 Mouse IgG1, κ SK3 1:25 BioLegend Cat #344634; RRID:AB_2566017
CD4 PerCP/Cy5.5 Mouse IgG2b, κ OKT4 1:25 BioLegend Cat# 317428; RRID:AB_1186122
CD8a AF488 Mouse IgG1, κ HIT8a 1:25 BioLegend Cat# 300916; RRID:AB_756152
CD8a APC Mouse IgG1, κ HIT8a 1:25 BioLegend Cat #300912; RRID:AB_314116
CD8 Pacific Blue Mouse IgG1, κ SK1 1:25 BioLegend Cat #344718; RRID:AB_10551438
CD11b Pacific Blue Rat IgG2b, κ M1/70 1:25 BioLegend Cat #101224; RRID:AB_755986
CD19 PE-Cy7 Mouse IgG1, κ HIB19 1:25 BioLegend Cat #302216; RRID:AB_314246
CD38 BV785 Mouse IgG1, κ HIT2 1:25 BioLegend Cat #303529; RRID:AB_2565893
CD45 FITC Mouse IgG1, κ HI30 1:25 BioLegend Cat #304006; RRID:AB_314394
Granzyme A FITC Mouse IgG1, κ CB9 1:25 BioLegend Cat #507204; RRID:AB_315470

	Granzyme B PE Rat IgG1, κ QA18A28 1:25 BioLegend Cat #396406; RRID:AB_2801075<br>Granzyme K PerCP/Cy5.5 Mouse IgG1, κ GM26E7 1:25 BioLegend Cat #370514; RRID:AB_2632852<br>HLA-DR BV711 Mouse IgG2a, κ L243 1:25 BioLegend Cat #307644; RRID:AB_2562913<br>IFN-γ BV510 Mouse IgG1, κ 4S.B3 1:25 BioLegend Cat #502544; RRID:AB_2563883<br>IFN-γ BV785 Mouse IgG1, κ 4S.B3 1:25 BioLegend Cat #502542; RRID:AB_2563882<br>Ki67 BV605 Mouse IgG1, κ Ki-67 1:25 BioLegend Cat #350522; RRID:AB_2563863<br>T-bet APC Mouse IgG1, κ 4B10 1:25 BioLegend Cat# 644814; RRID:AB_10901173<br>TNF-α PE Mouse IgG1, κ MAb11 1:25 BioLegend Cat #502909; RRID:AB_315261<br>Isotype Pacific Blue Rat IgG2b, κ RTK4530 1:25 BioLegend Cat #400627; RRID:AB_493561<br>Isotype PE Mouse IgG1, κ MOPC-21 1:25 BioLegend Cat #400112; RRID:AB_2847829<br>Isotype BV785 Mouse IgG1, κ MOPC-21 1:25 BioLegend Cat #400169; RRID:AB_11219601<br><br>Antibodies used for multiplex immunofluorescence staining.<br>CD3 Agilent Cat# IR50361-2; RRID:AB_3094578<br>CD4 (clone EPR6855) Abcam Cat# ab133616; RRID:AB_2750883<br>CD8 (clone C8/144B) Agilent Cat# M710301-2; RRID:AB_2075537<br>CD103 (ITGAE) Atlas Antibodies Cat# HPA036313; RRID:AB_10795355<br>Cytokeratin (clone AE1/AE3) Agilent Cat# IR05361-2; RRID:AB_2868599<br>Ki-67 (clone MIB-1) Agilent Cat# IR62661-2; RRID:AB_2890068
Validation	The antibodies were validated by titration and the use of fluorescence-minus-one (FMO) and/or isotype controls for a reliable identification of cell populations. Further, specific markers were analyzed in cells that cannot express these markers; signals were thereby validated to be correctly positive.

Clinical data

Policy information about clinical studies

All manuscripts should comply with the ICMJE guidelines for publication of clinical research and a completed CONSORT checklist must be included with all submissions.

Clinical trial registration	The trial was registered at European Union Drug Regulating Authorities Clinical Trials Database: EudraCT-No. 2021-002073-26
Study protocol	The full trial protocol of the phase Ib/II clinical trial is provided as a supplement or available online on https://www.clinicaltrialsregister.eu/ under the specific trial registration number (EudraCT-No. 2021-002073-26).
Data collection	The prospective phase Ib/II clinical trial (EudraCT-No.: 2021-002073-26) was registered on April 15th 2021, initiated on July 15th 2022 and the first patient was included on August 1st 2022. A copy of the IRB-approved study protocol of the trial is included in the supplemental material. The trial was approved by the local ethic committees of the participating centers and the Federal Institute for Drugs and Medical Devices (BfArM) in Germany. Ethics Committee Freiburg approval number 22-1052-AMG-ff multi; 23-1485-S1; Albert-Ludwigs-Universität Freiburg, Germany.<br>All the patients provided written informed consent before participating in the trial, and approval was obtained from the Ethics Committee of the University of Freiburg. The trial was designed and conducted in accordance with the guidelines for Good Clinical Practice of the International Council for Harmonisation, the principles of the Declaration of Helsinki, and local regulations. An independent data monitoring committee reviewed the safety data to ensure the safety of the therapy. The trial was designed by the investigators. Data were collected and analyzed by the investigators and their research staff.<br>Key eligibility criteria included a minimum age of 18 years and steroid-refractory irAEs affecting the intestinal tract, liver, skin or lungs. Patients were eligible if they had received treatment with an anti-PD1, anti-PD-L1 or an anti-CTLA4 antibody or any combination of these for any type of malignancy in the last 24 months before screening. Patients should have clinical and/or histological evidence of immune-related adverse events as follows:<br>Colitis: Diarrhea with increase of ≥4 stools over baseline; No improvement after 72h treatment with at least 1 mg/kg BW/day prednisolone equivalent;<br>Hepatitis: Alanine aminotransferase and/or aspartate aminotransferase ≥3x ULN if baseline was normal; or ≥3x baseline if baseline was abnormal; No improvement after 72h treatment with at least 1 mg/kg BW/day prednisolone equivalent;<br>Pneumonitis: Radiographic changes and new symptoms such as cough, dyspnea or chest pain; No improvement after 72h treatment with 1 mg/kg BW/day prednisolone equivalent;<br>Dermatitis: Skin erythema, maculopapular or pustulopapular rash covering ≥30% of the body surface area; No improvement after 72h treatment with at least 1 mg/kg BW/day prednisolone equivalent;<br>Maximum of one additional (second line) therapy after steroid treatment before ECP starts (e.g. infliximab for colitis).<br>An Eastern Cooperative Oncology Group (ECOG) performance status of 0 to 2.<br>Additional inclusion and exclusion criteria are listed in the study protocol that is provided in the Supplementary materials.<br>The severity of irAE was evaluated by a clinical assessment of the symptoms, and carried out before the initiation of ECP as well as at every patient visit after initiation of the therapy. irAE severity and response definitions were established based on the ASCO guidelines. Grading of irAE was performed according to the Common Terminology Criteria for Adverse Events (CTC-AE v5). Complete response (CR) was defined as the complete resolution of all irAE-related symptoms. Partial response (PR) was defined as the reduction of at least 1 grade according to CTC-AE in at least one irAE organ manifestation. Stable disease (SD) was defined as no change in the CTC-AE grades of all irAE. Progressive disease (PD) was defined as the increase of at least 1 grade according to CTC-AE in at least one irAE organ manifestation. CR, PR and PD were diagnosed only if the symptom severity remained stable for at least 5 consecutive days. The full analysis data set (FAS) and the safety set (SAF) included all the patients who received at least one administration of ECP. Descriptive statistics was employed for reporting continuous variables and frequency and percentages of discrete variables. Exact two-sided 95% confidence intervals for ORR were calculated, based on the binomial distribution. Overall survival (OS) and progression free survival (PFS) were defined as time from start of treatment with ECP until death (OS) / death or disease progression (PFS), treating observation times where the event of interest did not occur as censored. Analyses of OS and PFS

were performed with the Kaplan Meier method.

Outcomes

The primary endpoint was the rate of treatment-related adverse events (AEs) and serious adverse events (SAEs) in patients treated with ECP for immune-checkpoint inhibitor-induced colitis, pneumonitis, hepatitis or dermatitis. A positive result from the study is defined as ≤50% of the patients developing a treatment-related SAE. The key secondary endpoint was objective response rate (ORR) to treatment after 6 and 12 weeks of ECP-therapy. The response criteria for each organ are described in the study protocol. Additional secondary endpoints were time to response, discontinuation of other immunosuppressive therapies and the incidence of tumor progression or relapse. Additional endpoints are described in the study protocol.

Flow Cytometry

Plots

Confirm that:

- [x] The axis labels state the marker and fluorochrome used (e.g. CD4-FITC).
- [x] The axis scales are clearly visible. Include numbers along axes only for bottom left plot of group (a 'group' is an analysis of identical markers).
- [x] All plots are contour plots with outliers or pseudocolor plots.
- [x] A numerical value for number of cells or percentage (with statistics) is provided.

Methodology

Sample preparation

For analysis of lymphocyte subpopulations in patient peripheral blood, 50µl EDTA blood was incubated with 5 µl of the respective antibody for 30 min. 2.5 ml Lyse/Fix (BD Biosciences) was added and the samples were incubated for additional 10 minutes. The samples were centrifuged for 5 minutes (2000rpm) and the supernatant removed before resuspension in FACS Flow (BD Biosciences). Starting with the 12th patient, analysis of lymphocyte subpopulations was done according to manufacturer´s protocol (BD Biosciences) using the BD Multitest 6-color TBNK together with BD Trucount tubes. All data were measured with a BD FACS Canto II flow cytometer (BD Biosciences) and analyzed using the FACSDiva Software V6 (BD Biosciences). Blood samples were collected before and after the first ECP procedure, and at two variable timepoints during ECP-therapy.

For immune cell characterization in irAE patients and healthy donor samples, PBMCs were isolated by density-gradient separation (Lymphocyte separation medium, Anprotec) as described previously at screening before ECP-therapy, and weeks 6 and 12 of ECP-therapy 12. Isolated PBMCs were frozen in 90% FCS with 10% DMSO, and all samples were thawed and analyzed at the same time. Viability was analyzed by Zombie NIR fixable dye (BioLegend; 1:500 in PBS) staining, and Fc receptors were blocked using FcR blocking reagent (Miltenyi) for 10 min at RT. Surface markers were stained with fluorochrome conjugated antibodies for 30 min at 4°C. For the analysis of intracellular proteins, cells were fixed and permeabilized for 45 min at 4 °C using the Transcription Factor Staining Buffer Set (eBioscience) or the Fixation/ Permeabilization Kit (BD Biosciences) for staining of intranuclear or cytoplasmatic proteins and cytokines, respectively. For staining of TNF-α and IFN-γ, cells were stimulated in the presence of Cell Stimulation Cocktail with protein transport inhibitor (1:500; eBioscience) for 4 h at 37 °C before surface and intracellular cytokine staining. For the analysis of Granzymes, cells were incubated in the presence of GolgiPlug (1:1000; BD Biosciences) and GolgiStop (1:1500; BD Biosciences) for 4 h at 37 °C, before surface and intracellular staining. Stimulation and incubations with GolgiPlug/GolgiStop were done in RPMI 1640 medium (ThermoFisher Scientific) supplemented with 10 % FCS (Fetal Constance, Anprotec) and 1 % P/S (ThermoFisher Scientific). The samples were acquired using a BD LSR Fortessa equipped with four lasers, and analyzed using the FlowJo V10 software. The following panels were used for PBMC analysis:
Panel 1 (activated T cells and Th1): anti-CD45 FITC, anti-CD3 BV650, anti-CD8 PB, anti-CD4 BV510, anti-CD103 PE, anti-CD69 PE-Cy7, anti-CD38 BV785, anti-HLA-DR BV711, anti-KI67 BV605, anti-T-bet APC; Panel 2 (IFN-γ and TNF-α in T cells): anti-CD3 BV650, anti-CD8 FITC, anti-CD4 PerCP-Cy5.5, anti-CD38 BV785, anti-HLA-DR BV711, anti-TNF-α PE, anti-IFN-γ BV510, anti-Perforin PB, anti-Granulysin PE-Cy7, anti-KI67 BV605, anti-T-bet APC; Panel 3 (granzymes in T cells): anti-CD3 PE-Cy7, anti-CD8 APC, anti-CD4 BV510, anti-CD38 BV785, anti-HLA-DR PB, anti-Granzyme A FITC, anti-Granzyme B PE, anti-Granzyme K PerCP-Cy5.5, anti-KI67 BV605; Panel 4 (cytokines in myeloid cells): anti-CD45 FITC, anti-CD11b PB, anti-CD3 BV650, anti-CD4 BV510, anti-CD8 APC, anti-CD19 PE-Cy7, anti-TNF-α PE, anti-IFN-γ BV785, Rat IgG2bκ Isotype Ctrl Antibody (PB), Mouse IgG1κ Isotype Ctrl Antibody (PE), Mouse IgG1κ Isotype Ctrl Antibody (BV785).

Instrument

The data were acquired on a BD LSR Fortessa and a BD Canto II.

Software

The majority of data were analyzed using FlowJo (Flowjo 10.6, LLC) and FACSDiva V6 (BD Biosciences) softwares.

Cell population abundance

The gating strategy was determined using fluorescence minus one (FMO) controls and/or isotype controls.

Gating strategy

All experiments were performed using appropiate controls and compensation controls. Cells were first gated using SSC-A vs FSC-A, doublet cells were excluded by gating on FSC-H vs FSC-A, dead cells were excluded using Zombie NIR™ Fixable Viability Kit. Subsequently, the desired parameters were gated. Unstained controls, single stains, isotype controls and fluorescence minus one (FMO) controls were used for appropiate gating on desired parameters and differentiation between positive and negative cells. Myeloid cells were gated on CD3-CD19- cells to exclude the possibility for lymphocyte contamination. The gating strategies are provided as supplementary figures.

- [x] Tick this box to confirm that a figure exemplifying the gating strategy is provided in the Supplementary Information.
