# Supplementary material for: Extracorporeal photopheresis for the treatment of steroid-refractory immune related adverse events from immune checkpoint inhibitor therapy: a multi-center phase Ib/II clinical trial
Source: Nat Commun. 2026 Oct 23;17:11078. doi: 10.1038/s41467-026-78897-9 (PMC13694692; doi:10.1038/s41467-026-78897-9)
Supplement: Supplementary file 3 [file 41467_2026_78897_MOESM3_ESM.pdf]

nature portfolio

Peer Review File

Extracorporeal photopheresis for the treatment of steroid-refractory immune related adverse events from immune checkpoint inhibitor therapy: a multi-center phase Ib/II clinical trial

Corresponding Author: Professor Robert Zeiser

This file contains all reviewer reports in order by version, followed by all author rebuttals in order by version.

Version 0:

Reviewer comments:

Reviewer #1

(Remarks to the Author)
This paper reports impressively positive overall results in a prospective historically controlled clinical trial of immunomodulatory ECP (Extracorporeal Photopheresis) in the treatment of serious adverse reactions to checkpoint immunotherapy of a range of cancers. Since the problematic adverse reactions (largely hepatitis, colitis and dermatitis) in the selected subjects were all resistant resistant to conventional immunosuppressive intervention, the remarkable clinical reversal of the iatrogenic autoimmunity, as well as laboratory correlates, in nearly all enrolled patients is especially impressive. The near absence of adverse reactions to ECP itself and the fact that the experimental therapy did not suppress the clinical anti-tumor immunity importantly distinguishes ECP from standard immuno-suppresive alternatives. While earlier retrospective studies have inspired this study, these more definitive findings strongly support the potential application of ECP in this important way. In short, in my view, this paper reports a quite significant much needed advance.

Particularly because of my perception of the broad importance of this paper, I am concerned that the manuscript's very brief description of ECP, as a widely available and broadly applied therapy principally at university centers, and its established scientific basis may preclude those unfamiliar with the modality from fully appreciate and even understand the implications of the paper's message. For example, since ECP is available and in daily administration at the large majority of university medical centers in the USA and Europe for treatment of T cell lymphoma, organ transplant rejection and post-allo-transplant GVHD, its availability for potential application of the findings of this paper is fortuitous. Similarly, now that it is established that ECP's clinical efficacy reflects in large part its large scale generation of physiologic dendritic antigen presenting cells, it is now understood how ECP differs from other immunotherapies. The scant mention of these details in the manuscript should be succinctly expanded for a number of reasons that would strengthen the paper's impact. As a cellular immunotherapy dependent on the interplay of dendritic cells and anti-cancer T cells and anti-tissue T cells, ECP's capacity to itself enhance desirable anti-cancer responses, while at the same time, actively suppressive undesirable autoreactive T cells, may well explain major aspects of the treatment's benefit in the reported study. At the very least, this all deserves concise but emphatic mention.

Along these lines, it would be key to point out that the observed capacity of ECP to preserve the anti-tumor responses, while still suppressive the colitis, hepatitis and dermatitis precipitated by the checkpoint inhibitors, might be either passage or active. While this study cannot distinguish between these two possibilities, the especially exciting chance that ECP actually synergizes with the checkpoint therapy in the enhancement of anti-cancer immunity. Of course, since ECP is essentially an anti-cancer vaccination in cutaneous T cell lymphoma, and animal models of melanoma and other solid tumors, this is a possibility deserving of further attention and at least mention.

So, in short, I salute the authors for their major accomplishments and the high quality of this clinical trial. Indeed, I suspect that it may well be field-shaping. I, therefore, simply suggest
that the context in which these findings emerge be more firmly and clearly communicated.

Reviewer #2

(Remarks to the Author)
This manuscript by Riemer et al, reports the results of a multi-institutional single arm trial using ECP for the treatment of several immune-related adverse events from checkpoint blockade immunotherapy. The strength of this study is the novelty of the approach and the high clinical need. The weakness related to study design, outcomes reporting, and the clinical significance of the measures highlighted. My specific concerns are reported below.

1. There are multiple sources of treatment recommendations for ICI toxicities. ASCO and ESMO are among the most widely used, but I think that it is best not to imply that these are overriding guidelines, simply one of several frameworks that largely, but do not completely, overlap.
2. "Several studies report an inferior OS upon administration of an anti-TNFα antibody plus GCs compared to GCs alone for steroid refractory (SR)-irAEs." These are two studies from the same group looking at the same set of data, data that are confounded by the fact that patients who receive anti-TNF therapy are also the most likely patients to receive high dose and long-duration GC, since anti-TNF therapy is generally a second line agent. In the cited papers, attempts to fully control for this GC dose/duration effect actually lead to non-significance in the anti-TNF result. There are also multiple animal models showing a benefit to anti-TNF therapy in patients undergoing immunotherapy, which is worth noting given the weakness in the present human data.
3. "Infliximab and the gut selective anti-α4β7-integrin antibody vedolizumab are frequently used as second-line immunosuppressive therapies for ICI-colitis, yet literature shows variable response rates (29%-89%)." This statement is true, but oddly phrased. Not every patient responds to infliximab or vedolizumab, though the majority of data indicate a very high response rate to one or both treatments (much closer to the 89% number than the 29% number cited here).
4. Clinically the response rate and complete response rate at 1 week is more meaningful than at 6 and 12 weeks for high grade toxicities. The 1 week point is when additional treatments would be considered by most practicing clinicians. Waiting a full 6 weeks for a response would be an unusually long period of time for any toxicity that was not mild. The way that the data are reported makes it very difficult to determine the clinical utility of these findings.
5. At 6 weeks, I would be interested in determining the "steroid free" response rate. And also seeing how many patients were able to achieve a steroid-free response without requiring any other immune modulating medications.
6. The study allowed enrollment of patients after 3 days of a steroid non response, why was the median duration of steroids 4 weeks in the study?
7. The main figure and text should contain more information about second line therapies, and whether they were continued after initiation of ECP. These do represent a significant fraction of the total population in the study. I am also not clear why so many patients did not receive secondary immune suppression when guidelines clearly indicated that they should have received secondary immune suppression long before week 4 of GC non response.
8. It is not clear to me how many patients were biopsied in this colitis cohort at baseline? 20% of suspected colitis patients do not have colitis on biopsy, and approximately 10% of patients with hepatitis.
9. In a prospective study, why were steroid treatment courses only available in 22 of the 30 patients.
10. This study would be much more impactful if a steroid free remission rate were reported, even if this were provided as a posthoc analysis.
11. Since safety is the primary endpoint, this should be the focus in the abstract and in the results. The other results should be reported as secondary analyses later in the text. It would be valuable to know which of these secondary analyses were prespecified and which were posthoc clearly spelled out in the methods section and in the results.
12. Showing each colitis patient's CTCAE score in a separate subfigure is space consuming without really providing useful information. It would be much more useful to have a summary average of response.
13. In addition, a single case of improving endoscopic colitis is nice, but we need more information. How many cases were histologically confirmed? What fraction were rebiopsied? How many were still on steroids at the time of biopsy? We can infer this from the next figure, but this really should be how the data are describe at the outset. I presume that the other colitis patients were not histologically confirmed?
14. The colitis data are convincing that in the subset of examined patients, most saw an improvement in a variety of markers that have been previously determined to be important in ICI colitis. The overall limitation of the study still stands, though, which is that this is a single arm study where patients remained on steroids, so we have no way of knowing whether the changes observed in the colon were related to continued steroids use or were the result of the ECP. The fact that the patients had been on steroids before is also not sufficient evidence that the steroids were not the causative factor in the improvement, since we do not have a pre-steroid biopsy to compare to, and steroids could plausibly have an increasing effect over time, particularly as a large fraction of patients did not show complete resolution of their inflammation on immunologic phenotyping.
15. Several of the patients took several weeks to respond to ECP, which is also consistent with spontaneous resolution of the disease.
16. I would like to see examples of the primary flow cytometry, particularly for the IFNg producing macrophages along with appropriate negative controls. The Tbet staining is not convincing as is. It would be useful to include cells in the gate that are unable to produce Tbet so that we can set an appropriate positive cutoff.
17. The hepatitis data are, overall, not particularly convincing, given the length of time for some of the patients to respond, the fact that several patients had marginal declines, the absence of absolute values, and the use of a single marker for hepatitis (ALT). Transaminases can "burn out" after sufficient liver damage has occurred, for example. Knowing how many, and which patients had a biopsy confirmed diagnosis would also be helpful here, since highly refractory hepatitis is uncommon. It would also be helpful if we had more useful information on what cotreatments were given alongside ECP for each patient, what was done with the prednisone dose, and what the trends were for transaminases prior to the initiation of ECP (if available). As is, I would characterize this as promising, but not definitive, and would highlight the number of patients with clear resolution of hepatitis and the time to resolution in the data description.

18. If dermatitis is going to be included in the manuscript, some of the data should be in a main figure. As for now, Figure 5 does not optimally use its space. As with hepatitis, I think the percentage of patients achieving complete remission and the average duration of treatment to achieve remission are necessary to highlight in the main text along with other cotreatments.
19. I think that it is very promising that the number of patients with progressive disease did not increase with ECP. That said, the authors should be careful about reading too much into such a small study in the language that they use to describe these clearly preliminary results.
20. The ctDNA testing is a bit misleading as described. Most of the patients had undetectable or nearly undetectable disease at baseline. We can't say anything about these samples other than that their burden of disease remained undetectable throughout the study. Of the patients with evaluable disease, one progressed at the ctDNA level. Were any of these patients transitioned to targeted therapy during the study.
21. For safety, how can the authors be sure that the SAE were unrelated to ECP. Might they not be severe consequences of combining ECP with immune-related toxicities?
22. For the discussion, the authors need to tone down their conclusions. As this is an uncontrolled trial with no comparator arm and several methodological concerns as highlighted above. I think they can reasonably claim safety (their primary endpoint), no obvious negative impact on antitumor immunity (though a very small sample size with heterogenous tumors), and evidence of some efficacy in most patients. Clinically, complete responses are really the only meaningful ones for most of these toxicities, and that should be highlighted rather than the overall response rate. I also think that the characterization of alternative ICI colitis treatments does not reflect clinical reality. Mimi Wang's work (multiple publications over a number of years) nicely highlights the response to both infliximab and vedolizumab, which is typically complete, durable, and rapid. This is also the experience of every gastroenterologist I know who sees these patients clinically. Fewer than 5% of patients require treatment beyond steroids, infliximab and vedolizumab. That doesn't mean that there isn't plenty of room for novel therapies, particularly ones that avoid interfering with the cancer treatment, but ECP should be situated a more realistic framing of current clinical practice.

Version 1:

Reviewer comments:

Reviewer #1

(Remarks to the Author)
As Reviewer #1 on the original submission, I praised the significance of the authors' findings as potentially field-shaping in the evolving realm of cancer immunotherapy. They demonstrated the dual efficacy of ECP in replacing standard nonspecific corticosteroids in reversing severe adverse autoimmune reactions to immuno-stimulatory checkpoint inhibitors (CPIs), thereby releasing or even enhancing the desirable generation of anti-tumor immunity. The deficiency which I identified was that, by framing ECP so succinctly as merely an "immunomodulatory" agent, the authors inadvertently diminished the traction of their own message, since the audience at large could underestimate how logical basis of their findings and their potential importance. In their response to that critique and their modifications of the manuscript, the authors impressively and completely resolve that key deficiency. They highlight, with appropriate referencing, why ECP, through its generation of platelet-signaled conversion of monocytes to physiologic dendritic antigen-presenting cells, and resultant T cell responses, may be tailor-made to safely and simultaneously accomplish the above-mentioned goals. Since therapeutic use of CPIs, and other anti-cancer immunostimulants, can be limited by adverse induction of autoimmunity, ECP may offer a prototype scientifically based adjunctive therapy. Therefore, I am fully satisfied with the revised manuscript.

Reviewer #2

(Remarks to the Author)
I appreciate the care that the authors have taken in addressing my comments. I do still have several substantive concerns that I believe should be addressed prior to publication.

General comments
1. Throughout the text, the authors infer causality, for example with language such as "ECP reduces" or "ECP induces." This isn't really appropriate for a single arm study. Instead the authors should use language that emphasizes correlation "ECP is associated with" throughout the text. I also think that for conclusions based on combination treatment with steroids and ECP, the concluding or summarizing comments should read "Addition of ECP to systemic steroids…"
2. The discussion remains far too expansive in its conclusions with the tempering comments added as side notes to the end of overly ambitious interpretations of the data, rather than as replacements for these statements. I believe that the discussion should start with "In this single arm trial, we observed safety of ECP without an obvious negative impact on antitumor immunity, though these findings are limited by a small sample size with heterogenous tumors." A discussion of the safety data in more detail but toned down from the way it currently reads could then follow. After discussing the safety data, the authors could reasonable discuss the efficacy data, which should start with a summary "We found evidence of some efficacy in most patients treated…"

Minor comment
3. I still have several concerns with the flow cytometry data. My main concern is that "stain minus one" as a control is really not adequate for macrophages, which express high levels FcR, and an isotype control is really necessary. For the macrophage data, the authors should have gated on CD11b+CD3- cells to avoid the possibility of lymphocyte contamination, particularly as the CD11b stain does not appear to be very strong, and thus identifying a clear macrophage

population is not really possible. The gates are also drawn very tightly for Tbet and Ki67, clearly including some cells that are in the negative population. This likely would not change the overall conclusions, but I do think that the numbers reported are not accurate.

Comments on responses to prior review

Response to point 5.
I do think that these numbers belong in the main text, as they are more easily compared with standard practice metrics. I also think that "intention to treat" is really the correct population to display, so the 3 patients who did not fully complete treatment should be included in the response rate.

Response to point 6.
The literature cited to support the 4 week duration is old in a rapidly advancing field. Contemporary treatment guidance at most high volume centers (including the centers responsible for several of the cited papers) is to start secondary immune suppression at day 3 for non responders to steroids and within a week for partial responders. In the cohorts highlighted, most of these patients were on steroids for a prolonged period of time precisely because the treating physician believed that the steroids were having an effect, even if the grade of diarrhea had not yet changed. The 4 week duration of steroids pre-secondary intervention makes it very difficult to compare ECP to standard of care therapies (anti-TNF or anti-integrin biologics). That doesn't negate the findings of this study, but it does temper my enthusiasm for a potential clinical impact.

Response to point 7.
For colitis, I am still confused why so few patients on this study received standard second or third line therapy. The problem with this, from my perspective, is that in a non randomized study where we are also not privy to the total number of eligible patients at the participating centers, it is likely that a group of people who are initially treated very differently from standard practice were treated differently for a biologically meaningful reason, even if we do not know what that reason was.

Response to point 8.
I am surprised that so few patients were considered at high risk for a biopsy. The risk of flexible sigmoidoscopy with biopsy is miniscule (estimated at 0.02%, much lower than the risk of an incorrect diagnosis which is closer to 20% in this population). I do think that this is a significant limitation because of my concern raised in the above point (#7). Perhaps this was a cohort that had a substantial number of patients where the diagnosis of colitis was incorrect to begin with.

Response to point 10.
As mentioned above, patients who die during the study or who prematurely exit the study should be included in the steroid free remission rate. They did not have a remission, maybe for no reason related to ECP or maybe because of ECP. Removing them isn't really appropriate because it makes the response rate look higher by removing treated patients who did not improve. If this was the prespecified analysis, removing these patients, then I think a posthoc analysis that does include them should be within the main text.

Response to point 11.
The primary endpoint needs to be reported first, both in the abstract and in the results. It is the primary endpoint of the study. Key secondary endpoints that are prespecified are important, but they are not the primary endpoint of the study, and by placing them first it could lead less careful readers to be confused about how the study was designed. This is, ultimately, a safety study.

Response to point 12.
I agree with the authors, the summary data is useful, but so is the individual data and the choice to include it as a supplement was a good one.

Response to point 14.
I agree with the authors that the study design is appropriate for a phase I/II study with safety as the primary endpoint. My concern is that the way that the paper is written and the data are presented, it is not sufficiently emphasized that all of the efficacy inferences are ultimately exploratory and require confirmation in an appropriately designed trial (as is planned). I think that this emphasis needs to appear repeatedly throughout the text, because the authors should be weary of encouraging use of an unproven therapy prior to completion of the phase 2 study based on data that, while intriguing, clearly does not meet the clinical bar for demonstrated efficacy.

Response to point 15.
I disagree with the authors here. The fact that steroids were continued leaves them as a reasonable contributing factor, particularly as we would expect ~50% of patients to eventually respond to steroids over a 6 week period (which is what published data suggest, and guidelines recommend 6-8 week steroid courses). Because a response to steroids remains plausible, and no control group is included, the correlate data are ultimately irrelevant – they only demonstrate that patients did get better, not why they did. In my mind, this concern is further increased by the factors highlighted above that make me concerned that this cohort is skewed toward a group that may be more likely to respond to steroids late – i.e. absence of universal biopsies, prolonged period of time on steroids without the initiation of secondary immune suppression.

Response to point 17.
B – you are correct about the ASCO and ESMO guidelines, though that does not change the fact that 10% of patients with suspected ICI hepatitis do not, in fact, have evidence of ICI hepatitis on biopsy. This is likely a higher fraction of patients who

do not respond to steroids in my clinical experience (PMID: 34554206). This is why other guidelines, for example GI society guidelines, look more favorably on biopsy.
F – it would be useful to include the percentage of patients with stable transaminases prior to initiation of steroids in the main figure.
G – the time to response remains long enough for the steroids to be a major contributing factor. This does need to be acknowledged in the discussion.

Response to point 20.
A – I actually think that the authors need to state that the cDNA testing was non diagnostic, and remained undetectable throughout the study in nearly all patients.
It is important to emphasize that we cannot conclude anything about cancer related safety from these data one way or the other. It is possible that every patient's cancer got worse going from 1000 fold below the limit of detection to 10 fold below the limit of detection. It is also possible that none of these patients had any cancer at all at the time of the study. The opposite is also true of course, maybe everyone's cancer burden was improving throughout the study. Since we have no way of knowing, the data really have to be characterized as non diagnostic.
B – the fact that 40% of these patients received an alternative cancer treatment is also important to emphasize, since this could mask an effect on antitumor immunity.

Response to point 21.
I think that this explanation is very thorough. Since this is a safety study, I believe that essentially this entire text belongs in the main text of the article.

Version 2:

Reviewer comments:

Reviewer #2

(Remarks to the Author)
The authors have essentially addressed all of my concerns and I want to thank them for their substantial efforts in this additional revision. I have two small, but I think still important final comments.

1. In the discussion, the authors write "Monitoring of ctDNA in the serum of melanoma patients harboring BRAFV600E or NRASQ61R mutations revealed stable or decreasing levels in 9/10 patients during ECP, suggesting that ECP did not lead to a loss of anti-tumor immune responses." This statement is not really supported by the data. The data show that most patients had undetectable ctDNA levels at all points throughout the analysis, making it impossible to make any conclusion about the effect on antitumor immunity from the available data.

2. The final paragraph remains too expansive. "In summary, our study extends prior clinical results on ECP as an immunomodulatory approach to control ICI-colitis, ICI-hepatitis and ICI-dermatitis, providing longitudinal intestinal gene expression analysis under a therapeutic intervention. Based on the observation that ECP specifically targets ICI-induced inflammation without affecting anti-tumor immunity, addition of ECP to systemic steroids is likely to meet the unmet clinical need for a therapeutic approach that reduces ICI-induced irAEs but allows for immunological tumor control."

This sentence implied that the authors clearly demonstrated that ECP controlled inflammation in ICI colitis, hepatitis, and dermatitis, which is not the case in this study. The authors showed that ECP was safe, and showed some evidence supporting a beneficial treatment effect for these inflammatory syndromes. In addition, the statement "ECP specifically targets ICI-induced inflammation without affecting anti-tumor immunity" is not clearly established by this study. This is what the authors hope to show in a more definitive, randomized, ideally placebo-controlled study. Similarly, I think that saying that this approach "is likely to meet the unmet clinical need..." is premature. The authors certainly have established sufficient evidence to justify further clinical testing.

Open Access This Peer Review File is licensed under a Creative Commons Attribution 4.0 International License, which permits use, sharing, adaptation, distribution and reproduction in any medium or format, as long as you give appropriate credit to the original author(s) and the source, provide a link to the Creative Commons license, and indicate if changes were made.

In cases where reviewers are anonymous, credit should be given to 'Anonymous Referee' and the source.

The images or other third party material in this Peer Review File are included in the article's Creative Commons license, unless indicated otherwise in a credit line to the material. If material is not included in the article's Creative Commons license and your intended use is not permitted by statutory regulation or exceeds the permitted use, you will need to obtain permission directly from the copyright holder.

To view a copy of this license, visit https://creativecommons.org/licenses/by/4.0/

Response to Reviewer's comments:

Reviewer #1 (Remarks to the Author)

This paper reports impressively positive overall results in a prospective historically controlled clinical trial of immunomodulatory ECP (Extracorporeal Photopheresis) in the treatment of serious adverse reactions to checkpoint immunotherapy of a range of cancers. Since the

problematic adverse reactions (largely hepatitis, colitis and dermatitis) in the selected subjects were all resistant to conventional immunosuppressive intervention, the remarkable clinical reversal of the iatrogenic autoimmunity, as well as laboratory correlates, in nearly all enrolled patients is especially impressive. The near absence of adverse reactions to ECP itself and the fact that the experimental therapy did not suppress the clinical anti-tumor immunity importantly distinguishes ECP from standard immuno-suppresive alternatives. While earlier retrospective studies have inspired this study, these more definitive findings strongly support the potential application of ECP in this important way. In short, in my view, this paper reports a quite significant much needed advance.

Response: We would like to thank the reviewer for this positive evaluation of our study and for highlighting that ECP may be a much needed advance in the field of immune-related adverse events following ICI therapy.

Particularly because of my perception of the broad importance of this paper, I am concerned that the manuscript's very brief description of ECP, as a widely available and broadly applied therapy principally at university centers, and its established scientific basis may preclude those unfamiliar with the modality from fully appreciate and even understand the implications of the paper's message. For example, since ECP is available and in daily administration at the large majority of university medical centers in the USA and Europe for treatment of T cell lymphoma, organ transplant rejection and post-allo-transplant GVHD, its availability for potential application of the findings of this paper is fortuitous. Similarly, now that it is established that ECP's clinical efficacy reflects in large part its large scale generation of physiologic dendritic antigen presenting cells, it is now understood how ECP differs from other immunotherapies. The scant mention of these details in the manuscript should be succinctly expanded for a number of reasons that would strengthen the paper's impact. As a cellular immunotherapy dependent on the interplay of dendritic cells and anti-cancer T cells and anti-tissue T cells, ECP's capacity to itself enhance desirable anti-cancer responses, while at the same time, actively suppressive undesirable autoreactive T cells, may well explain major aspects of the treatment's benefit in the reported study. At the very least, this all deserves concise but emphatic mention.

Response: We agree that ECP as a modality may need a better description to include also readers who may not be familiar with the therapy. We agree that this would make the study more understandable for many readers.

In the revised version, we have included a broader description of ECP as a therapeutic modality in the introduction of the manuscript (page 5).

We state that ECP is available and in daily administration at the large majority of university medical centers in the US and Europe for treatment of T cell lymphoma, organ transplant rejection and GVHD.

Further, we would like to thank the reviewer for mentioning that the generation of physiologic dendritic cells may be one of the reasons why ECP suppressed tissue-reactive T cells while preserving anti-tumor activities.

We have now included a description of the mechanisms of action of ECP including the generation of physiologic dendritic cells.

We cite:

Berger C, Hoffmann K, Vasquez JG, Mane S, Lewis J, Filler R, Lin A, Zhao H, Durazzo T, Baird A, Lin W, Foss F, Christensen I, Girardi M, Tigelaar R, Edelson R. Rapid generation of maturationally synchronized human dendritic cells: contribution to the clinical efficacy of extracorporeal photochemotherapy. Blood. 2010 Dec 2;116(23):4838-47. doi: 10.1182/blood-2009-11-256040.

Along these lines, it would be key to point out that the observed capacity of ECP to preserve the anti-tumor responses, while still suppressive the colitis, hepatitis and dermatitis precipitated by the checkpoint inhibitors, might be either passage or active. While this study cannot distinguish between these two possibilities, the especially exciting chance that ECP actually synergizes with the checkpoint therapy in the enhancement of anti-cancer immunity. Of course, since ECP is essentially an anti-cancer vaccination in cutaneous T cell lymphoma, and animal models of melanoma and other solid tumors, this is a possibility deserving of further attention and at least mention.

Response: We thank the reviewer for pointing out this particularly important aspect on the mechanism of action of ECP and its role in sustaining anti-tumor immunity. In the revised version, we are discussing available literature in more depth and cite the respective studies evaluating ECP as an anti-cancer vaccination strategy.

We now state:

DCs assist the immune response as key professional antigen-presenting cells, processing and presenting antigens to T cells. The capacity of ECP to promote this differentiation and to generate antigen-specific immunity supports the concept that ECP also contributes to anti-tumor immunity.

The following literature is supporting this concept:

Ventura A, Vassall A, Robinson E, Filler R, Hanlon D, Meeth K, Ezaldein H, Girardi M, Sobolev O, Bosenberg MW, Edelson RL. Extracorporeal Photochemotherapy Drives Monocyte-to-Dendritic Cell Maturation to Induce Anticancer Immunity. Cancer Res. 2018 Jul 15;78(14):4045-4058. doi: 10.1158/0008-5472.CAN-18-0171.

Girardi M, Berger C, Hanlon D, Edelson RL. Efficient tumor antigen loading of dendritic antigen presenting cells by transimmunization. Technol Cancer Res Treat. 2002 Feb;1(1):65-9. doi: 10.1177/153303460200100109. PMID: 12614179.

Kibbi N, Sobolev O, Girardi M, Edelson RL. Induction of anti-tumor CD8 T cell responses by experimental ECP-induced human dendritic antigen presenting cells. Transfus Apher Sci. 2016 Aug;55(1):146-52. doi: 10.1016/j.transci.2016.06.001.

Tatsuno K, Yamazaki T, Hanlon D, Han P, Robinson E, Sobolev O, Yurter A, Rivera-Molina F, Arshad N, Edelson RL, Galluzzi L. Extracorporeal photochemotherapy induces bona fide immunogenic cell death. Cell Death Dis. 2019 Aug 2;10(8):578. doi: 10.1038/s41419-019-1819-3.

So, in short, I salute the authors for their major accomplishments and the high quality of this clinical trial. Indeed, I suspect that it may well be field-shaping. I, therefore, simply suggest that the context in which these findings emerge be more firmly and clearly communicated.

Response: We thank the reviewer for the overall positive evaluation of our clinical trial and highlighting that the findings may bring changes to the field. We follow the reviewer's advice i) to describe the modality itself more clearly, ii) to discuss potential modes of action in more depth, iii) and to improve the clarity of the communication of our findings.

Reviewer #2 (Remarks to the Author)

This manuscript by Riemer et al, reports the results of a multi-institutional single arm trial using ECP for the treatment of several immune-related adverse events from checkpoint blockade immunotherapy. The strength of this study is the novelty of the approach and the high clinical need. The weakness related to study design, outcomes reporting, and the clinical significance of the measures highlighted. My specific concerns are reported below.

Response: We thank the reviewer for the thoughtful and constructive assessment of our manuscript. We appreciate the recognition of the novelty and clinical relevance of our study. Below, we respond to each point in detail and have revised the manuscript accordingly to

address concerns on study design, outcomes reporting, and the clinical significance of the measures.

1. There are multiple sources of treatment recommendations for ICI toxicities. ASCO and ESMO are among the most widely used, but I think that it is best not to imply that these are overriding guidelines, simply one of several frameworks that largely, but do not completely, overlap.

Response: We thank the reviewer for this important clarification. We agree that the ESMO and ASCO clinical practice guidelines are widely used frameworks for managing ICI toxicities, but they are not the only available sources of guidance and should not be portrayed as overriding recommendations. As recommended we have revised the manuscript to make clear that these guidelines are among several approaches that largely, though not completely, overlap.

We now include this statement in the introduction:

ESMO and ASCO clinical practice guidelines are widely used frameworks for managing ICI toxicities, but they are not the only available sources of guidance.

2. “Several studies report an inferior OS upon administration of an anti-TNFα antibody plus GCs compared to GCs alone for steroid refractory (SR)-irAEs.” These are two studies from the same group looking at the same set of data, data that are confounded by the fact that patients who receive anti-TNF therapy are also the most likely patients to receive high dose and long-duration GC, since anti-TNF therapy is generally a second line agent. In the cited papers, attempts to fully control for this GC dose/duration effect actually lead to non-significance in the anti-TNF result. There are also multiple animal models showing a benefit to anti-TNF therapy in patients undergoing immunotherapy, which is worth noting given the weakness in the present human data.

Response: We thank the reviewer for this important comment and appreciate the reviewer´s perspective on the confounding factors inherent in retrospective clinical analysis we had cited. We agree that the available clinical data are confounded by indication, since anti-TNF therapy is generally reserved for patients with more severe steroid-refractory irAEs who are also more likely to receive prolonged and high-dose corticosteroids.

We now include the following statement in the introduction after the statement on TNF:

The relationship between TNF blockade and clinical outcomes is likely influenced by highly context-dependent variables that warrant further investigation. The currently available literature is not definitive.

3. “Infliximab and the gut selective anti-α4β7-integrin antibody vedolizumab are frequently used as second-line immunosuppressive therapies for ICI-colitis, yet literature shows variable response rates (29%-89%).” This statement is true, but oddly phrased. Not every patient responds to infliximab or vedolizumab, though the majority of data indicate a very high response rate to one or both treatments (much closer to the 89% number than the 29% number cited here).

Response: We agree that the reported response rates in the literature vary widely due to heterogeneity in study populations and definitions of 'response', but we acknowledge that the consensus in the field points toward high response rates for both infliximab and vedolizumab, generally at the higher end of the range we cited.

We now include correct our statement to:

Infliximab and the gut selective anti-α4β7-integrin antibody vedolizumab are frequently used as second-line immunosuppressive therapies for ICI-colitis with reported response rates up to 89%.

Our study intends to describe the response rates to ECP and we do not wish to criticize other reports on irAE responses to infliximab and vedolizumab. Our own prior work has contributed to a report showing that vedolizumab is high effective in reducing intestinal acute GVHD:

Chen YB, Mohty M, **Zeiser R**, Teshima T, Jamy O, Maertens J, Purtill D, Chen J, Cao H, Rossiter G, Jansson J, Fløisand Y. Vedolizumab for the prevention of intestinal acute GVHD after allogeneic hematopoietic stem cell transplantation: a randomized phase 3 trial. ***Nature Medicine***. 2024 Aug;30(8):2277-2287. doi: 10.1038/s41591-024-03016-4.

4. Clinically the response rate and complete response rate at 1 week is more meaningful than at 6 and 12 weeks for high grade toxicities. The 1 week point is when additional treatments would be considered by most practicing clinicians. Waiting a full 6 weeks for a response would be an unusually long period of time for any toxicity that was not mild. The way that the data are reported makes it very difficult to determine the clinical utility of these findings.

Response: We agree that it is important to also report responses at week 1. We would like to make the reviewer aware of the fact that this was only after two ECP sessions given in one week.

We observed an overall response rate (ORR) of 63.3% at one week. The novel data is shown in Suppl. Figure 1B and reported in the results part. This was done as a post-hoc analysis to answer the comment by this reviewer. One patient exhibited an increase in colitis irAE grade

after one ECP session (Suppl. Fig. 1B). However, this patient achieved a complete remission (CR) after 4 ECP sessions, which lasted until end of study (12 months after start of ECP). We believe that the long-term responses at weeks 6 and 12 are also clinically meaningful because it supports a lasting effect of the therapy. The ORR endpoints week 6 and week 12 were also accepted by the FDA and EMA for the randomized follow-up study as a secondary endpoint (week 6) and as primary endpoint (week 12).

5.

A: At 6 weeks, I would be interested in determining the "steroid free" response rate.

B: And also seeing how many patients were able to achieve a steroid-free response without requiring any other immune modulating medications.

Response:

A: *"At 6 weeks, I would be interested in determining the "steroid free" response rate."*

We agree and now provide the "steroid free" response rate at weeks 6 and 12.

Week 6: The steroid free response rate at week 6 was 46.2% (12 / 26 patients). The 14 responder patients (53.8%) that were still on steroids at week 6 had a median steroid dose of only 8.75mg/d prednisolone equivalent. A recent trial reported that high corticosteroid dose was associated with worse PFS after ICI therapy: Verheijden RJ, de Groot JS, Fabriek BO, Hew MN, May AM, Suijkerbuijk KPM. Corticosteroids for Immune-Related Adverse Events and Checkpoint Inhibitor Efficacy: Analysis of Six Clinical Trials. J Clin Oncol. 2024 Nov;42(31):3713-3724. doi: 10.1200/JCO.24.00191.

The daily dose of 8.75mg/d prednisolone equivalent reported in our patients is below the reported peak doses that increase the risk for relapse.

Week 12: 23 / 26 patients (88.5%) were steroid free responders at week 12. The 3 patients (11.5%) that were still on steroids at week 12 had a median steroid dose of only 5 mg/d prednisolone equivalent.

26/29 (89.7%) of responders could be analyzed for steroid free response rate at week 6 and 12. 3 patients could not be analyzed due to premature EOS (n=2) and premature EOT (n=1).

The new data were included in the results part of the manuscript.

B: *"And also seeing how many patients were able to achieve a steroid-free response without requiring any other immune modulating medications."*

28/30 patients had no other immune modulating medications at any time after ECP was started. Two patients had simultaneous MMF started before ECP. During ECP the dose of MMF in these 2 patients was reduced and in 1 patient discontinued completely.
The new data on the duration of the second line treatment was included in main Figure 2H.

In the revised manuscript we now emphasize the fact, that response rates were achieved without the additional need of other immune modulating medications in 28/30 patients (results part).

6. The study allowed enrollment of patients after 3 days of a steroid non response, why was the median duration of steroids 4 weeks in the study?
Response: The minimum requirement for study inclusion was 3 days of a steroid non response as recommended in the ESMO/ASCO guidelines. There was no maximum duration of steroid treatment prior to ECP start, as it is known from the literature that most patients are treated much longer than 3 days before a second line therapy is started.

This study reported that the median duration of steroid treatment for immune checkpoint inhibitor (ICI)-induced colitis in their patient population is **64 days**:
Burla J, Bluemel S, Biedermann L, Barysch MJ, Dummer R, Levesque MP, Gubler C, Morell B, Rogler G, Scharl M. Retrospective Analysis of Treatment and Complications of Immune Checkpoint Inhibitor-Associated Colitis: Histological Ulcerations as Potential Predictor for a Steroid-Refractory Disease Course. Inflamm Intest Dis. 2020 Aug;5(3):109-116. doi: 10.1159/000507579.
See Table 3.

Another study reported a mean length of steroid treatment of **59 days** - see Table 3 in:
Wang Y, Abu-Sbeih H, Mao E, Ali N, Ali FS, Qiao W, Lum P, Raju G, Shuttlesworth G, Stroehlein J, Diab A. Immune-checkpoint inhibitor-induced diarrhea and colitis in patients with advanced malignancies: retrospective review at MD Anderson. J Immunother Cancer. 2018 May 11;6(1):37. doi: 10.1186/s40425-018-0346-6.

30 patients received steroids for more than **6 weeks** prior to second line:
Abu-Sbeih H, Ali FS, Wang X, Mallepally N, Chen E, Altan M, Bresalier RS, Charabaty A, Dadu R, Jazaeri A, Lashner B, Wang Y. Early introduction of selective immunosuppressive therapy associated with favorable clinical outcomes in patients with immune checkpoint inhibitor-induced colitis. J Immunother Cancer. 2019 Apr 2;7(1):93. doi: 10.1186/s40425-019-0577-1.

Therefore, the median duration of 4 weeks (30 days) of corticosteroid treatment is within the expected range reported by multiple other groups.

Additionally, the fact that the patients included in our study received a median duration of 4 weeks of corticosteroids indicates that highly refractory irAEs were treated, for which the chance for a late response to steroids was very low.

We now discuss the literature on median corticosteroid treatment in the discussion part. We point out that corticosteroid treatment in our population was not unusually long but within the range reported in the literature.

Discussion: The median duration of 4 weeks of corticosteroid treatment of the study patients prior to ECP start is within the expected range reported by multiple other groups. The reported median duration of steroid treatment for ICI-induced colitis is 64 days, 59 days or 6 weeks (References).

7. The main figure and text should contain more information about second line therapies, and whether they were continued after initiation of ECP. These do represent a significant fraction of the total population in the study. I am also not clear why so many patients did not receive secondary immune suppression when guidelines clearly indicated that they should have received secondary immune suppression long before week 4 of GC non response.

Response: We agree and now include a new graph on the second line therapies. Only 2/30 patients (6.6%) received a second line immunosuppressant (MMF) during ECP. In both patients the MMF could be tapered. Two patients (2/30) had received a second line immunosuppressant prior to ECP (MMF, infliximab) without clinical improvement and then received ECP and responded.

In the initial version of the manuscript, these data had been shown in Suppl. Table 1 and mentioned in the results part. The new data on the duration of the second line treatment was included in main Figure 2H.

8. It is not clear to me how many patients were biopsied in this colitis cohort at baseline? 20% of suspected colitis patients do not have colitis on biopsy, and approximately 10% of patients with hepatitis.

Response: In 11/14 (78.6%) patients with any grade of colitis the ICI-colitis was histologically confirmed at diagnosis.

We now provide this information in a new **Suppl. Table 2**:

Colitis (clinical, CTC-AE)	Number of patients / all colitis patients	Number of patients with a biopsy **before** ECP (%) (Baseline)	Number of patients with a biopsy **before and after** ECP (%)	Histological grading provided **before and after** ECP (%)
G1	1	1/1 (100.0%)	1/1 (100.0%)	1/1 (100.0%)
G2	7	6/7 (85.7%)	4/7 (57.1%)	4/7 (57.1%)
G3	3	2/3 (66.7%)	2/3 (66.7%)	2/3 (66.7%)
G4	3	2/3 (66.7%)	2/3 (66.7%)	1/3 (33.3%)

4/6 patients (66.7%) with severe colitis (grade 3 and 4) were biopsied at baseline and after ECP. One patient with grade 4 colitis did not receive a colonoscopy because the risk for perforation was considered to be too high. One patient with colitis grade 2 and one patient with colitis grade 3 did not receive biopsy before ECP. The risk to injure the patient with the biopsy was considered higher than the benefit of histological confirmation of colitis. This is now explained in more detail in the results part and Suppl Table 2 is included.

9. In a prospective study, why were steroid treatment courses only available in 22 of the 30 patients.

Response: The prespecified endpoint was Corticosteroid treatment at week 0 and week 12. For this endpoint the data of 25 / 30 (83.3%) patients are available as shown in Figure 2F. For 5 patients these data are not available because: n=2 did not reach week 12 and had premature end of study (EOS) due to death because of tumor progression, n=1 premature end of treatment because of complete remission of irAEs, 2 patients were steroid free in week 0 of ECP start after having a steroid treatment before ECP started.

We now show as a post-hoc analysis the comparison of week -1 versus week 12 in Suppl. Figure 1D which includes 27/30 patients.

We explain the reasons why data are not available at week 12 in 5/30 patients in more detail in the results part of the revised the manuscript.

It is not unexpected that 2 / 30 patients with advanced tumors died from tumor progression within the 12 weeks period.

The detailed steroid course on multiple time points in between week 0 and week 12 in 22 patients is displayed in Fig. 1G. This was a post-hoc analysis in which data was not available in 8 / 30 patients.

10. This study would be much more impactful if a steroid free remission rate were reported, even if this were provided as a posthoc analysis.
Response: We agree with the reviewer and now report **the steroid-free remission** rate as a post-hoc analysis.

Week 6: The steroid free response rate at week 6 was 46.2% (12 / 26 patients). The 14 responder patients (53.8%) that were still on steroids at week 6 had a median steroid dose of only 8.75mg/d prednisolone equivalent. A recent trial reported that high corticosteroid dose was associated with worse PFS after ICI therapy: Verheijden RJ, de Groot JS, Fabriek BO, Hew MN, May AM, Suijkerbuijk KPM. Corticosteroids for Immune-Related Adverse Events and Checkpoint Inhibitor Efficacy: Analysis of Six Clinical Trials. J Clin Oncol. 2024 Nov;42(31):3713-3724. doi: 10.1200/JCO.24.00191.
The daily dose of 8.75mg/d prednisolone equivalent reported in our patients at week 6 is below the reported peak doses that increase the risk for relapse.

Week 12: 23 / 26 patients (88.5%) were steroid free responders at week 12. The 3 patients (11.5%) that were still on steroids at week 12 had a median steroid dose of only 5 mg/d prednisolone equivalent.

26/29 (89.7%) of responders could be analyzed for steroid free response rate at week 6 and 12. 3 patients could not be analyzed due to premature EOS (n=2) and premature EOT (n=1).

The new data were included in the results part of the manuscript.

11.
A: Since safety is the primary endpoint, this should be the focus in the abstract and in the results. The other results should be reported as secondary analyses later in the text.
B: It would be valuable to know which of these secondary analyses were prespecified and which were posthoc clearly spelled out in the methods section and in the results.
Response:

A: *“Since safety is the primary endpoint, this should be the focus in the abstract and in the results. The other results should be reported as secondary analyses later in the text.”*

We appreciate the reviewer’s comment regarding the emphasis on safety versus efficacy in the abstract and results. We now report safety before efficacy in the abstract. However, the key secondary endpoint of this study was to evaluate the efficacy of ECP as prespecified in the study protocol. For this reason, we chose to present efficacy findings first in the results part, as they reflect the main therapeutic question under investigation, followed by a comprehensive description of safety outcomes. Safety of ECP was high which is consistent with prior reports by others on ECP used for example for GVHD.

B: *“It would be valuable to know which of these secondary analyses were prespecified and which were posthoc clearly spelled out in the methods section and in the results.”*

We have revised the Methods section to clearly state which efficacy and safety analyses were prespecified in the study protocol and which were conducted post-hoc.

The study protocol names as Key secondary endpoints (see submitted study protocol):

- To evaluate the investigator-assessed objective response rate (ORR) to treatment after 6 and 12 weeks of ECP therapy. The response criteria for each organ are described in chapter 6.2.
- To assess time to response
- To assess the duration of investigator-assessed clinical response
- To assess overall survival (OS) at 1 year after end of ECP treatment
- To measure the time to complete discontinuation of other immunosuppressive therapy
- To assess changes in immune cell phenotype and metabolism during treatment
- To assess the relapse rates of irAEs upon discontinuation of ECP and rechallenge with an immune checkpoint inhibitor
- To assess the incidence of tumor progression or relapse

The analysis of steroid intake at multiple time points between d0, week 6 and 12 was a post-hoc analysis and is therefore not as complete as the prespecified endpoints. We now include this information in the manuscript.

12. Showing each colitis patient’s CTCAE score in a separate subfigure is space consuming without really providing useful information. It would be much more useful to have a summary average of response.

Response: We agree and follow the reviewer's suggestion on the presentation of individual colitis patient's CTCAE scores. We have now included a summary graph that reports the CTCAE grades over time in all patients in the Figure 2J.
We have moved the individual-patient graphs to the Suppl. Figure 1E-R. We chose to display each colitis patient's CTC-AE course in the revised Suppl. Figure 1E-R to explicitly illustrate the heterogeneity and temporal dynamics of immune-related colitis in this cohort.

13. In addition, a single case of improving endoscopic colitis is nice, but we need more information.
A: How many cases were histologically confirmed?
B: What fraction were rebiopsied?
C: How many were still on steroids at the time of biopsy? We can infer this from the next figure, but this really should be how the data are describe at the outset.
D: I presume that the other colitis patients were not histologically confirmed?
Response:
A: *"How many cases were histologically confirmed?"*
In 11/14 cases (78.6%) the ICI-colitis was histologically confirmed at diagnosis.
We now provide this information in a new **Suppl. Table 2**:

Colitis (clinical, CTC-AE)	Number of patients / all colitis patients (%)	Number of patients with a biopsy **before** ECP (%)	Number of patients with a biopsy **before and after** ECP (%)	Histological grading provided **before and after** ECP (%)
G1	1	1/1 (100.0%)	1/1 (100.0%)	1/1 (100.0%)
G2	7	6/7 (85.7%)	4/7 (57.1%)	4/7 (57.1%)
G3	3	2/3 (66.7%)	2/3 (66.7%)	2/3 (66.7%)
G4	3	2/3 (66.7%)	2/3 (66.7%)	1/3 (33.3%)

B: *"What fraction were rebiopsied?"*
This information is now shown in Suppl. Table 2.
4/6 patients (66.7%) with severe colitis (grade 3 and 4) were biopsied at baseline and after ECP. 1 patient with grade 4 colitis did not receive a colonoscopy because the risk for perforation was considered to be too high. One patient with colitis grade 2 and one patient with

colitis grade 3 did not receive a biopsy before ECP. The risk to injure the patient with the biopsy was considered higher than the benefit of histological confirmation.
This is now explained in more detail in the results part and Suppl Table 2 is included. In 13 of the 14 (92.9%) patients ICI-colitis was the leading irAE. In one patient with ICI-colitis (irAE grade 1) ICI-dermatitis was more severe and reported as leading irAE.

C: *"How many were still on steroids at the time of biopsy? We can infer this from the next figure, but this really should be how the data are describe at the outset."*
At initial biopsy 100% were on steroids. At re-biopsy, 3/9 patients (33.3%) were still on steroids.

D: *"I presume that the other colitis patients were not histologically confirmed?"*
Correct – in the other patients (3/14, 21.4%) the diagnosis of ICI-colitis was based on clinical assessment. The ESMO guidelines recommend biopsy if the clinical risk is acceptable. In the patients that did not receive a biopsy comorbidity or the clinical risk for bleeding or intestinal perforation was considered to be too high.

14. The colitis data are convincing that in the subset of examined patients, most saw an improvement in a variety of markers that have been previously determined to be important in ICI colitis.
A: The overall limitation of the study still stands, though, which is that this is a single arm study where patients remained on steroids, so we have no way of knowing whether the changes observed in the colon were related to continued steroids use or were the result of the ECP.
B: The fact that the patients had been on steroids before is also not sufficient evidence that the steroids were not the causative factor in the improvement, since we do not have a pre-steroid biopsy to compare to, and steroids could plausibly have an increasing effect over time, particularly as a large fraction of patients did not show complete resolution of their inflammation on immunologic phenotyping.
Response:
We thank the reviewer for stating that our colitis data are convincing.

A: *"The overall limitation of the study still stands, though, which is that this is a single arm study where patients remained on steroids, so we have no way of knowing whether the changes observed in the colon were related to continued steroids use or were the result of the ECP."*
A phase Ib/II study has normally a single arm design and not a randomization against other therapies. This comparison comes later in the planned randomized trial.

B: *"The fact that the patients had been on steroids before is also not sufficient evidence that the steroids were not the causative factor in the improvement, since we do not have a pre-steroid biopsy to compare to, and steroids could plausibly have an increasing effect over time, particularly as a large fraction of patients did not show complete resolution of their inflammation on immunologic phenotyping."*

Two points speak against a late response to corticosteroids:

The patients had been on steroids for a median time of 4 weeks without clinical response. Therefore, it is unlikely that this steroid therapy is the causative factor for the later clinical response. Secondly, the steroids were tapered in all patients after ECP start as shown in Figure 2F.

Nevertheless, we follow this reviewer's thought and discuss that in some patients we cannot exclude a steroid effect that contributed to the response.

We include this in the discussion:

Although the pre-treatment with GC prior to ECP was 4 weeks, in some patients the corticosteroids given as first line therapy could have contributed to the clinical response under ECP.

15. Several of the patients took several weeks to respond to ECP, which is also consistent with spontaneous resolution of the disease.

Response: Response to ECP was fast in 63% of the patients which experienced a response already in week 1 after 2x ECP. All patients had already been exposed to prolonged courses of systemic corticosteroids without meaningful or sustained improvement, which makes a purely spontaneous resolution of their irAE less likely.

In addition to the clinical responses, we observed a decrease of pathogenic T cells and cytokines in the intestinal biopsies of ICI-colitis patients as shown in Figures 3 and 4.

Taken together the clinical responses, decrease in histological disease scores and decrease of pathogenic T cells, serum calprotectin and pro-inflammatory cytokines supports an ECP treatment-related effect.

16.

A: I would like to see examples of the primary flow cytometry, particularly for the IFNg producing macrophages along with appropriate negative controls.

B: The Tbet staining is not convincing as is. It would be useful to include cells in the gate that are unable to produce Tbet so that we can set an appropriate positive cutoff.

Response:

A: *"I would like to see examples of the primary flow cytometry, particularly for the IFNg producing macrophages along with appropriate negative controls."*

Response: We now provide primary flow plots for all findings in the revised version of the manuscript. In particular, we have now added the appropriate negative control (fluorescence-minus-one, FMO) for the analysis of IFN-γ in myeloid cells ($CD11b^{+}$). This was now added to the figure displaying the gating strategy. The primary flow cytometry plots, gating strategies and controls were included in main Figure 4 and the Suppl. Figures 3, 4, 8, 9, 10, 11.

***B:** "The Tbet staining is not convincing as is. It would be useful to include cells in the gate that are unable to produce Tbet so that we can set an appropriate positive cutoff."*

To control for the setting of gates, we had included fluorescence-minus-one (FMO) controls in the analysis which is according to standard analysis for flow cytometry.

To confirm that the gate for Tbet was correctly set, we now additionally provide a separate analysis in the Suppl. Figure 8B showing Tbet gating in CD3 negative cells in our analyzed PBMCs. These CD3 negative cells are unable to produce Tbet and can be used as negative control because Tbet is a T cell intrinsic transcription factor.

Tbet expression in CD3 negative cells overlaps with the FMO control. This additional control confirms that the gate for Tbet was correctly set.

The new data are shown in Suppl. Figure 8B.

17.

A: The hepatitis data are, overall, not particularly convincing, given the length of time for some of the patients to respond, the fact that several patients had marginal declines, the absence of absolute values, and the use of a single marker for hepatitis (ALT). Transaminases can "burn out" after sufficient liver damage has occurred, for example.

B: Knowing how many, and which patients had a biopsy confirmed diagnosis would also be helpful here, since highly refractory hepatitis is uncommon.

C: It would also be helpful if we had more useful information on what cotreatments were given alongside ECP for each patient, (**D**) what was done with the prednisone dose, and (**F**) what the trends were for transaminases prior to the initiation of ECP (if available).

(**G**) As is, I would characterize this as promising, but not definitive, and would highlight the number of patients with clear resolution of hepatitis and the time to resolution in the data description.

Response:

***A:** "The hepatitis data are, overall, not particularly convincing, given the length of time for some of the patients to respond, the fact that several patients had marginal declines, the absence of absolute values, and the use of a single marker for hepatitis (ALT). Transaminases can "burn out" after sufficient liver damage has occurred, for example."*

We appreciate the reviewer's critical assessment of the hepatitis data and address this in the revised manuscript version. We did not measure only ALT but also AST in all patients. However, we chose to present ALT in the initial version of the manuscript as our primary marker because it is more liver-specific than AST. In contrast AST is also present in other tissues (heart, muscles, and kidneys) and therefore less specific for hepatic inflammation. ALT is one of the markers for ICI-hepatitis recommended by the ASCO guidelines, page 4082 (Schneider et al JCO 2021) and ESMO guidelines, page 1222 (Haanen et al. Annals of Oncology 2023). Bilirubin is not considered as a liver cell damage marker as it increased upon cholestasis which can be due to liver metastases, drug toxicity and others.
We now also present the data on AST and bilirubin in all patients in the Suppl. Figure 5A-C.

B: "*Knowing how many, and which patients had a biopsy confirmed diagnosis would also be helpful here, since highly refractory hepatitis is uncommon.*"

Response: Only two patients in this cohort underwent liver biopsy; for all others, hepatitis was defined biochemically, which is in agreement with the recommendations in the ESMO and ASCO guidelines. See below:

ASCO guidelines, page 4082 (Schneider et al JCO 2021): Liver biopsy should be considered **if there is a concern** for other differential diagnoses that would alter medical management.

ESMO guidelines, page 1222 (Haanen et al. Annals of Oncology 2023): Liver biopsy **may assist** in the differential diagnosis of more severe hepatitis.
We had ruled out other causes for increased liver values including for example metastases, virus hepatitis, alcohol and others. All patients were discussed in the Board for immune related adverse events at Freiburg University Medical Center or Essen Medical Center. We now include this information in the revised manuscript.

C: "*It would also be helpful if we had more useful information on what cotreatments were given alongside ECP for each patient.*"

Response: We agree that cotreatment should be described more explicitly. No cotreatment beyond steroids was given alongside ECP in 15/17 (88.2%) ICI-hepatitis patient. The cotreatment with MMF in one hepatitis patients was short (4 weeks and then stopped) and the duration is displayed in Figure 2H. The other patient had MMF which was reduced from 1000 mg twice daily to 500 mg thrice daily.

D: "*what was done with the prednisone dose.*"

Response: We now provide the prednisone dose selectively for the hepatitis patients as requested by this reviewer.

Please find below the graph showing time courses of steroid reduction (% from baseline) over time in 14 patients with ICI-hepatitis. 3 patients were not included in the graph due to confounding factors (n=1 additional dexamethasone therapy for 3 days per cycle chemotherapy as antiemetic therapy, n=1 premature EOS, n=1 high dose dexamethasone therapy for brain metastases and perifocal edema resulting in premature EOS)

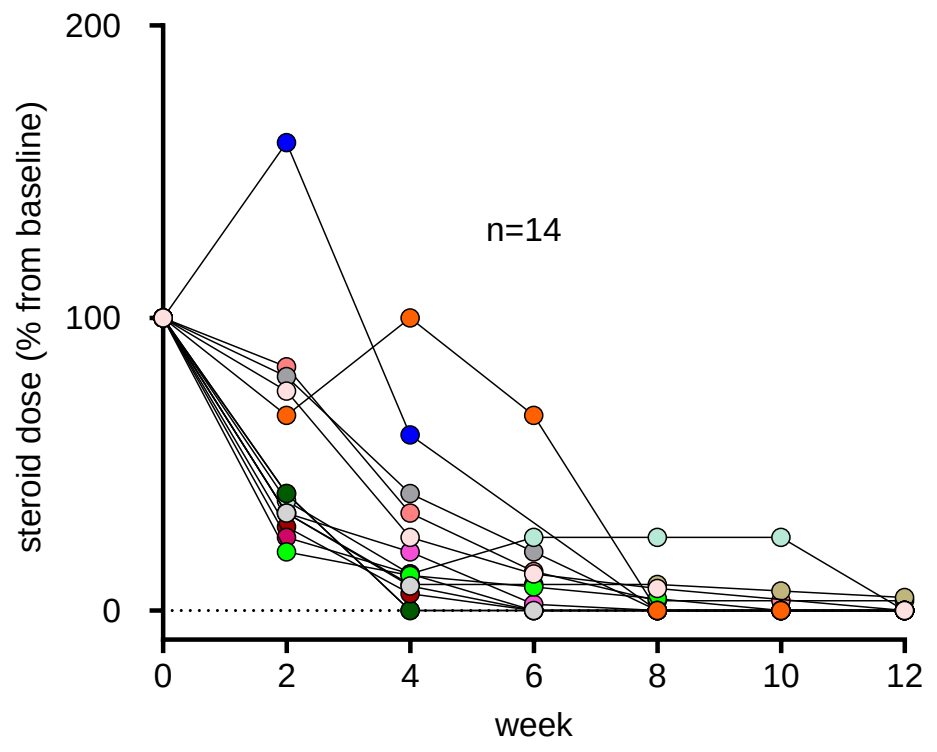

Prednisone dose is shown selectively for the ICI-hepatitis patients.

F: *"what the trends were for transaminases prior to the initiation of ECP (if available)"*

Response: We now analyzed in a post-hoc analysis the ALT levels within 2 weeks prior to ECP start. There was no evidence for a decrease of ALT prior to ECP in the majority of the patients. This is now shown now in Suppl. Figure 5D.

G: *"As is, I would characterize this as promising, but not definitive, and would highlight the number of patients with clear resolution of hepatitis and the time to resolution in the data description."*

Response: We agree and now provide separately the number of patients with clear resolution of hepatitis and the time to resolution in the results part. This was done as a post-hoc analysis to address the comment of the reviewer.

ALT reached the normal range in 13/17 patients (CR), while it declined but did not reach normal values in 4/17 patients (PR).

Additionally, we provide now the time to resolution in the data description in the results part. We state that "Median time to CR was 4 weeks (range 1-10) and median time to PR was 1 week (range 1-3) in patients with ICI-hepatitis."

As suggested we state in the discussion that our data on ECP for ICI hepatitis are promising, but not definitive.

18. If dermatitis is going to be included in the manuscript, some of the data should be in a main figure. As for now, Figure 5 does not optimally use its space. As with hepatitis, I think the percentage of patients achieving complete remission and the average duration of treatment to achieve remission are necessary to highlight in the main text along with other cotreatments.
Response: We agree and now present the percentage of patients achieving complete remission and the average duration of treatment to achieve remission. No other treatment than ECP was given. 66.7% (2/3) of ICI-dermatitis patients achieved a complete remission.
The new data is shown in Figure 5R and 5S.

19. I think that it is very promising that the number of patients with progressive disease did not increase with ECP. That said, the authors should be careful about reading too much into such a small study in the language that they use to describe these clearly preliminary results.
Response: We thank the reviewer for stating that it is very promising to see that the number of patients with progressive disease did not increase with ECP. We agree and now tone down our interpretation of the observation that the number of patients with progressive disease did not increase with ECP.

20.
A: The ctDNA testing is a bit misleading as described. Most of the patients had undetectable or nearly undetectable disease at baseline. We can't say anything about these samples other than that their burden of disease remained undetectable throughout the study. Of the patients with evaluable disease, one progressed at the ctDNA level.
B: Were any of these patients transitioned to targeted therapy during the study.
Response:
A: *"We can't say anything about these samples other than that their burden of disease remained undetectable throughout the study"*
We agree and now state in the results part that the burden of disease remained undetectable throughout the study.

B: *"Were any of these patients transitioned to targeted therapy during the study"*

40% (4/10) of the patients shown in ctDNA testing received targeted therapy during the ECP treatment period. Two of these patients showed a decrease in tumor size in corresponding imaging methods, while one patient showed a stable tumor control according to RECIST 1.1 (SD in corresponding imaging before and after ECP). The CT based imaging in one patient showed progressive disease according to RECIST 1.1 at both staging timepoints, which also correlated with an increase in ctDNA for the tumor specific mutation during the trial.

21. For safety, how can the authors be sure that the SAE were unrelated to ECP. Might they not be severe consequences of combining ECP with immune-related toxicities?
Response:
The adverse events were reported by the treating physicians of the patients and their relation to treatment was also judged by these physicians, which is according to Good clinical practice (GCP). In our study, each SAE was prospectively recorded and reviewed in detail, including timing, clinical context, and concomitant conditions. Where possible, a clear alternative etiology was established (for example, seizure and hemiparesis due to brain metastases or SARS-CoV-2 infection), and these were explicitly stated in the manuscript.
SAEs were reported to the responsible Pharmacovigilance Clinical Trial Unit according to protocol within 24 hours after occurrence. Each AE and SAE was assessed for causal relationship to ECP by the investigator.
We therefore believe that the described SAEs can reasonably be considered as unrelated to ECP. To make this process of AE/SAE reporting more transparent we state that SAE attribution is based on clinical evaluation.

22. For the discussion, the authors need to tone down their conclusions. As this is an uncontrolled trial with no comparator arm and several methodological concerns as highlighted above. I think they can reasonably claim safety (their primary endpoint), no obvious negative impact on antitumor immunity (though a very small sample size with heterogenous tumors), and evidence of some efficacy in most patients.
A: Clinically, complete responses are really the only meaningful ones for most of these toxicities, and that should be highlighted rather than the overall response rate.
B: I also think that the characterization of alternative ICI colitis treatments does not reflect clinical reality. Mimi Wang's work (multiple publications over a number of years) nicely highlights the response to both infliximab and vedolizumab, which is typically complete, durable, and rapid. This is also the experience of every gastroenterologist I know who sees

these patients clinically. Fewer than 5% of patients require treatment beyond steroids, infliximab and vedolizumab.

C: That doesn't mean that there isn't plenty of room for novel therapies, particularly ones that avoid interfering with the cancer treatment, but ECP should be situated a more realistic framing of current clinical practice.

Response:

***A:** "Clinically, complete responses are really the only meaningful ones for most of these toxicities, and that should be highlighted rather than the overall response rate."*

Response: We now highlight the CR rates as suggested. We state:

CR rates were 56.7% (CI: 37.4-74.5%) and 70.4% (CI: 49.8-86.2%) at week 6 and 12, respectively (**Fig. 2A**).

***B:** "I also think that the characterization of alternative ICI colitis treatments does not reflect clinical reality. Mimi Wang's work (multiple publications over a number of years) nicely highlights the response to both infliximab and vedolizumab, which is typically complete, durable, and rapid."*

Response: We now discuss that the response to both infliximab and vedolizumab is typically high and cite Mimi Wang's work in the discussion. We now state that

"Response to both infliximab and vedolizumab is typically high as reported by the group of M. Wang."

We had already included the following publications by Dr Wang in the initial version of the manuscript:

Zou, F., Faleck, D., Thomas A, Harris J, Satish D, Wang X, Charabaty A, Ernstoff MS, Glitza Oliva IC, Hanauer S, McQuade J, Obeid M, Shah A, Richards DM, Sharon E, Wolchok J, Thompson J, **Wang Y**. Efficacy and safety of vedolizumab and infliximab treatment for immune-mediated diarrhea and colitis in patients with cancer: a two-center observational study. J Immunother Cancer. 11, e003277 (2021)

Abu-Sbeih, H., Ali, F.S., Alsaadi D, Jennings J, Luo W, Gong Z, Richards DM, Charabaty A, **Wang Y**. Outcomes of vedolizumab therapy in patients with immune checkpoint inhibitor-induced colitis: a multi-center study. *J Immunother Cancer.* **6**, 142 (2018).

We now included the following additional publications as recommended:

Wang, Y., Abu-Sbeih H, Mao E, Ali N, Ali FS, Qiao W, Lum P, Raju G, Shuttlesworth G, Stroehlein J, Diab A. Immune-checkpoint inhibitor-induced diarrhea and colitis in patients with advanced malignancies: retrospective review at MD Anderson. J Immunother Cancer. 6, 37 (2018).

Harvey C, Nahar KJ, McKeown J, Lo SN, Farag S, Yousaf N, Young K, Tas L, Meerveld-Eggink A, Blank C, Thomas A, McQuade J, Schilling B, Johnson DB, Huertas RM, Arance A, Lee J, Zimmer L, Long GV, Carlino MS, Wang Y, Menzies AM. Management of infliximab refractory immune checkpoint inhibitor gastrointestinal toxicity: a multicenter case series. J Immunother Cancer. 2024 Jan 31;12(1):e008232. doi: 10.1136/jitc-2023-008232. PMID: 38296594; PMCID: PMC10831444.

C: *"That doesn't mean that there isn't plenty of room for novel therapies, particularly ones that avoid interfering with the cancer treatment, but ECP should be situated a more realistic framing of current clinical practice."*

Response: We thank the reviewer for stating that there is plenty of room for novel therapies, particularly ones that avoid interfering with the cancer treatment.
We now state we observed safety of ECP (the primary endpoint), no obvious negative impact on antitumor immunity, limited by a small sample size with heterogenous tumors, and evidence of some efficacy in most patients.

We hereby confirm that all authors concur with the resubmission.
Thank you for your time and consideration.

Sincerely

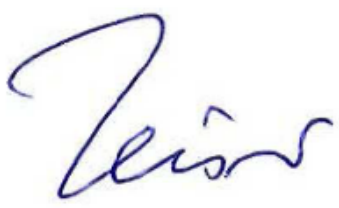

Robert Zeiser, M.D.

Manuscript entitled ‘Extracorporeal photopheresis for steroid-refractory immune related adverse events after immune checkpoint inhibitor therapy‘

Reference: NCOMMS-26-018123A

Reviewer #1 (Remarks to the Author):

As Reviewer #1 on the original submission, I praised the significance of the authors' findings as potentially field-shaping in the evolving realm of cancer immunotherapy. They demonstrated the dual efficacy of ECP in replacing standard nonspecific corticosteroids in reversing severe adverse autoimmune reactions to immuno-stimulatory checkpoint inhibitors (CPIs), thereby releasing or even enhancing the desirable generation of anti-tumor immunity. The deficiency which I identified was that, by framing ECP so succinctly as merely an "immunomodulatory" agent, the authors inadvertently diminished the traction of their own message, since the audience at large could underestimate how logical basis of their findings and their potential importance. In their response to that critique and their modifications of the manuscript, the authors impressively and completely resolve that key deficiency. They highlight, with appropriate referencing, why ECP, through its generation of platelet-signaled conversion of monocytes to physiologic dendritic antigen-presenting cells, and resultant T cell responses, may be tailor-made to safely and simultaneously accomplish the above-mentioned goals. Since therapeutic use of CPIs, and other anti-cancer immunostimulants, can be limited by adverse induction of autoimmunity, ECP may offer a prototype scientifically based adjunctive therapy. Therefore, I am fully satisfied with the revised manuscript.

Response: We are thankful to read that the reviewer is fully satisfied with the revised manuscript.

Reviewer #2 (Remarks to the Author):

I appreciate the care that the authors have taken in addressing my comments. I do still have several substantive concerns that I believe should be addressed prior to publication.

Response: We thank the reviewer for the positive statement and now address the remaining concerns.

General comments

1. Throughout the text, the authors infer causality, for example with language such as “ECP reduces” or “ECP induces.” This isn’t really appropriate for a single arm study. Instead the authors should use language that emphasizes correlation “ECP is associated with” throughout the text. I also think that for conclusions based on combination treatment with steroids and ECP, the concluding or summarizing comments should read “Addition of ECP to systemic steroids…”

Response: We have now changed the wording and state in the results and discussion:

“Treatment with ECP was associated with …” (results part)

“Addition of ECP to systemic steroids…” (discussion, last sentence)

2. The discussion remains far too expansive in its conclusions with the tempering comments added as side notes to the end of overly ambitious interpretations of the data, rather than as replacements for these statements. I believe that the discussion should start with “In this single arm trial, we observed safety of ECP without an obvious negative impact on antitumor immunity, though these findings are limited by a small sample size with heterogenous tumors.” A discussion of the safety data in more detail but toned down from the way it currently reads could then follow. After discussing the safety data, the authors could reasonable discuss the efficacy data, which should start with a summary “We found evidence of some efficacy in most patients treated…”

Response: We have now modified the discussion and start with the sentence:

“In this single arm trial, we observed safety of ECP without an obvious negative impact on antitumor immunity, though these findings are limited by a small sample size with heterogenous tumors.”

We also changed the text of the discussion and now state:

“We found evidence of some efficacy in most patients treated…”

Minor comment

3. **A:** I still have several concerns with the flow cytometry data. My main concern is that “stain

minus one” as a control is really not adequate for macrophages, which express high levels FcR, and an isotype control is really necessary. For the macrophage data, the authors should have gated on CD11b+CD3- cells to avoid the possibility of lymphocyte contamination, particularly as the CD11b stain does not appear to be very strong, and thus identifying a clear macrophage population is not really possible.

B: The gates are also drawn very tightly for Tbet and Ki67, clearly including some cells that are in the negative population. This likely would not change the overall conclusions, but I do think that the numbers reported are not accurate.

Response:

A: For the macrophage data, the authors should have gated on CD11b+CD3- cells …

Already in the initial version of the manuscript, we had blocked the FcR on myeloid cells using the FcR blocking reagent (Miltenyi, 130-059-901). Nevertheless, we have now repeated the flow-cytometry experiment using the cryo-preserved patient samples to control for lymphocyte contamination in the myeloid population. For this experiment, we followed the reviewer’s suggestions and used an isotype control antibody for CD11b, together with an FMO control and staining for lymphocytes. The myeloid gate (CD11b) is now set on $CD11b^{+}$ $CD3^{-}CD19^{-}$ cells. Thereby, we avoid lymphocyte contamination.

In the revised version of the manuscript, we have replaced the prior data with the new data in Figure 4 and Suppl. Figure 4. The updated gating strategy for the analysis of myeloid cells is shown in Suppl. Figure 11.

B: The gates are also drawn very tightly for Tbet and Ki67, clearly including some cells that are in the negative population. This likely would not change the overall conclusions, but I do think that the numbers reported are not accurate.

The gates were set based on the Fluorescence Minus One (FMO), according to flow cytometry standards. A Fluorescence Minus One (FMO) control is a sample stained with all antibodies in our panel except the one we are testing. It measures the background signal, or "spread," created by other colors. FMOs help to set precise gates to tell true positive cells from background noise

This was described in: Sun J, Kroeger JL, Markowitz J. Introduction to Multiparametric Flow Cytometry and Analysis of High-Dimensional Data. Methods Mol Biol. 2021;2194:239-253. doi: 10.1007/978-1-0716-0849-4_13. PMID: 32926370; PMCID: PMC7868168.

To underline the differences reported for the expression of T-bet and Ki67, we now additionally provide the mean fluorescence intensity (MFI) for T-bet in $CD4^+$ T cells, and Ki67 in both $CD4^+$ and $CD8^+$ T cells. This is independent of gating these markers, but just shows the expression of these molecules in the cells. In the revised version of the manuscript, we provide both, frequencies and MFI data. We believe that it becomes evident that the expression of both markers is reduced after ECP treatment. The new data were included in Suppl. Figure 3 and Suppl. Figure 4.

Comments on responses to prior review

Response to point 5.

A: I do think that these numbers belong in the main text, as they are more easily compared with standard practice metrics.

B: I also think that "intention to treat" is really the correct population to display, so the 3 patients who did not fully complete treatment should be included in the response rate.

Response:

"A: I do think that these numbers belong in the main text, as they are more easily compared with standard practice metrics."

Response: This relates to point 5 of the first revision on the "steroid free" response rates.

We already had included this in the main text as requested here.

"B: I also think that "intention to treat" is really the correct population to display, so the 3 patients who did not fully complete treatment should be included in the response rate. "

We have now included all 30 patients in the steroid free response rate in an additional intend to treat analysis. We now state:

"We performed a post-hoc analysis for the steroid free remission rate including also patients who prematurely exited the study. The steroid free response rate including all patients was 40% (12 / 30 patients) at week 6 and 76.6% (23 / 30) patients at week 12."

Response to point 6.

The literature cited to support the 4 week duration is old in a rapidly advancing field. Contemporary treatment guidance at most high volume centers (including the centers responsible for several of the cited papers) is to start secondary immune suppression at day 3 for non responders to steroids and within a week for partial responders. In the cohorts highlighted, most of these patients were on steroids for a prolonged period of time precisely because the treating physician believed that the steroids were having an effect, even if the grade of diarrhea had not yet changed. The 4 week duration of steroids pre-secondary intervention makes it very difficult to compare ECP to standard of care therapies (anti-TNF or anti-integrin biologics). That doesn't negate the findings of this study, but it does temper my enthusiasm for a potential clinical impact.

Response: We thank the reviewer for making this point and now include the following text in the discussion:

"In contrast to the long steroid treatment in the patients included in this trial, contemporary treatment guidance at most high-volume centers is to start secondary immune suppression at day 3 for non-responders to steroids and within a week for partial responders."

Response to point 7.

For colitis, I am still confused why so few patients on this study received standard second or third line therapy. The problem with this, from my perspective, is that in a non randomized study where we are also not privy to the total number of eligible patients at the participating centers, it is likely that a group of people who are initially treated very differently from standard practice were treated differently for a biologically meaningful reason, even if we do not know what that reason was.

Response: We agree that only a randomized trial can answer if there is a biological reason for some patients being treated longer with steroids and if that has impacted the response to ECP versus how this would impact the response to other second line treatments.

To account for this, we state in the discussion:

"Only a randomized trial can answer if there is a biological reason for some patients being treated longer with steroids and if that has impacted the response to ECP versus how this would impact the response to other second line treatments."

Response to point 8.

I am surprised that so few patients were considered at high risk for a biopsy. The risk of flexible sigmoidoscopy with biopsy is miniscule (estimated at 0.02%, much lower than the risk of an incorrect diagnosis which is closer to 20% in this population). I do think that this is a significant

limitation because of my concern raised in the above point (#7). Perhaps this was a cohort that had a substantial number of patients where the diagnosis of colitis was incorrect to begin with.
Response: 3 of 13 (23.0 %) patients with grade 2-4 ICI colitis did not receive a biopsy (1 with grade 2, one with grade 3 and one with grade 4). 77% of the patients therefore had a histologically confirmed ICI colitis diagnosis. The remaining 23% (n=3) had the typical clinical signs of colitis and other etiologies were excluded (infection, toxicity).

The ASCO guidelines, page 4081 (Schneider et al JCO 2021) states: "… colonoscopy should be considered …"

This indicates that colonoscopy is recommended but not mandatory.

Response to point 10.

As mentioned above, patients who die during the study or who prematurely exit the study should be included in the steroid free remission rate. They did not have a remission, maybe for no reason related to ECP or maybe because of ECP. Removing them isn't really appropriate because it makes the response rate look higher by removing treated patients who did not improve. If this was the prespecified analysis, removing these patients, then I think a posthoc analysis that does include them should be within the main text.
Response: We have now included patients who died during the study or who prematurely exit the study in the steroid free remission rate. These data were included in the results part:

At week 6, the steroid free response rate including all patients was 40% (12 / 30 patients).

At week 12, the steroid free response rate including all patients was 76.6% (23 / 30 patients).

We now state:

"We performed a post-hoc analysis for the steroid free remission rate including also patients who prematurely exited the study. The steroid free response rate including all patients was 40% (12 / 30 patients) at week 6 and 76.6% (23 / 30) patients at week 12."

Response to point 11.
The primary endpoint needs to be reported first, both in the abstract and in the results. It is the primary endpoint of the study. Key secondary endpoints that are prespecified are important, but they are not the primary endpoint of the study, and by placing them first it could lead less careful readers to be confused about how the study was designed. This is, ultimately, a safety study.

Response: We have changed the order in the text and now report the primary endpoint first, both in the abstract and in the result.

Response to point 12.

I agree with the authors, the summary data is useful, but so is the individual data and the choice to include it as a supplement was a good one.

Response: Thank you for agreeing that we show both presentations of the data.

Response to point 14.

I agree with the authors that the study design is appropriate for a phase I/II study with safety as the primary endpoint. My concern is that the way that the paper is written and the data are presented, it is not sufficiently emphasized that all of the efficacy inferences are ultimately exploratory and require confirmation in an appropriately designed trial (as is planned). I think that this emphasis needs to appear repeatedly throughout the text, because the authors should be weary of encouraging use of an unproven therapy prior to completion of the phase 2 study based on data that, while intriguing, clearly does not meet the clinical bar for demonstrated efficacy.

Response: We have now emphasized that our phase I/II study has safety as the primary endpoint and that efficacy inferences are ultimately exploratory.

We include this in the results part and in the discussion part:

"This phase I/II study has safety as the pre-defined primary endpoint and efficacy inferences are ultimately exploratory."

Response to point 15.

I disagree with the authors here. The fact that steroids were continued leaves them as a reasonable contributing factor, particularly as we would expect ~50% of patients to eventually respond to steroids over a 6 week period (which is what published data suggest, and guidelines recommend 6-8 week steroid courses). Because a response to steroids remains plausible, and no control group is included, the correlate data are ultimately irrelevant – they only demonstrate that patients did get better, not why they did. In my mind, this concern is further increased by the factors highlighted above that make me concerned that this cohort is skewed toward a

group that may be more likely to respond to steroids late – i.e. absence of universal biopsies, prolonged period of time on steroids without the initiation of secondary immune suppression.
Response: We now temper our statement and state that ECP was “associated with” clinical responses and a decrease of pathogenic T cells.

We state in the discussion:

“In some patients the corticosteroids given as first line therapy could have contributed to the clinical response under ECP.”

Response to point 17.
B – you are correct about the ASCO and ESMO guidelines, though that does not change the fact that 10% of patients with suspected ICI hepatitis do not, in fact, have evidence of ICI hepatitis on biopsy. This is likely a higher fraction of patients who do not respond to steroids in my clinical experience (PMID: 34554206). This is why other guidelines, for example GI society guidelines, look more favorably on biopsy.
Response: We agree and now state that liver biopsy is recommended by the GI society guidelines.

“Other guidelines, for example GI society guidelines, look more favorably on liver biopsy.”

In addition, we cite the following publication:

Li M, Sack JS, Bell P, Rahma OE, Srivastava A, Grover S, Zucker SD. Utility of Liver Biopsy in Diagnosis and Management of High-grade Immune Checkpoint Inhibitor Hepatitis in Patients With Cancer. JAMA Oncol. 2021 Nov 1;7(11):1711-1714. doi: 10.1001/jamaoncol.2021.4342. PMID: 34554206; PMCID: PMC8461544.

F – it would be useful to include the percentage of patients with stable transaminases prior to initiation of steroids in the main figure.
Response: We have now analyzed the percentage of patients with stable versus increasing transaminases prior to initiation of glucocorticoids (GC; steroids). We observed that the ALT values measured prior to the initiation of GC were increasing in 28/30 (93.3%) and not available in 2/30 patients (6.6%).

These data were collected before study inclusion and are therefore classified as a post-hoc analysis.

The new data are shown in main Figure 5R.

“In a post-hoc analysis, we observed that the ALT values measured prior to the initiation of GC were increasing in 28/30 (93.3%) patients, while they were not available in 2/30 (6.6%) patients (Figure 5R).”

G – the time to response remains long enough for the steroids to be a major contributing factor. This does need to be acknowledged in the discussion.

Response: We have now included this in the discussion:

“In some patients the corticosteroids given as first line therapy could have contributed to the clinical response under ECP.”

Response to point 20.

A – I actually think that the authors need to state that the cDNA testing was non diagnostic, and remained undetectable throughout the study in nearly all patients. It is important to emphasize that we cannot conclude anything about cancer related safety from these data one way or the other. It is possible that every patient’s cancer got worse going from 1000 fold below the limit of detection to 10 fold below the limit of detection. It is also possible that none of these patients had any cancer at all at the time of the study. The opposite is also true of course, maybe everyone’s cancer burden was improving throughout the study. Since we have no way of knowing, the data really have to be characterized as non diagnostic.

Response: We now state in the results part “The ctDNA monitoring was performed as an additional non-diagnostic readout, and ctDNA levels remained undetectable for nearly all patients throughout the observation period.”

B – the fact that 40% of these patients received an alternative cancer treatment is also important to emphasize, since this could mask an effect on antitumor immunity.

Response: We agree and now state that:

“40% (4/10) of the patients shown in ctDNA testing received targeted therapy during the ECP treatment period.”

Response to point 21.

I think that this explanation is very thorough. Since this is a safety study, I believe that essentially this entire text belongs in the main text of the article.

Response: We have now included this in the main text:

“The adverse events were reported by the treating physicians of the patients and their relation to treatment was also judged by these physicians, which is according to Good clinical practice (GCP). No ECP-related SAE was observed. 105 AEs were documented (Suppl. Table 2). The number of patients with possibly ECP-related AEs were 6/30. ECP-related AEs included hypopigmentation (1/6), exanthema (1/6), and photosensitivity (3/6), and anemia (1/6), of which 33.3% resolved completely. In our study, each SAE was prospectively recorded and reviewed in detail, including timing, clinical context, and concomitant conditions. Where possible, a clear alternative etiology was established. Ten SAEs were documented in 6 patients, which had no causal relationship with ECP. These SAEs included SARS-CoV2 infection, intestinal infection, cholestasis (2 events), gangrenous cholecystitis, hemorrhagic colitis, hemorrhagic shock, sepsis and hemiparesis and seizures due to known brain metastases. All SAEs resolved completely. Individual AEs and SAEs are shown (Suppl. Table 3). SAE attribution to ECP or not was based on clinical evaluation. SAEs were reported to the responsible Pharmacovigilance Clinical Trial Unit according to protocol within 24 hours after occurrence. Each AE and SAE was assessed for causal relationship to ECP by the investigator.”

We hereby confirm that all authors concur with the resubmission.
Thank you for your time and consideration.

Sincerely

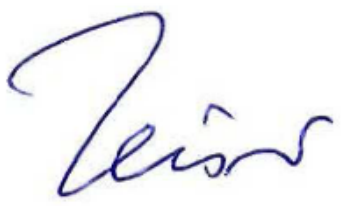

Robert Zeiser, M.D.
